# Comparative Genomic Analysis of Asian Cultivated Rice and Its Wild Progenitor (*Oryza rufipogon*) Has Revealed Evolutionary Innovation of the Pentatricopeptide Repeat Gene Family through Gene Duplication

**DOI:** 10.3390/ijms242216313

**Published:** 2023-11-14

**Authors:** Li-Ying Feng, Pei-Fan Lin, Rong-Jing Xu, Hai-Qi Kang, Li-Zhi Gao

**Affiliations:** 1 Institution of Genomics and Bioinformatics, South China Agricultural University, Guangzhou 510642, China; lyfengad@163.com (L.-Y.F.); lamevan0754@gmail.com (P.-F.L.); 2 Tropical Biodiversity and Genomics Research Center, Hainan University, Haikou 570228, China; Xuuuurj@163.com (R.-J.X.); kanghq@126.com (H.-Q.K.)

**Keywords:** rice, *Oryza*, pentatricopeptide repeat, innovation, evolution, segmental duplication

## Abstract

The pentatricopeptide repeat (*PPR*) gene family is one of the largest gene families in land plants. However, current knowledge about the evolution of the *PPR* gene family remains largely limited. In this study, we performed a comparative genomic analysis of the *PPR* gene family in *O. sativa* and its wild progenitor, *O. rufipogon*, and outlined a comprehensive landscape of gene duplications. Our findings suggest that the majority of *PPR* genes originated from dispersed duplications. Although segmental duplications have only expanded approximately 11.30% and 13.57% of the *PPR* gene families in the *O. sativa* and *O. rufipogon* genomes, we interestingly obtained evidence that segmental duplication promotes the structural diversity of *PPR* genes through incomplete gene duplications. In the *O. sativa* and *O. rufipogon* genomes, 10 (~33.33%) and 22 pairs of gene duplications (~45.83%) had non-*PPR* paralogous genes through incomplete gene duplication. Segmental duplications leading to incomplete gene duplications might result in the acquisition of domains, thus promoting functional innovation and structural diversification of *PPR* genes. This study offers a unique perspective on the evolution of *PPR* gene structures and underscores the potential role of segmental duplications in *PPR* gene structural diversity.

## 1. Introduction

Since the discovery of the pentatricopeptide repeat (PPR) protein motif 23 years ago [1], this protein family has played an immense role in plant organelle biology [2]. As one of the largest gene families reported in land plants [3], it is exceedingly varied in land plants with hundreds or even thousands of members [4], each of which seems to have definite targets in the transcriptomes of chloroplasts or mitochondria. *PPR* genes are characterized by the presence of pentatricopeptide repeat motifs, which are approximately 35 amino acid sequences that often form a helical structure [5,6,7,8]. These repeat motifs are responsible for protein–protein interactions and binding to target RNA molecules [9,10,11,12]. The *PPR* gene family is involved in various biological processes, including post-transcriptional regulation of gene expression [2,13,14], RNA editing [15,16], RNA splicing [17,18], and RNA stability [19]. In plants, PPR proteins play critical roles in organelle gene expression, particularly in chloroplasts and mitochondria [16,17,20].

PPR proteins are often classified into two major subfamilies, P and PLS, based on the characteristic properties of their PPR motifs [21,22]. The P subfamily is composed of multiple P motifs, the involvement of P-class PPR proteins in RNA stability, processing, and mRNA translation initiation [18,23,24], whereas the PLS subfamily is unique to land plants and typically encompasses P motifs along with two derived variants, namely S motifs (short) and L motifs (long). Moreover, the majority of PLS subfamily members are characterized by the presence of additional C-terminal domains, including E, E+, and DYW domains [25]. Meanwhile, the primary function assigned to PLS-class PPR proteins is the C-to-U editing of organellar transcripts [26].

Heterosis, widely applied in crops, aims to enhance yield potential and improve quality by harnessing the phenomenon of hybrid vigor [27,28]. The male sterility of plants is induced by the Cytoplasmic Male Sterility (CMS) genes in the mitochondria, whereas fertility restoration is achieved through the interaction of *RF* genes in the nucleus. Thus, the CMS genes in the mitochondria result in male sterility, while the *RF* genes in the nucleus act reciprocally to restore plant fertility [29,30,31]. The majority of known restorer genes belong to the P-class of the PPR protein family [32,33].

The first comparative genomic analyses of *Arabidopsis thaliana* and *Oryza sativa* and *Physcomitrella patens* revealed the expansion of the *PPR* gene family, becoming a huge family in the two flowering plants (450 members in *A. thaliana* and 477 in rice) in sharp contrast to 103 *PPR* genes in the moss [7,34,35]. The availability of an increasing number of plant genomes permits one to recognize the *PPR* gene family to be one of the largest families in angiosperms. The lowest number of *PPR* genes is reported in *Medicago truncatula* (365), while the highest number is found in *Glycine max* (629). The partially assembled *Selaginella moellendorffii* (spikemoss, lycophyte) genome is suggested to contain >1000 *PPR* genes [36]. It is clear that a wide range of angiosperms have a large number of *PPR* genes whilst the moss *Physcomitrella patens* [37] contains 103 [34,35], and the green alga *Chlamydomonas reinhardtii* has only 12 *PPR* genes [38]. These results suggest that the initial expansion of *PPR* genes occurred after the separation of the land plants from green algae such as *Chlamydomonas* but before the divergence of seed plants from bryophytes [36]. Recent comprehensive comparative transcriptomic analysis of OneKP datasets including over 1000 transcriptomes from diverse plants and algae has established a clear picture of the evolution of this massive gene family [4]. They interestingly found that, across the plant kingdom, rather than a single expansion, most land plant lineages with high numbers of editing factors have continued to generate novel sequence diversity.

The *PPR* gene family provides an unprecedented opportunity to understand the evolutionary nature of driving a 50-fold expansion of such a single-gene family. Up to 80% of the *PPR* genes in *A. thaliana* and rice were intron-less [7,34,39], while >75% of *P. patens PPR* genes contained introns. Evolutionarily older *PPR* genes in rice and *A. thaliana* had more introns [34] and some of the putative orthologues to the *P. patens* intron-containing genes lack introns in angiosperms [34]. Such results offer compelling evidence that one or more waves of retrotransposition were responsible for the expansion of the *PPR* gene family in flowering plants [34]. Thus, the duplication of genomic sequences stands as one of the fundamental mechanisms for generating novel genes [40,41,42]. Gene duplication, which mainly originates from unequal crossing over, retrotransposition, and segmental duplication, has been reported to play a critical role in gene family expansion [36,43,44]. Gene families can also increase in size by whole-genome duplication events. Angiosperm genomes have undergone multiple rounds of whole-genome duplication events [45,46], which have a great impact on the number of large gene families. It was suggested that *PPR* gene content seemed quite conserved between *A. thaliana* and rice [34] and that the *PPR* genes duplicated by independent whole-genome duplication events were rarely retained [36]. However, current knowledge about the evolution of the *PPR* gene family remains largely limited, particularly in the context of the role of gene duplications.

It deserves mentioning that segmental duplications (SDs) are large (≥1 kb), nearly identical copies of genomic DNA sequences that map to two or more genomic locations [47,48,49]. These duplications can encompass various genomic elements, including gene sequences and common repetitive elements, and can be organized either in tandem or dispersed throughout the genome [50]. In plants, the impacts of segmental duplications include genome size expansion, nuclear DNA structure modeling, and adaptive evolution [51,52,53]. Many studies have explored how SDs contribute to the emergence of new genes especially in the evolution of primate genomes [54,55,56]. Not only the duplication of an entire gene but also the exon shuffling and fusion transcripts that result from juxtaposing unrelated SDs appear to be the mechanism by which SDs give rise to novel genes [50,57]. In addition, due to the high sequence identity, duplication serves as the foundation for subsequent rearrangements facilitated by non-allelic homologous recombination. Thus, it is expected to aid in gaining novel insights into the structural complexity of *PPR* genes, which will provide more evidence for us to further understand the evolution of this gene family.

In this study, we performed genome-wide identification and characterization of the *PPR* gene family in *O. sativa* and its wild progenitor, *O. rufipogon*. In addition, we presented a comparative genomic analysis of these two rice genomes and outlined a comprehensive landscape of gene duplications towards understanding the molecular evolutionary mechanisms of *PPR* genes. We finally obtained a full map of segmental duplications that greatly enhance structural variation and the innovation of *PPR* genes through incomplete gene duplications. The findings will also form a solid foundation for future functional studies on *PPR* genes such as *RFL* genes of agronomical importance for rice genetic improvement programs.

## 2. Results

### 2.1. Identification and Classification of PPR Genes in the O. sativa and O. rufipogon Genomes

**Figure 1:**
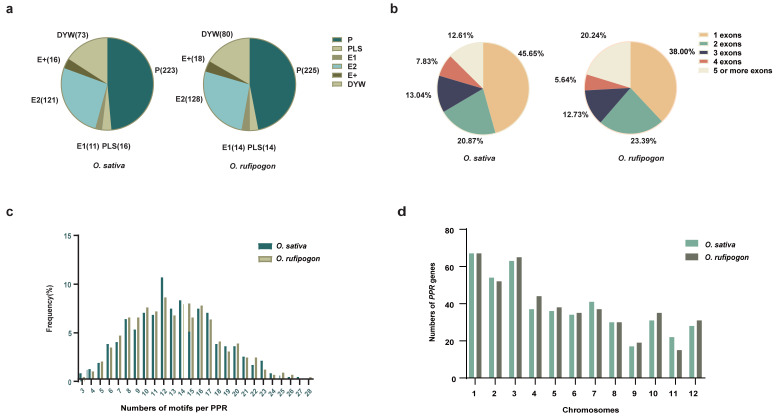
Classification of the *PPR* genes in *O. sativa* and *O. rufipogon*. (**a**) PPR protein numbers in *O. sativa* and *O. rufipogon*; (**b**) the distribution of exon numbers in two rice species; (**c**) the motif quantity of *PPR* genes in two rice species; (**d**) the distribution and quantity of *PPR* genes across 12 chromosomes in two rice species.

In this study, a total of 460 and 479 *PPR* genes were identified in *O. sativa* and *O. rufipogon*, respectively (Figure 1a). Based on the protein structure, 460 PPR proteins consisted of 223 P-class and 237 PLS-class subfamilies in *O. sativa*. There were 148 members in the E subgroup and 73 members in the DYW subgroup within the PLS subfamily. In *O. rufipogon*, the 479 PPR proteins comprise 225 P-class and 254 PLS-class members. In the PLS subfamily, there were 160 members in the E subgroup and 80 members in the DYW subgroup. Information regarding the physical lengths, classification, and protein lengths of these genes can be found in Tables S1 and S2.

Previous findings suggested that *PPR* genes are predominantly composed of a single exon, accounting for the largest proportion [2,8]. The gene structure analysis indicated that in *O. sativa*, 45.65% of the genes had a single exon, while 20.87%, 13.04%, 7.83%, and 12.61% of the genes had two, three, four, and five or more exons, respectively (Figure 1b; Table S1). Our results showed that in *O. rufipogon*, 38.00% of the genes had a single exon, and 23.39%, 12.73%, 5.64%, and 20.25% of the genes had two, three, four, and five or more exons, respectively (Figure 1b; Table S2). Overall, the PLS subfamily gene is more abundant than the P subfamily gene in terms of individual exons of the two species. In *O. sativa*, there were 128 PLS subfamily genes and 82 P subfamily genes, while *O. rufipogon* harbored 106 PLS subfamily genes and 76 P subfamily genes (Figure S1a). The characteristic feature of PPR proteins was the tandem array of PPR motifs. For both rice species, the number of PPR motifs per protein ranged from 3 to 28, with the highest number of proteins having 12 motifs (Figure 1c; Tables S1 and S2).

**Figure 2:**
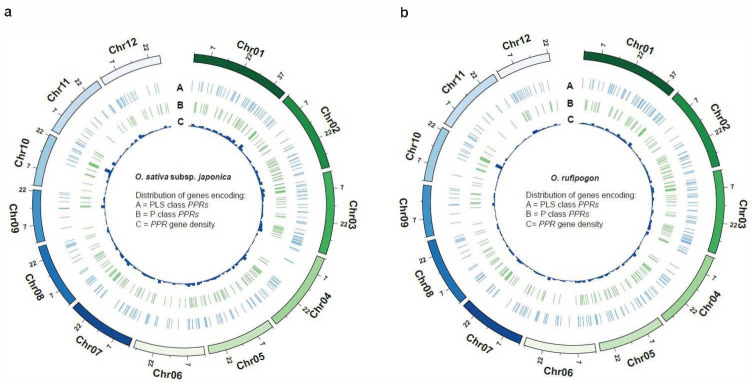
Genome-wide distribution of *PPR* genes. The density of *PPR* genes is presented along each chromosome in 2 Mb windows. (**a**) *O. sativa*; (**b**) *O. rufipogon*.

It is noteworthy that the number of *PPR* genes with a single exon in *O. rufipogon* displayed a significant decrease compared to cultivated rice, while the number of *PPR* genes with five or more exons exhibited a significant increase. There were no significant changes observed in the quantities of PPR proteins with other numbers of exons. The results indicated that *PPR* genes in these two species were extensively and unevenly distributed across the 12 chromosomes (Figure 1d; Table S1). Both rice species exhibited the highest number of *PPR* genes on Chromosome 1; cultivated rice exhibited the fewest number on Chromosome 9, while *O. rufipogon* had the fewest number on Chromosome 11 (Figure 1d; Table S2). Our results showed that the P-subclass and PLS-subclass of the *PPR* genes were widely but unevenly positioned across 12 chromosomes (Figure 2). However, *O. sativa* and *O. rufipogon* exhibited similar distribution of *PPR* genes. Compared to eleven other chromosomes, they were densely distributed on Chromosome 10 for both rice species.

To investigate the regulatory elements of *PPR* genes, we predicted cis-regulatory elements for the promoter sequences of *PPR* genes in *O. sativa* and *O. rufipogon* (Figures S2 and S3). Our results showed that, for both species, the cis-regulatory elements on the promoters of *PPR* genes mainly included hormone response elements, growth and development elements, and stress response elements. Among the cis-acting elements associated with hormone response, both species had the highest number. These elements primarily included abscisic acid response elements (ABRE), the CAAT-box transcription factor, and the core promoter element (TATA-box).

### 2.2. Phylogenetic Analysis of PPR Genes in the O. sativa and O. rufipogon Genomes

**Figure 3:**
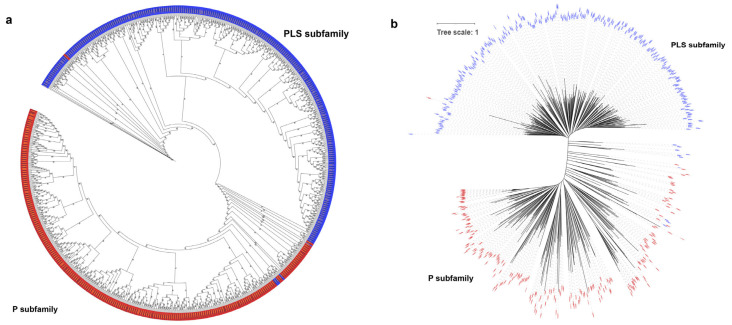
Phylogenetic trees of the *PPR* gene family in *O. sativa* and *O. rufipogon*. The maximum likelihood trees were constructed using IQ-TREE V2.2.3 software with 1000 bootstrap replicates. Blue represents the PLS subfamily, while red symbolizes the P subfamily. (**a**) Phylogenetic tree of *PPR* genes of two species with bootstrap values. The black font represents *O. sativa* and the gold font denotes *O. rufipogon*; (**b**) phylogenetic tree of *PPR* genes of two rice species with branch length. Before constructing the phylogenetic tree, the LG+F+R10 model was determined as the best model.

Based on the full amino acid sequences of *PPR* genes in the two species, a phylogenetic tree was constructed using the Maximum Likelihood (ML) method. The phylogenetic tree was divided into two distinct groups, known as the P and PLS subfamilies (Figure 3a,b). A small subset of P-class genes clustered within the PLS class subfamily, including genes such as *LOC_Os09g26190, RUF029470*, *LOC_Os07g46730*, and *RUF025381*. PLS-class genes also clustered within the P-class subfamily, including *RUF012986*, *LOC_Os03g62400*, *RUF002433*, and *LOC_Os01g42280*. Such an observation is similar to the previous phylogenetic analysis of the *PPR* gene family in poplar [5].

### 2.3. Comparative Genomic Analysis of PPR Gene Families between O. sativa and O. rufipogon

**Figure 4:**
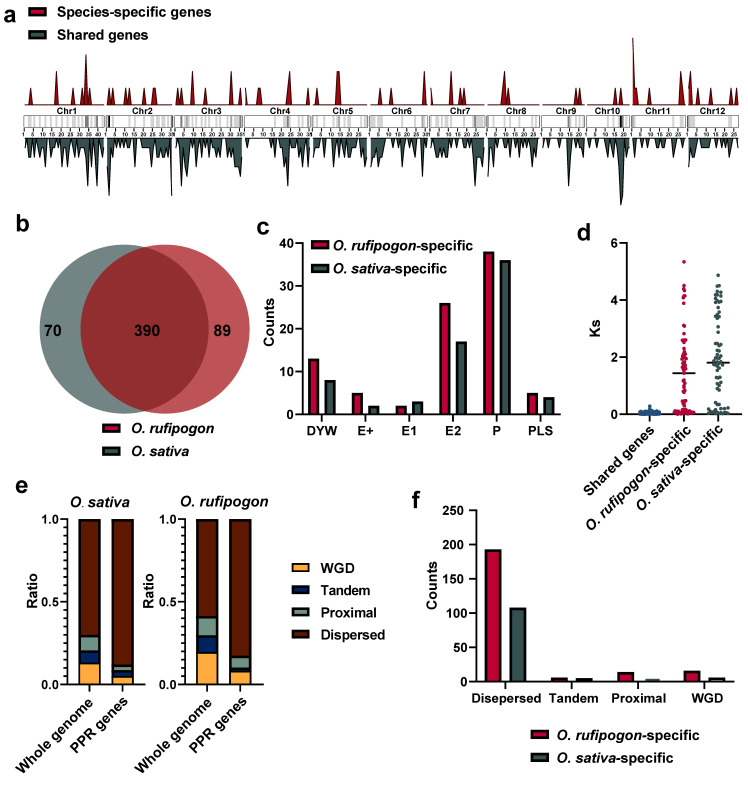
Shared and species-specific genes between *O. sativa* and *O. rufipogon*. (**a**) Chromosomal distribution of shared and species-specific *PPR* genes in the *O. sativa* and *O. rufipogon* genomes. The intensity of chromosome band color indicates the density of *PPR* genes. Red represents species-specific genes, while dark green denotes shared genes; (**b**) number of shared and species-specific *PPR* genes between *O. sativa* and *O. rufipogon*; (**c**) *PPR* subfamilies of specific genes in *O. sativa* and *O. rufipogon*; (**d**) *Ks* values of shared gene pairs and the smallest *Ks* values of paralogous genes for specific genes within their own genomes; © composition of gene duplication types for the whole genome and *PPR* genes in *O. sativa* and *O. rufipogon*; (**f**) composition of gene duplication types of species-specific genes in *O. sativa* and *O. rufipogon*.

To investigate the origins and evolution of *PPR* gene families after the domestication of *O. sativa* from its wild progenitor, we employed a stringent ortholog identification strategy, including specifically reciprocal best-hit and synteny comparisons, to identify shared and specific *PPR* genes between the *O. sativa* and *O. rufipogon* genomes. Our results showed that a total of 390 gene pairs met both criteria. We also identified 10 gene pairs that were located on scaffolds not anchored to any chromosome, and 13 gene pairs were outside syntenic regions (Figure S4). Hence, approximately 84.78% to 89.78% of *PPR* genes were shared between the *O. sativa* and *O. rufipogon* genomes (Figure 4a,b).

The analysis of species-specific *PPR* genes showed that *O. sativa* possessed approximately 70 *PPR* genes, while *O. rufipogon* had 89 (Figure 4b). Statistical analysis was performed on the exons of shared and unique *PPR* genes in the two species. We observed that the largest proportion among the 390 shared genes was the 180 genes with only one exon. Among the 70 unique *PPR* genes in *O. sativa*, the highest proportion of 30 genes contained one exon. Interestingly, out of 89 unique *PPR* genes, 34 had 5 exons with the highest proportion in *O. rufipogon*, surpassing the number of 25 genes with only 1 exon (Figure S1b).

To investigate the nature of species-specific *PPR* genes, we classified them into different subfamilies. Our results showed that the P subfamily harbored the most prominent different genes with 36 and 38 genes in *O. sativa* and *O. rufipogon*, respectively, followed by 17 and 26 genes in the E2 subgroup (Figure 4c). Species-specific genes may arise due to the quality of genome assembly and annotation, or potentially from the loss of one copy of old duplicated genes or newly duplicated genes. After excluding the 10 gene pairs on unanchored sequences and 13 gene pairs outside syntenic regions, we calculated the *Ks* values of these species-specific genes with all paralogous genes in their genomes and selected the minimum *Ks* values. By comparing them with the *Ks* values obtained from each pair of orthologous genes, their origins were determined to have resulted from the loss of one copy of old duplicated genes or the emergence of newly duplicated genes. Our data showed that in *O. sativa*, 17 species-specific genes had *Ks* values lower than the average *Ks* value between orthologous genes. In *O. rufipogon*, 29 species-specific genes had *Ks* values below the average. Thus, it may be estimated that ~36.17% and 42.94% of *PPR* genes originated from new gene duplications in the *O. sativa* and *O. rufipogon* genomes, respectively (Figure 4d). Of them, the wild progenitor, *O. rufipogon*, had more newly duplicated genes than the cultivated rice.

We further examined which types of gene duplications have generated *PPR* genes in *O. sativa* and *O. rufipogon*. Our results showed that, in the *O. sativa* genome, approximately 5.10% of *PPR* genes were derived from whole-genome duplication, only approximately 3.60% of them were from tandem duplications, and around 3.40% of them resulted from proximal duplications, while the majority (~87.99%) of the *PPR* genes were from dispersed duplications. *PPR* genes were remarkably enriched in the dispersed duplication category (~87.99%), which was ~1.26 times higher than the genome-wide average (~69.99%). Similarly, *PPR* genes were outstandingly augmented in the category of dispersed duplication (~82.64%), which was ~1.40 times greater than the genome-wide average (~58.63%) in the *O. rufipogon* genome (Figure 4e; Table S3). Consequently, our findings strongly suggested that the majority of *PPR* genes originated from dispersed duplications for both rice species (Figure 4f).

**Figure 5:**
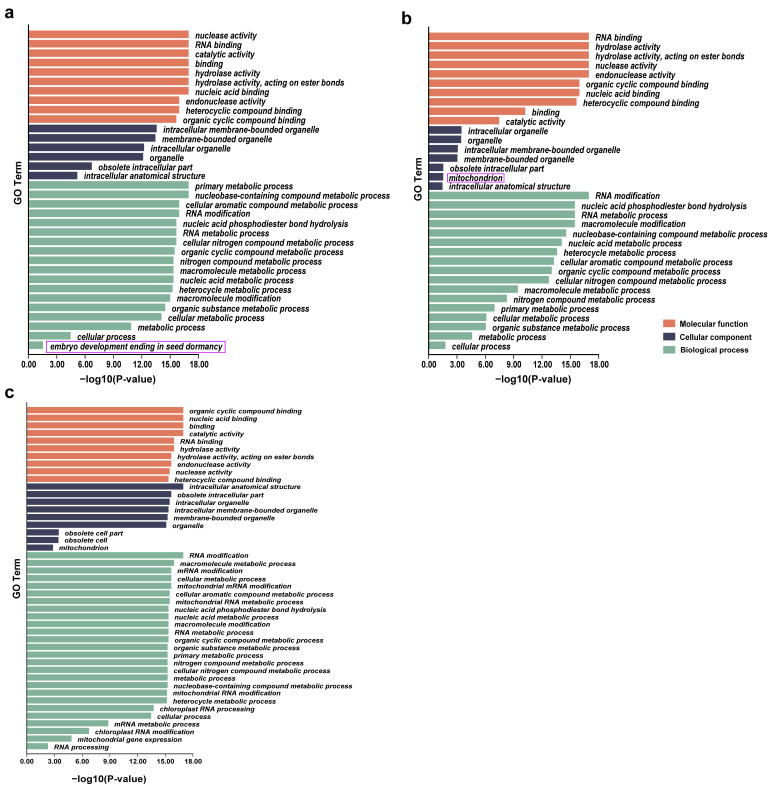
GO enrichment analyses for *PPR* genes. (**a**) *O. sativa*; (**b**) *O. rufipogon*; (**c**) shared *PPR* genes. In the purple box are the pathways specifically enriched by lineage-specific *PPR* genes in two species.

We further conducted Gene Ontology (GO) enrichment analysis of *PPR* genes uniquely identified in *O. sativa* and *O. rufipogon* (Figure 5). Interestingly, *PPR* genes in *O. sativa* exhibited an enrichment of a unique biological process related to the termination of embryo development and seed dormancy, indicating that the presence of specific *PPR* genes may be functionally related to embryo development and reduced seed dormancy during the domestication from wild rice to cultivated rice. The genes involved in this process included *LOC_Os11g03850*, *LOC_Os12g42120*, *LOC_Os03g19650*, *LOC_Os11g43800*, *LOC_Os06g09880*, *LOC_Os02g43080*, and *LOC_Os05g30240* (Figure 5a). It is worth noting that *PPR* genes in *O. rufipogon* displayed enrichment of the mitochondria, as a cellular component. These genes contained *RUF036508*, *RUF037041*, *RUF037004*, *RUF006046*, *RUF002492*, *RUF004777,* and *RUF030818* (Figure 5b). GO enrichment analysis of a total of 390 shared *PPR* genes (Figure 5c) revealed that, compared to unique *PPR* genes, there were two additional components in the cellular component category (intracellular anatomical structure and obsolete cell).

### 2.4. Expression Patterns of PPR Genes in O. sativa and O. rufipogon

We examined the differential expression patterns of these *PPR* genes in the two rice species. To ensure consistency in transcriptome data, we obtained 24 samples across four tissues, including the root, leaf, seedling, and panicle of both rice species during the same growth phase (Figure S5). By mapping the quality-controlled RNA-seq data to their respective genomes, we garnered a comprehensive overview of *PPR* gene expression. Using TPM as a metric, we stratified gene expression into five levels (very high, TPM ≥ 50; high, 10 ≤ TPM < 50; moderate, 2 ≤ TPM < 10; low, 0.1 ≤ TPM < 2; and very low, 0 < TPM < 0.1). Our findings indicate that the majority of *PPR* genes in the seedling and root tissues exhibited an expression level exceeding the moderate range (*O. sativa*: ~ 65.87% in seedling, ~66.96% in root; *O. rufipogon*: ~58.87% in seedling, ~69.51% in root), while only ~10.65% and ~26.93% of *PPR* genes exhibited an expression level exceeding the moderate range in the leaf tissue of *O. sativa* and *O. rufipogon* (Figure S6). A pronounced disparity in *PPR* gene expression surfaced in the panicle tissue, particularly in *O. rufipogon*, which exhibited elevated expression levels (Figure S6).

**Figure 6:**
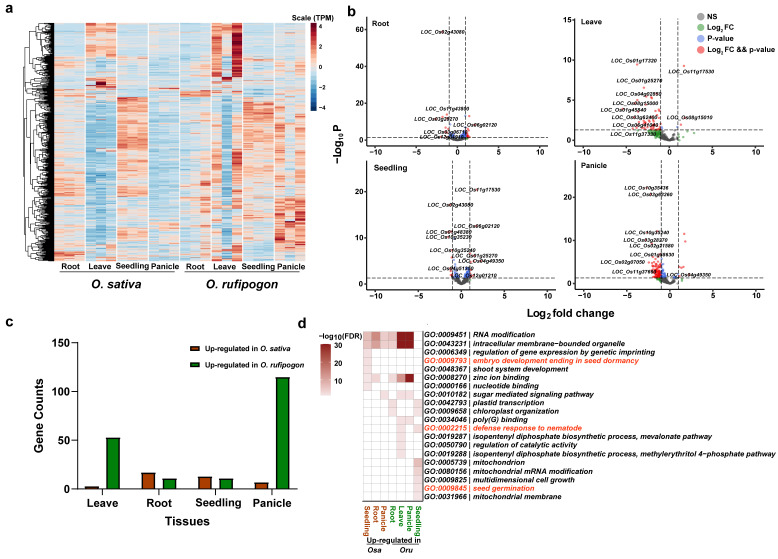
Expression patterns and DEGs of *PPR* genes in *O. sativa* and *O. rufipogon*. (**a**) Heatmap of *PPR* gene expression in four tissues of *O. sativa* and *O. rufipogon*. The four tissues include leaves, roots, seedlings, and panicles, each with three biological replicates. Heatmap was plotted based on normalized TPM values. (**b**) Volcano plots showing differentially expressed genes across tissues, based on a Log_2_FoldChange > 1 and a *p*-value < 0.05. (**c**) The number of differentially expressed genes. Brown bars represent up-regulated genes in *O. sativa* while green bars signify *O. rufipogon* up-regulated genes (**d**) GO enrichment analyses of DEGs in various tissues.

To measure the differential expression of *PPR* genes between the two rice species, we further mapped the RNA-Seq data to the MSU7 genome and identified differentially expressed genes (DEGs). We observed the different expression patterns of *PPR* genes in the panicle tissue in the heatmap of the expression matrix (Figure 6a). Based on the criteria of Fold Change > 2 and P-adj < 0.05, we identified 28 DEGs in the root tissue, 56 DEGs in the leaf tissue, 24 DEGs in the seedling, and 122 DEGs in the panicle (Figure 6b). Notably, within the panicle DEGs, the fertility restoration gene *Rf1* (*LOC_Os10g35436*) with the highest fold change, and another fertility restoration gene, *LOC_Os10g352140*, were also upregulated in *O. rufipogon*. We further categorized the DEGs into genes upregulated in *O. sativa* and upregulated in *O. rufipogon*. We found that, in the root and seedling tissues, *O. sativa* had more upregulated genes, with 17 and 13 genes showing increased expression in the root and seedling, respectively (Figure 6c). We subsequently carried out functional annotation and GO enrichment analysis on genes upregulated in both *O. sativa* and *O. rufipogon* across various tissues. We discovered that genes upregulated in the seedling tissue of *O. sativa* were enriched in the biological process term “embryo development ending in seed dormancy.” However, all these genes were shared genes between *O. sativa* and *O. rufipogon*. In the leaf tissue of *O. rufipogon*, upregulated *PPR* genes were enriched in the “defense response to nematode” biological process term. In the seedling tissue, upregulated *PPR* genes were enriched in the “seed germination” biological process term (Figure 6d).

### 2.5. Segmental Duplication Promotes the Structural Diversity of PPR Genes through Incomplete Gene Duplications

**Figure 7:**
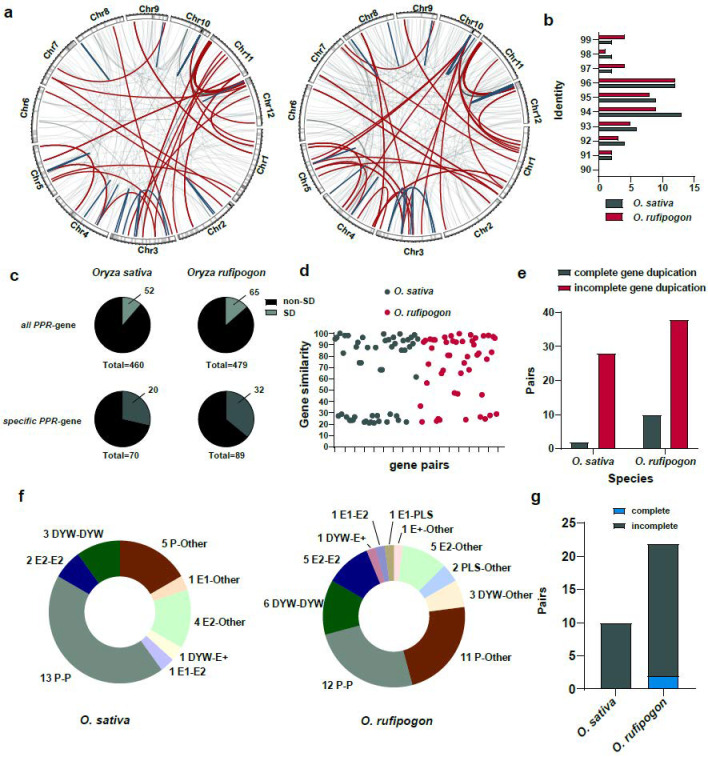
Segmental duplications facilitate incomplete gene duplications and promote the birth of new *PPR* genes. (**a**) *PPR* genes arising from segmental duplications in the *O. sativa* and *O. rufipogon* genomes. Left panel: *O. sativa*; right panel: *O. rufipogon*. Red lines represent inter-chromosomal duplications, dark green lines indicate intra-chromosomal duplications, and light gray lines denote other types of segmental duplications. 1 Mb sliding window with the shading indicates the density of *PPR* genes on each chromosome; (**b**) distribution of identities of segmental duplications. Dark green represents *O. sativa*, while red indicates *O. rufipogon*; (**c**) proportion of *PPR* genes derived from segmental duplications among all *PPR* genes and species-specific *PPR* genes; (**d**) protein similarity between duplicated genes arising from segmental duplications. Each dot represents the similarity between a pair of duplicated genes; (**e**) pairs of complete and incomplete gene duplications through segmental duplications; (**f**) numbers and proportions of duplicated genes across subfamilies with “Other” denoting non-*PPR* paralogous genes; (**g**) number of duplications between *PPR* genes and non-*PPR* genes in *O. sativa* and *O. rufipogon*.

We further identified segmental duplications in the *O. sativa* and *O. rufipogon* genomes using WGAC (Whole Genome Assembly Comparison) and obtained the genome-wide landscape and impact of segmental duplications on *PPR* genes (Figure 7a). Our results suggested that, in the *O. sativa* and *O. rufipogon* genomes, 52 and 65 *PPR* genes, respectively, resulted from segmental duplications. This accounted for the expansion of approximately 11.30% and 13.57% of the *PPR* gene families (Figure 7c; Table S4). Among the lineage-specific *PPR* genes, 20 and 32 genes in the two genomes, respectively, were affected by segmental duplications. We observed that in the *O. sativa* and *O. rufipogon* genomes, identities of segmental duplications mainly ranged from ~94% to 96% (Figure 7b). Additionally, we observed that two pairs and four pairs of segmental duplications with an identity exceeding 99%, representing the youngest segmental duplications, appeared in *O. sativa* and *O. rufipogon*, respectively.

The results showed that the similarity between protein products of homologous genes arising from segmental duplications was relatively lower. Specifically, only 19 and 20 gene pairs with sequence similarities were above 90%, accounting for approximately ~36.54% and ~41.6% of the duplicated gene pairs, respectively. The average similarity among all duplicated gene pairs was ~64.39% for *O. sativa* and ~71.34% for *O. rufipogon* (Figure 7d; Tables S5 and S6). Interestingly, our results suggested that, among the 30 and 48 pairs of segmental duplications in *O. sativa* and *O. rufipogon*, 28 and 38 pairs of segmental duplications, respectively, represented incomplete gene duplications. Only 2 and 10 pairs of segmental duplications were complete gene duplications (Figure 7e; Tables S5 and S6).

We further investigated paralogues of subfamilies of the identified segmental duplications. Our results indicated that 18 and 23 pairs of segmental duplications in *O. sativa* and *O. rufipogon*, respectively, had paralogous copies belonging to the same subfamily. In *O. sativa*, there were 13 pairs of P-P, 3 pairs of DYW-DYW, and 2 pairs of E2-E2, with P-P type gene duplications accounting for ~43.33% of all segmental duplications (Figure 7f). In *O. rufipogon*, there were 12 pairs of P-P, 6 pairs of DYW-DYW, and 5 pairs of E2-E2, with P-P type gene duplications accounting for ~25% of all segmental duplications (Figure 7f). There were two and three gene pairs in *O. sativa* and *O. rufipogon*, respectively, that had paralogous copies belonging to other subfamilies, for example, one pair of E1-E2 duplication and one pair of DYW-E2 duplication in *O. sativa* (Figure 7f). Interestingly, apart from the above-mentioned cases in which both paralogues belonged to the *PPR* gene family itself, a number of gene duplications had a non-*PPR* paralogous gene. In the *O. sativa* and *O. rufipogon* genomes, 10 (~33.33%) and 22 pairs of gene duplications (~45.83%) had non-*PPR* paralogous genes (Figure 7f,g). Specifically, in *O. rufipogon*, there were 11 pairs of P-Other type duplications, 5 pairs of E2-Other type duplications, and 3 pairs of DYW-Other type duplications. Of these duplicated gene pairs with non-*PPR* paralogous genes, *O. sativa* had no complete gene duplications, while *O. rufipogon* only had two complete gene duplications and the others were mainly incomplete gene duplications (~90.9%).

We investigated expression patterns of duplicated genes produced by segmental duplications and further classified them into three categories. When one copy exhibits significantly higher expression levels in two tissues compared to its sister gene and expression in other tissues is not lower than its sister copy, we categorize it as asymmetrically expressed duplicates (AEDs). A gene pair is defined as potentially sub- or neo-functionalized if each copy of the duplicates is expressed at a significantly higher level than the other in at least one tested tissue. The remaining pairs are classified as having no difference. Of 20 pairs in *O. sativa* and 26 pairs in *O. rufipogon*, where both copies belong to the *PPR* gene family, we identified 14 and 16 pairs as AEDs, and 6 and 10 pairs, respectively, as having no significant expression differences across tissues (Figures S7a, S8a and S9). In this study, we failed to find evidence of any sub- or neo-functionalized gene pairs. Within the AEDs, most genes with relatively lower expression than their sister gene maintained at least the “low” level of expression in three or more tissues. The only exception was *LOC_Os11g03850* with a “very-low” level in three tissues, possibly representing a pseudogene or silenced gene, which is a truncated copy of *LOC_Os03g40020* (Figures S7b and S8b).

**Figure 8:**
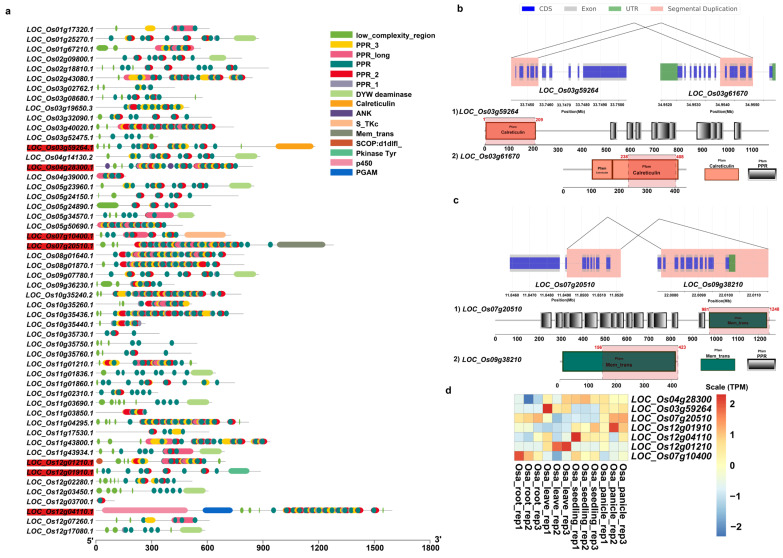
Domain composition of *PPR* genes produced by SDs in *O. sativa*. (**a**) Domain composition of 52 *PPR* genes generated by segmental duplications. Genes marked in red indicate that the protein product contained domains other than *PPR* domain; (**b**) incomplete gene duplications occurred in *PPR* genes that acquired domains from other proteins such as (**b**) *LOC_Os03g59264* and (**c**) *LOC_Os7g20510*. Pink represents homologous regions of segmental duplications. The structures of paralogous genes are depicted; coding sequences are represented in blue, exon sequences are represented in gray, and UTR regions are represented in green. The annotation of domain composition of the homologous genes is shown below; the gene structure with the homologous regions is marked in red and the location of homologous region is also labeled; (**d**) expression profiling of 7 *PPR* genes with other domains.

**Figure 9:**
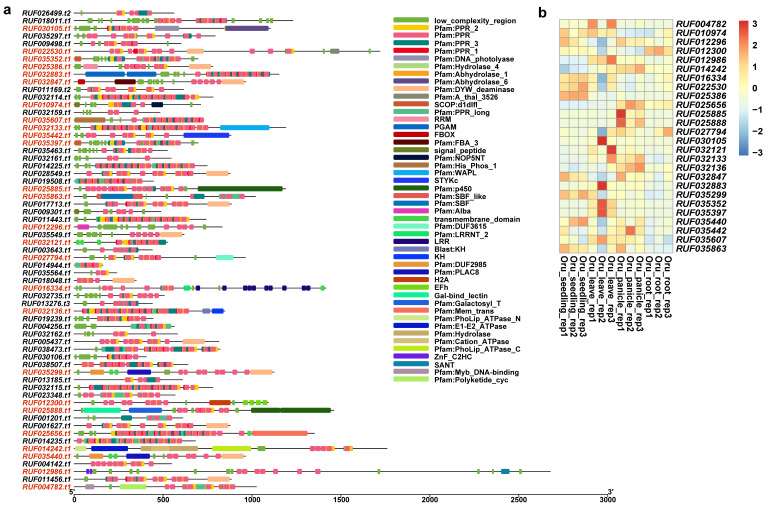
Domain composition of *PPR* genes produced by SDs in *O. rufipogon*. (**a**) Domain composition of 65 *PPR* genes duplicated by segmental duplications. Genes marked in red indicate that the protein product of the gene contains domains other than *PPR* domain; (**b**) expression of 26 *PPR* genes with other domains.

We further predicted domain structures of the protein products of these duplicated genes with non-*PPR* paralogous genes using SMART. Among these incomplete genes produced by segmental duplications, our results showed that, in addition to *PPR* motifs, *O. sativa* had a total of 7 genes with other domains, while *O. rufipogon* had 26 genes (Figure 8a and Figure 9a). Previously reported examples of *PPR* genes with other domains included PPR-SMRs, which had eight members in the *Arabidopsis* genome that contained both *PPR* motifs and SMR domains [58]. In addition, domains such as proteinaceous RNase P (PRORP) and LAGLIDADG motifs were also present in different subclasses of *PPR* genes [3]. We examined the structural origins of these *PPR* genes with other domains, which were mainly generated by incomplete duplications through segmental duplications. Our results showed that these homologous sequences were not *PPR* motifs but rather other domains. For instance, *LOC_Os03g59264* had a Calreticulin domain, *LOC_Os07g10400* contained a S_TKc domain, and *LOC_Os07g20500* featured a Mem_trans domain. We then performed comparative analyses with other *Oryza* genomes to determine the direction of gene duplications. Our results intriguingly suggested that members of the *PPR* gene family often act as the “accepter” of other genes’ domains (Figure 8b,c). We further investigated the expression of these genes, and our results showed that five of them had moderate levels of expression in at least one tissue while *LOC Os12g01910* and *LOC Os12g04110* had low levels of expression in three and four tissues (Figure 8d). Of a greater number of duplicated genes with other domains in *O. rufipogon* than *O. sativa*, we interestingly found that some members even had multiple different domains, such as *RUF025888*, which had the Gal-bind_lectin, Galactosyl_T, and p450 domains (Figure 9a). In *O. rufipogon*, 23 of them had moderate-level expression in at least one tissue while *RUF010974, RUF03544,* and *RUF035442* had low levels of expression in all four tissues (Figure 9b).

## 3. Discussion

PPR proteins are a protein family that widely exists in plants and some protists, and they regulate gene expression and RNA metabolism by binding to specific RNA sequences [1,25,59]. Recent decades have witnessed that the *PPR* gene families have been identified in multiple plant species, such as *O. sativa* [34,60], *A. thaliana* [7], *Populus trichocarpa* [5], *Citrullus lanatus* [61], and *Camellia sinensis* [62]. In this study, we performed whole-genome analysis and identified 460 and 479 *PPR* genes in *O. sativa* and *O. rufipogon*, respectively, which were then classified into six subgroups. Our findings are consistent with previous characterization of this gene family in *O. sativa* [34,60].

Based on the analysis of conserved domain alignment, *PPR* genes can be classified into two subfamilies, P and PLS, which were further confirmed by phylogenetic analysis. However, a few members within each subfamily were admixed. This phenomenon was also observed in previous studies [5,6]. The amino acid arrangement of PPR protein domains in these two rice species is highly consistent with other plant species [5], indicating that PPR proteins are highly conserved across flowering plants.

Previous studies have shown that in most species of *PPR* genes in angiosperms, over half of the individuals have only one exon [60,63]. The proportions of genes with only one exon were the highest with percentages of 45.65% and 38.00% in *O. sativa* and *O. rufipogon*, respectively, which agree with the observation that up to 80% of the *PPR* genes in rice were intron-less [34]. The results support that retrotransposition has resulted in the expansion of the *PPR* gene family in rice and its wild progenitor.

We investigated the origins and evolution of *PPR* gene families since the domestication of *O. sativa* from its wild progenitor. There were ~36.17% and ~42.94% of species-specific *PPR* genes from new gene duplications in the *O. sativa* and *O. rufipogon* genomes, respectively. The results thus provide evidence that the evolution of the gene family is ongoing as a result of newly occurring gene duplications, although the *PPR* gene family is relatively conserved across land plants.

In spite of the recent split of rice and *O. rufipogon*, GO enrichment analysis of *PPR* genes uniquely identified in *O. sativa* and up-regulated genes in seedling tissue of *O. sativa* exhibited an enrichment of a unique biological process related to the embryo development ending in seed dormancy. We found a unique SMR domain in the included gene, *LOC_Os06g09880.1*. Genes with this domain were previously reported to be directly involved in RNA editing of chloroplast gene *rps8* transcripts [64]. PPR-SMR1 is a PPR protein containing the SMR domain, and Zm-mCSF1 is a protein containing the CRM domain, both of which target mitochondria. The loss-of-function mutations of these two genes severely hindered embryogenesis and endosperm development in maize [65]. Although we still cannot determine whether the upregulation of these *PPR* genes is associated with artificial selection during the domestication process, this finding may highlight the potential role of *PPR* genes in regulating seed embryo development and seed dormancy.

It is worthwhile noting that these *PPR* genes are agronomically very important functional genes, such as *RFL* (Restoration Fertility Like) genes for rice breeding programs (Table S7). Furthermore, our results showed that the genes upregulated in the leaf tissues of *O. rufipogon* were enriched entries related to biological processes for nematode defense. These loci may have potential breeding values for insect-resistant traits.

Although segmental duplications have only expanded approximately 11% of the *PPR* gene family members, we interestingly obtained evidence that segmental duplication promotes the structural diversity of *PPR* genes through incomplete gene duplications. Our results indicate that the similarity between protein products of homologous genes arising from segmental duplications was relatively lower. This could be explained by incomplete gene duplications, and only partial sequences are homologous between duplicated gene pairs. We interestingly observed that, among duplicated gene pairs with non-*PPR* paralogous genes, *O. sativa* had two complete gene duplications, while *O. rufipogon* only had ten complete gene duplications and the others were mainly incomplete gene duplications. Our results showed that most of the duplicated genes that both belong to the *PPR* gene resulting from segmental duplications exhibited asymmetrical expression. Except for two potentially silenced genes, the rest of the AEDs maintained low expression levels. These duplicated genes might be limited by dosage effects, as most of the recently duplicated genes tend to be functionally redundant, making them susceptible to loss-of-function mutations that could lead to the degradation of one copy into a pseudogene [66]. However, among the duplicated genes in which *PPR* genes contained other domains, which were mainly generated by incomplete duplication through segmental duplications, 5 out of 7 in *O. sativa* and 23 out of 26 in *O. rufipogon* exhibited expression levels above moderate in at least one tissue. These foreign domains might promote functional diversification, allowing them to escape the constraints of dosage effects.

As previously reported, *PPR-SMR*s are present in species representing major angiosperms, and recent gene duplication may account for the additional copies of some *PPR-SMR*s in *A. thaliana* [58]. In this study, we illustrate examples of *PPR* genes contained in other domains in the two closely related rice species, with incomplete gene duplications explaining the origins of these domains. While we are uncertain if these *PPR* genes with newly acquired domains follow the same mechanism in other plants, based on our observation here, we speculate that, during plant evolution, segmental duplications leading to incomplete gene duplications might result in the acquisition of domains, thus promoting the functional innovation and diversification of *PPR* genes. Accordingly, this study offers a unique perspective on the evolution of *PPR* gene structures and underscores the potential role of segmental duplications in *PPR* gene structural diversity.

## 4. Materials and Methods

### 4.1. Identification of PPR Genes

The rice genome of MSU7 was downloaded from http://rice.uga.edu/, accessed on 10 September 2023. The *O. rufipogon* genome data file can be found in the National Genomics Data Center under the accession number PRJCA002346 [67]. To comprehensively detect the *PPR* gene family in these two genomes, we downloaded the seed file (PF01535) of the *PPR* gene family based on the Hidden Markov Model (HMM) from the Pfam V31.0 database (http://pfam.xfam.org/, accessed on 9 September 2023). To identify *PPR* genes using the HMMER 3.0 program [68], we set the E-value < 10 as a cutoff to filter out fewer significant matches to increase the confidence in identifying true *PPR* genes. We used the SMART program (http://smart.embl-heidelberg.de/, accessed on 11 September 2023) to analyze the protein domains of the candidate *PPR* genes obtained from the two genomes. We finally used the HMMER 3.0 [68] matrix defined by conserved domains of *PPR* gene subfamilies (P, PLS, E1, E2, E+, and DYW) in *A. thaliana* to search, analyze, and classify these protein sequence domains. Proteins that do not have any *PPR* motifs or have only one *PPR* motif are typically discarded or excluded from further analyses.

### 4.2. Chromosomal Location and Gene Structure and Cis-Acting Element Analysis

We employed Weblogo (http://weblogo.berkeley.edu/logo.cgi, accessed on 11 September 2023) to create a motif recognition to assess the conservation of *PPR* motifs. The position of *PPR* genes in the genome was visualized in Circos V0.69 [69]. To analyze the cis-acting elements in the major promoter regions of the *PPR* genes, a sequence of 2000 base pairs upstream of the transcription start site was selected. An online tool named PlantCARE (http://bioinformatics.psb.ugent.be/webtools/plantcare/html/, accessed on 12 September 2023) was used to obtain all the cis-acting elements in each gene’s promoter region, and important responsive elements were selected through screening. The plots were created using the heatmap Package in R V4.2.3 [70].

### 4.3. Phylogenetic Analysis and Gene Ontology (GO) Enrichment Analysis

The *PPR* genes from the two rice species were aligned using the MAFFT V7.310 method [71]. Alignments were trimmed using TrimAL V1.4 [72] with a minimum conservation threshold and a gap threshold of 20%. The phylogenetic tree was built using the maximum likelihood method in IQ-TREE V2.2.3 [73], with the best evolutionary model “LG+F+R10”. We constructed the phylogenetic tree using Maximum likelihood with 1000-replicate bootstrap analysis. iTOL is used to beautify and annotate evolutionary trees and add subgroup information [74]. We performed functional annotation of the PPR gene family in *O. sativa* and *O. rufipogon* using the InterProScan 5.54-87.0 program [75]. The results were visualized using Tbtools V2.008 [76].

### 4.4. Analysis of PPR Gene Expression and Classification of Expression Patterns of Sister Duplicates

Field-grown rice (*O. sativa* ‘Nipponbare’; *O. rufipogon* ‘R1’) tissues including 7-d-old seedlings, mature plant flag leaves, roots, and panicles were harvested and frozen in liquid nitrogen for subsequent RNA sequencing and proteomic experiments. The RNeasy Mini Kit (Qiagen) bench protocol was used for plant total RNA extraction. To investigate patterns of *PPR* gene expression, we sequenced transcriptome data from four different tissues of *O. sativa* and *O. rufipogon*. The raw sequencing data were submitted to the Genome Sequence Archive (Genomics, Proteomics & Bioinformatics 2017) in the BIG Data Center, Beijing Institute of Genomics (BIG), Chinese Academy of Sciences, under accession number PRJCA020852. RNA-sequencing data were mapped using HISAT2 V2.2.1 [77] on the reference genome after building the index for *O. sativa* and *O. rufipogon*. The read counts matrix for 24 samples (4 tissues, each tissue with three biological replicates) was generated using featureCounts V2.0.1 [78]. Differentially expressed genes (DEGs) were evaluated by DESeq2 V1.42.0 [79]. The threshold for DEGs was Fold Change > 2 and p-adj < 0.05. For the duplicated genes generated by segmental duplications, we selected gene pairs in which both copies belong to the *PPR* gene family for downstream analysis. Differential gene expression analyses among the duplicated gene pairs for each tissue were performed using DESeq2 V1.42.0 [79] with the same threshold. We divided the duplicated gene pairs into three classes according to their expression in four tissues: (1) sub- or neo-functionalized pairs, in which each of the two duplicates was significantly more highly expressed than the other in at least one tissue; (2) AEDs, when one copy exhibits significantly higher expression levels in two tissues compared to its sister gene and expression in other tissues is not lower than its sister copy; and (3) the remaining duplicates were classified as no-difference pairs [66]. A heatmap and volcano plot were plotted using pheatmap V1.0.12 and EnhancedVolcano V1.20.0 [70].

### 4.5. Identification of Shared and Species-Specific PPR Genes in O. sativa and O. rufipogon and Ks Analysis

To identify orthologous *PPR* genes in *O. sativa* and *O. rufipogon*, we first performed a reciprocal blast using Blastp 2.14 [80] on the genes retrieved from the two rice genomes. Subsequently, we selected the reciprocal best hits as candidates for orthologous genes. Next, we assessed whether the neighboring genes also had reciprocal best hits with the neighboring orthologous genes in the other genome. The criteria for the local synteny filter was based on the orthologous relationship of genes surrounding the *PPR* genes. Specifically, if there is at least one gene on each side within five upstream and downstream genes surrounding a *PPR* gene that has an orthologous relationship with counterpart genes in the other species, then the pair of genes passes the local synteny filter. If both conditions were met, these genes were categorized as shared genes; otherwise, they were considered species-specific genes. We further validated the results by using Orthofinder V2.5.2 [81] and MCScan V1.1.12 [82], which validated 387 gene pairs (Figures S10 and S11). For the three gene pairs that could not be identified as synteny genes by MCScan V1.1.12 [82], we performed sequence-level synteny analysis using Nucmer 4.0.0rc1 [83], which was certified (Figure S12). We conducted *Ks* analysis for orthologous gene pairs (orthogroups) and gene duplication groups (paralogous groups). We defined gene duplication groups as follows: firstly, we identified all duplicated gene pairs in both *O. sativa* and *O. rufipogon* using dupgen_finder. We then extracted putative duplicated pairs of lineage-specific *PPR* genes. Subsequently, we used paraAT2.0 [84] to generate aligned pairwise sequences in axt format through Muscle [85] alignment. We then calculated *Ks* values for each pair of genes, both orthologous and all duplicated gene pairs, using the YN model in KaKs_Calculator V2.0 [86]. For the following data processing, we took the minimum *Ks* value from all duplicated gene pairs as an approximation of the most recent gene duplication event. We then compared this value to the average *Ks* value of all orthologous *PPR* genes.

### 4.6. Identification of Gene Duplications and Segmental Duplications

We characterized different types of gene duplications using DupGen_finder [87] (https://github.com/qiao-xin/DupGen_finder, accessed on 14 September 2023) and counted the number and proportion of each type of *PPR* gene duplication in the whole genome. We identified segmental duplications in *O. sativa* and *O. rufipogon* with the Whole-Genome Assembly Comparison (WGAC) pipeline [49]. Firstly, the genome assemblies were split into trackable 400-kb segments after the TEs were removed leaving the unique genome for further searching all sequence similarity using BLAST 2.14 [80] and self-blast using webb_self program in SUN workstation. The BLAST results were parsed for alignments if they had >88% identity and >200 bp aligned length. After reinserting common repeats into resulting pairwise alignments and trimming the ends, the final set of segmental duplications was identified if global alignment bases were longer than 1 kb matched and had >90% identity.

### 4.7. Identification of Domain and Visualization of Segmental Duplication

We used SMART [88] (http://smart.embl-heidelberg.de/smart/, accessed on 11 September 2023) to identify the domains of the *PPR* gene and employed Tbtools V2.008 [76] to visualize the domains. KaryoploteR [89] and Circos V0.69 [69] were used to depict segmental duplication in the genomes. We employed our own script to plot the structures of duplicated genes.

## Data Availability

Data are contained within the article or Supplementary Material.

## Supplementary Materials

The following supporting information can be downloaded at: https://www.mdpi.com/article/10.3390/ijms242216313/s1.
