# Supplementary material for: Comparative Genomic Analysis of Asian Cultivated Rice and Its Wild Progenitor (Oryza rufipogon) Has Revealed Evolutionary Innovation of the Pentatricopeptide Repeat Gene Family through Gene Duplication
Source: Int J Mol Sci. 2023 Nov 14;24(22):16313. doi: 10.3390/ijms242216313 (PMC10671101; doi:10.3390/ijms242216313)
Supplement: Supplementary file 1 [file ijms-24-16313-s001.zip › Captions.pdf]

Figure S1: Classification and statistical analysis of exons. (a) Classification of genes with one exon in the two subfamilies among all *PPR* genes in the two species.; (b) Exon statistics for shared and species-specific genes in the two species;

Figure S2: Types and quantity of cis-regulatory elements in the *PPR* gene promoter of *O. sativa*;

Figure S3: Types and quantity of cis-regulatory elements in the *PPR* gene promoter of *O. rufipogon*;

Figure S4: Identification of shared *PPR* genes between *O. sativa* and *O. rufipogon*. Red links represent reciprocal best hit and synteny *PPR* genes between two genomes. Blue links indicate only reciprocal best hit genes;

Figure S5: Transcriptome correlation heatmap and cluster analysis;

Figure S6: Expression levels of *PPR* genes in four tissues across the two rice species;

Figure S7: Expression of *PPR* genes generated from segmental duplications in *O. sativa* (both copies belong to the *PPR* family). (a) Heatmap plotted by pheatmap and scaled by column; (b) bar chart of TPM expression levels for the two copies;

Figure S8: Expression of *PPR* genes generated from segmental duplications in *O. rufipogon* (both copies belong to the *PPR* family). (a) Heatmap plotted by pheatmap and scaled by column; (b) bar chart of TPM expression levels for the two copies;

Figure S9: Number of each category of duplicated gene pairs with different expression patterns in *O. sativa* and *O. rufipogon*;

Figure S10: Synteny visualization of 387 shared genes validated by MCScan, Each shared *PPR* gene pair is labeled in the figure;

Figure S11: Number of anchor genes within 5 (a) and 20 (b) up-/down-stream orthologous *PPR* genes;

Figure S12: The sequence synteny visualization of 2 Mb flanking regions of the three gene pairs that could not be identified as synteny genes by using MCScan. (a) *LOC_Os01g37870-RUF002107*, (b) *LOC_Os11g29230-RUF033983,* and (c) *LOC_Os11g29360-RUF033991*. Left panel: Dotplot of 2 Mb flanking regions. Right panel: Synteny view with the position of the gene, which is marked in red.

Table S1: The number of *PPR* genes in the *O. sativa* genome;

Table S2: The number of *PPR* genes in the *O. rufipogon* genome;

Table S3: The number of various types of gene duplication in the *PPR* gene family;

Table S4: The number of *PPR* genes generated by segmental duplications;

Table S5: The number of *PPR* genes generated by segmental duplications in the *O. rufipogon* genome;

Table S6: Characteristics of segmental duplications in the *O. sativa* genome;

Table S7: List of the identified Quantitative Trait Loci (QTLs) in the *PPR* gene family.
