# Supplementary material for: Comparative Genomic Analysis of Asian Cultivated Rice and Its Wild Progenitor (Oryza rufipogon) Has Revealed Evolutionary Innovation of the Pentatricopeptide Repeat Gene Family through Gene Duplication
Source: Int J Mol Sci. 2023 Nov 14;24(22):16313. doi: 10.3390/ijms242216313 (PMC10671101; doi:10.3390/ijms242216313)

LOC_Os01g01115 RUF000036

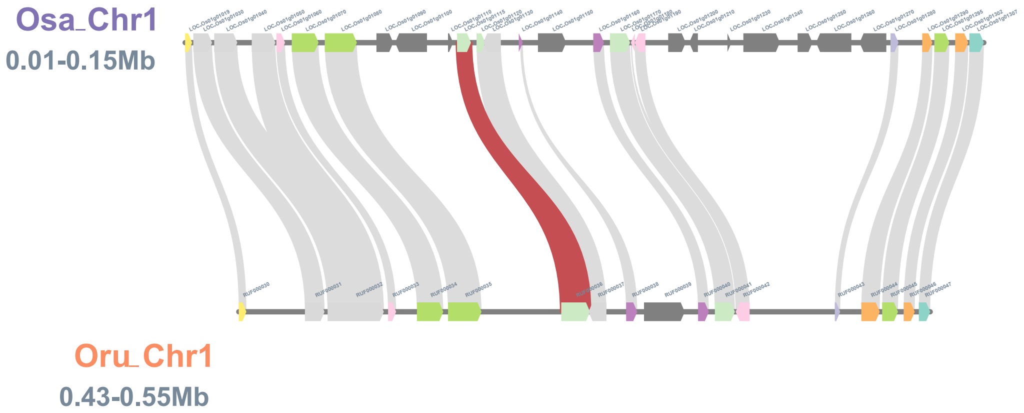

LOC_Os01g05860 RUF000374

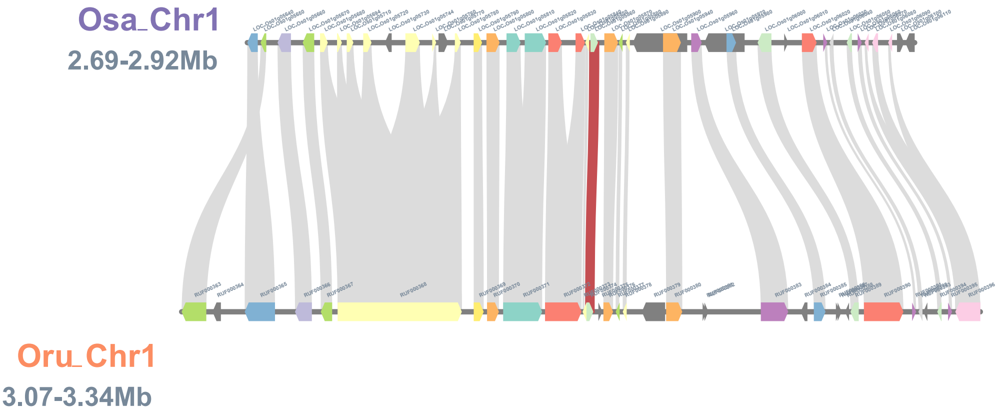

LOC_Os01g06000 RUF000389

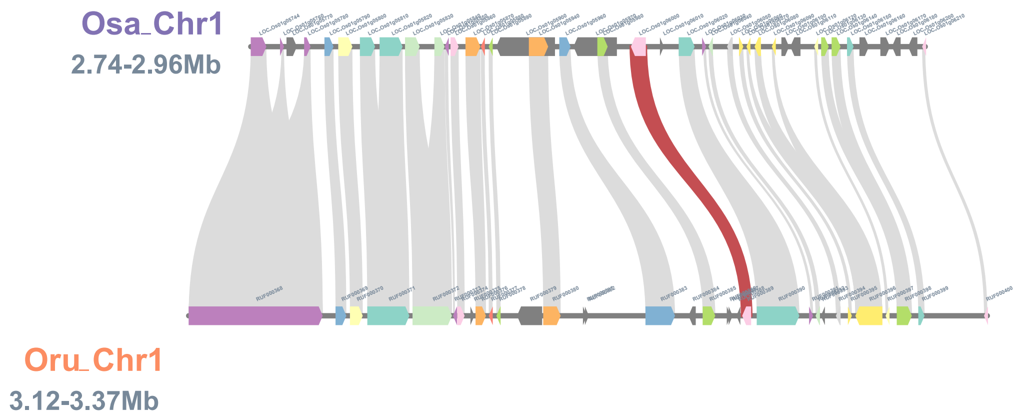

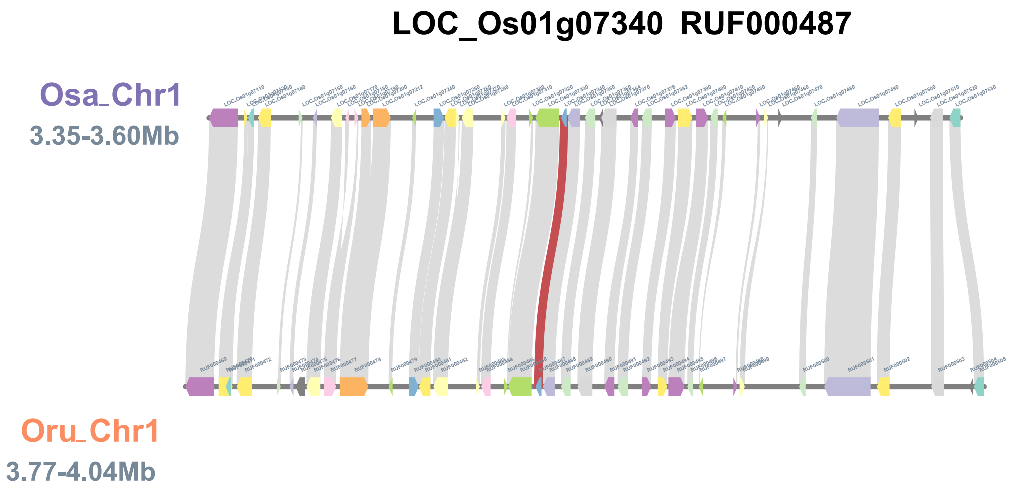
LOC_Os01g07340 RUF000487
Osa_Chr1
3.35-3.60Mb
Oru_Chr1
3.77-4.04Mb

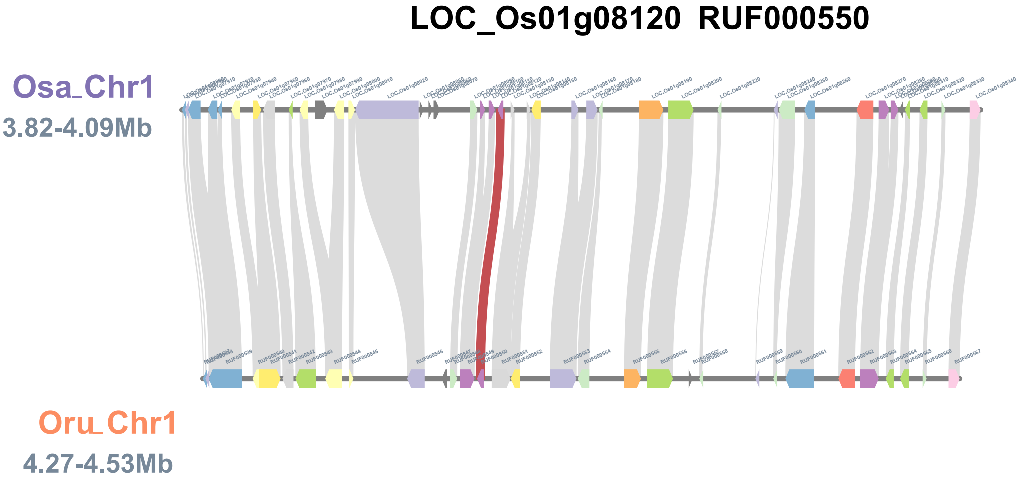
LOC_Os01g08120 RUF000550
Osa_Chr1
3.82-4.09Mb
Oru_Chr1
4.27-4.53Mb

LOC_Os01g10090 RUF000699

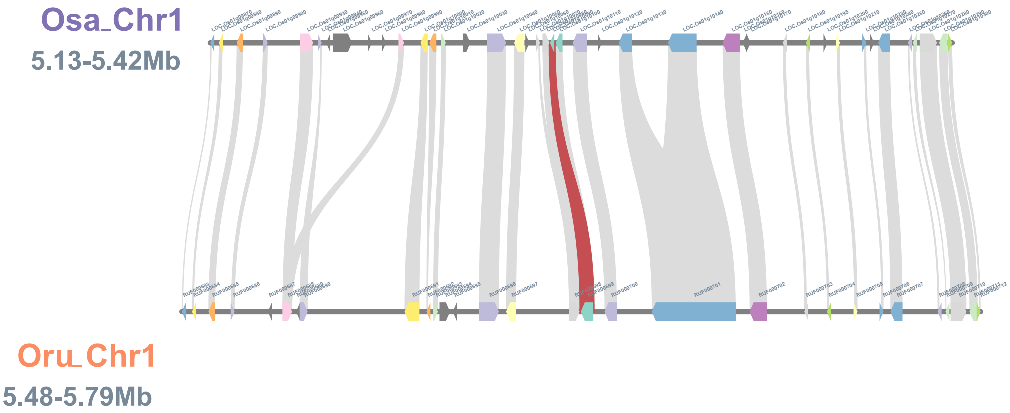

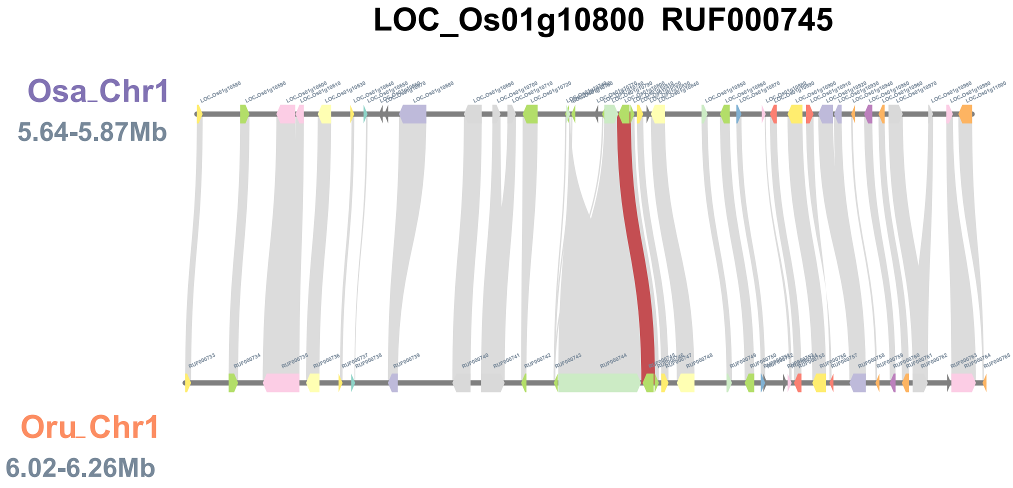
LOC_Os01g10800 RUF000745
Osa_Chr1
5.64-5.87Mb
Oru_Chr1
6.02-6.26Mb

LOC_Os01g12810 RUF000907

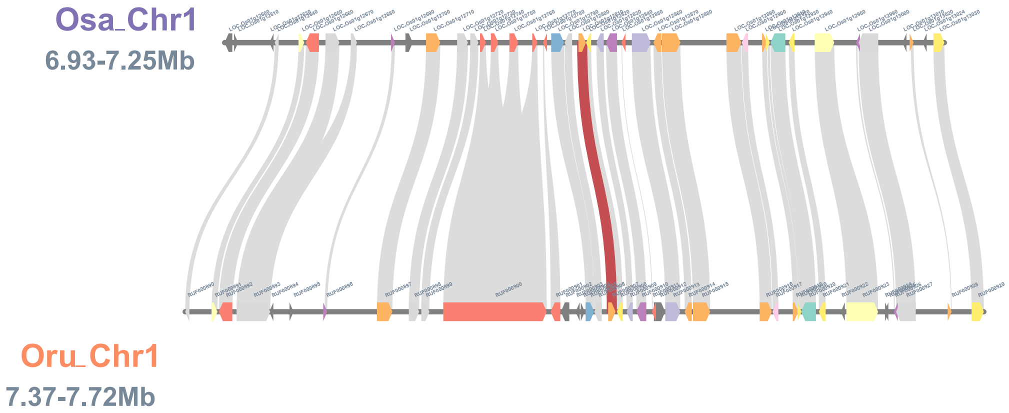

LOC_Os01g15530 RUF001055

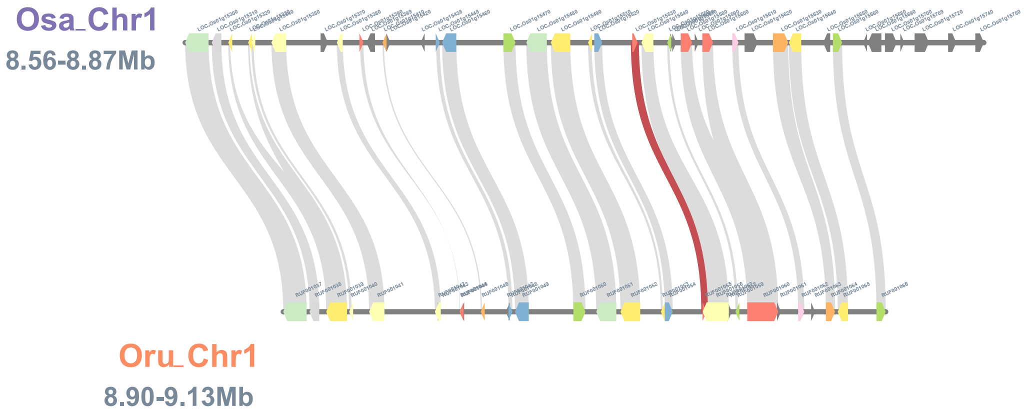

LOC_Os01g15840 RUF001075

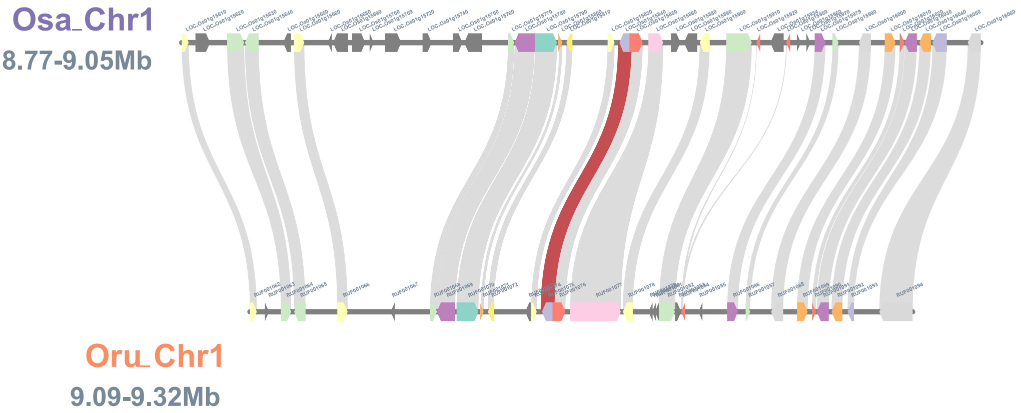

LOC_Os01g17320 RUF001201

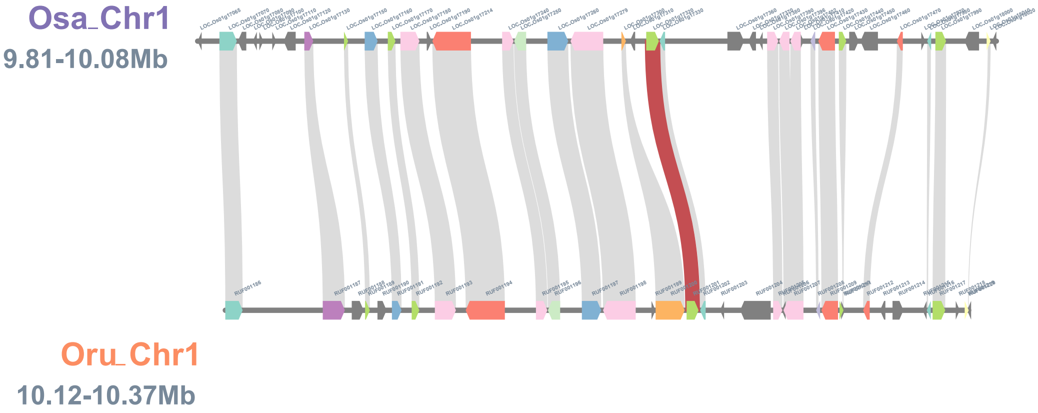

LOC_Os01g19380 RUF001315

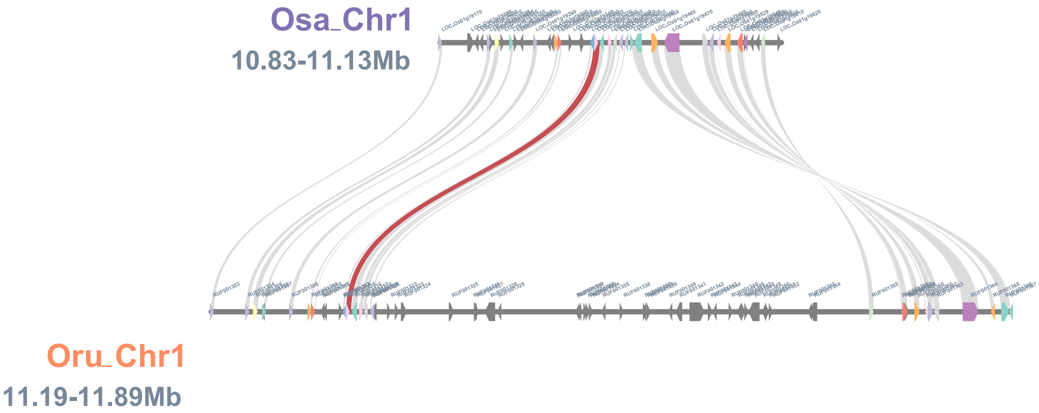

LOC_Os01g19490 RUF001362

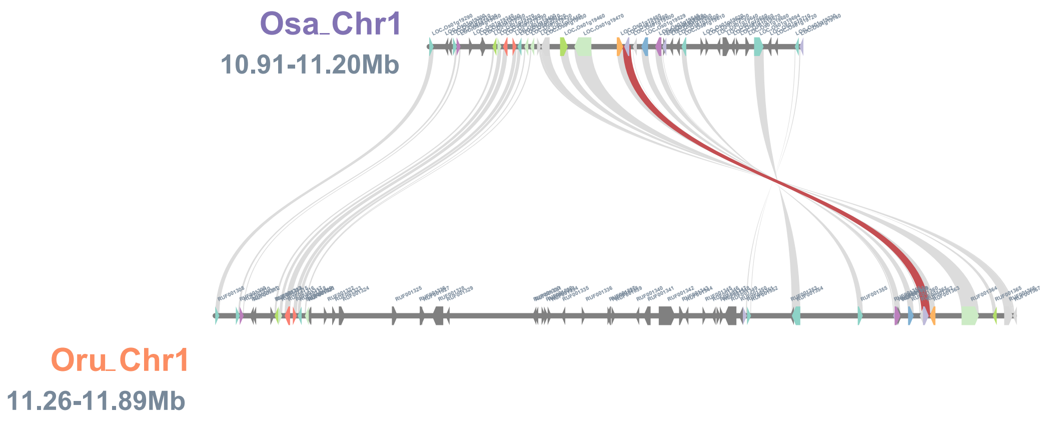

LOC_Os01g19529 RUF001360

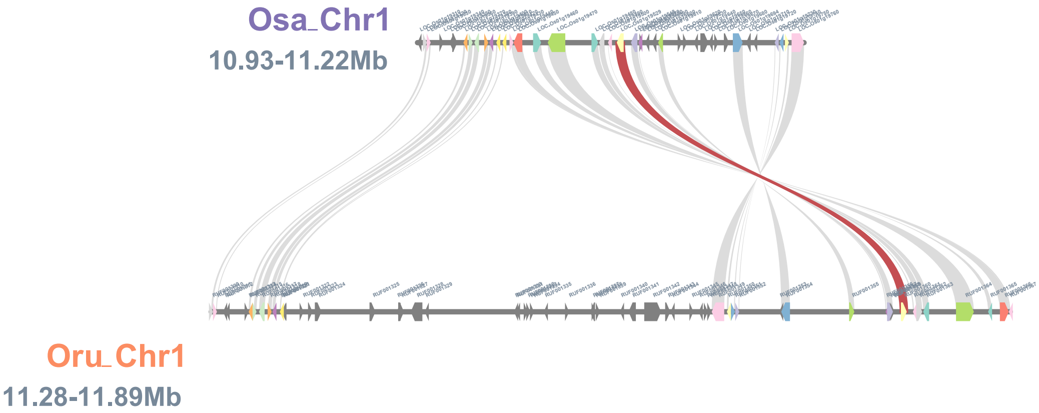

LOC_Os01g19548 RUF001357

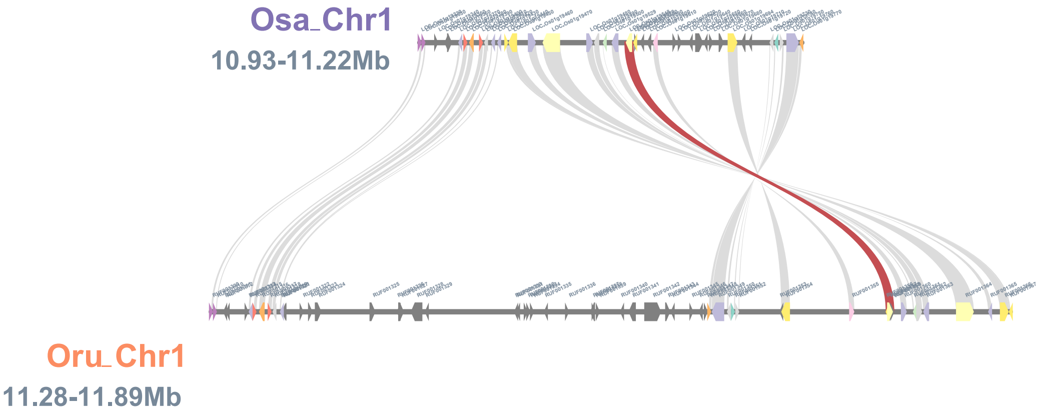

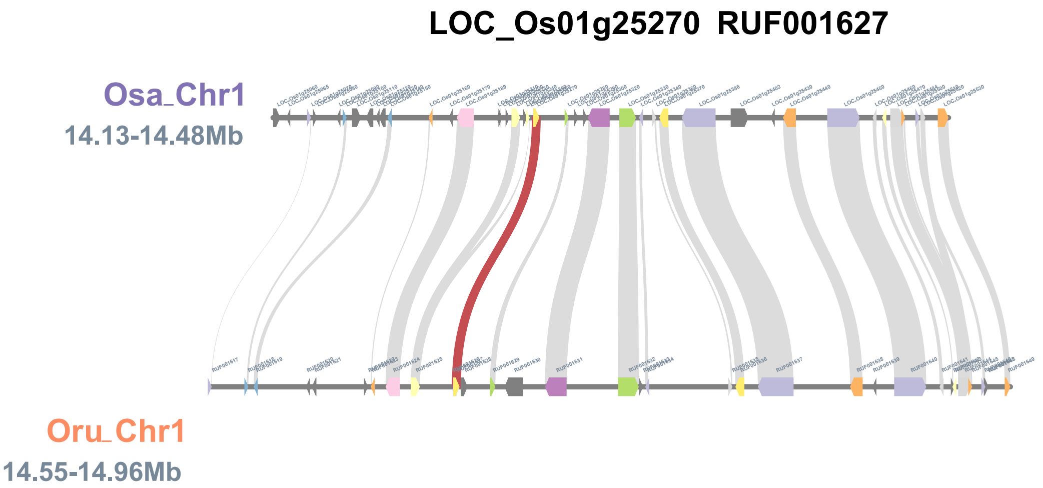
LOC_Os01g25270 RUF001627
Osa_Chr1
14.13-14.48Mb
Oru_Chr1
14.55-14.96Mb

LOC_Os01g25530 RUF001649

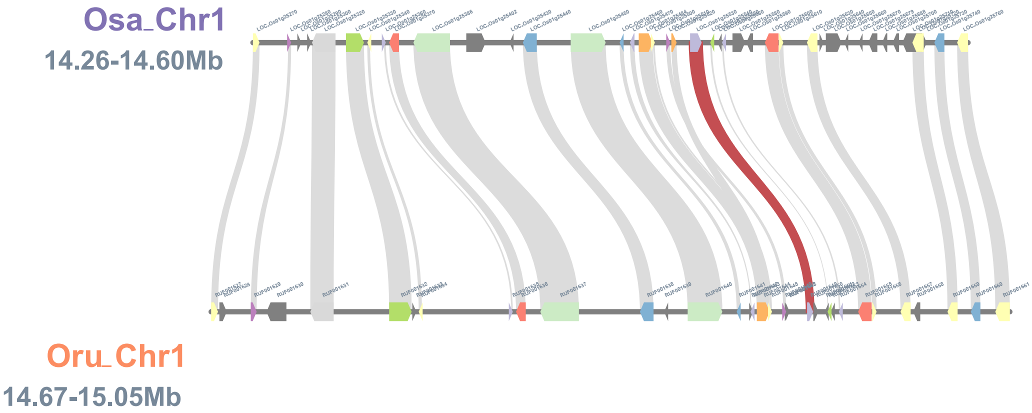

LOC_Os01g27650 RUF001730

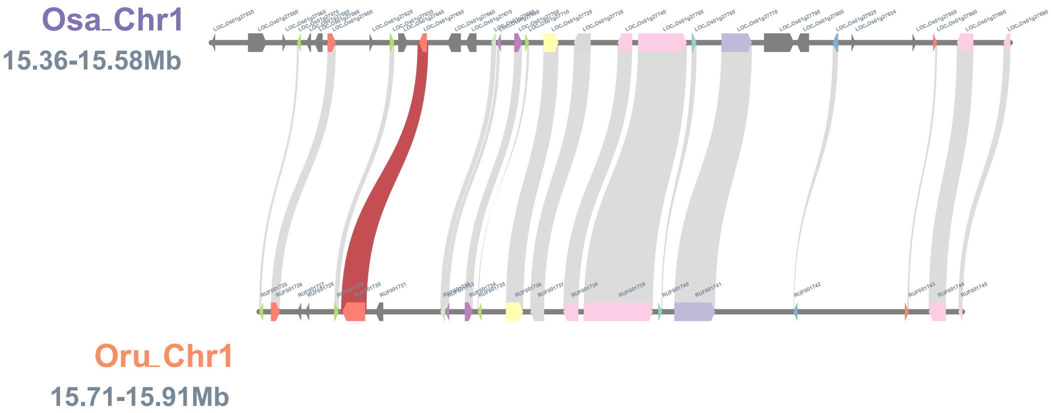

LOC_Os01g29430 RUF001799

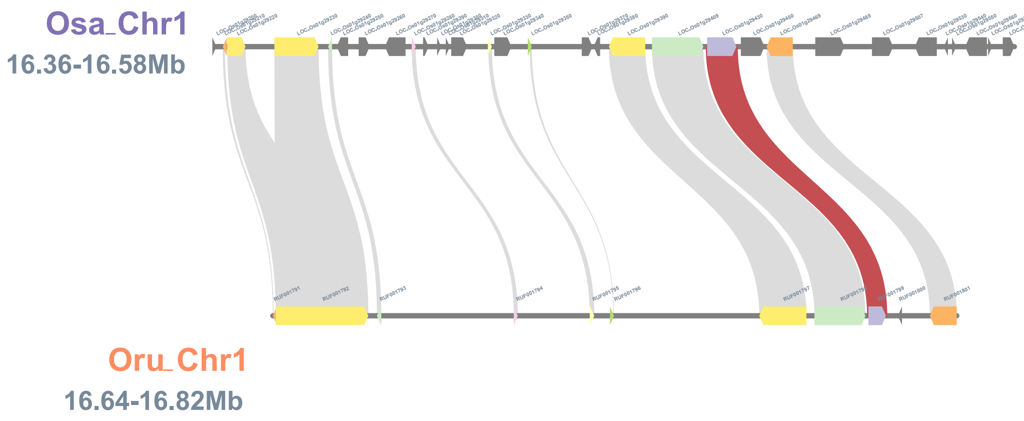

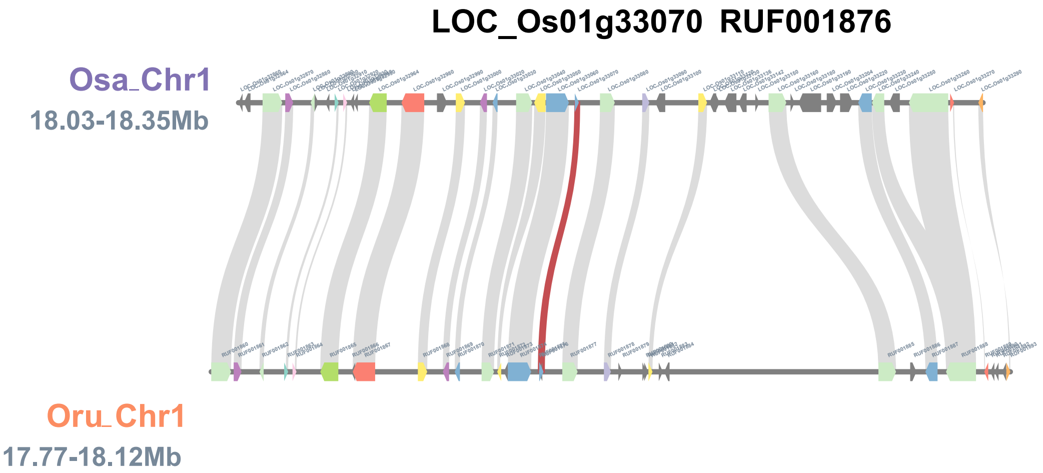
LOC_Os01g33070 RUF001876
Osa_Chr1
18.03-18.35Mb
Oru_Chr1
17.77-18.12Mb

LOC_Os01g36540 RUF002036

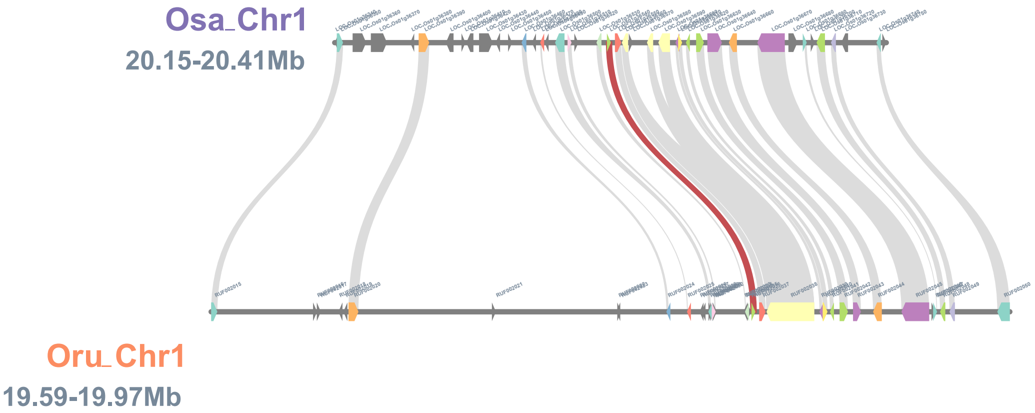

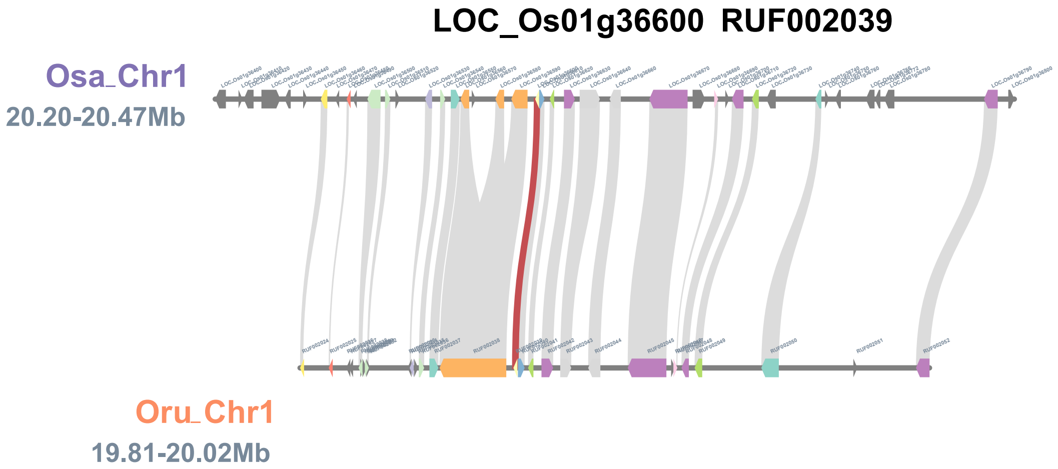
LOC_Os01g36600 RUF002039
Osa_Chr1
20.20-20.47Mb
Oru_Chr1
19.81-20.02Mb

LOC_Os01g36610 RUF002040

Osa_Chr1

20.21-20.48Mb

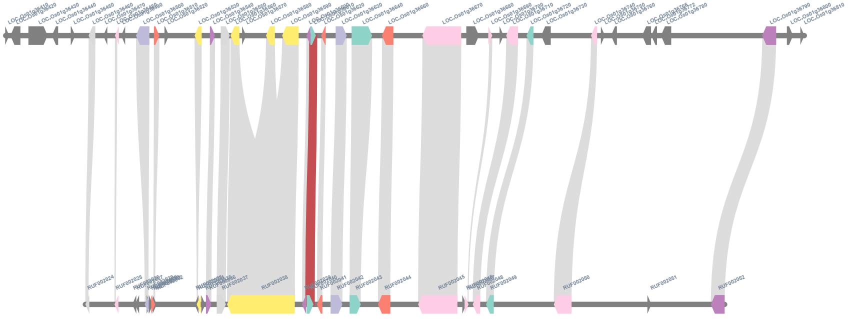

Oru_Chr1

19.81-20.02Mb

LOC_Os01g40680 RUF002304

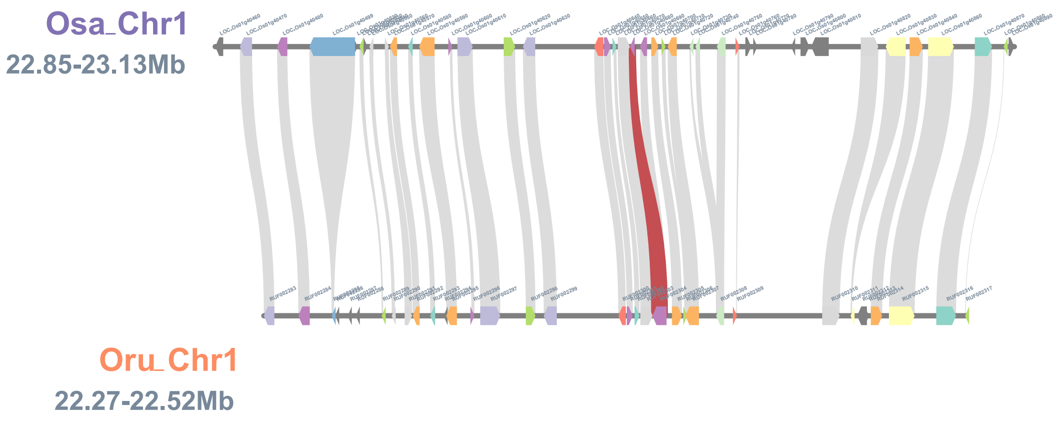

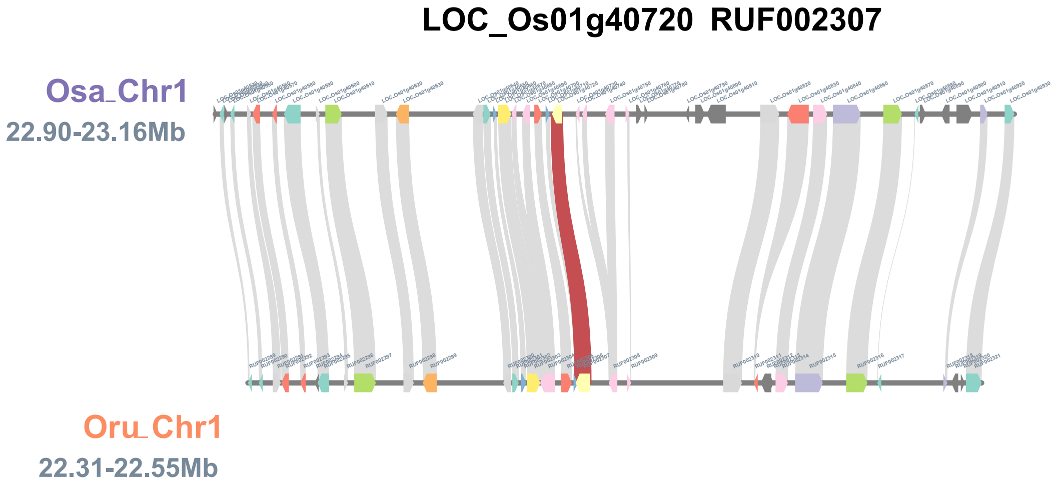
LOC_Os01g40720 RUF002307
Osa_Chr1
22.90-23.16Mb
Oru_Chr1
22.31-22.55Mb

LOC_Os01g41650 RUF002373

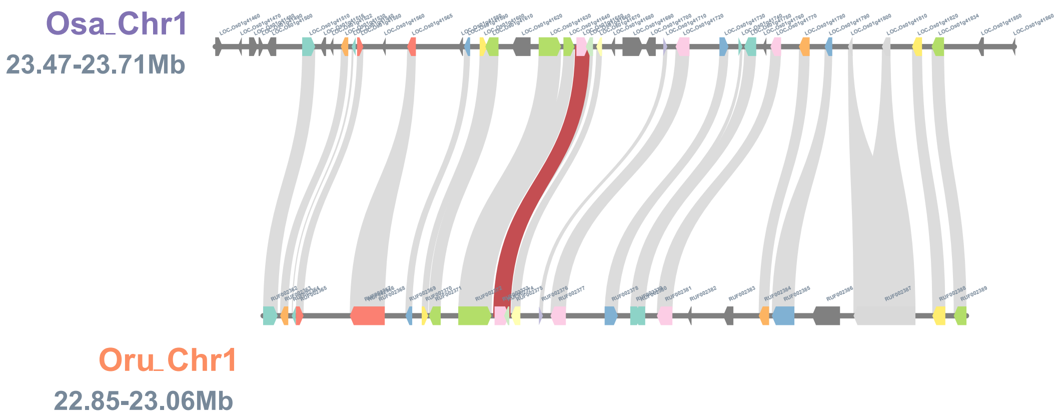

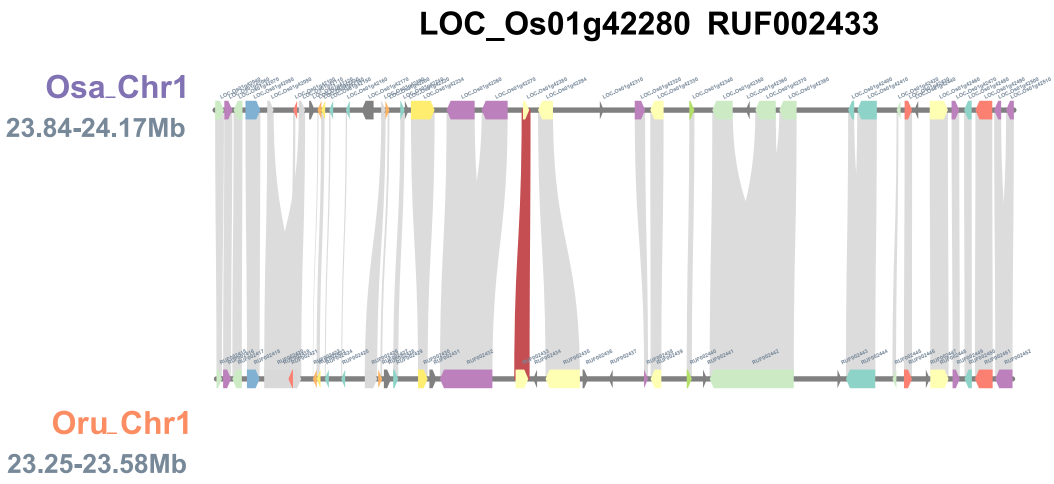
LOC_Os01g42280 RUF002433
Osa_Chr1
23.84-24.17Mb
Oru_Chr1
23.25-23.58Mb

LOC_Os01g42620 RUF002459

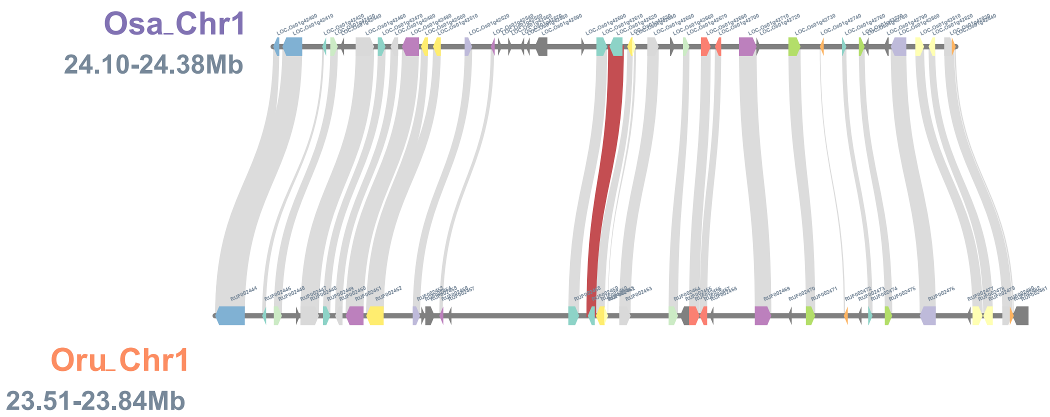

LOC_Os01g46230 RUF002698

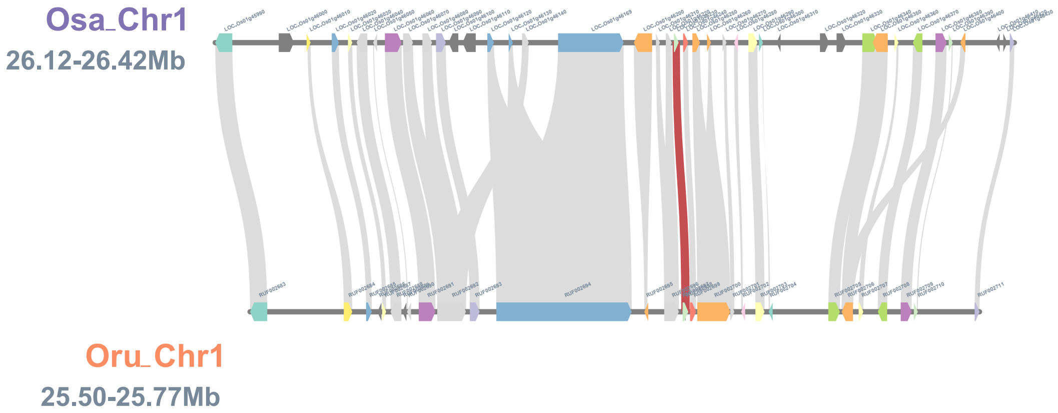

LOC_Os01g48140 RUF002832

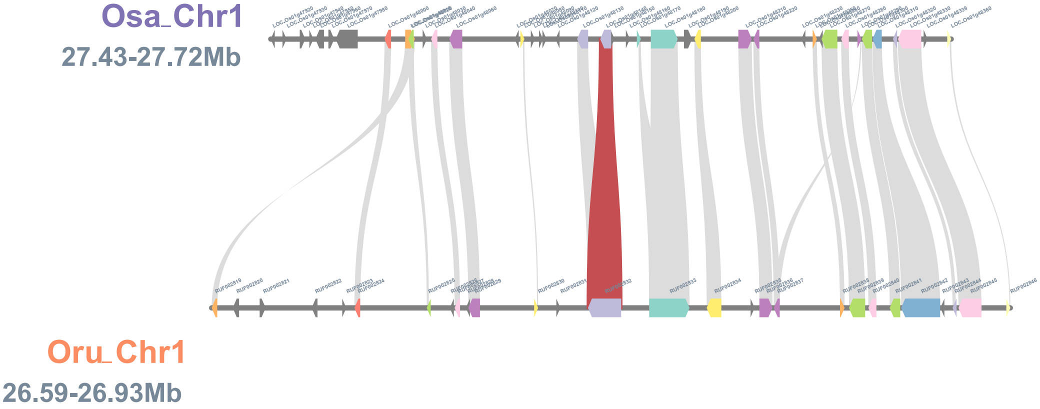

LOC_Os01g48380 RUF002848

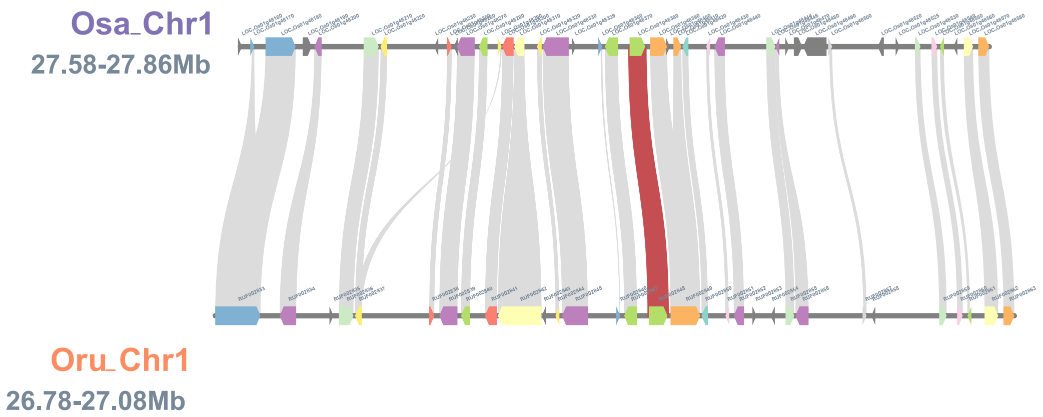

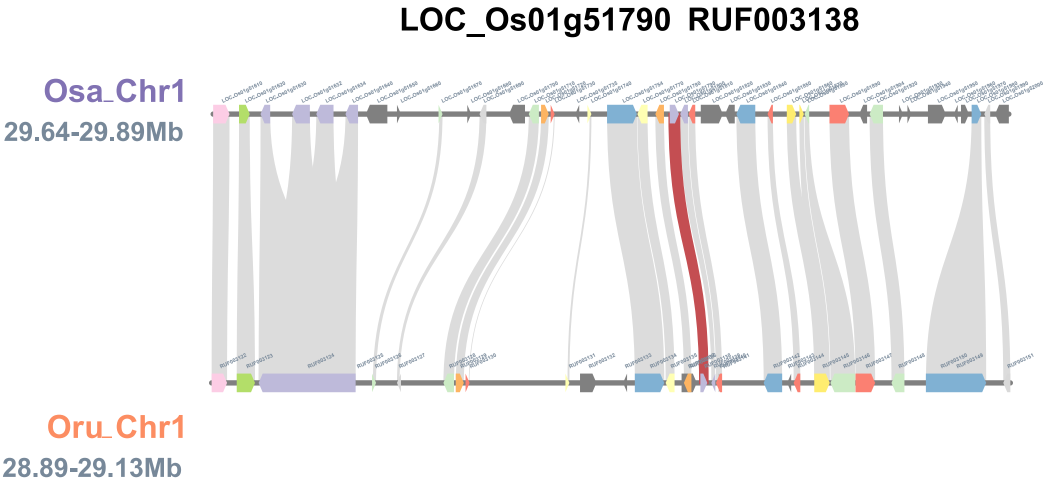
LOC_Os01g51790 RUF003138
Osa_Chr1
29.64-29.89Mb
Oru_Chr1
28.89-29.13Mb

LOC_Os01g51810 RUF003140

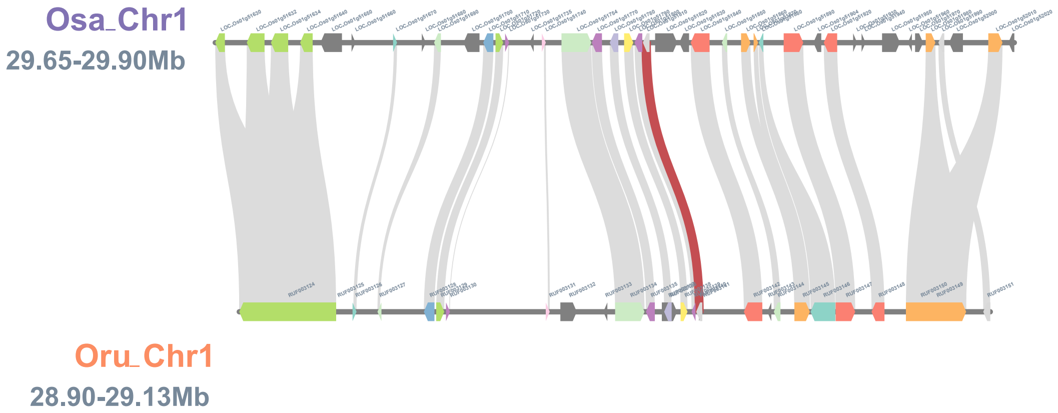

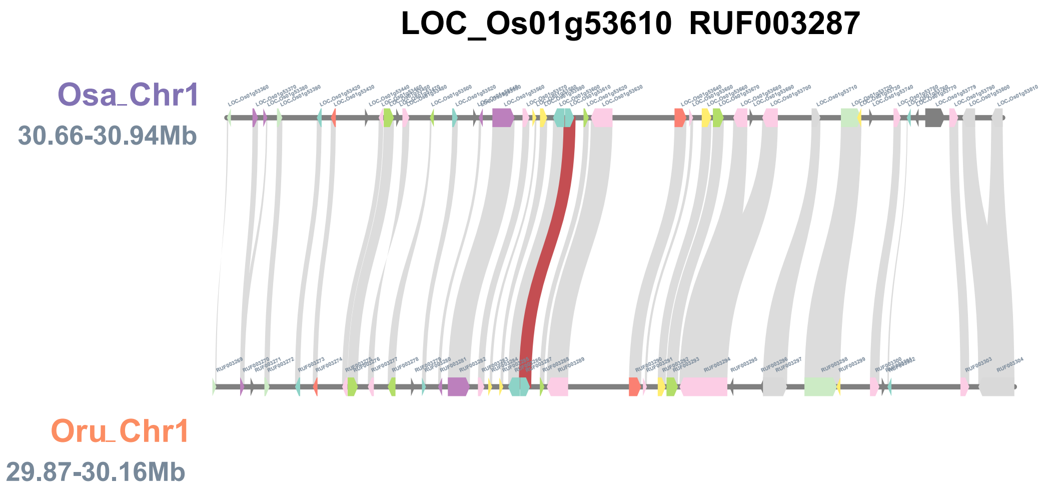
LOC_Os01g53610 RUF003287
Osa_Chr1
30.66-30.94Mb
Oru_Chr1
29.87-30.16Mb

LOC_Os01g55070 RUF003408

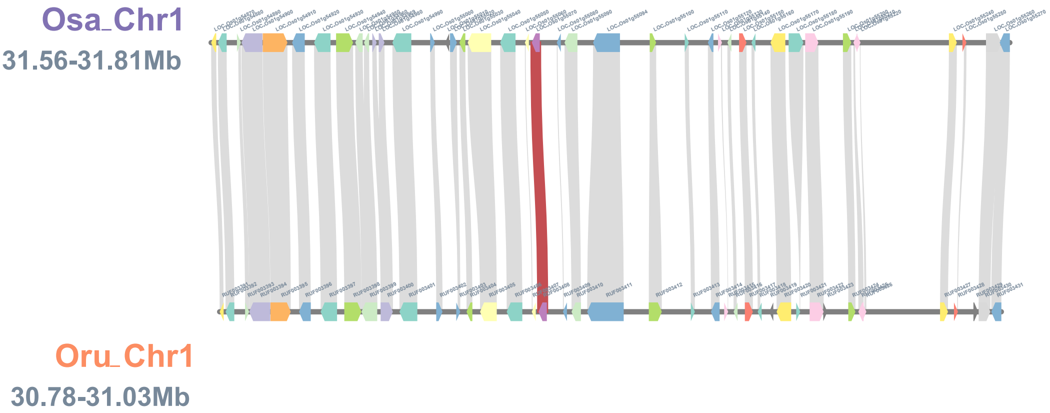

LOC_Os01g55960 RUF003492

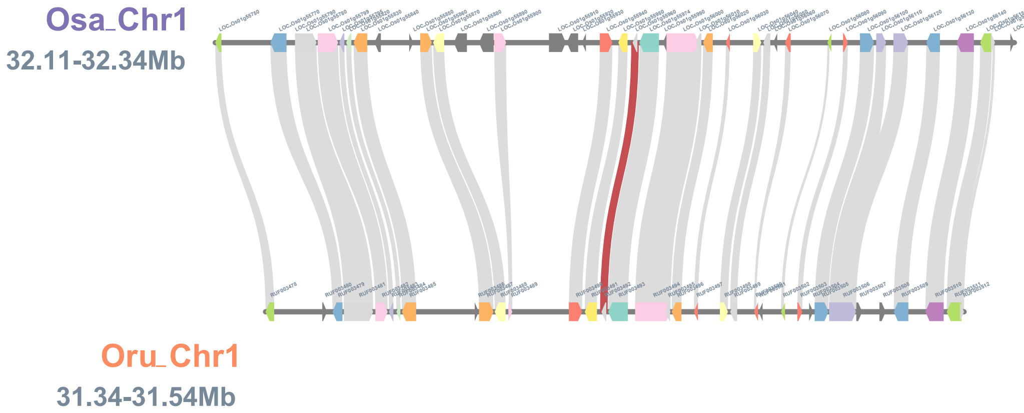

LOC_Os01g57410 RUF003604

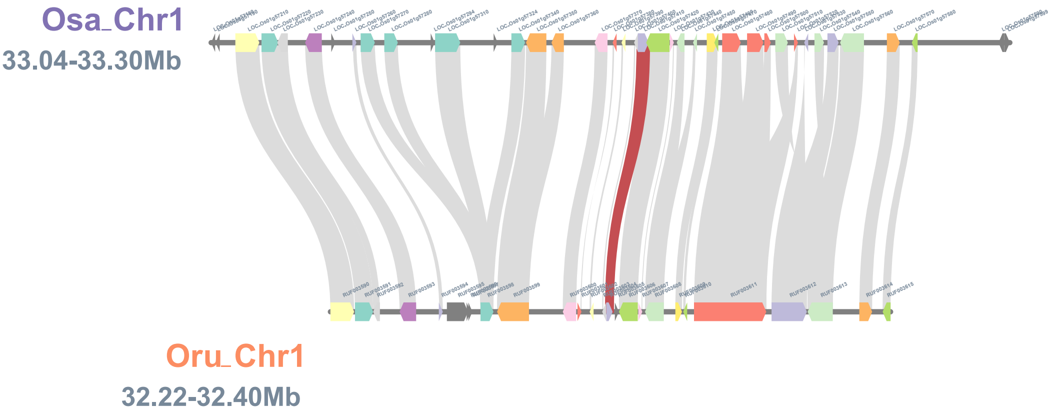

LOC_Os01g58630 RUF003643

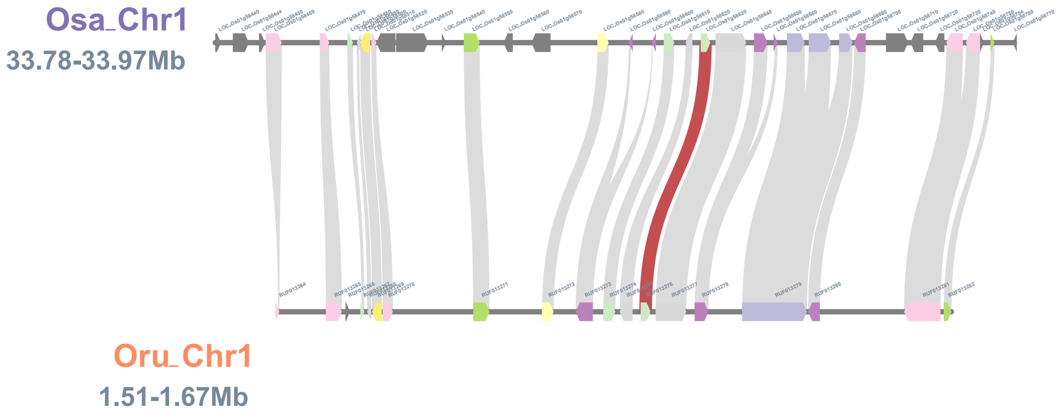

LOC_Os01g59890 RUF003683

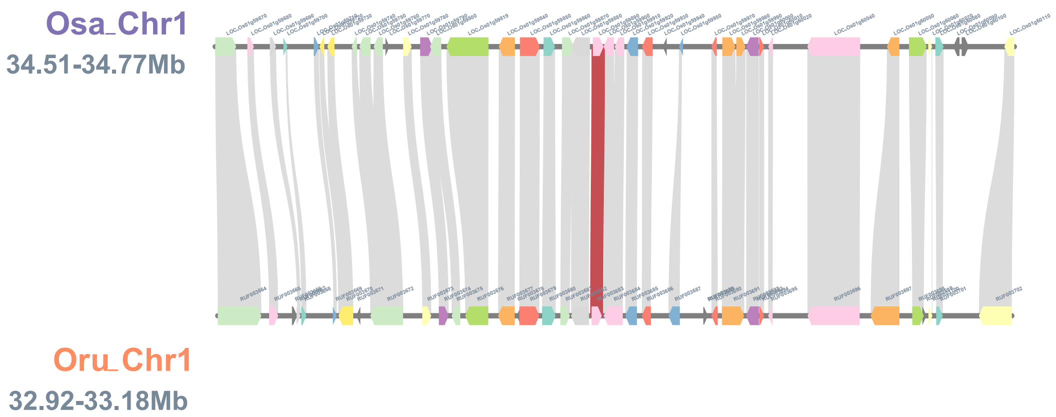

LOC_Os01g60000 RUF003692

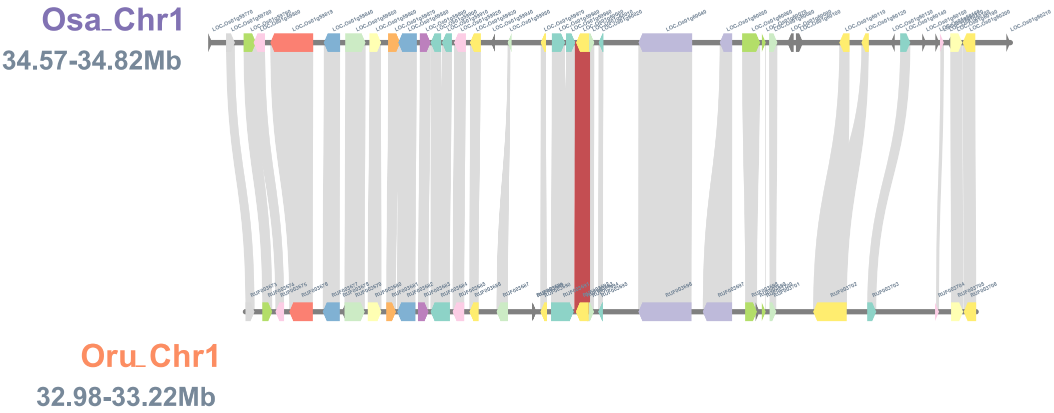

LOC_Os01g60010 RUF003694

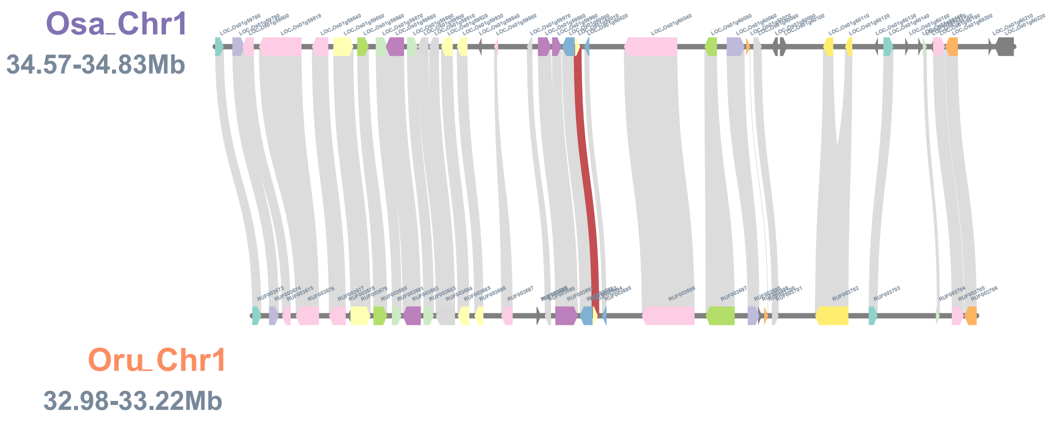

LOC_Os01g60250 RUF003710

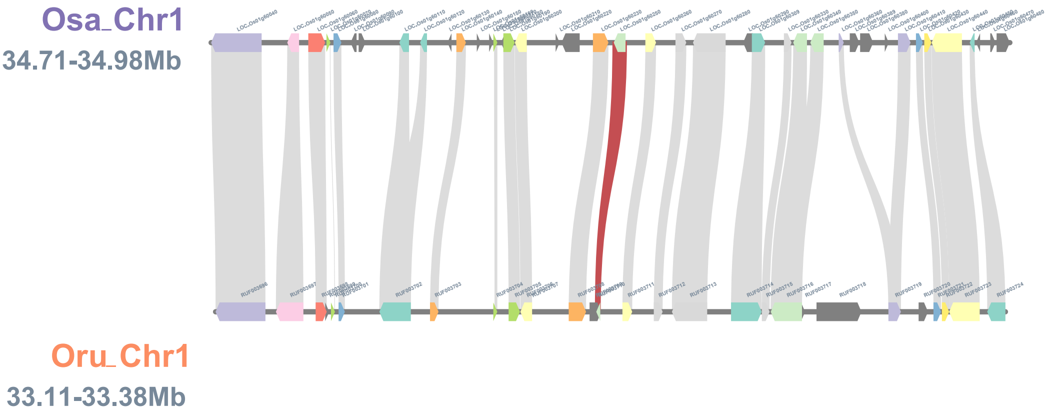

LOC_Os01g60430 RUF003722

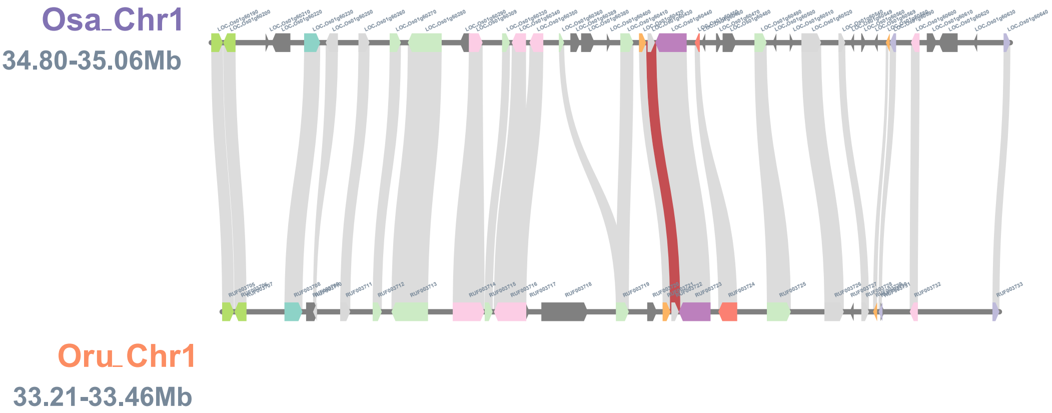

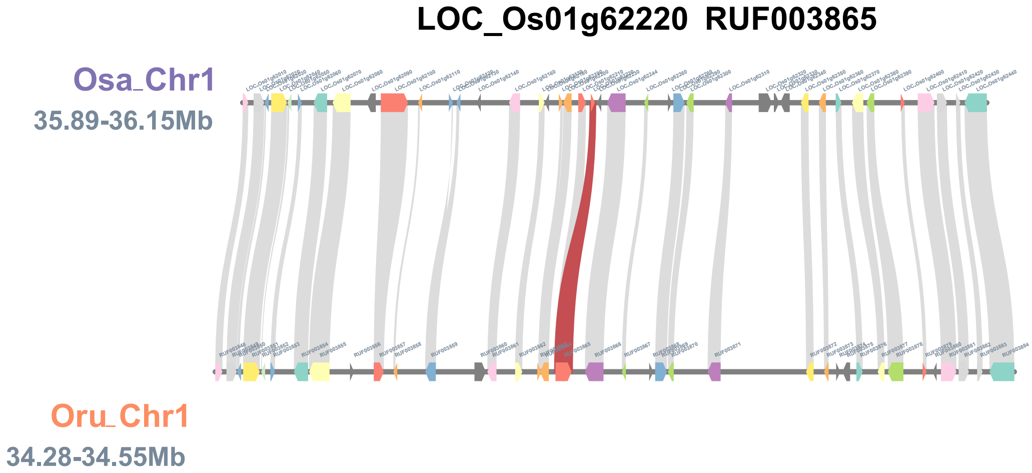
LOC_Os01g62220 RUF003865
Osa_Chr1
35.89-36.15Mb
Oru_Chr1
34.28-34.55Mb

LOC_Os01g62910 RUF003922

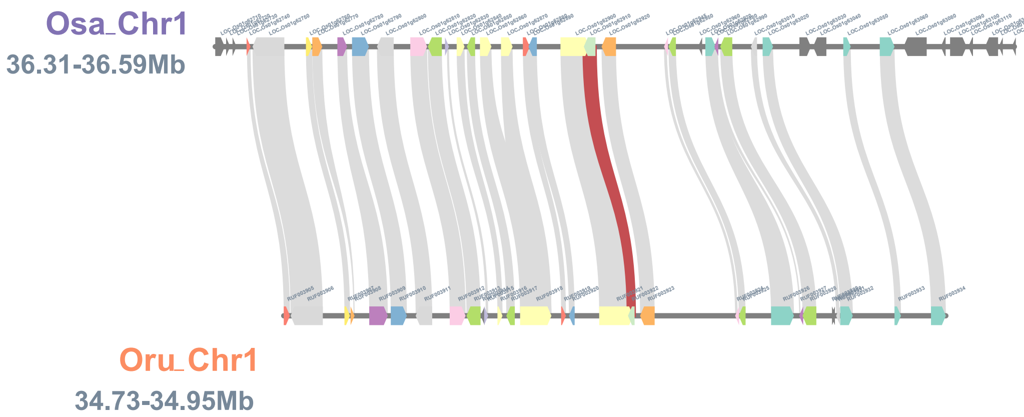

LOC_Os01g63400 RUF003951

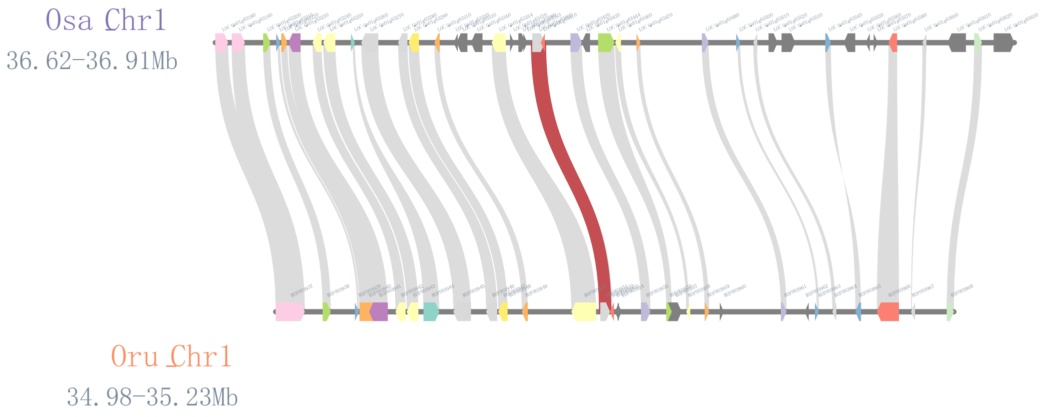

LOC_Os01g65840 RUF004142

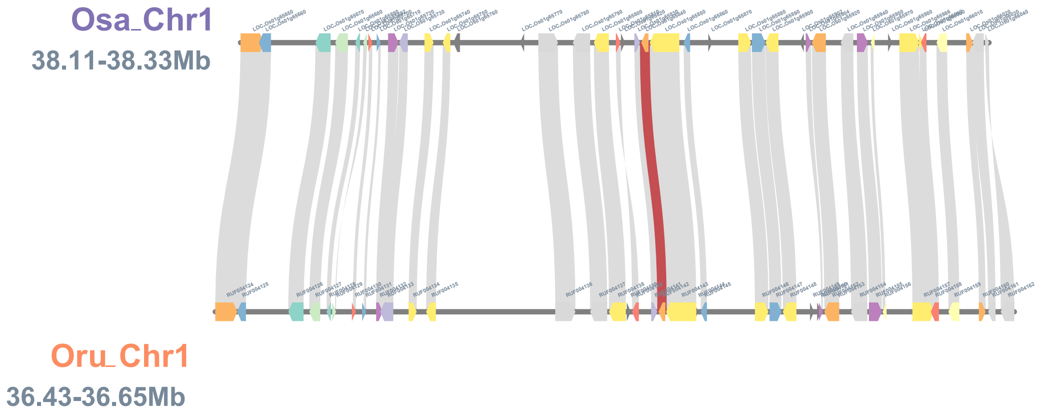

LOC_Os01g66160 RUF004172

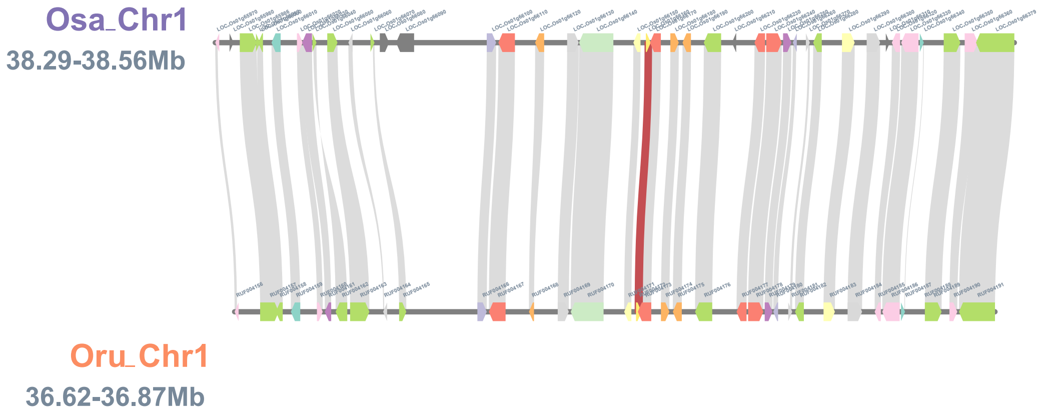

LOC_Os01g67210 RUF004256

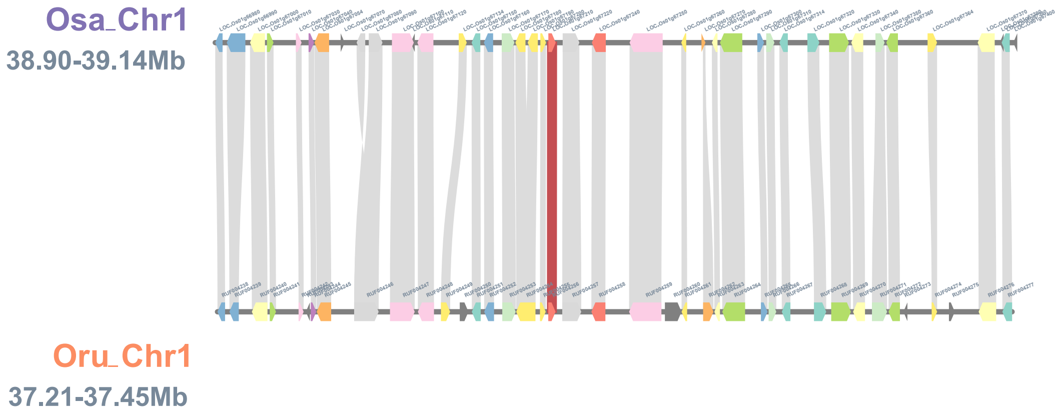

LOC_Os01g68070 RUF004332

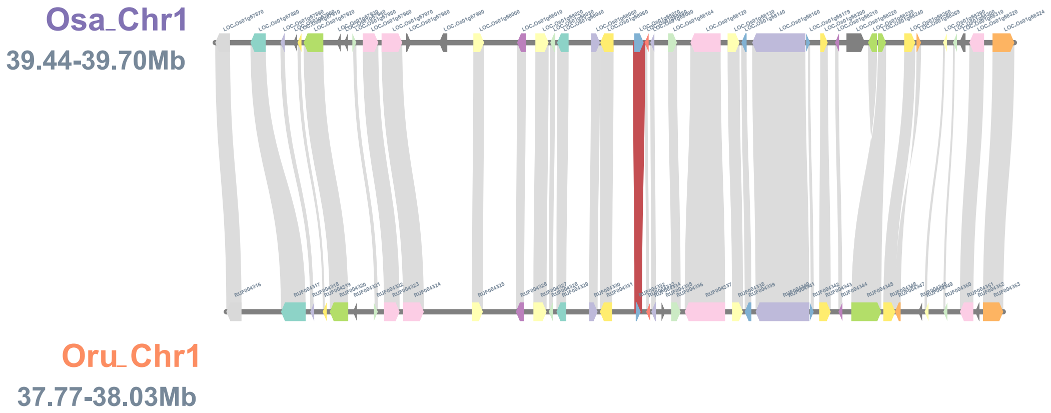

LOC_Os01g68490 RUF004365

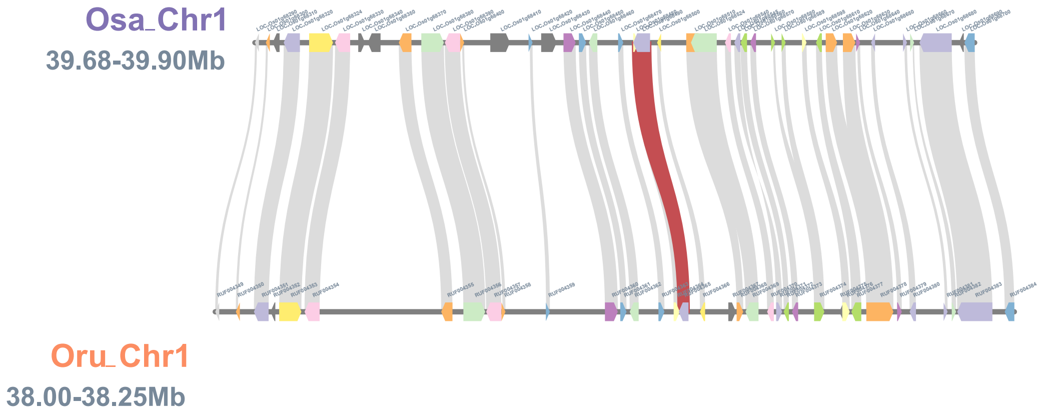

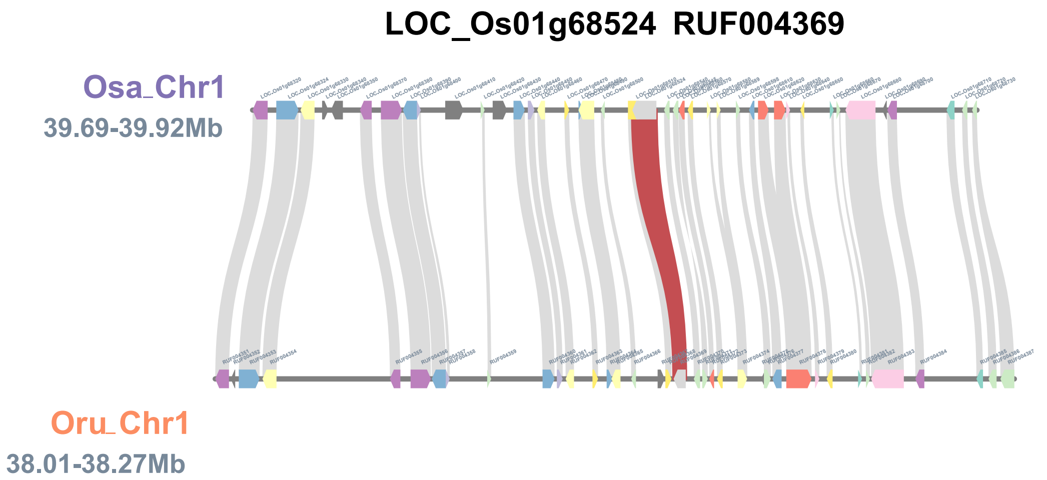
LOC_Os01g68524 RUF004369
Osa_Chr1
39.69-39.92Mb
Oru_Chr1
38.01-38.27Mb

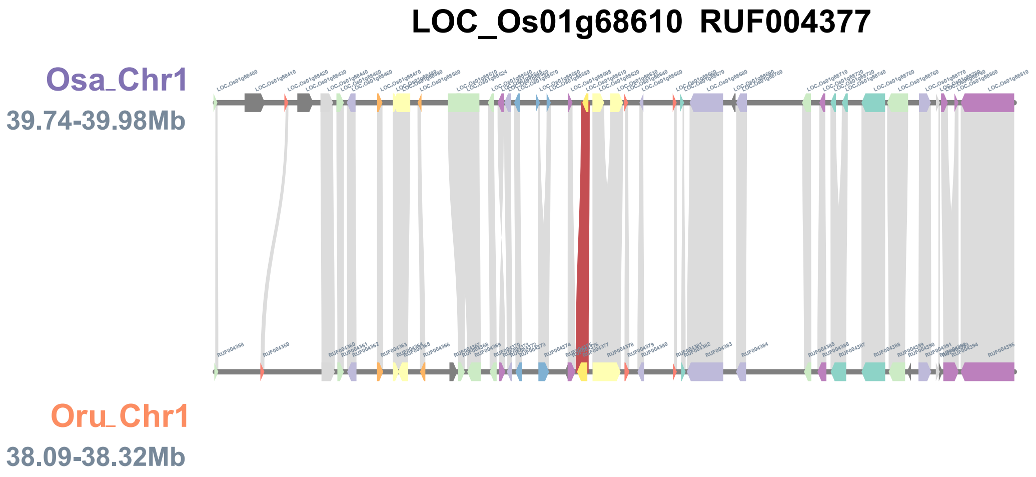
LOC_Os01g68610 RUF004377
Osa_Chr1
39.74-39.98Mb
Oru_Chr1
38.09-38.32Mb

LOC_Os01g71180 RUF004565

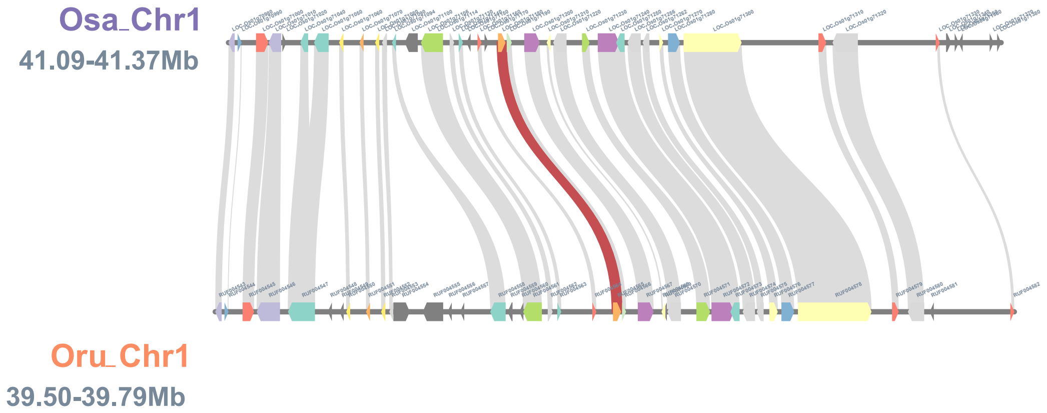

LOC_Os01g72930 RUF004695

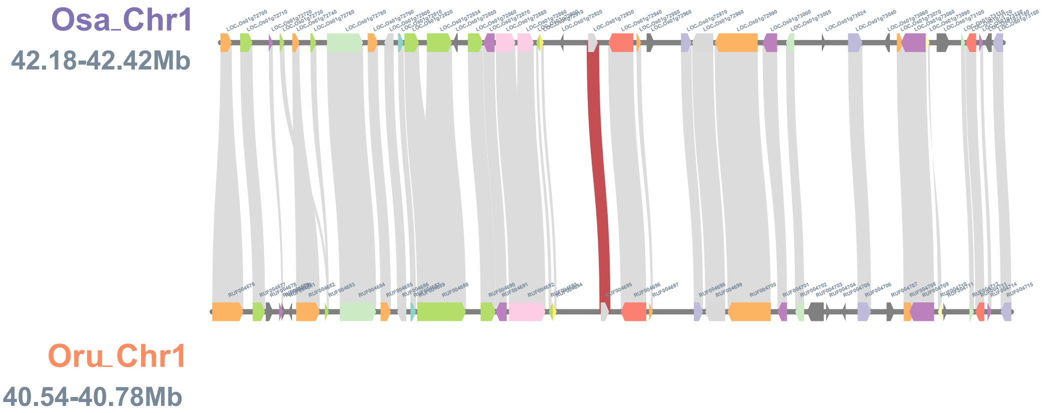

LOC_Os01g73950 RUF004777

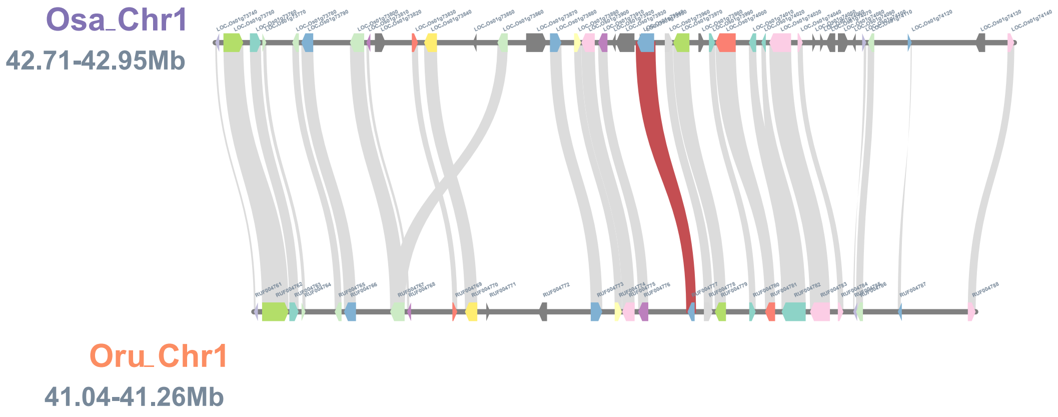

LOC_Os01g74600 RUF004823

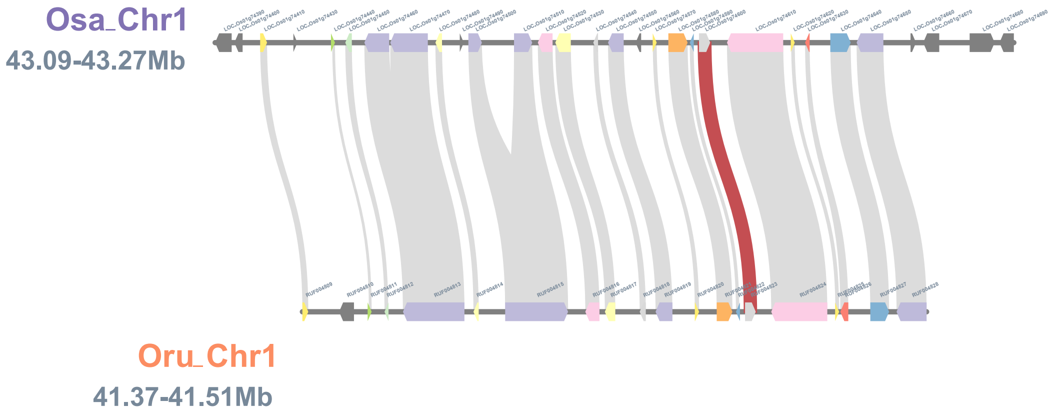

LOC_Os02g01610 RUF004886

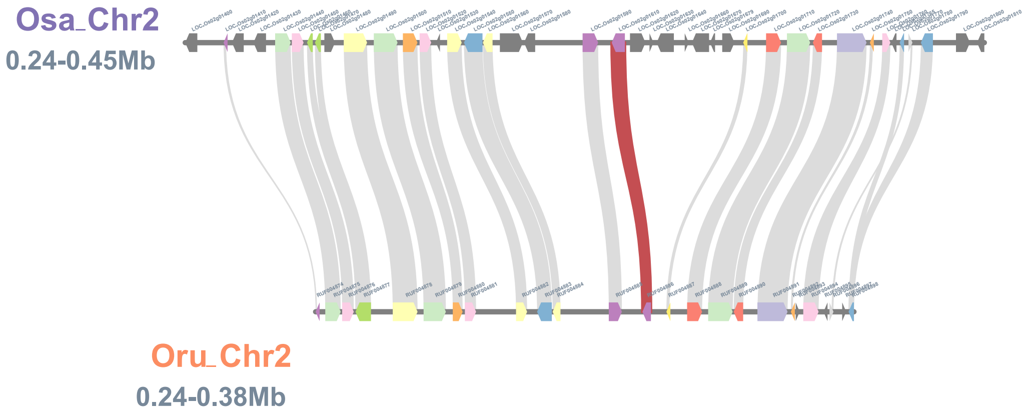

LOC_Os02g02020 RUF004921

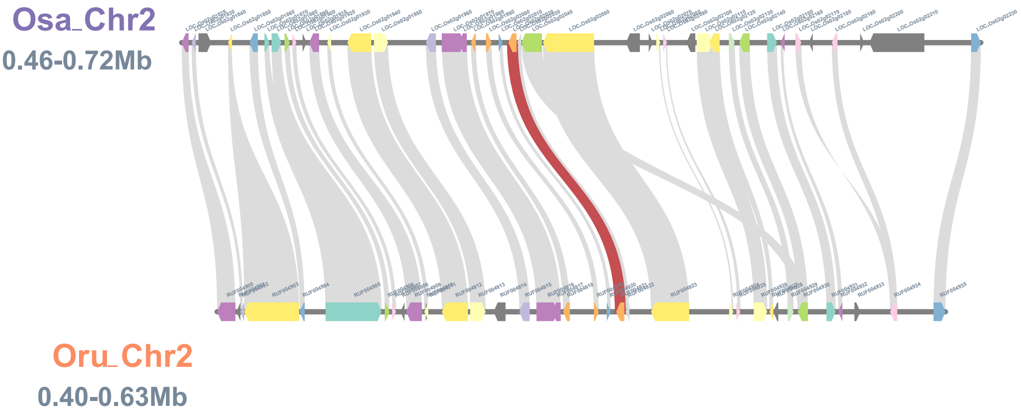

LOC_Os02g02590 RUF004960

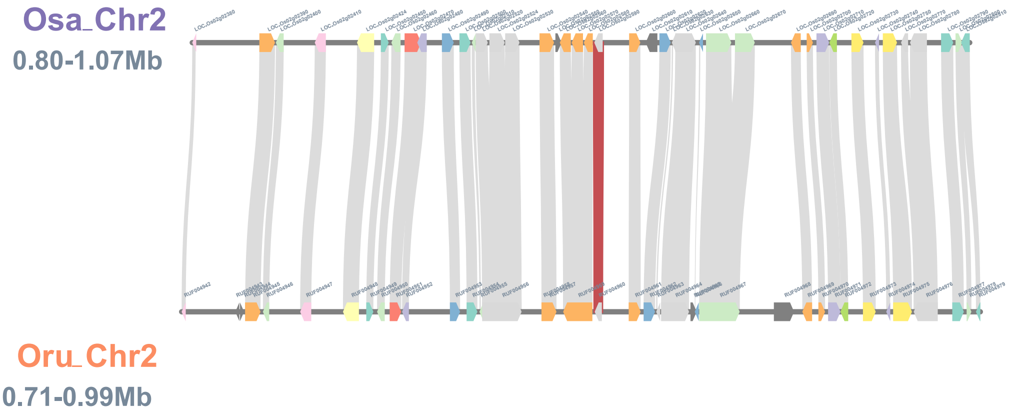

LOC_Os02g02740 RUF004974

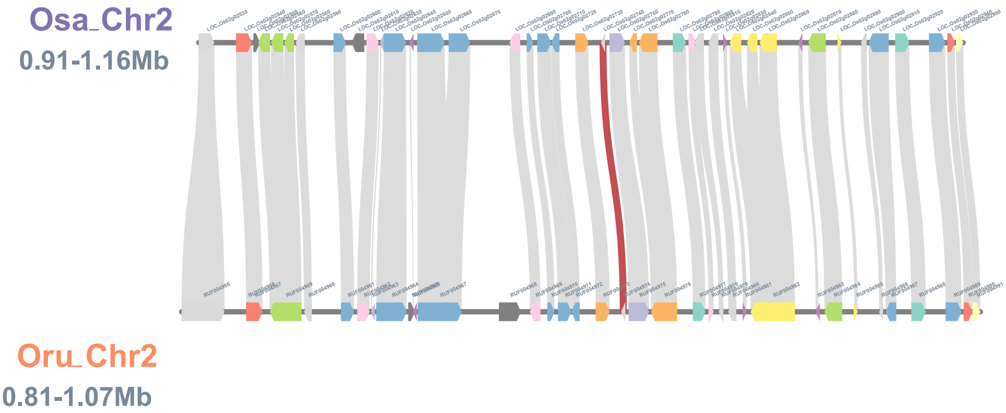

LOC_Os02g02950 RUF004991

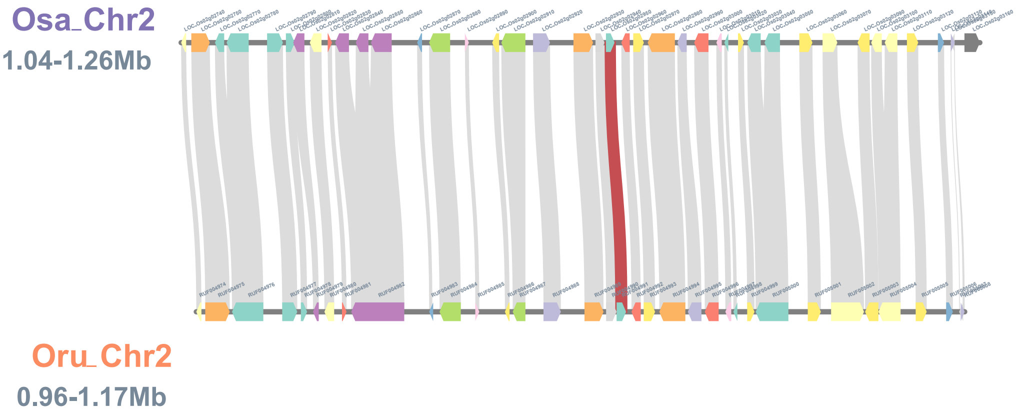

LOC_Os02g03420 RUF005025

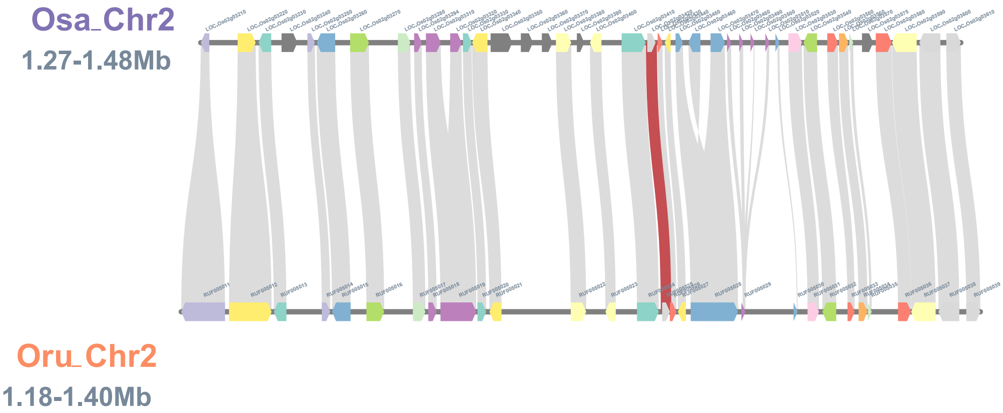

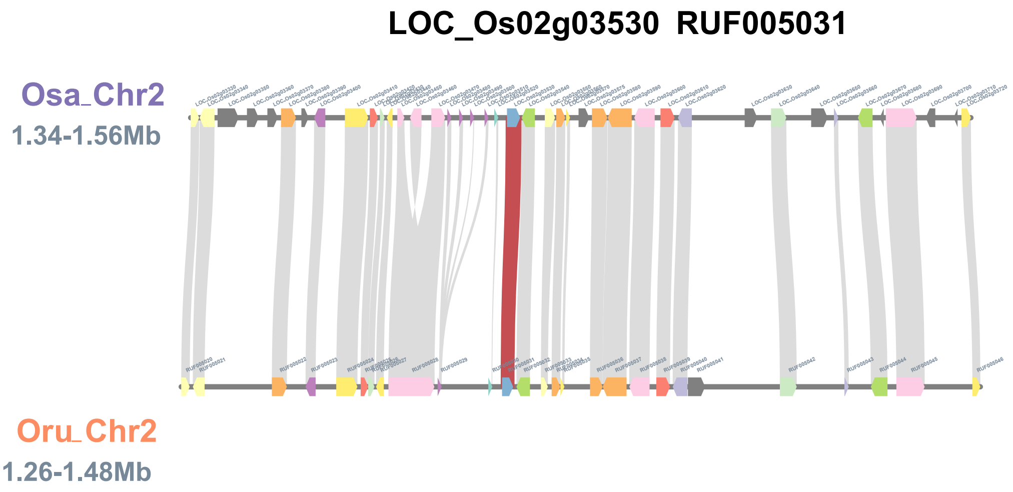

LOC_Os02g03530 RUF005031
Osa_Chr2
1.34-1.56Mb
Oru_Chr2
1.26-1.48Mb

LOC_Os02g03930 RUF005061

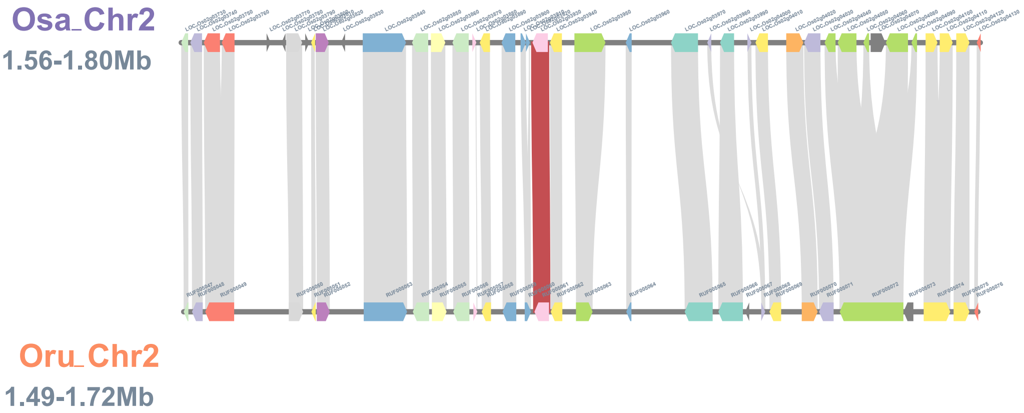

LOC_Os02g03970 RUF005065

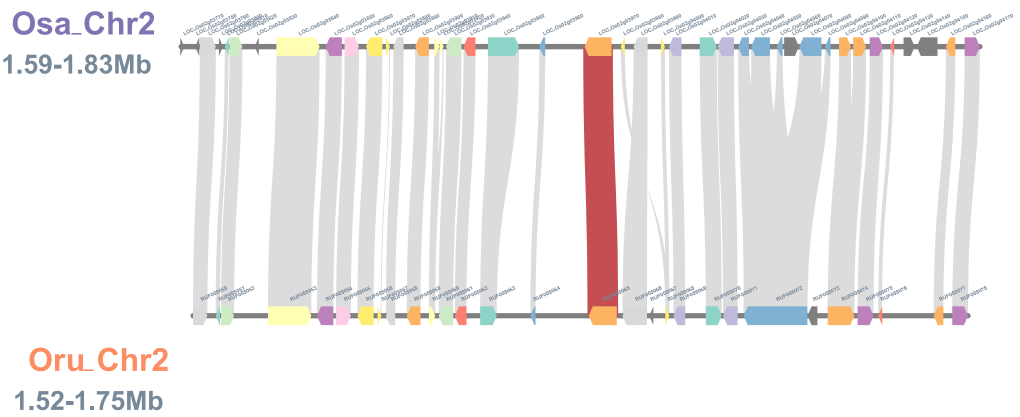

LOC_Os02g05720 RUF005171

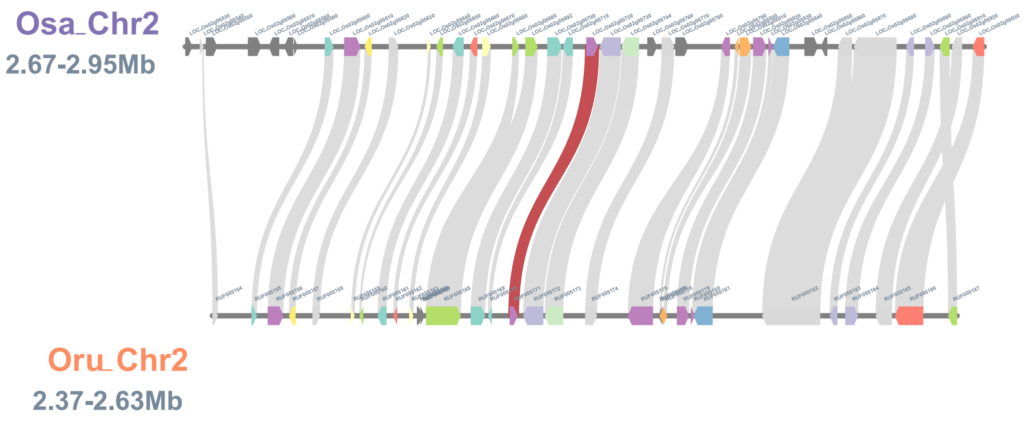

LOC_Os02g06880 RUF005238

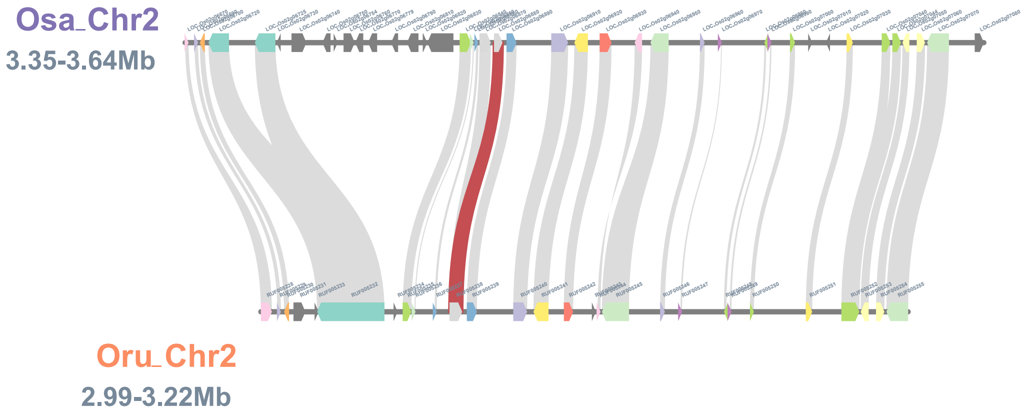

LOC_Os02g07050 RUF005253

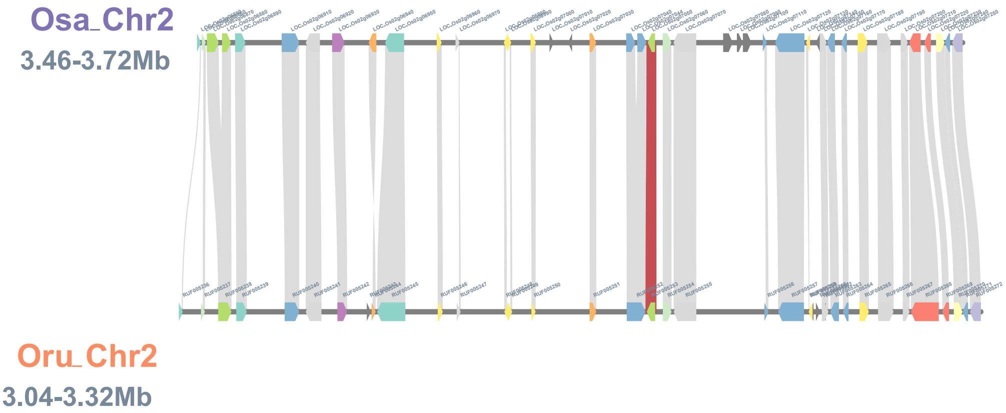

LOC_Os02g09800 RUF005437

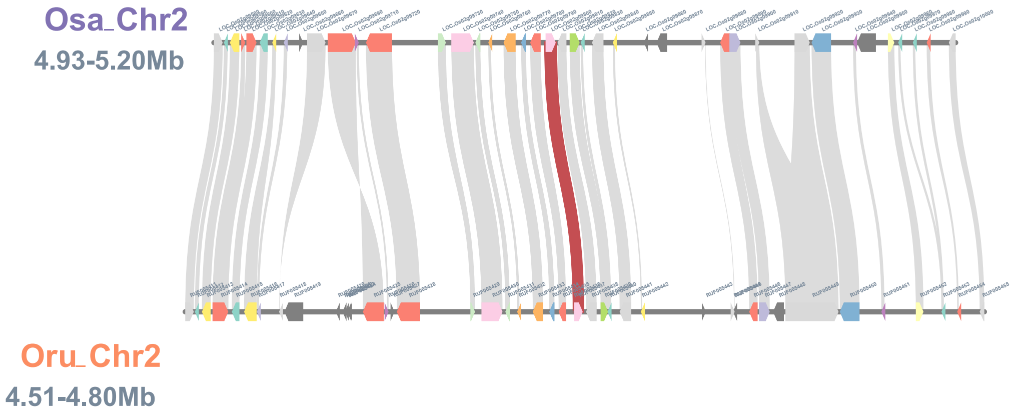

LOC_Os02g13340 RUF005719

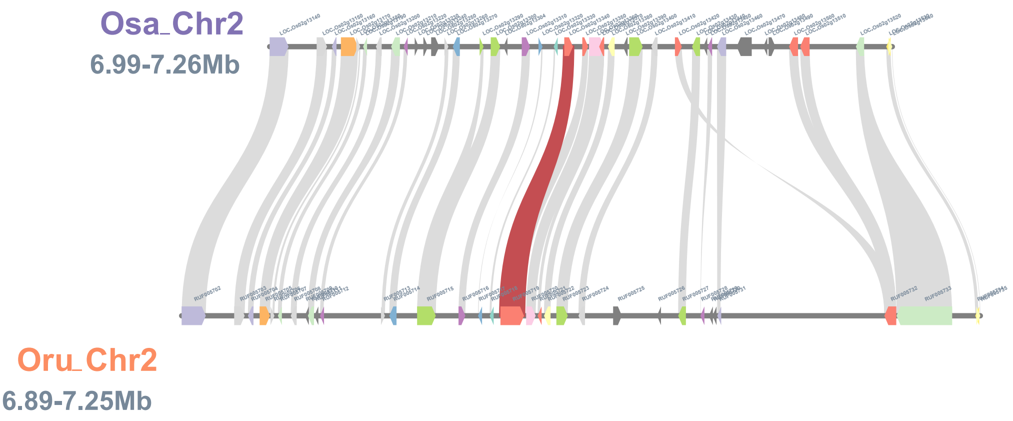

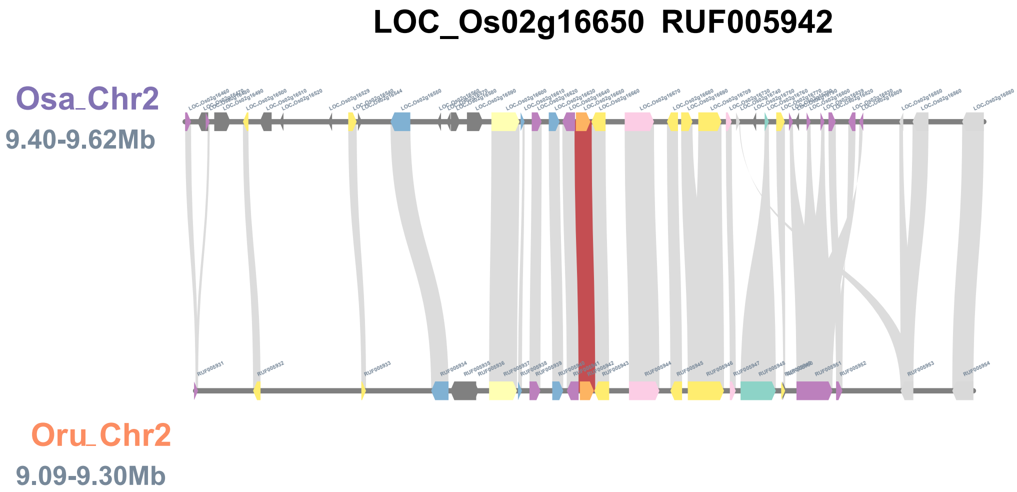
LOC_Os02g16650 RUF005942
Osa_Chr2
9.40-9.62Mb
Oru_Chr2
9.09-9.30Mb

LOC_Os02g20160 RUF006133

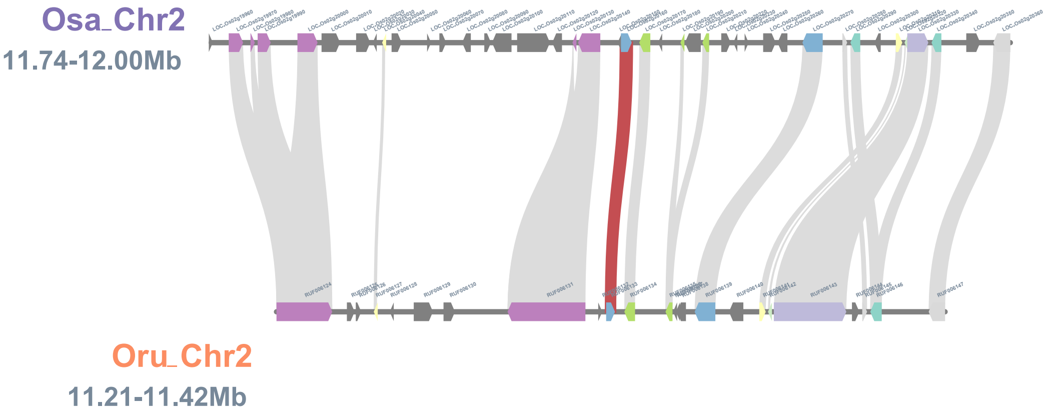

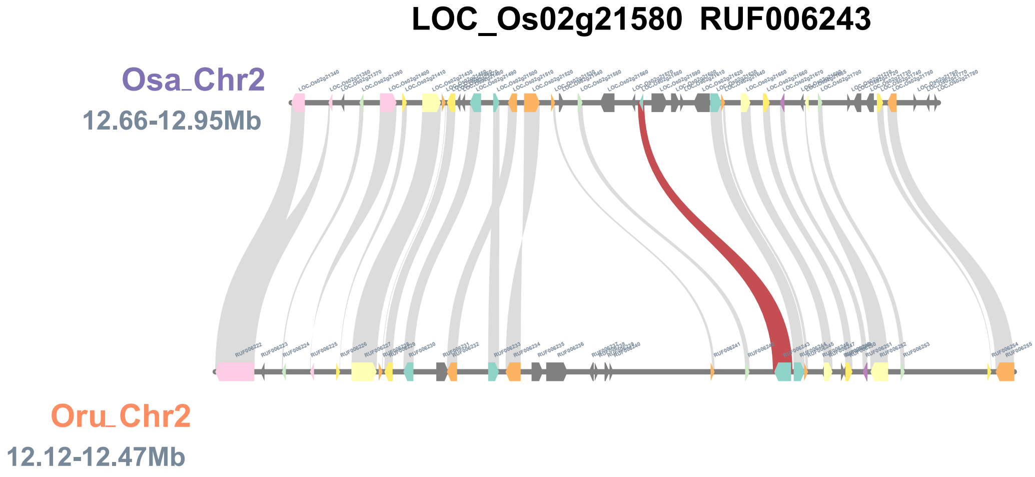
LOC_Os02g21580 RUF006243
Osa_Chr2
12.66-12.95Mb
Oru_Chr2
12.12-12.47Mb

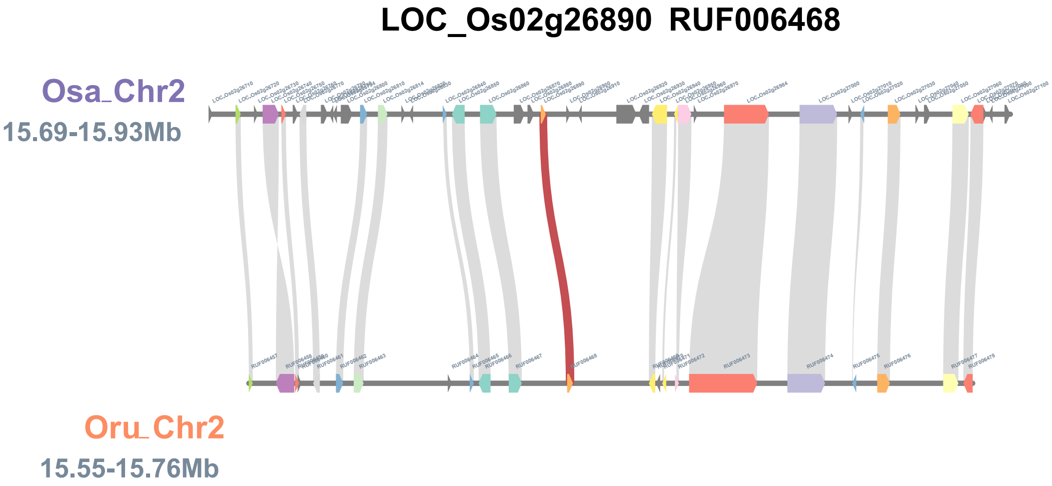
LOC_Os02g26890 RUF006468
Osa_Chr2
15.69-15.93Mb
Oru_Chr2
15.55-15.76Mb

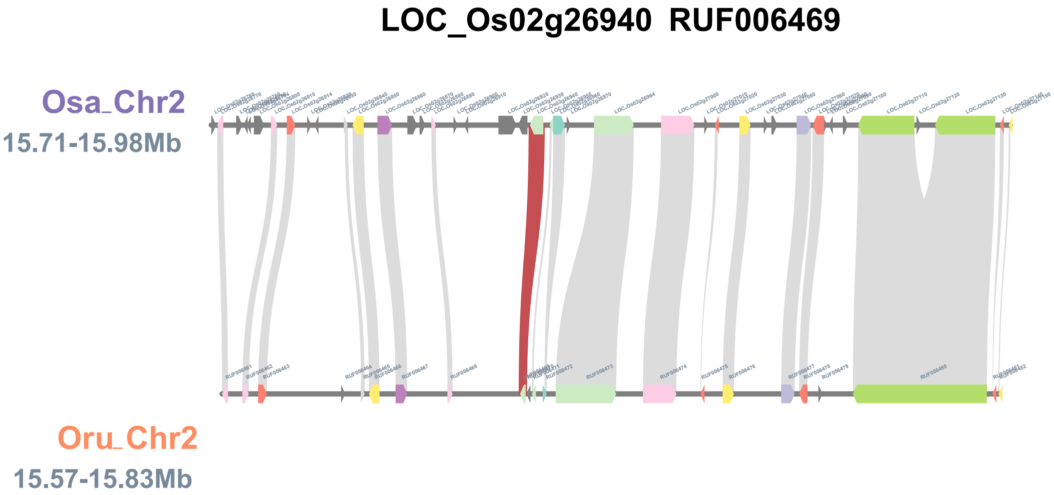
LOC_Os02g26940 RUF006469
Osa_Chr2
15.71-15.98Mb
Oru_Chr2
15.57-15.83Mb

LOC_Os02g27070 RUF006478

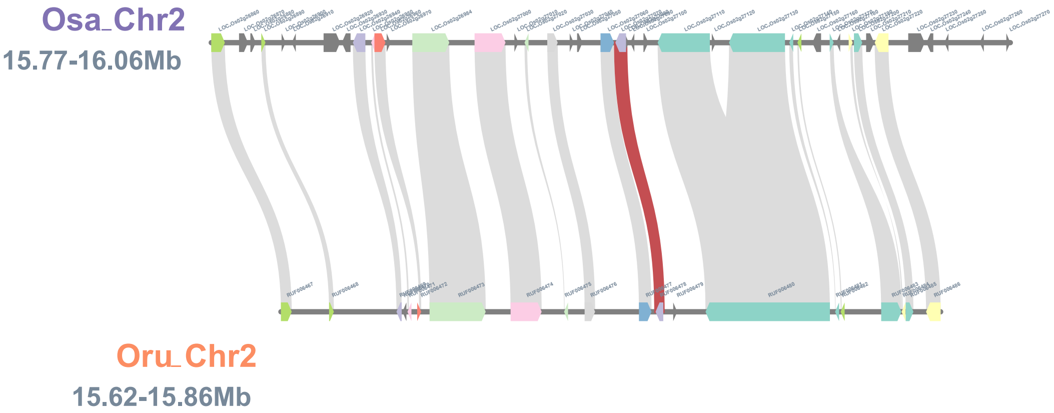

LOC_Os02g32780 RUF006808

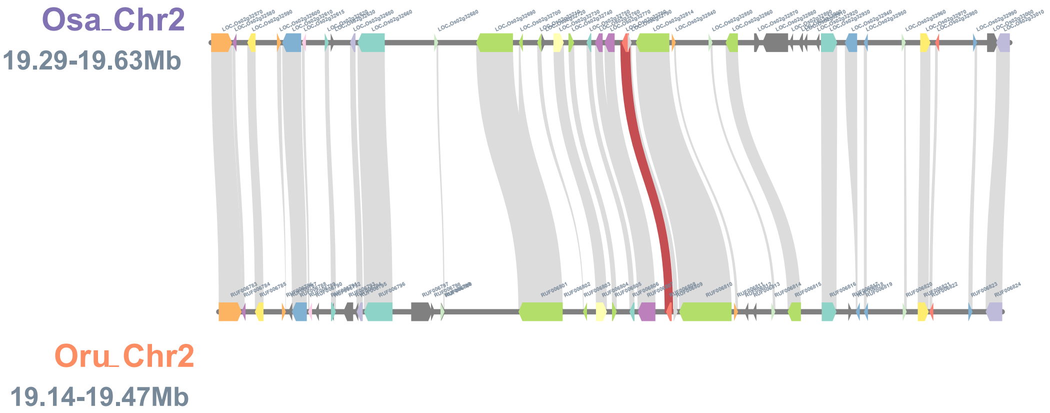

LOC_Os02g34960 RUF006956

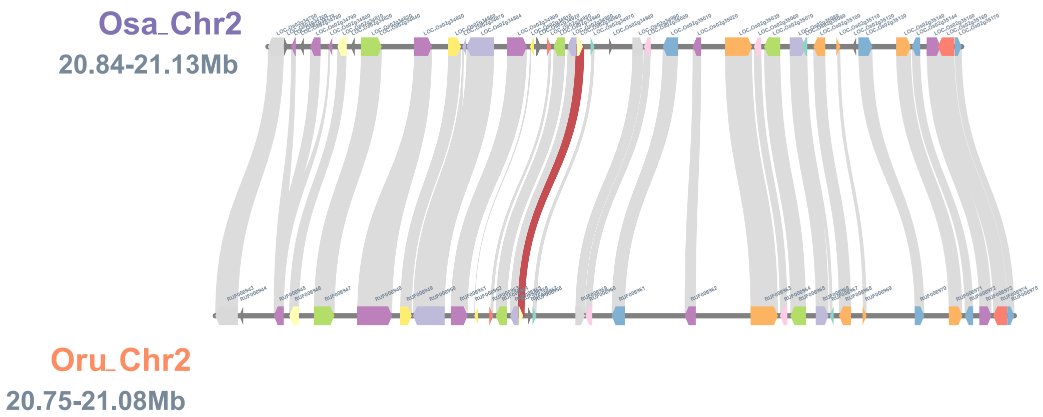

LOC_Os02g35750 RUF007019

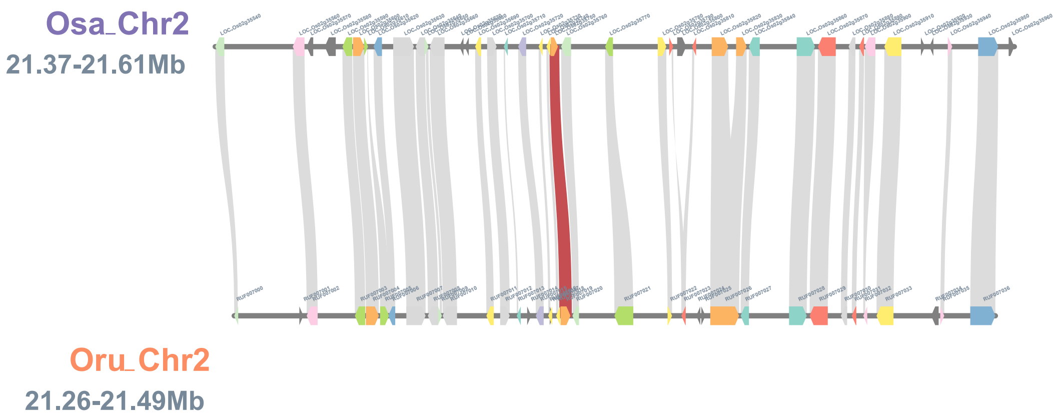

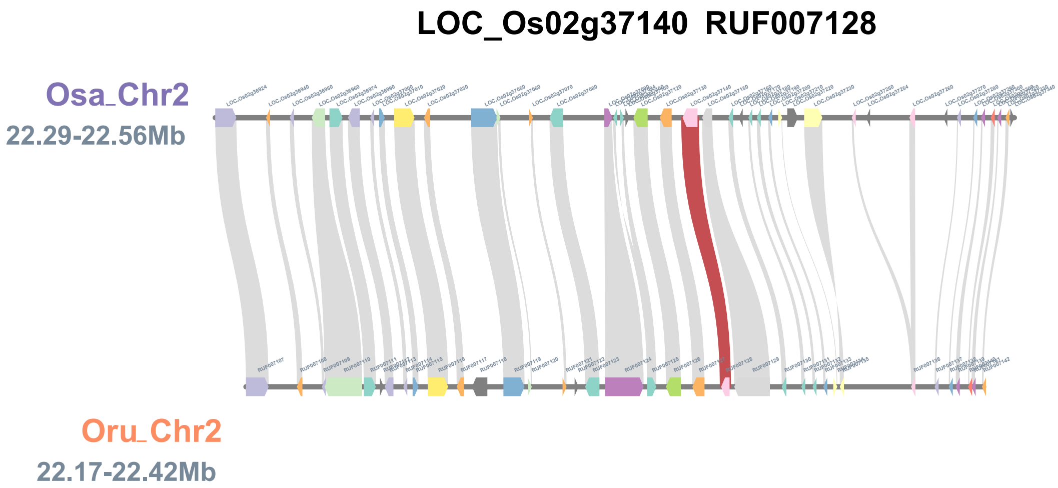

LOC_Os02g37140 RUF007128
Osa_Chr2
22.29-22.56Mb
Oru_Chr2
22.17-22.42Mb

LOC_Os02g38950 RUF007254

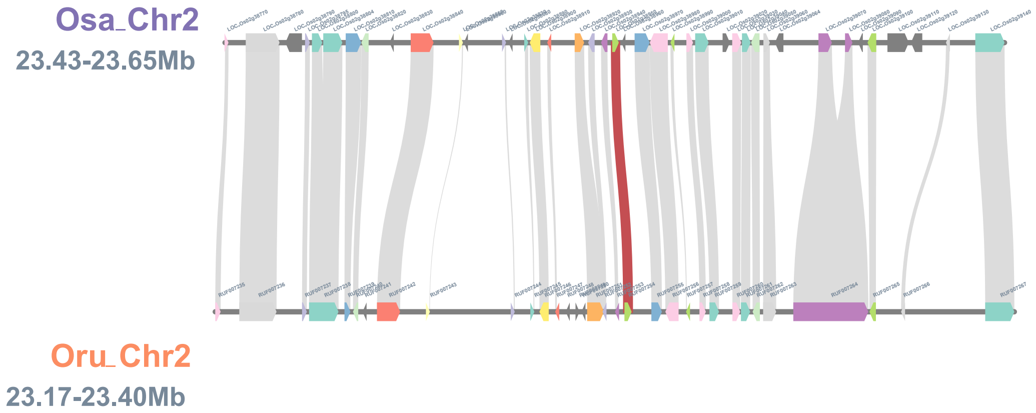

LOC_Os02g39820 RUF007343

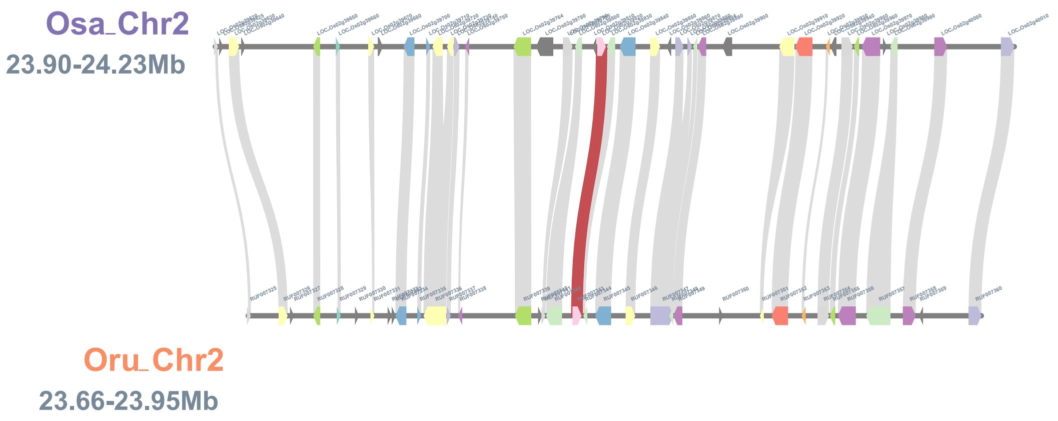

LOC_Os02g40750 RUF007418

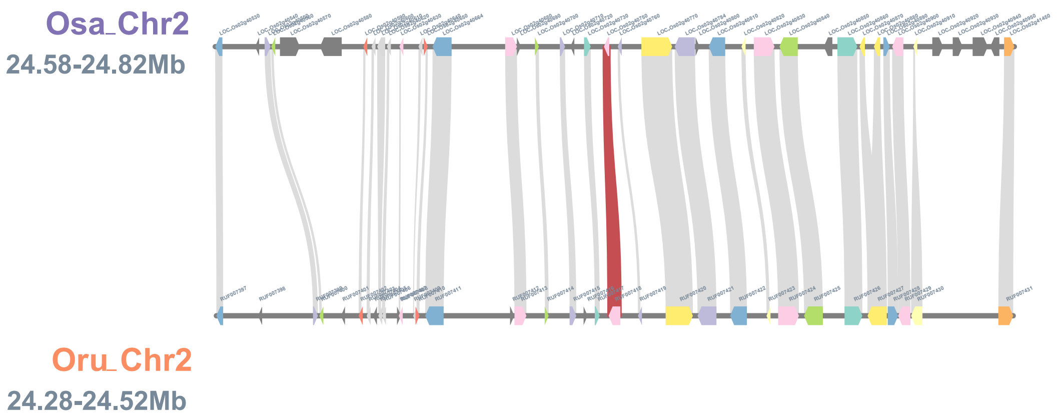

LOC_Os02g41560 RUF007444

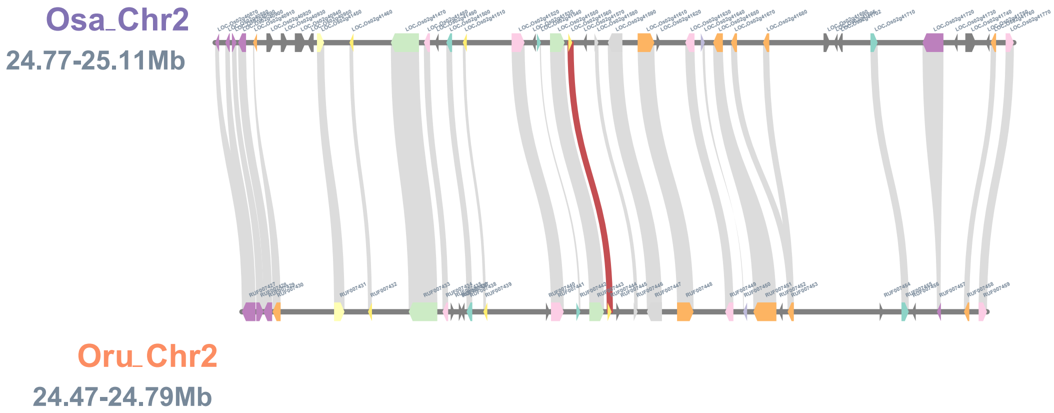

LOC_Os02g42210 RUF007492

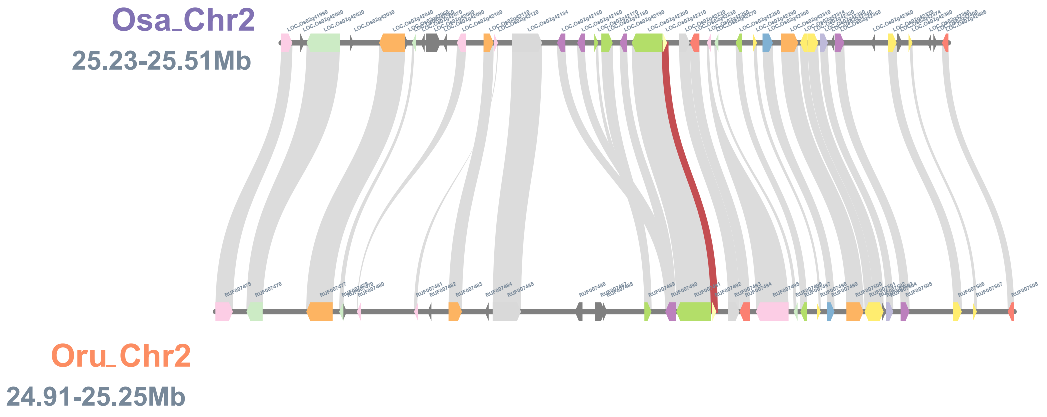

LOC_Os02g44970 RUF007722

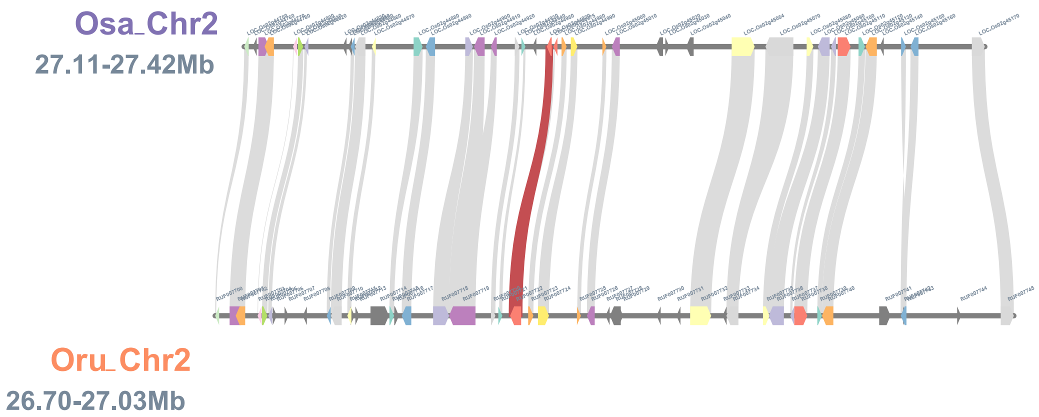

LOC_Os02g45590 RUF007784

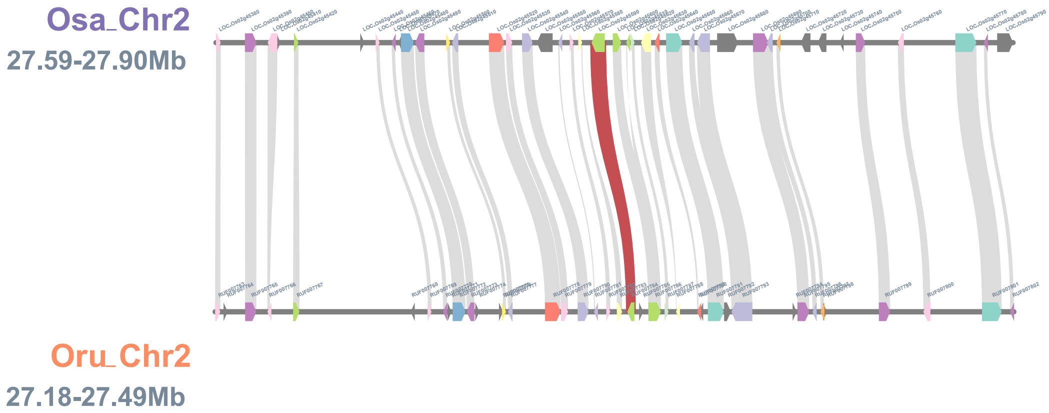

LOC_Os02g46980 RUF007899

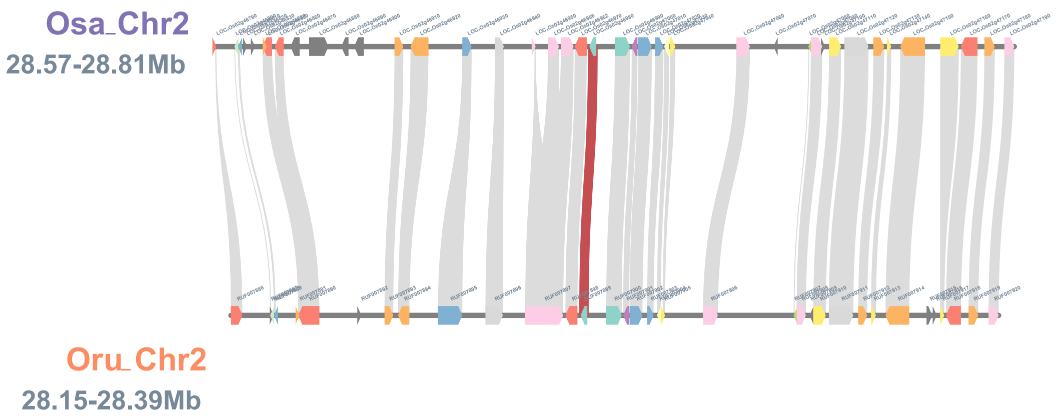

LOC_Os02g47360 RUF007932

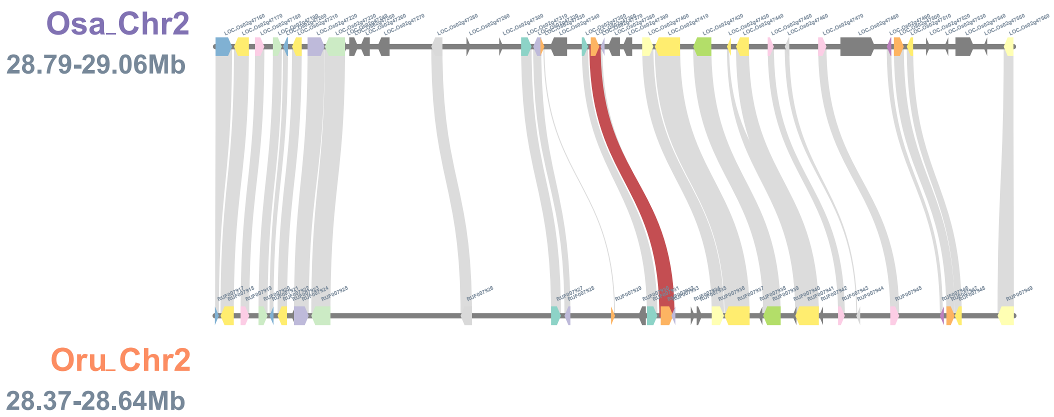

LOC_Os02g48122 RUF007999

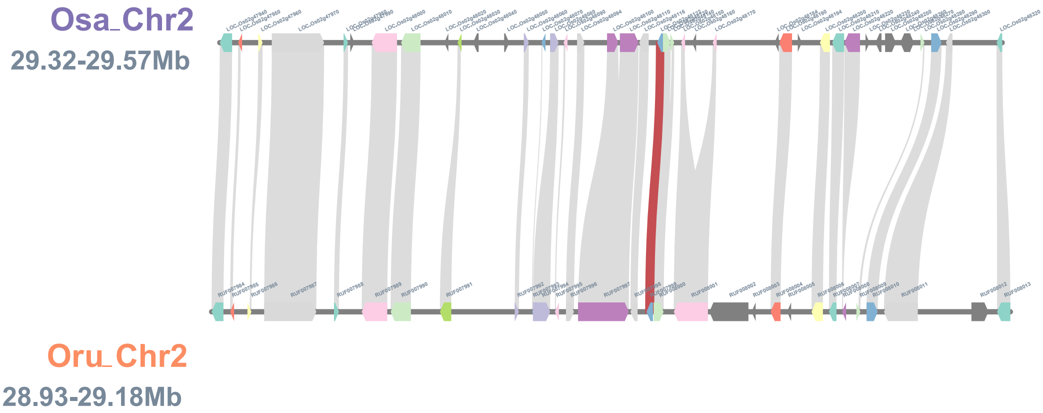

LOC_Os02g49830 RUF008147

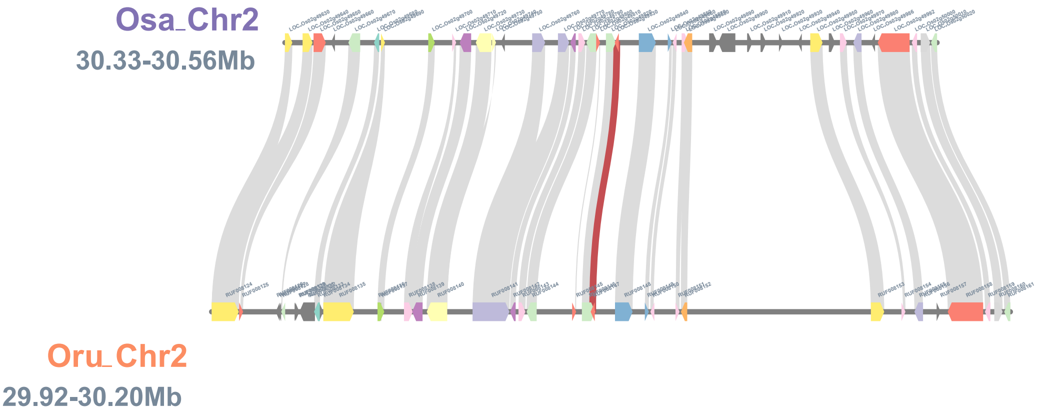

LOC_Os02g50280 RUF008181

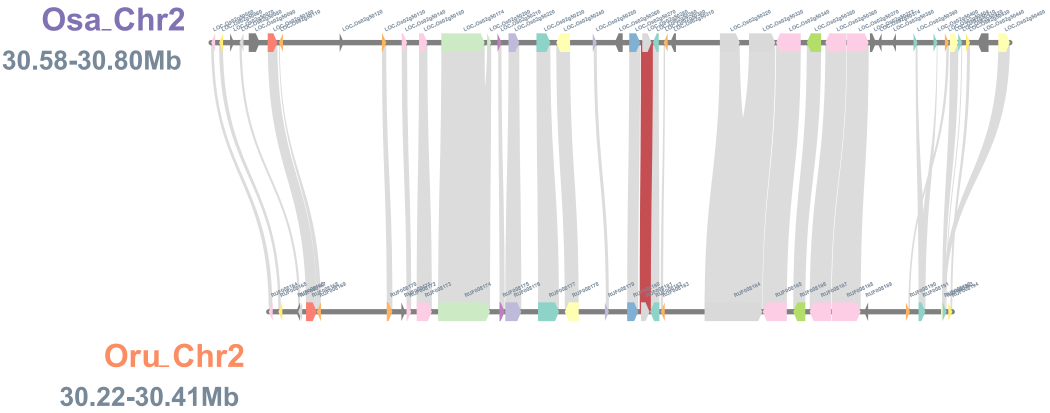

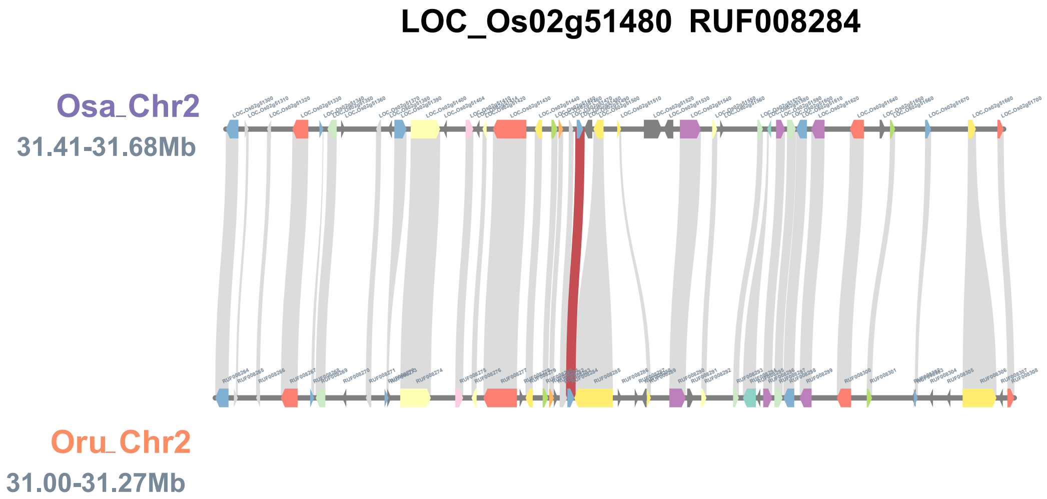
LOC_Os02g51480 RUF008284
Osa_Chr2
31.41-31.68Mb
Oru_Chr2
31.00-31.27Mb

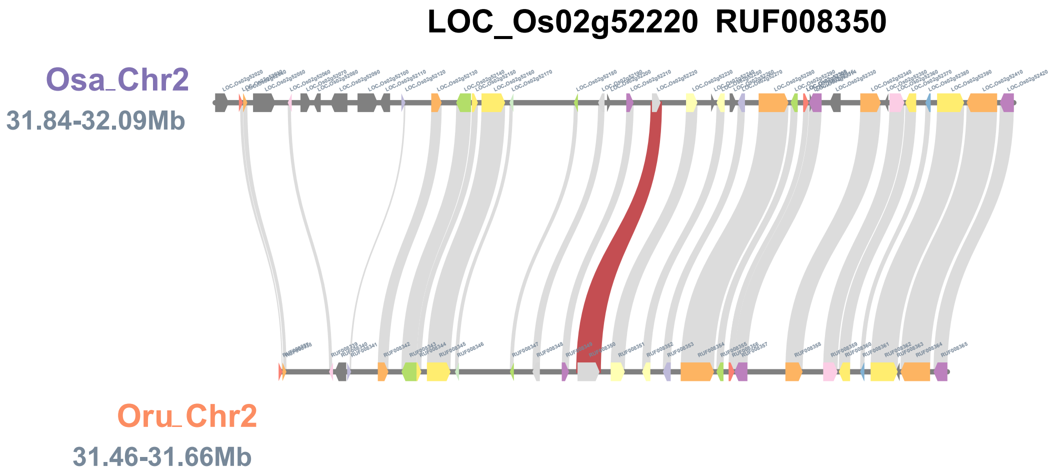

LOC_Os02g52220 RUF008350
Osa_Chr2
31.84-32.09Mb
Oru_Chr2
31.46-31.66Mb

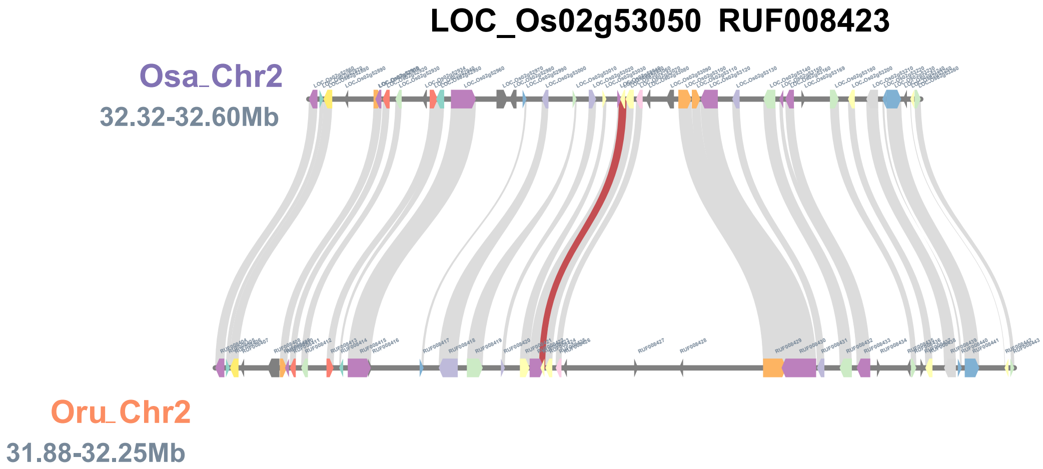
LOC_Os02g53050 RUF008423
Osa_Chr2
32.32-32.60Mb
Oru_Chr2
31.88-32.25Mb

LOC_Os02g55020 RUF008614

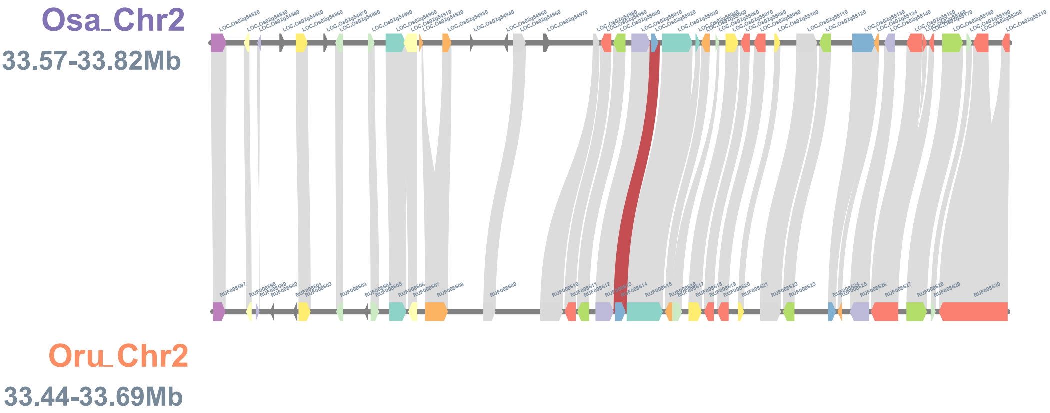

LOC_Os02g55310 RUF008638

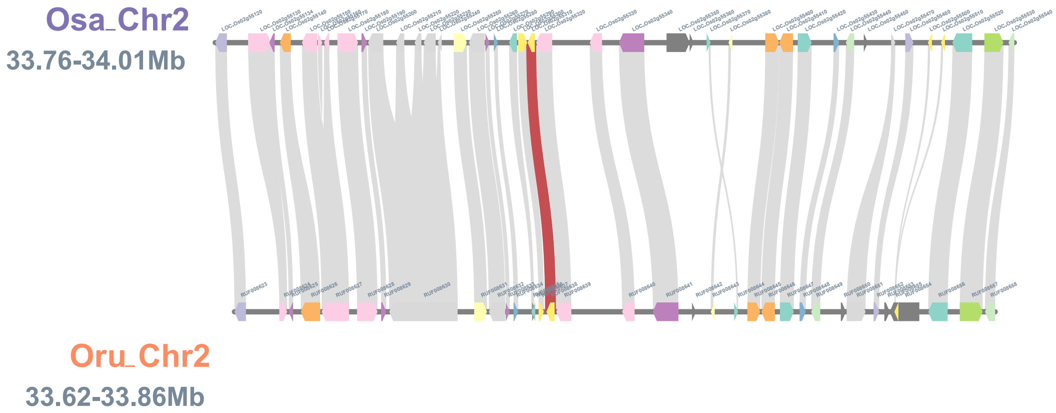

LOC_Os02g57340 RUF008792

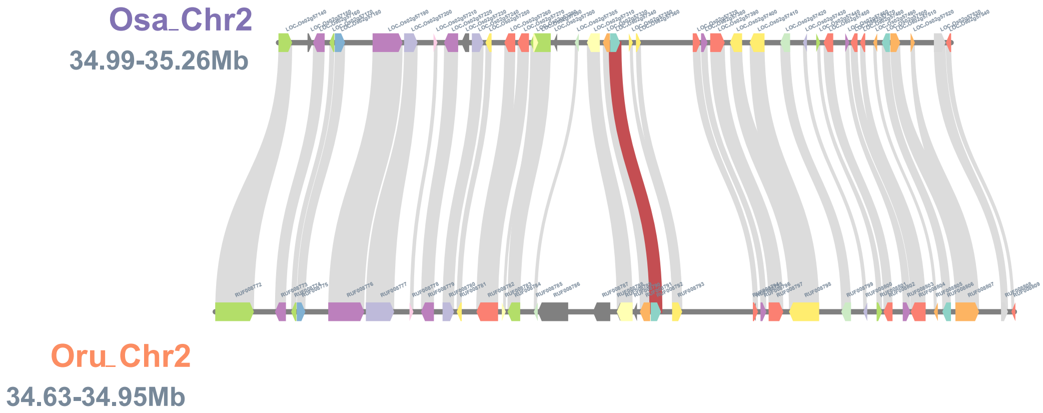

LOC_Os02g57800 RUF008832

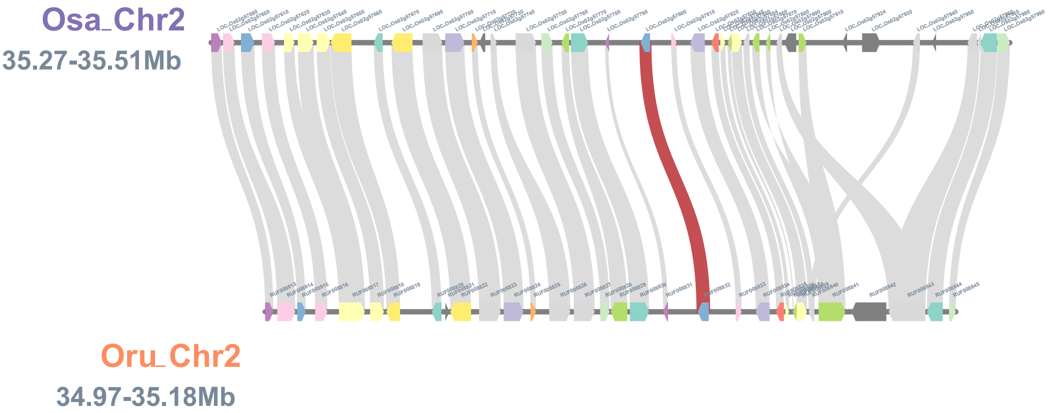

LOC_Os02g58139 RUF008856

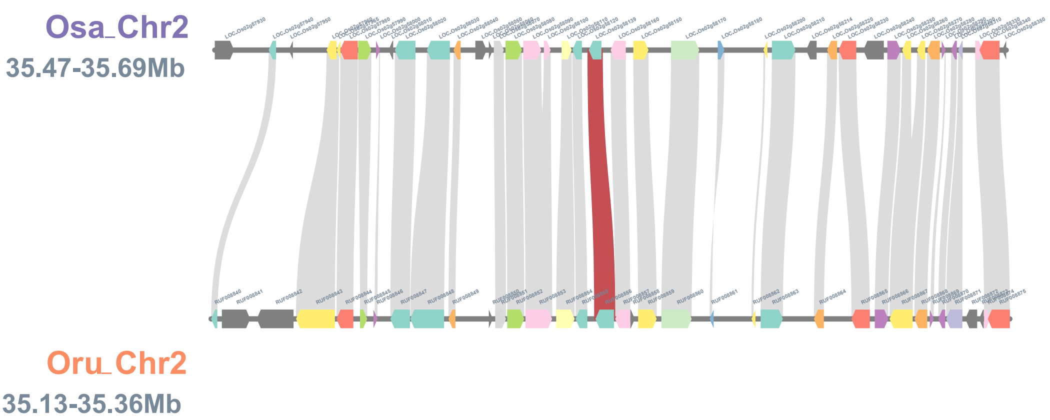

LOC_Os02g58300 RUF008870

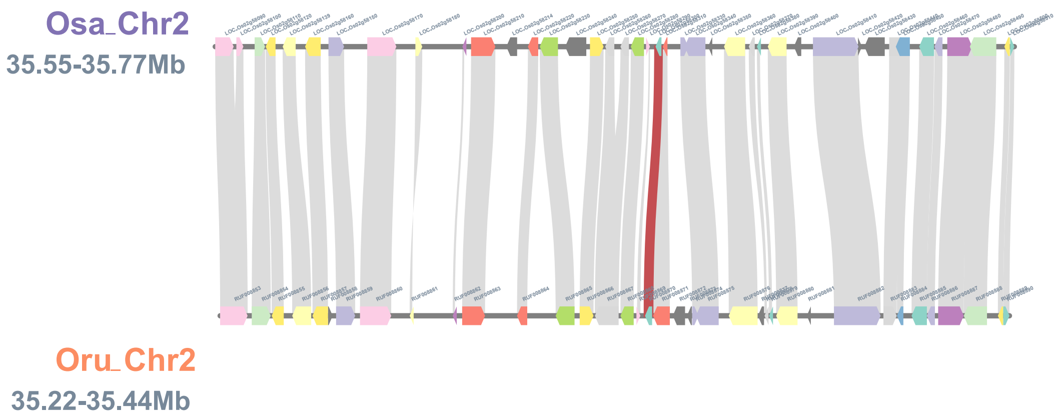

LOC_Os02g58330 RUF008874

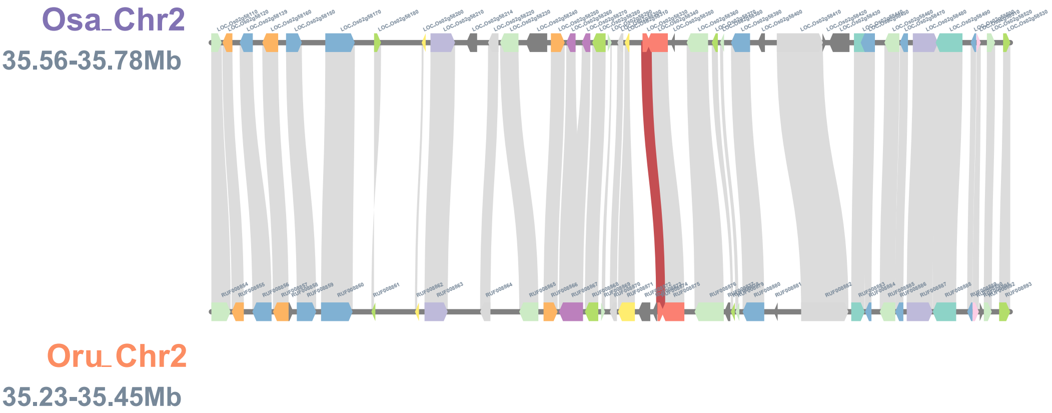

LOC_Os02g58620 RUF008903

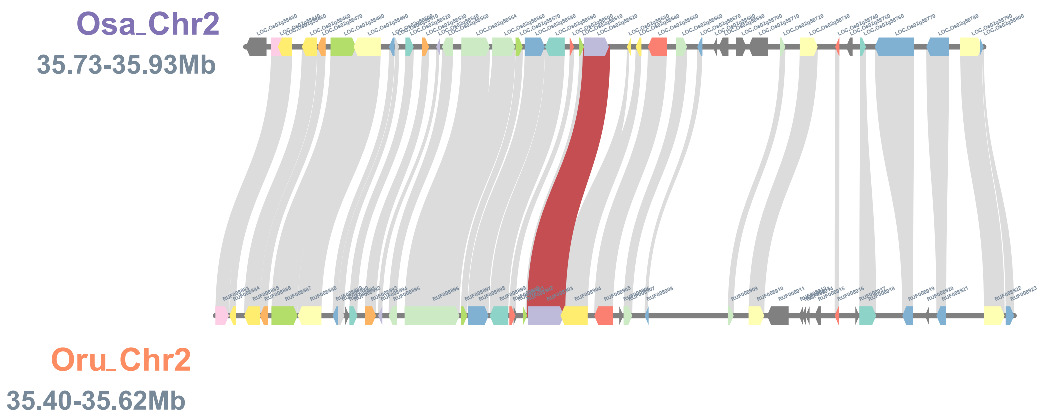

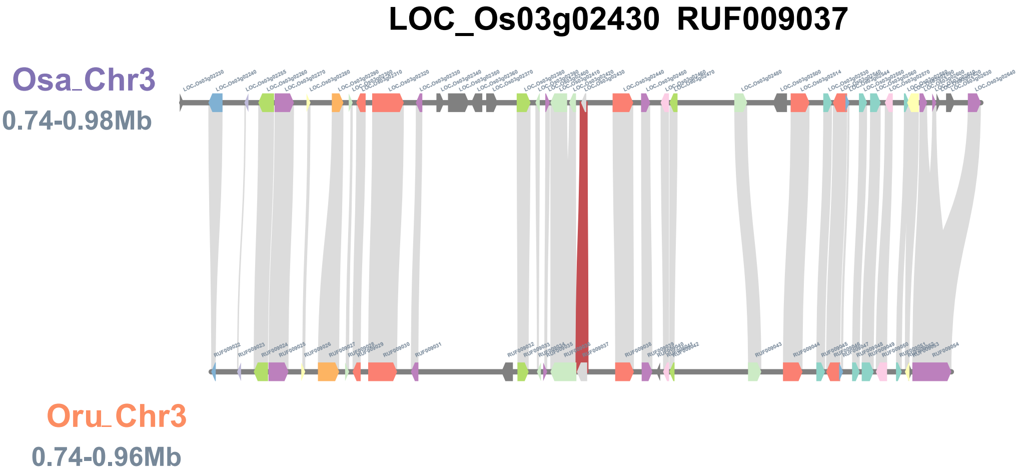
LOC_Os03g02430 RUF009037
Osa_Chr3
0.74-0.98Mb
Oru_Chr3
0.74-0.96Mb

LOC_Os03g04390 RUF009148

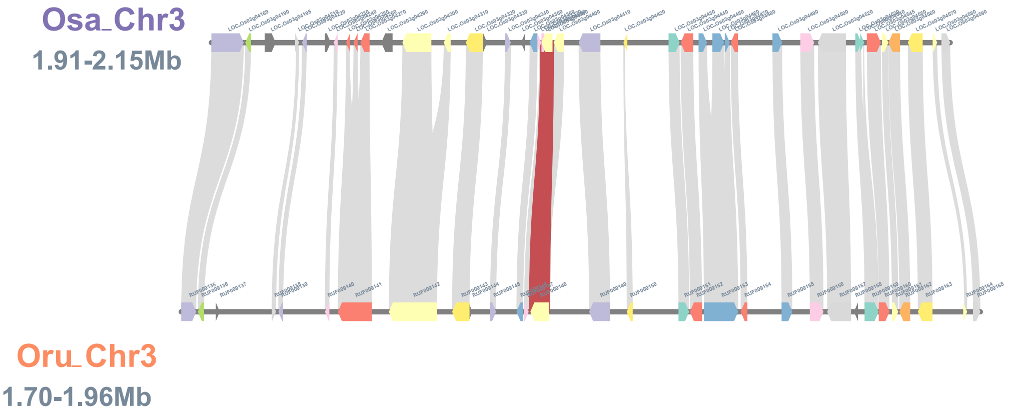

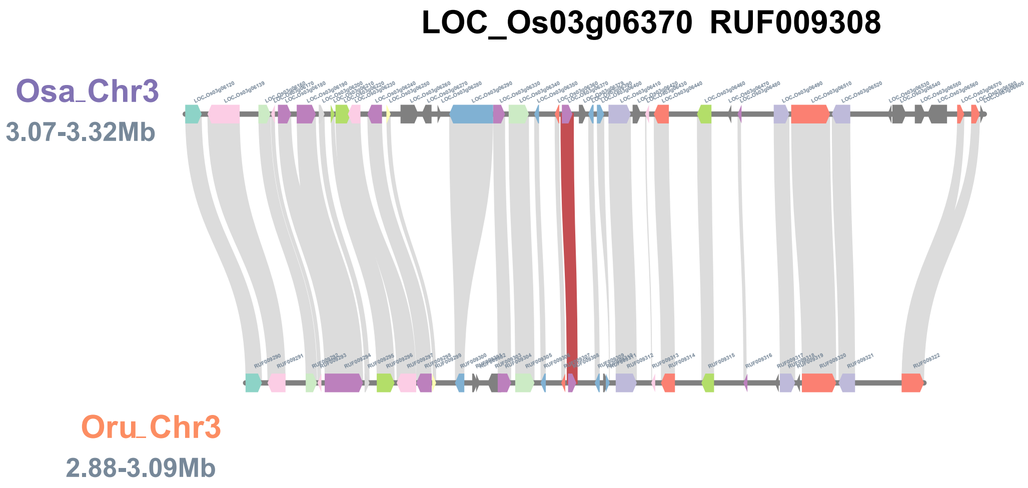
LOC_Os03g06370 RUF009308
Osa_Chr3
3.07-3.32Mb
Oru_Chr3
2.88-3.09Mb

LOC_Os03g06910 RUF009348

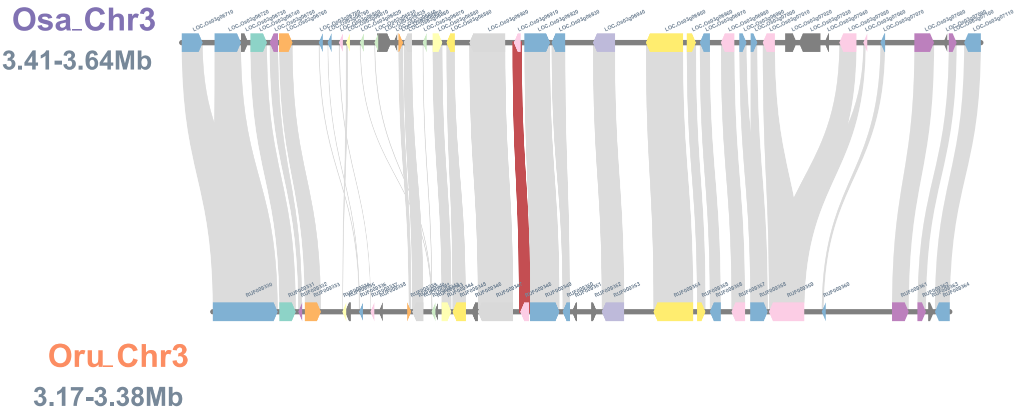

LOC_Os03g07220 RUF009375

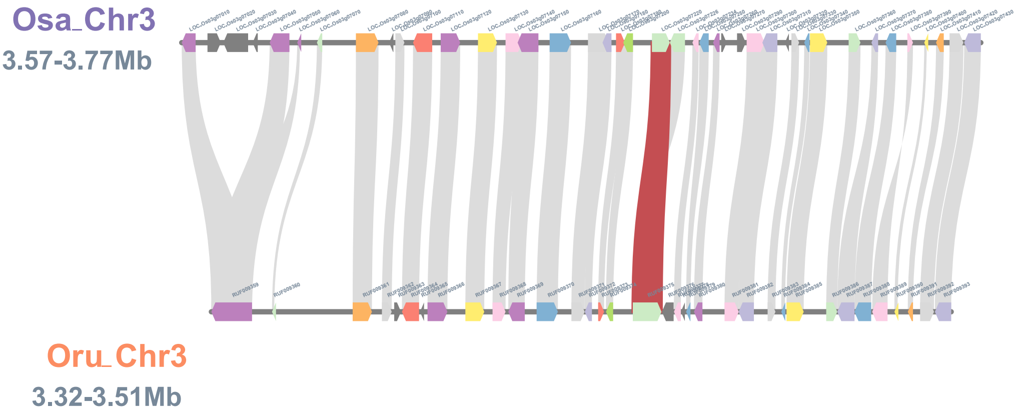

LOC_Os03g07234 RUF009377

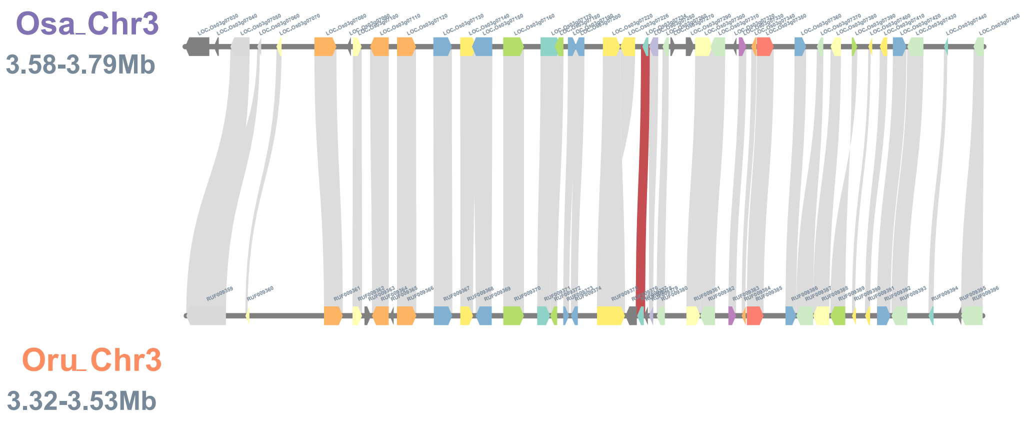

LOC_Os03g07330 RUF009383

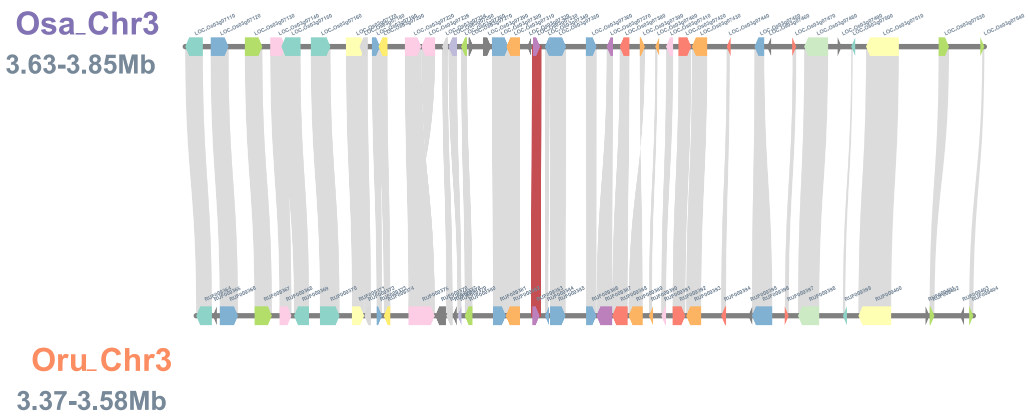

LOC_Os03g08680 RUF009498

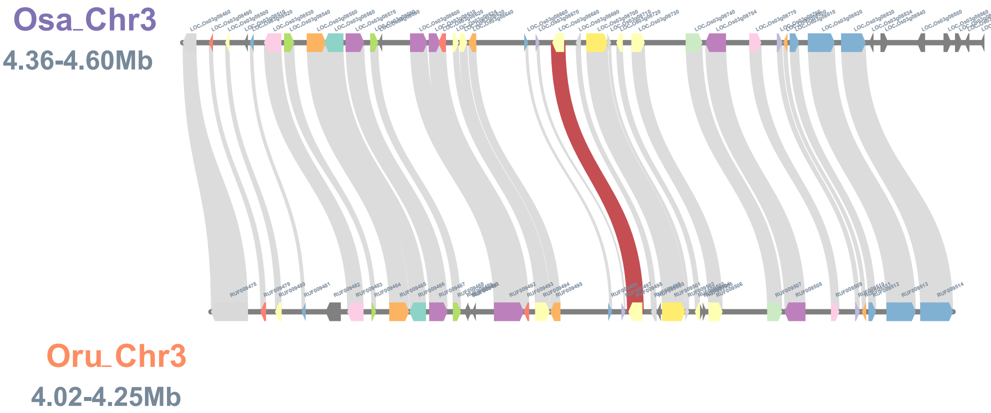

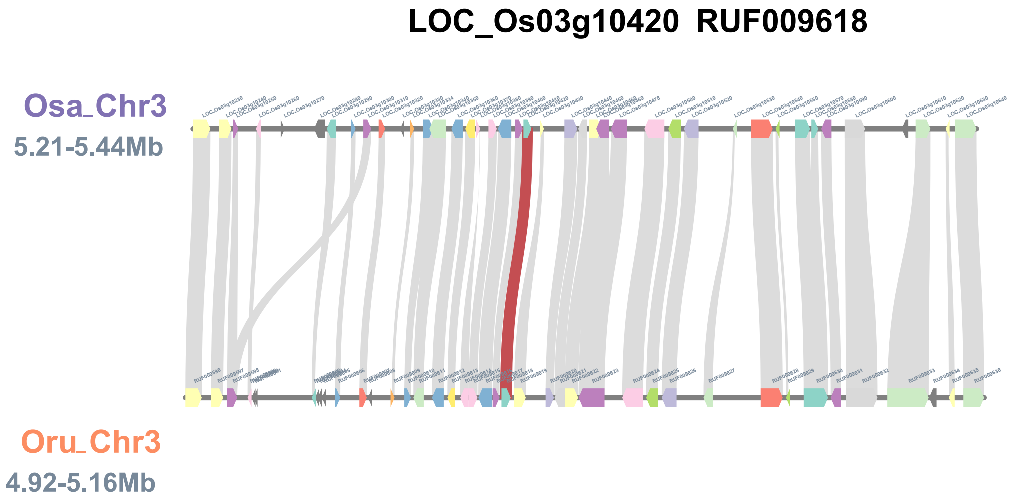
LOC_Os03g10420 RUF009618
Osa_Chr3
5.21-5.44Mb
Oru_Chr3
4.92-5.16Mb

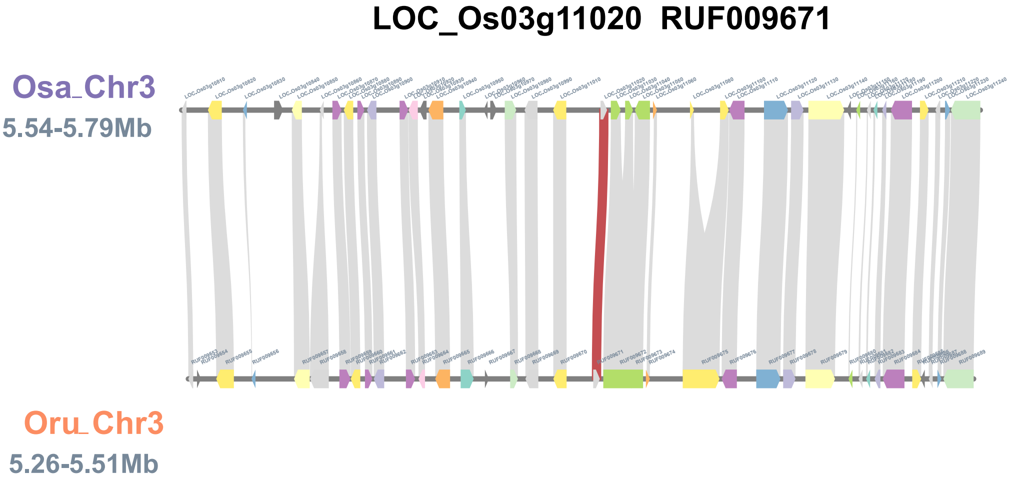
LOC_Os03g11020 RUF009671
Osa_Chr3
5.54-5.79Mb
Oru_Chr3
5.26-5.51Mb

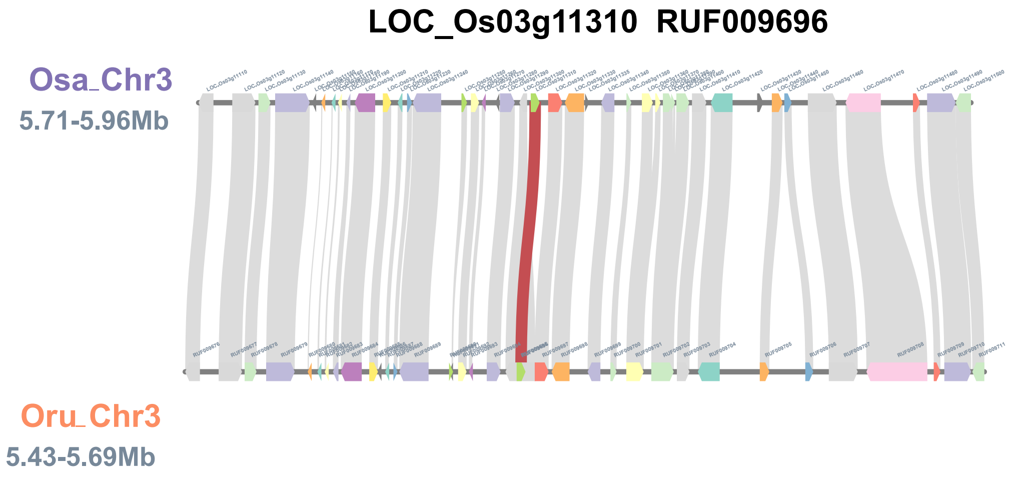
LOC_Os03g11310 RUF009696
Osa_Chr3
5.71-5.96Mb
Oru_Chr3
5.43-5.69Mb

LOC_Os03g11670 RUF009725

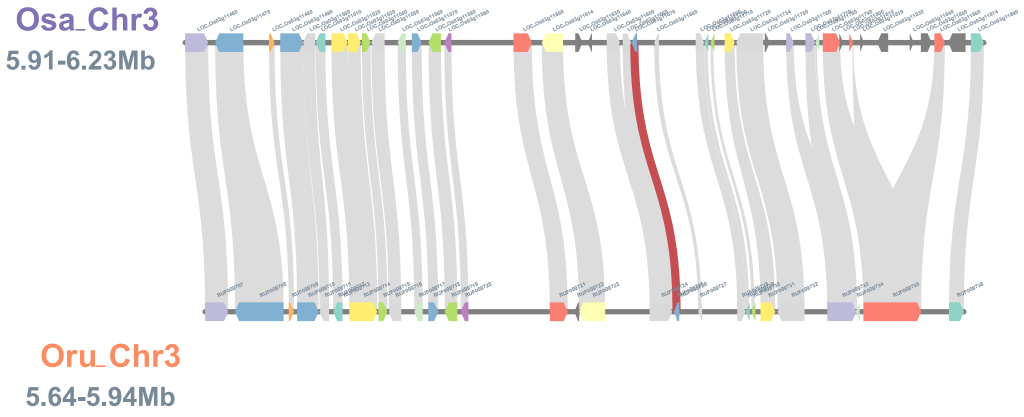

LOC_Os03g11690 RUF009728

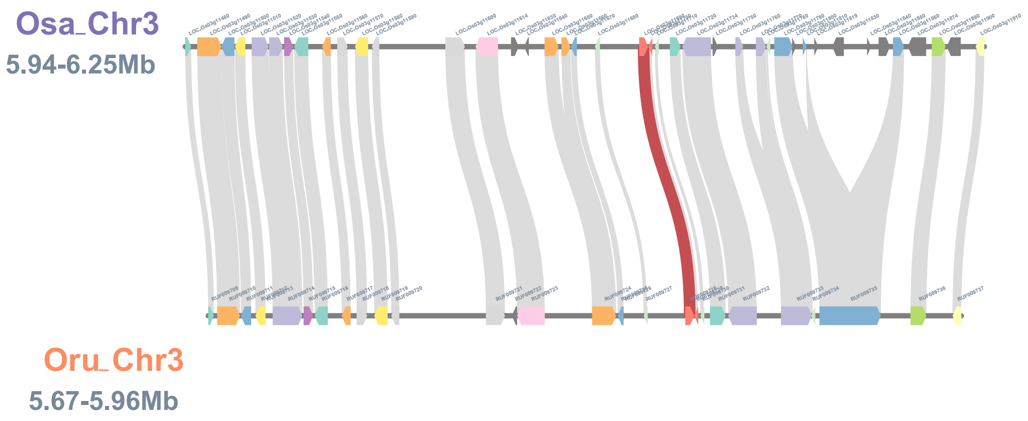

LOC_Os03g11700 RUF009729

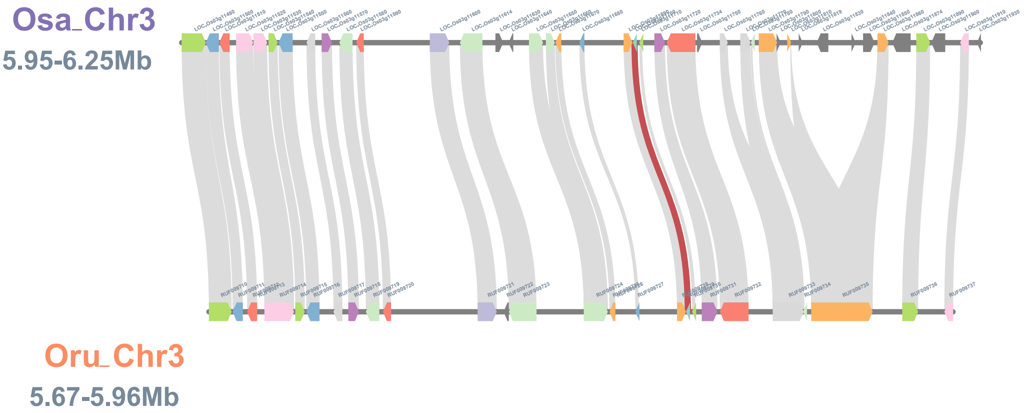

LOC_Os03g12430 RUF009787

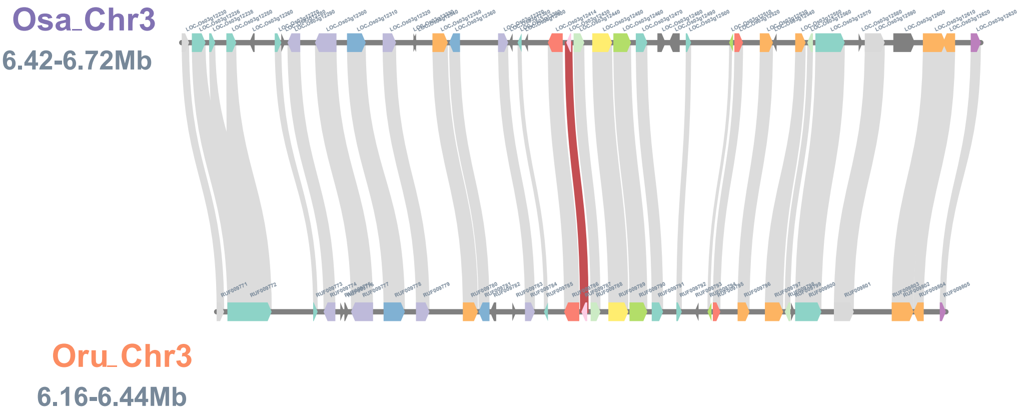

LOC_Os03g13230 RUF009850

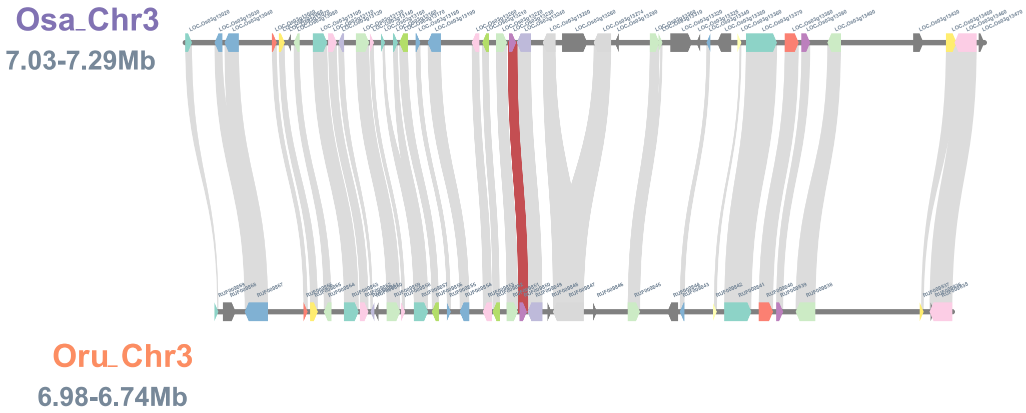

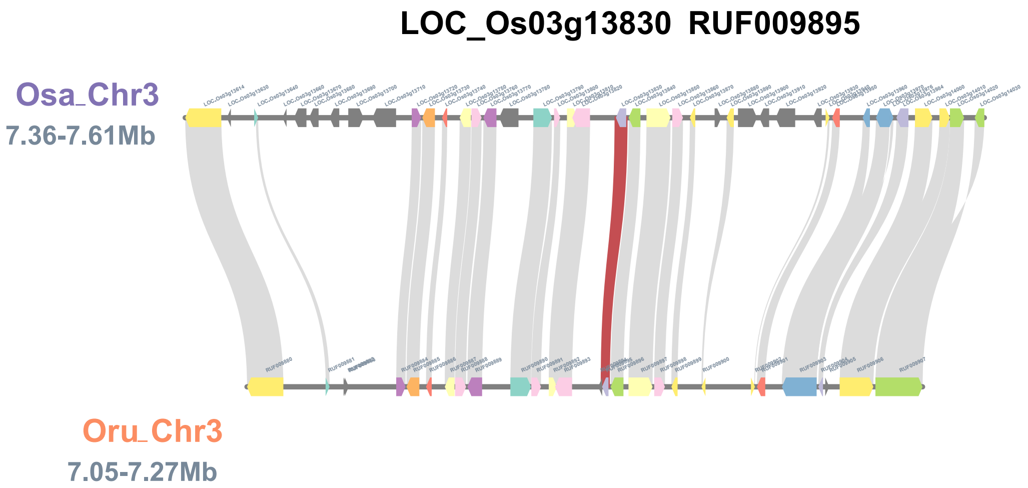
LOC_Os03g13830 RUF009895
Osa_Chr3
7.36-7.61Mb
Oru_Chr3
7.05-7.27Mb

LOC_Os03g17634 RUF010179

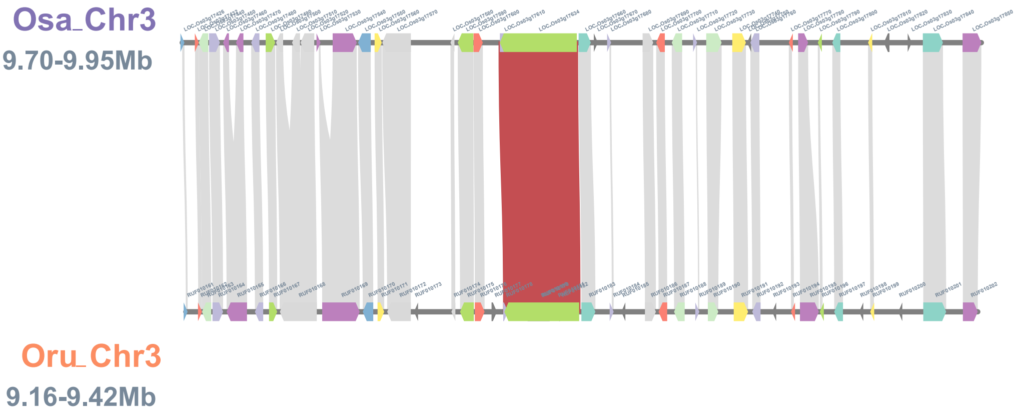

LOC_Os03g19650 RUF010360

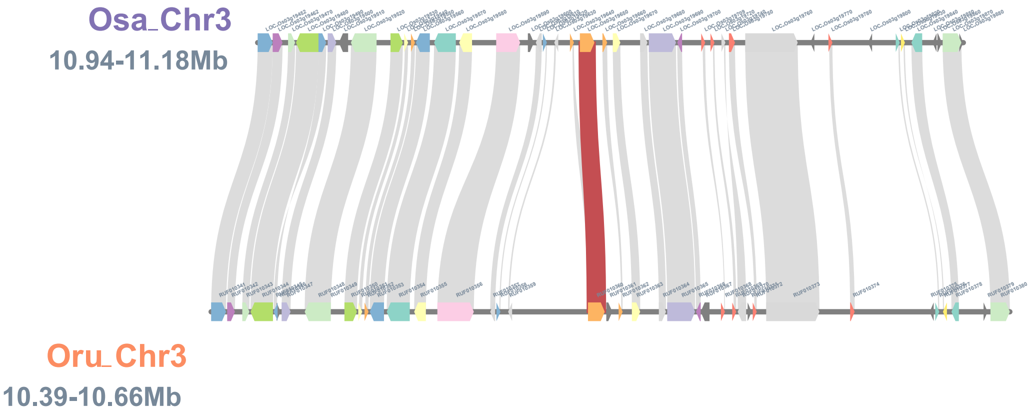

LOC_Os03g19980 RUF010391

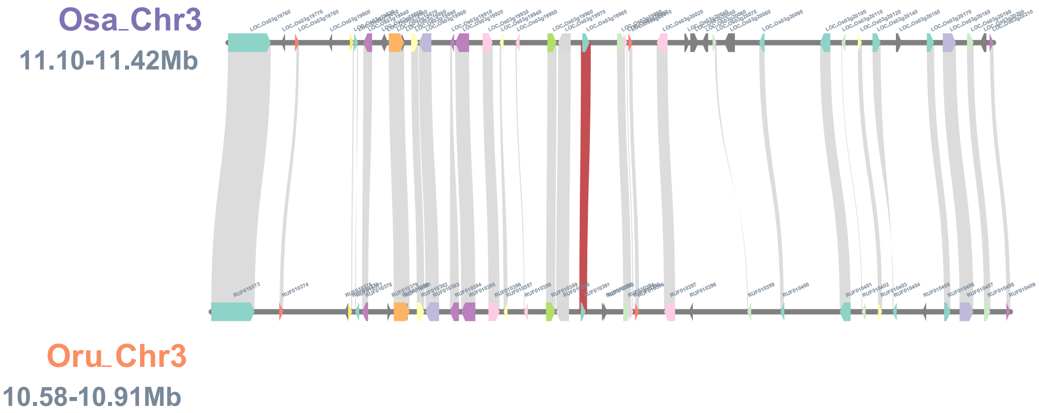

LOC_Os03g20190 RUF010408

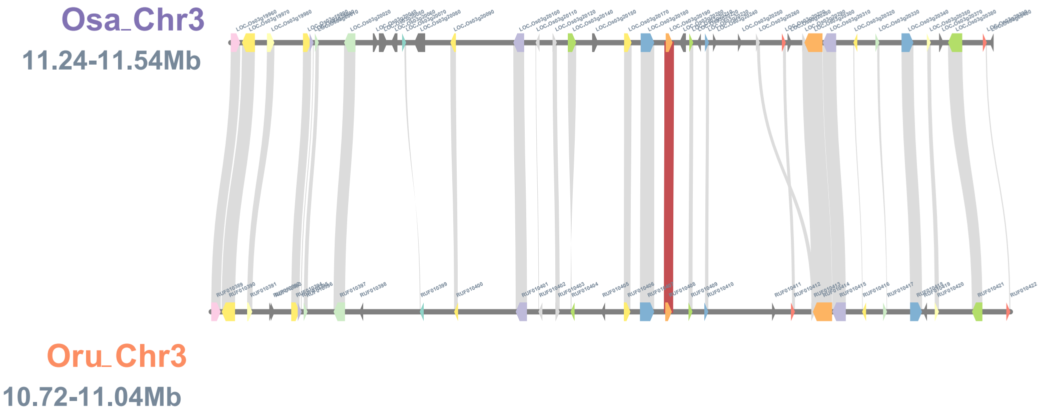

LOC_Os03g21780 RUF010545

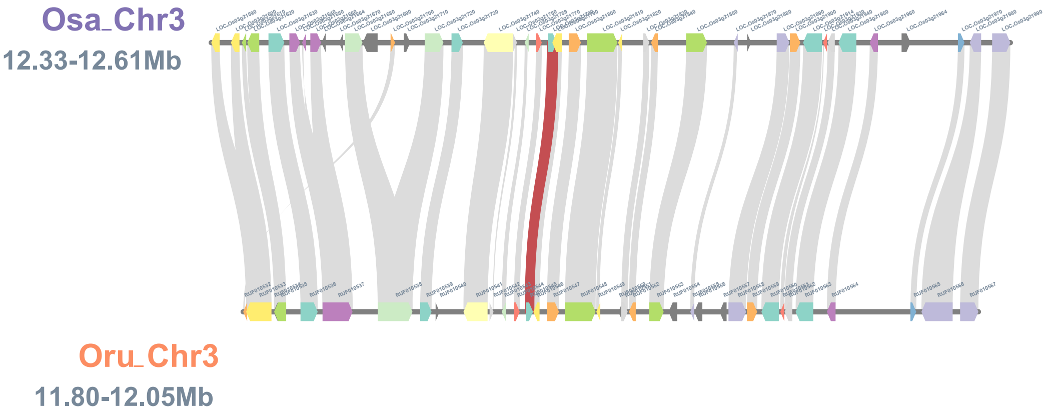

LOC_Os03g24880 RUF010720

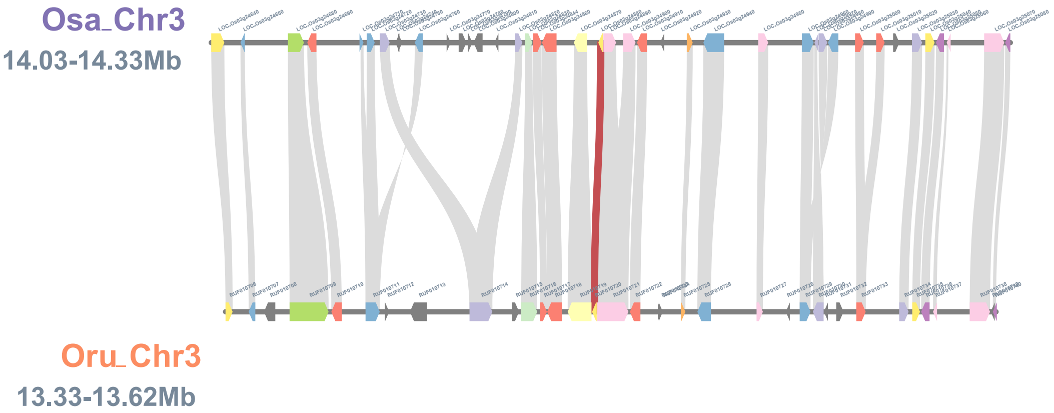

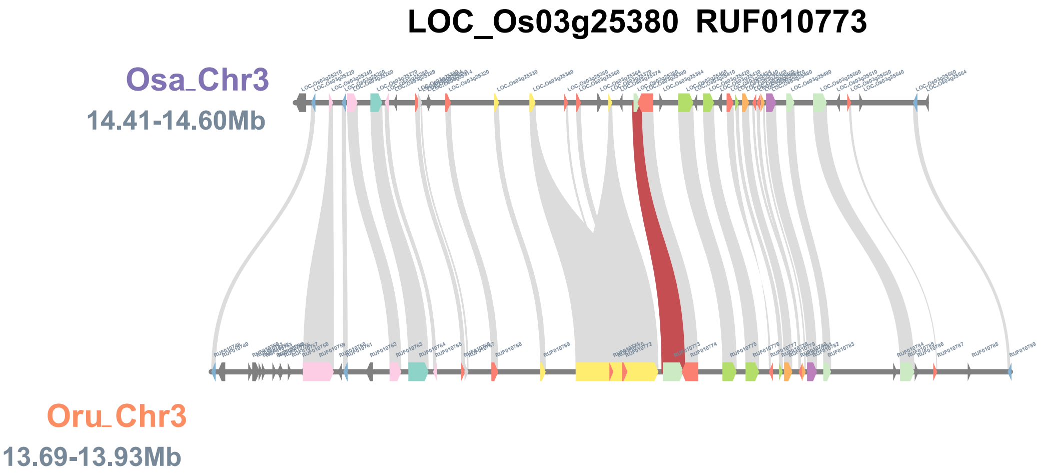
LOC_Os03g25380 RUF010773
Osa_Chr3
14.41-14.60Mb
Oru_Chr3
13.69-13.93Mb

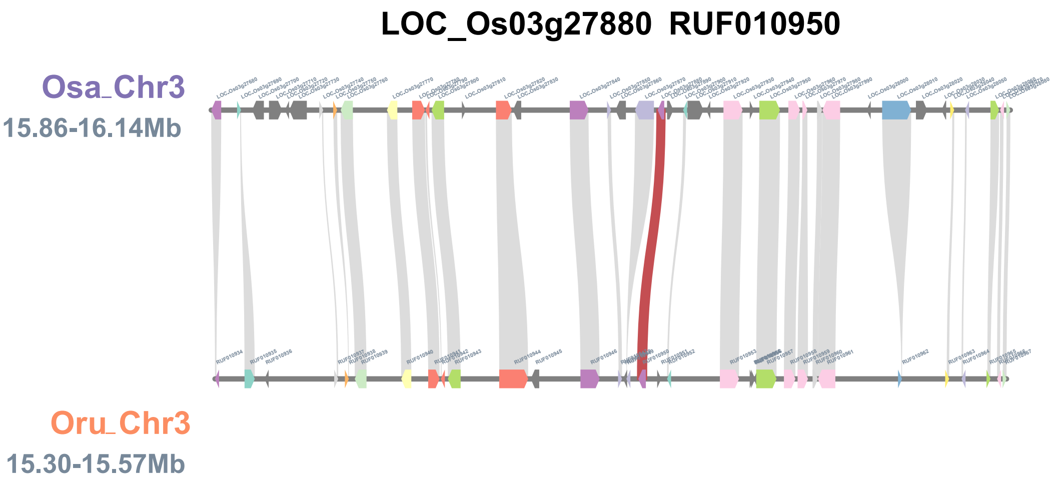
LOC_Os03g27880 RUF010950
Osa_Chr3
15.86-16.14Mb
Oru_Chr3
15.30-15.57Mb

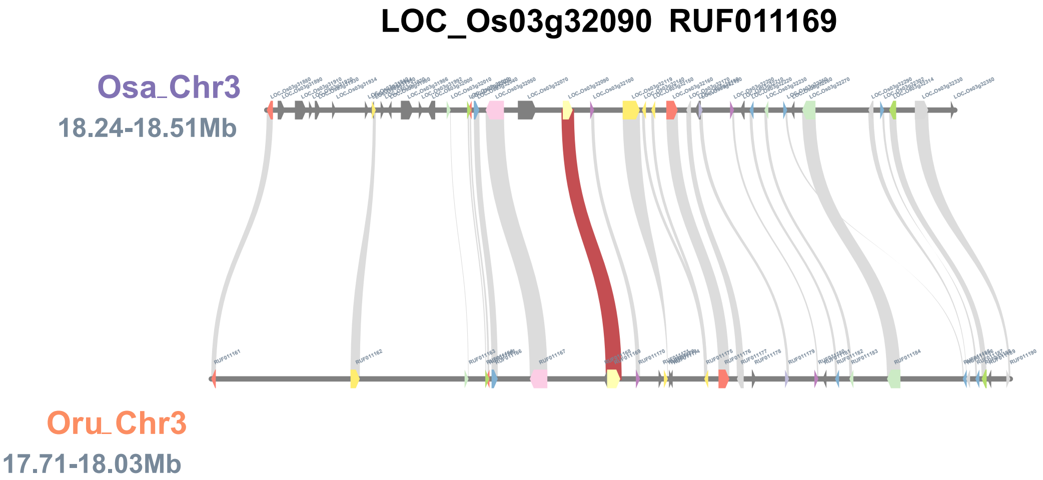
LOC_Os03g32090 RUF011169
Osa_Chr3
18.24-18.51Mb
Oru_Chr3
17.71-18.03Mb

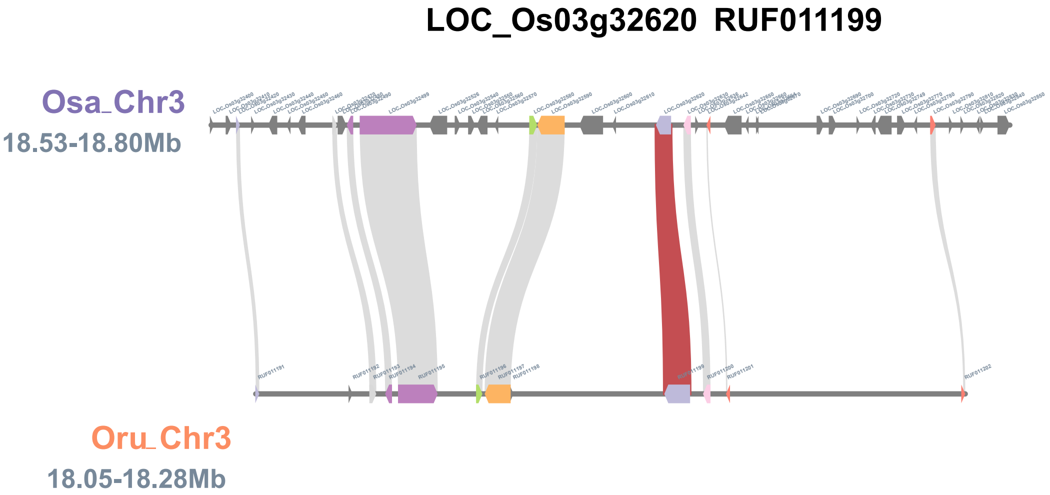
LOC_Os03g32620 RUF011199
Osa_Chr3
18.53-18.80Mb
Oru_Chr3
18.05-18.28Mb

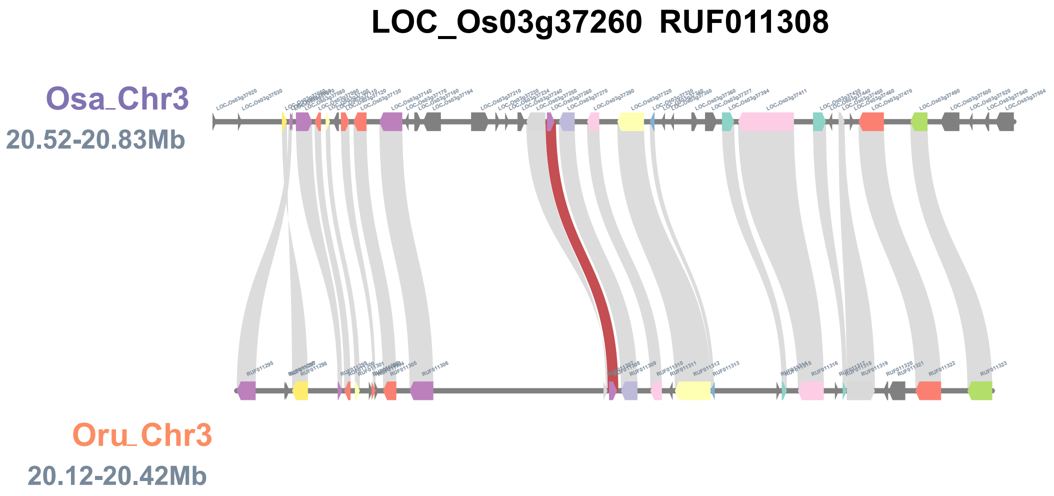
LOC_Os03g37260 RUF011308
Osa_Chr3
20.52-20.83Mb
Oru_Chr3
20.12-20.42Mb

LOC_Os03g38580 RUF011373

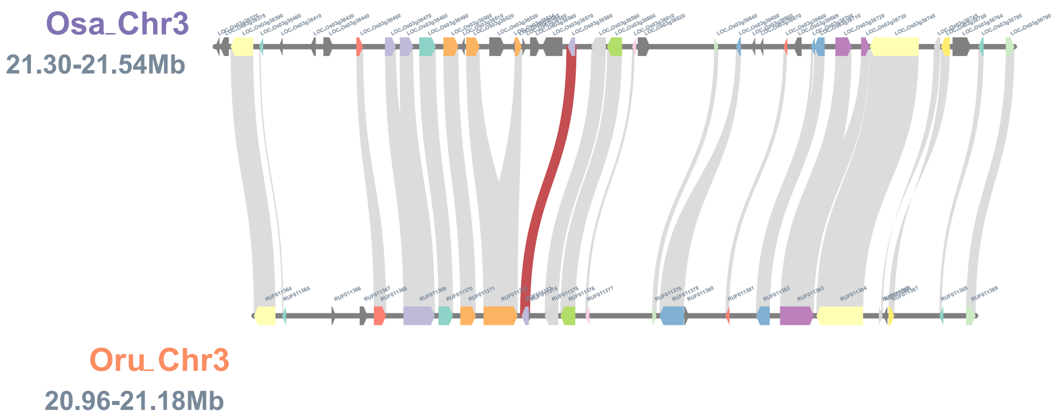

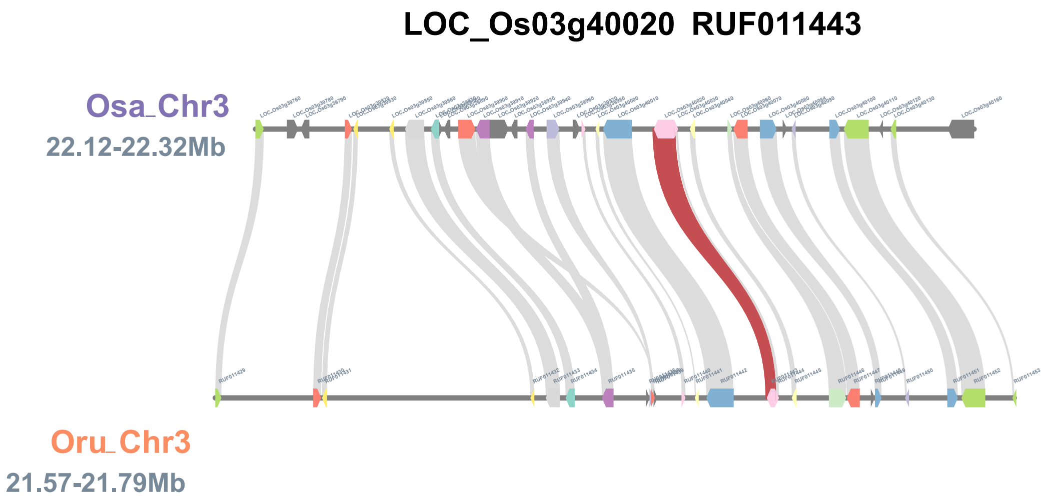
LOC_Os03g40020 RUF011443
Osa_Chr3
22.12-22.32Mb
Oru_Chr3
21.57-21.79Mb

LOC_Os03g40690 RUF011502

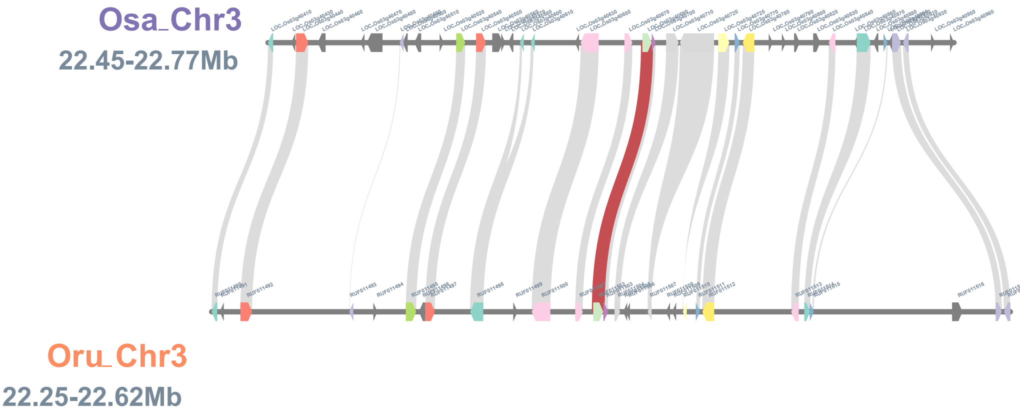

LOC_Os03g42650 RUF011588

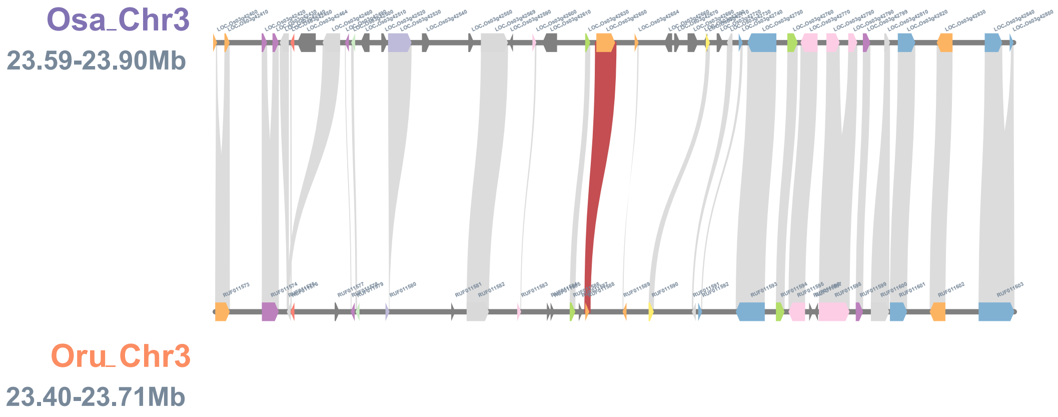

LOC_Os03g43470 RUF011636

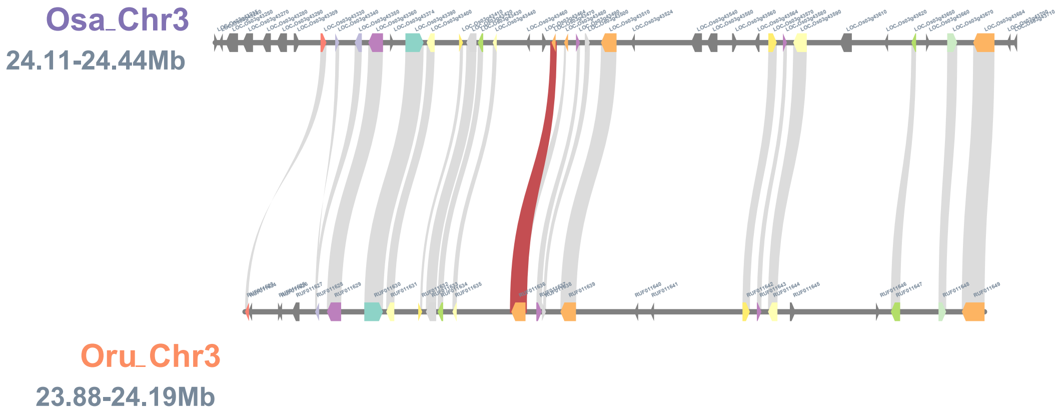

LOC_Os03g44210 RUF011690

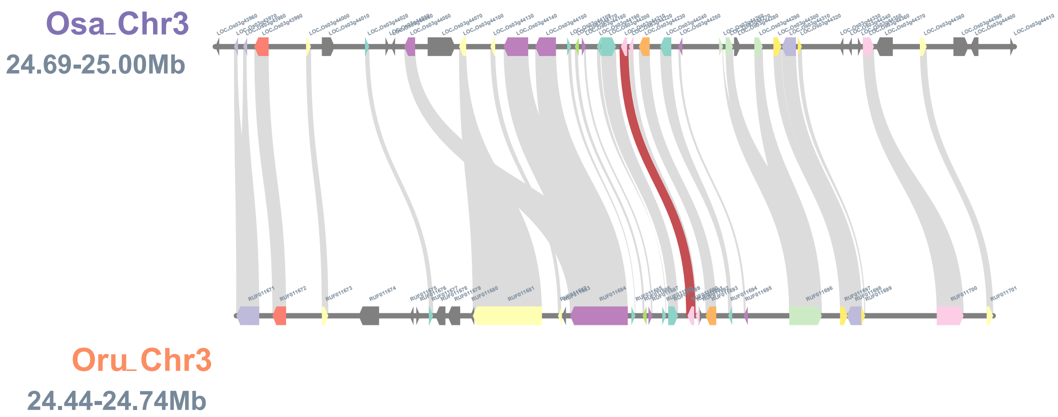

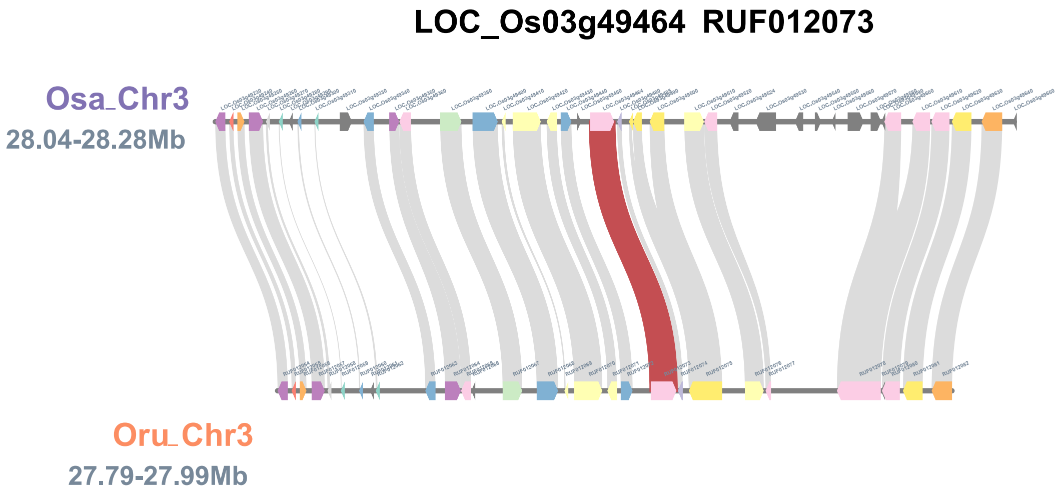
LOC_Os03g49464 RUF012073
Osa_Chr3
28.04-28.28Mb
Oru_Chr3
27.79-27.99Mb

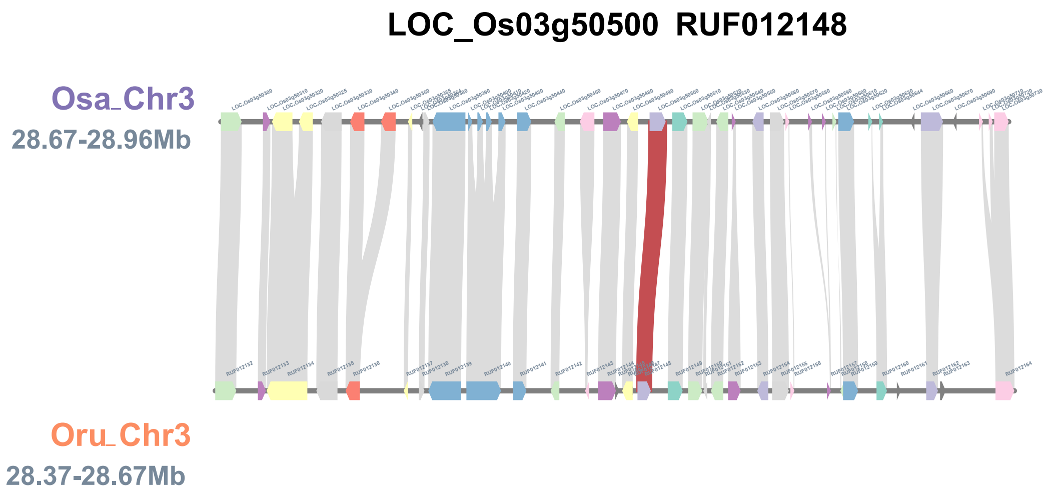
LOC_Os03g50500 RUF012148
Osa_Chr3
28.67-28.96Mb
Oru_Chr3
28.37-28.67Mb

LOC_Os03g51840 RUF012261

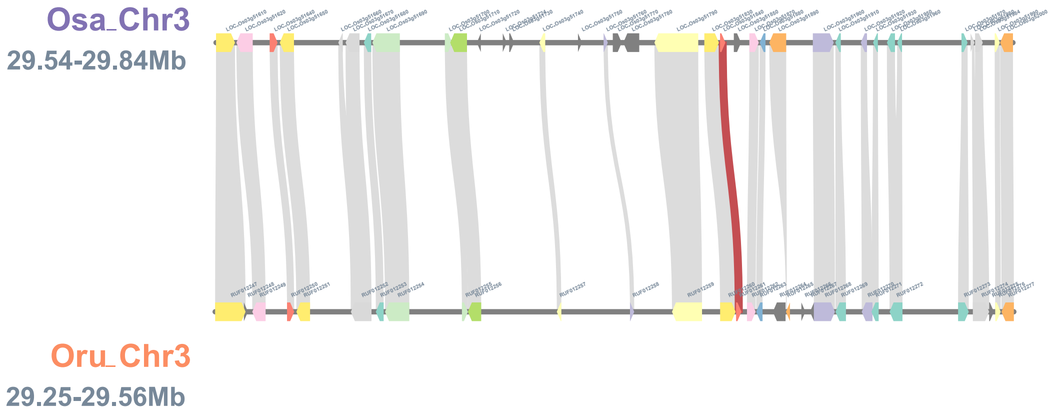

LOC_Os03g53170 RUF012300

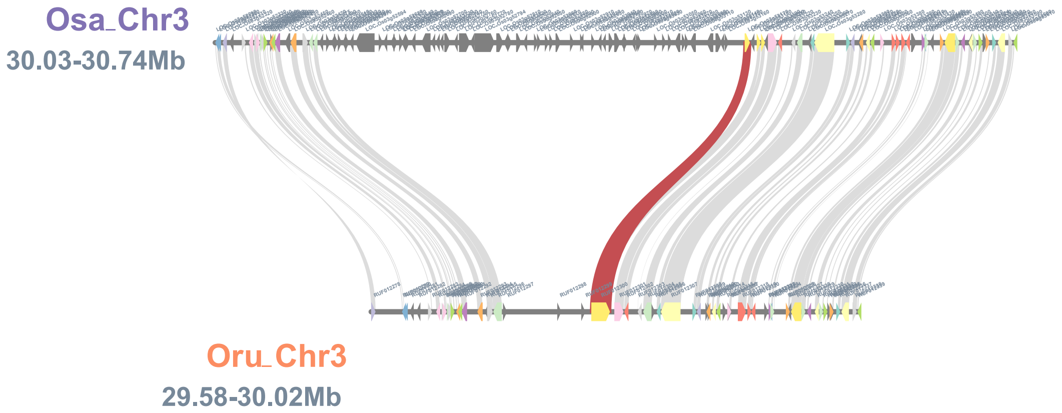

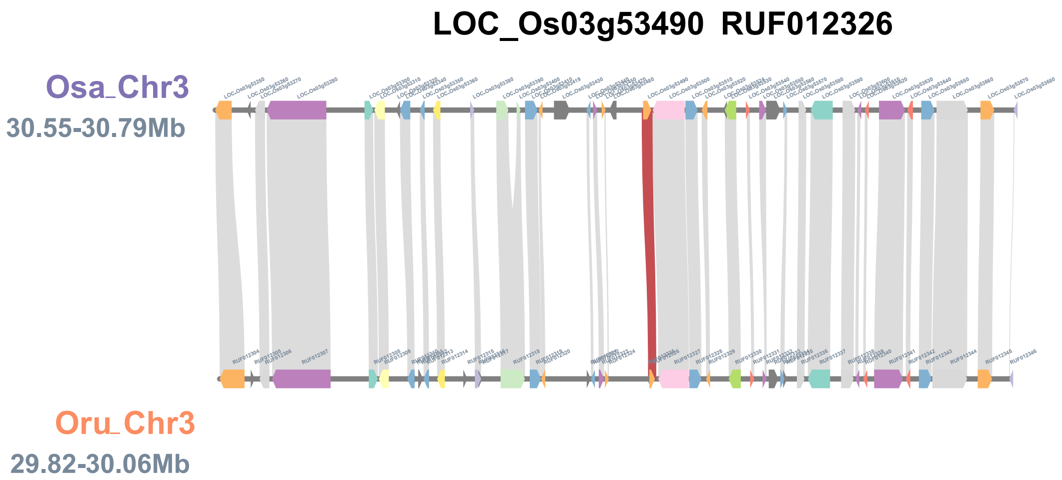
LOC_Os03g53490 RUF012326
Osa_Chr3
30.55-30.79Mb
Oru_Chr3
29.82-30.06Mb

LOC_Os03g55340 RUF012437

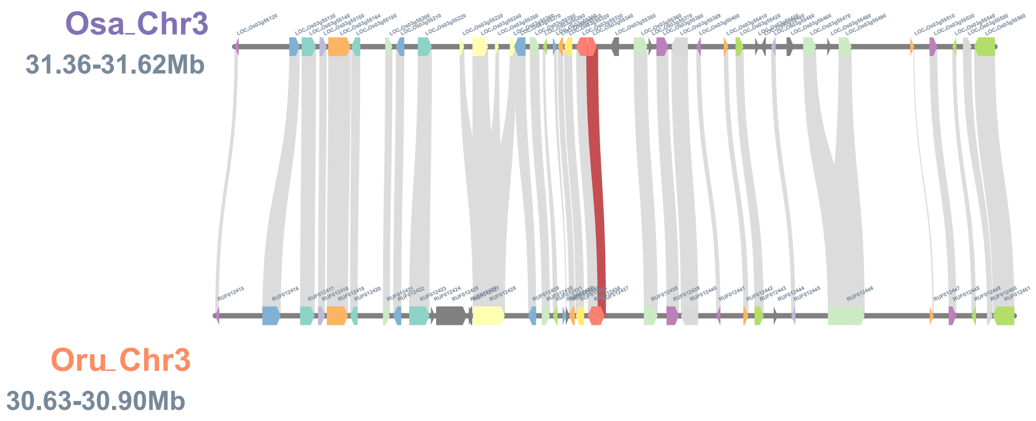

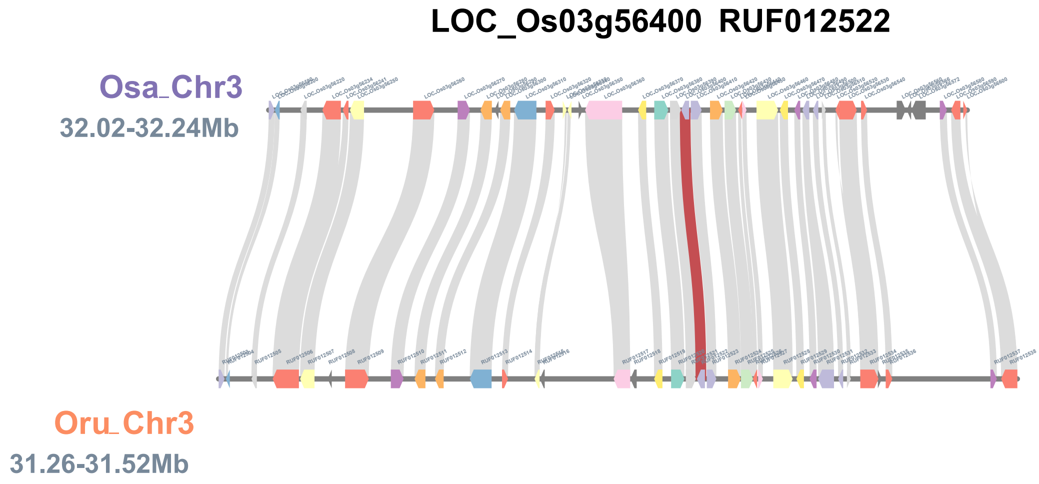
LOC_Os03g56400 RUF012522
Osa_Chr3
32.02-32.24Mb
Oru_Chr3
31.26-31.52Mb

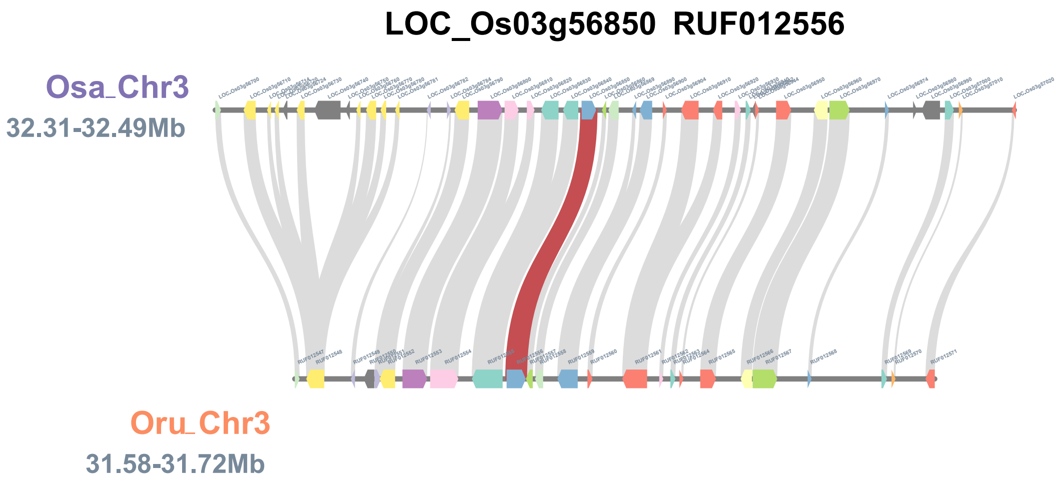
LOC_Os03g56850 RUF012556
Osa_Chr3
32.31-32.49Mb
Oru_Chr3
31.58-31.72Mb

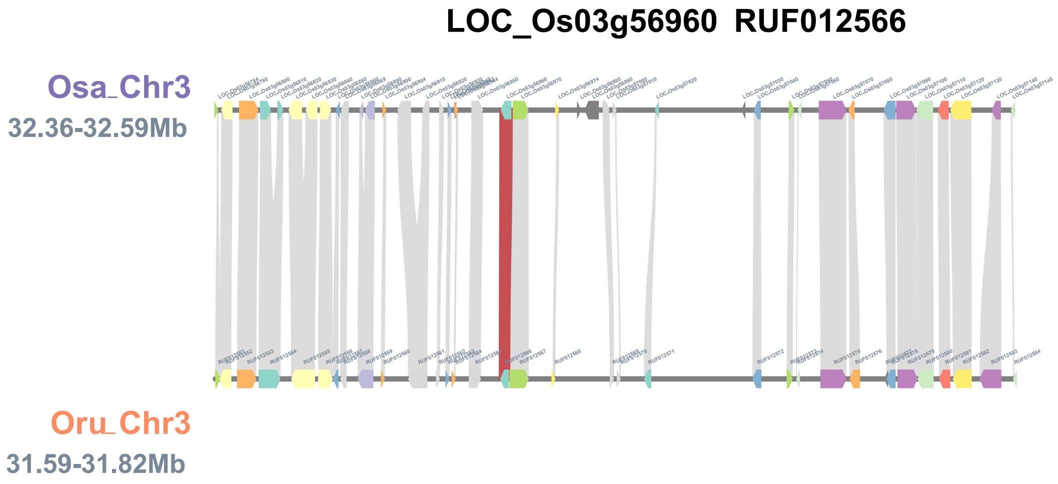
LOC_Os03g56960 RUF012566
Osa_Chr3
32.36-32.59Mb
Oru_Chr3
31.59-31.82Mb

LOC_Os03g58100 RUF012654

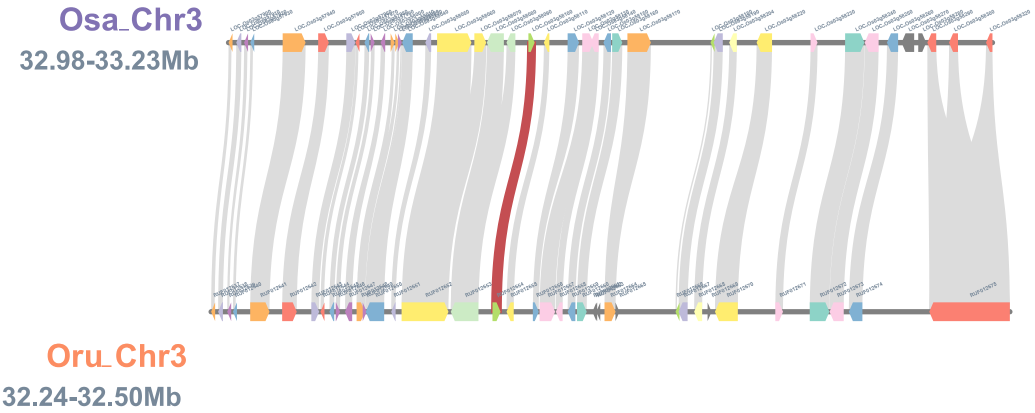

LOC_Os03g58120 RUF012656

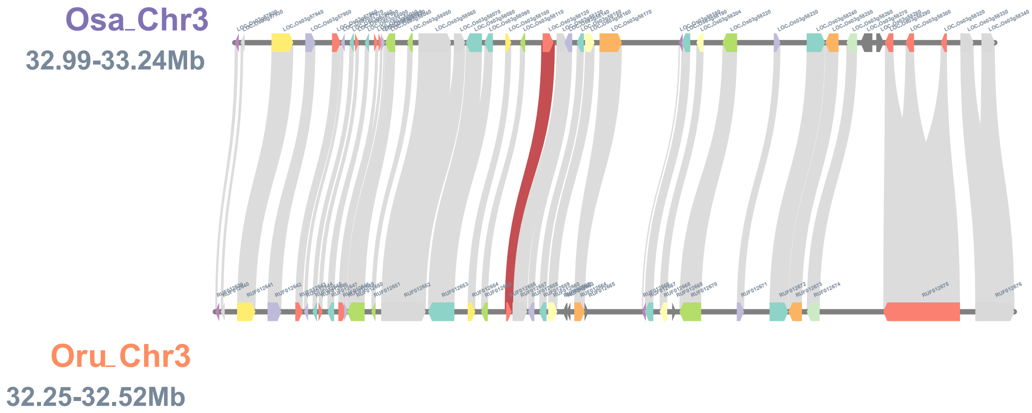

LOC_Os03g59264 RUF012753

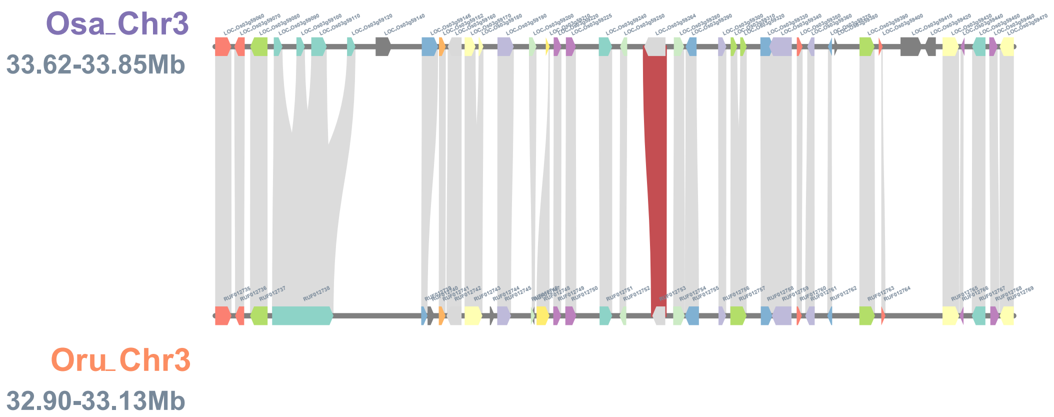

LOC_Os03g60200 RUF012817

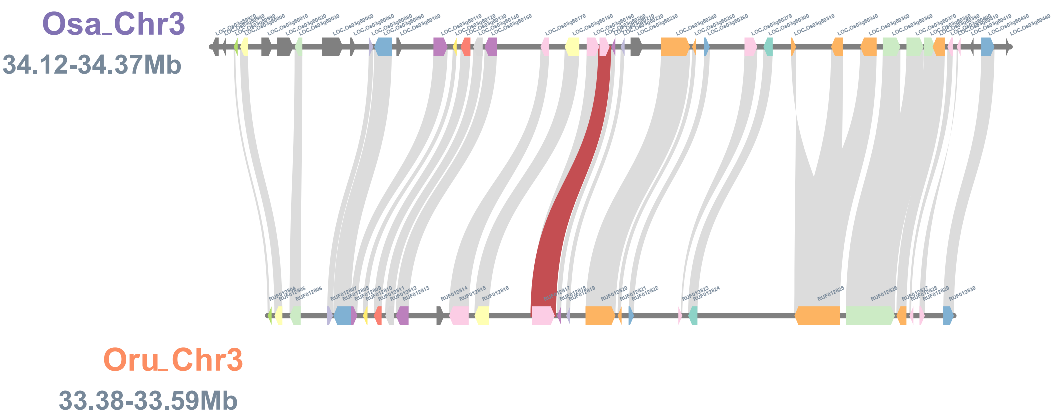

LOC_Os03g60690 RUF012851

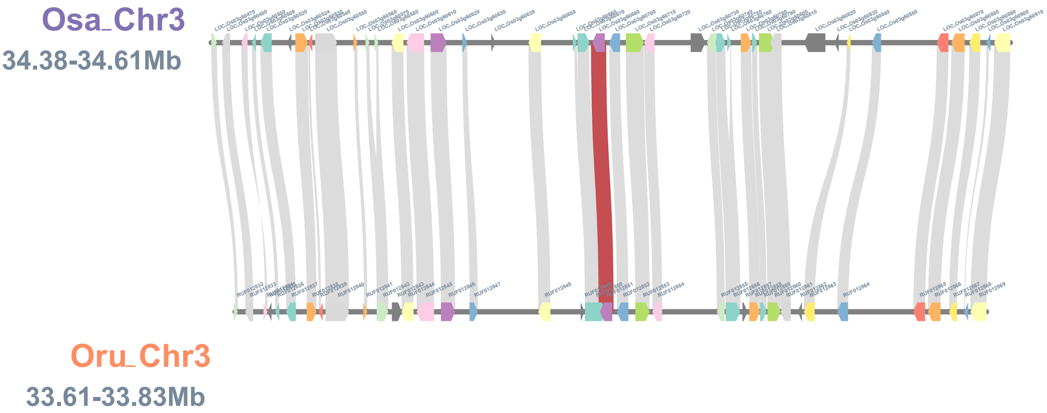

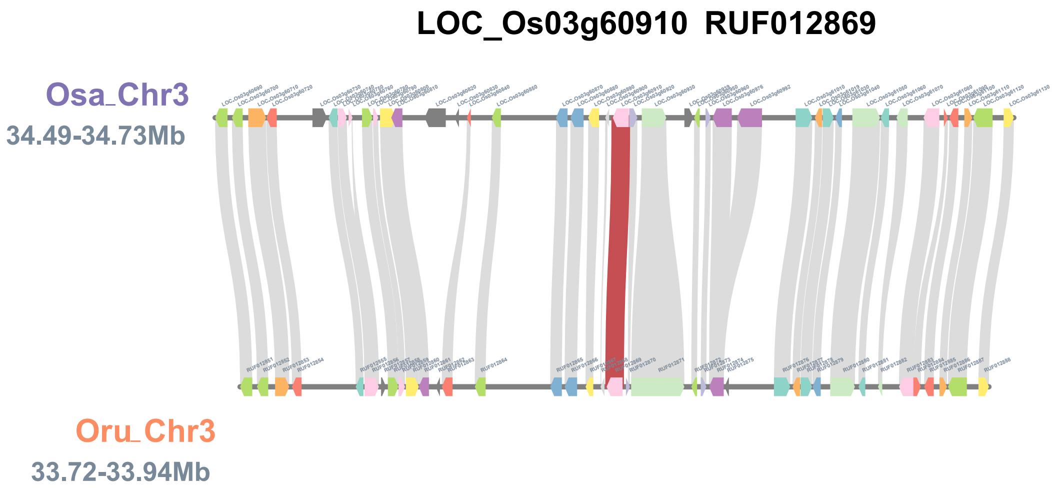
LOC_Os03g60910 RUF012869
Osa_Chr3
34.49-34.73Mb
Oru_Chr3
33.72-33.94Mb

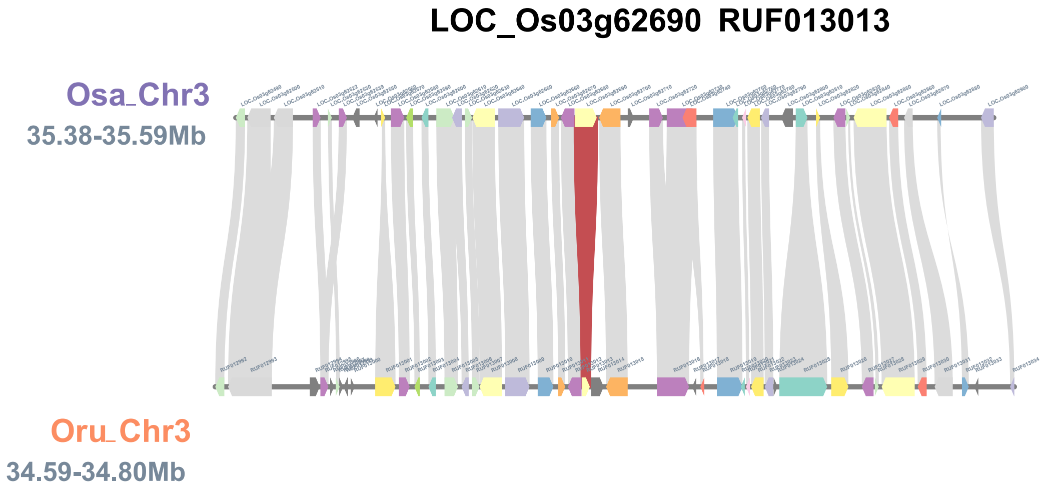
LOC_Os03g62690 RUF013013
Osa_Chr3
35.38-35.59Mb
Oru_Chr3
34.59-34.80Mb

LOC_Os03g63260 RUF013053

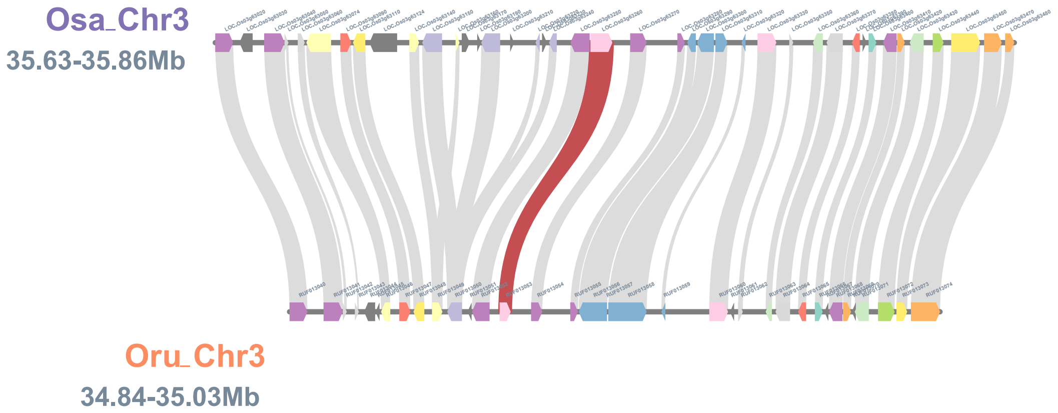

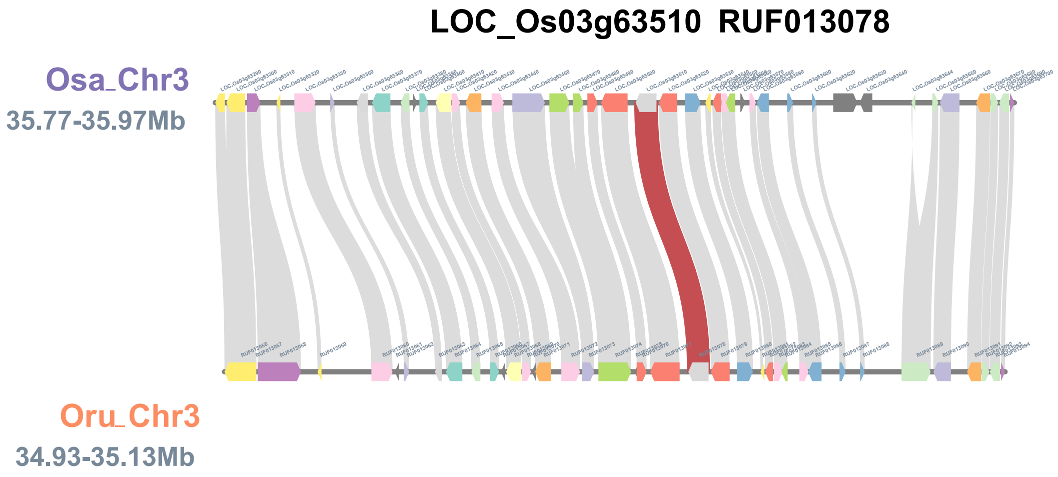
LOC_Os03g63510 RUF013078
Osa_Chr3
35.77-35.97Mb
Oru_Chr3
34.93-35.13Mb

LOC_Os03g63560 RUF013084

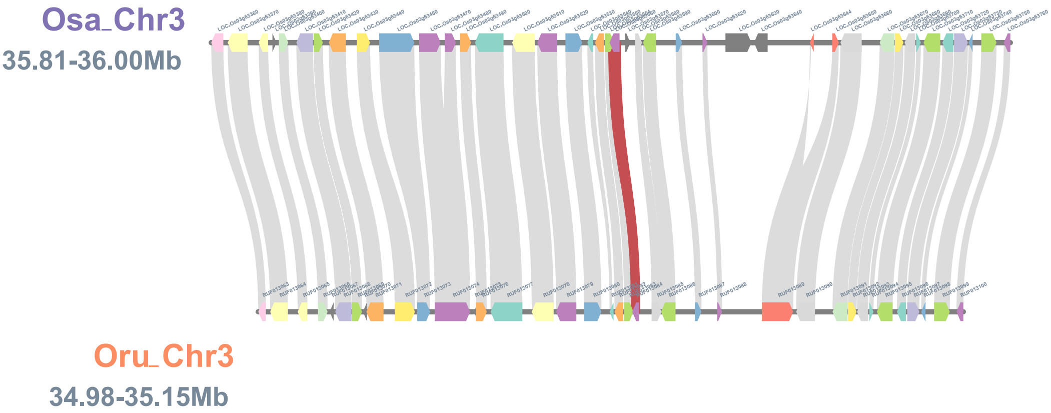

LOC_Os03g63910 RUF013110

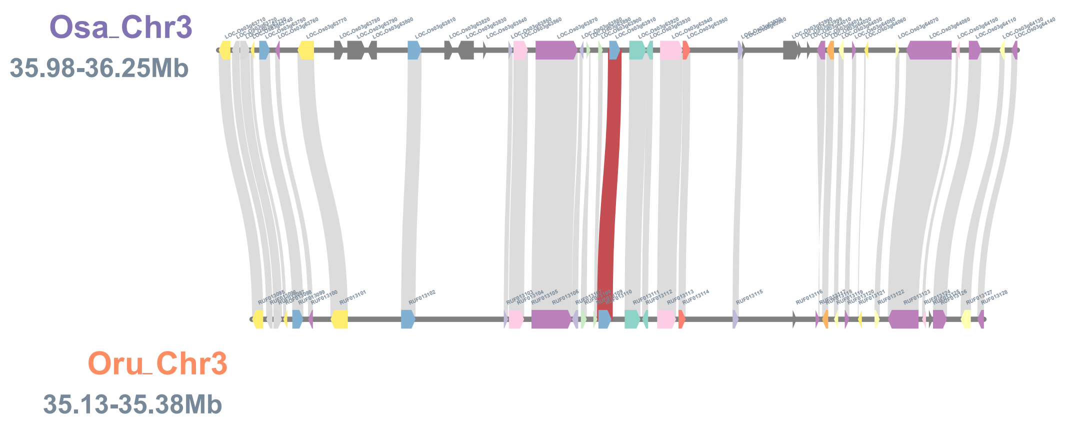

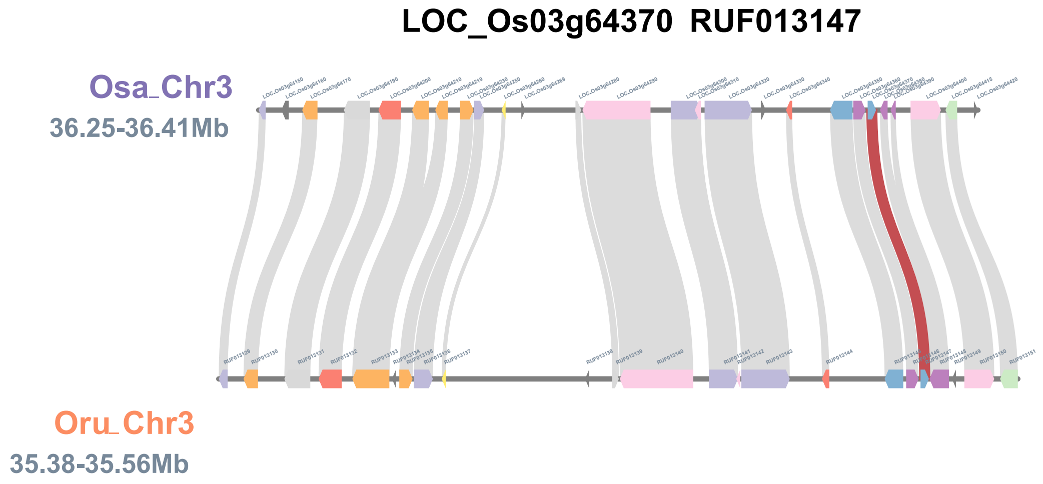
LOC_Os03g64370 RUF013147
Osa_Chr3
36.25-36.41Mb
Oru_Chr3
35.38-35.56Mb

LOC_Os04g02850 RUF013409

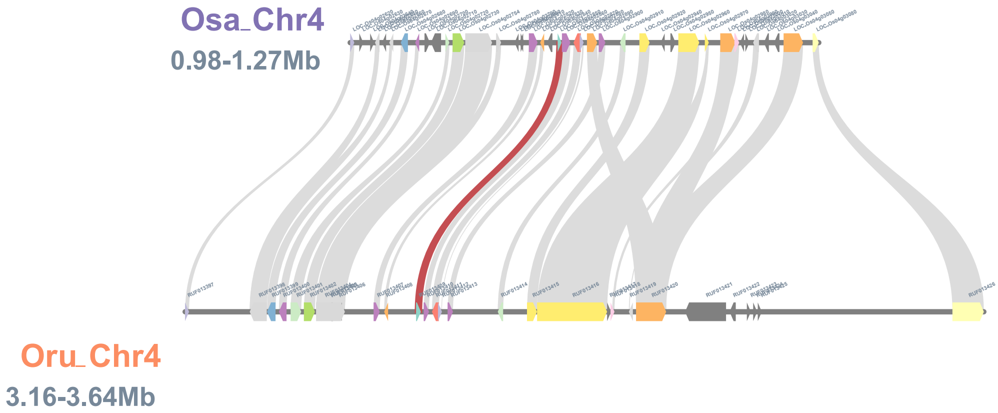

LOC_Os04g25410 RUF014104

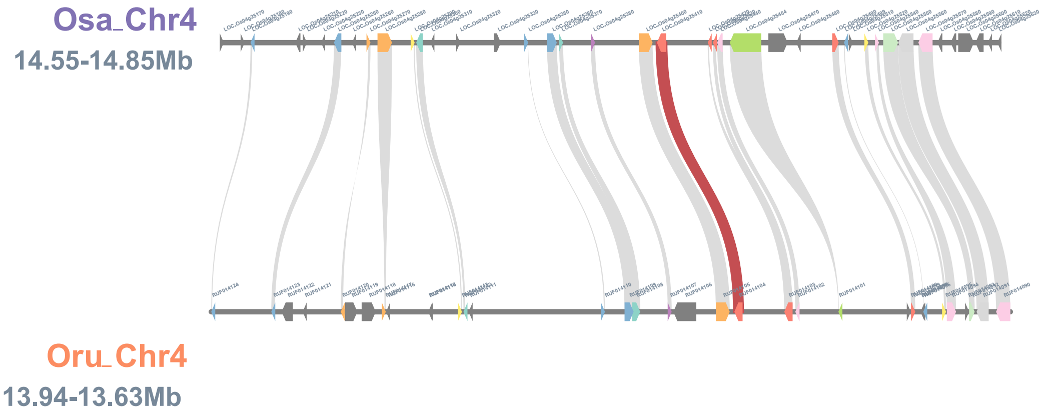

LOC_Os04g28234 RUF014227

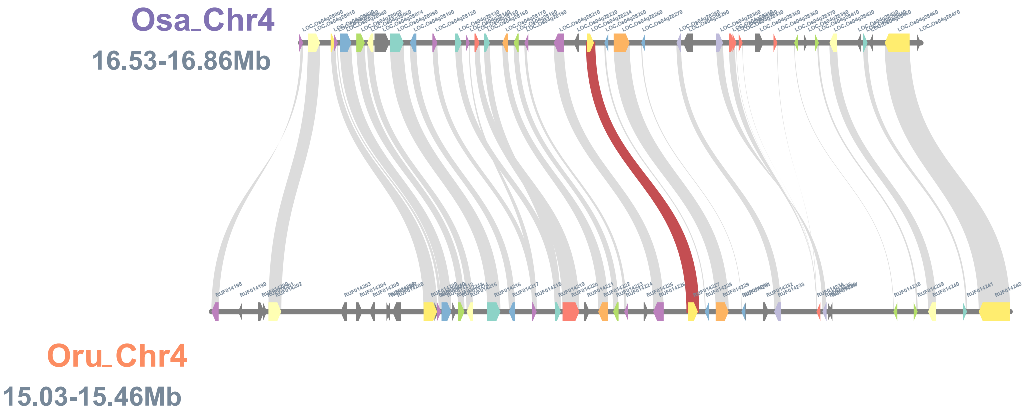

LOC_Os04g28300 RUF014235

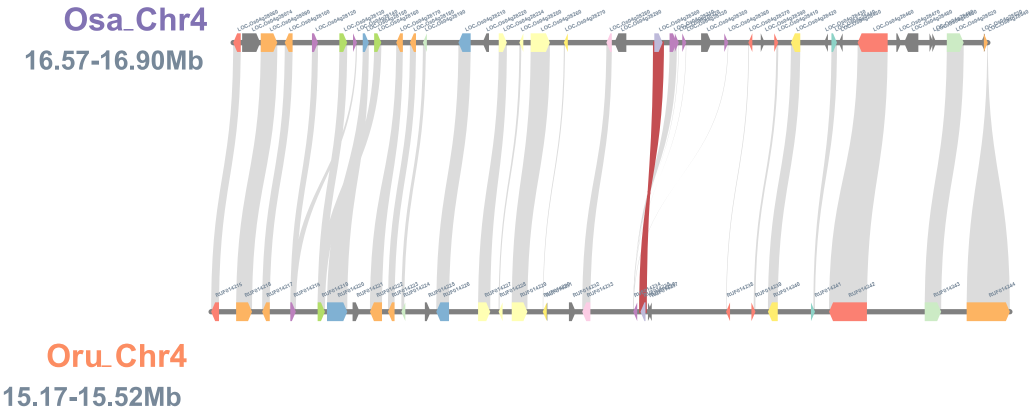

LOC_Os04g32870 RUF014521

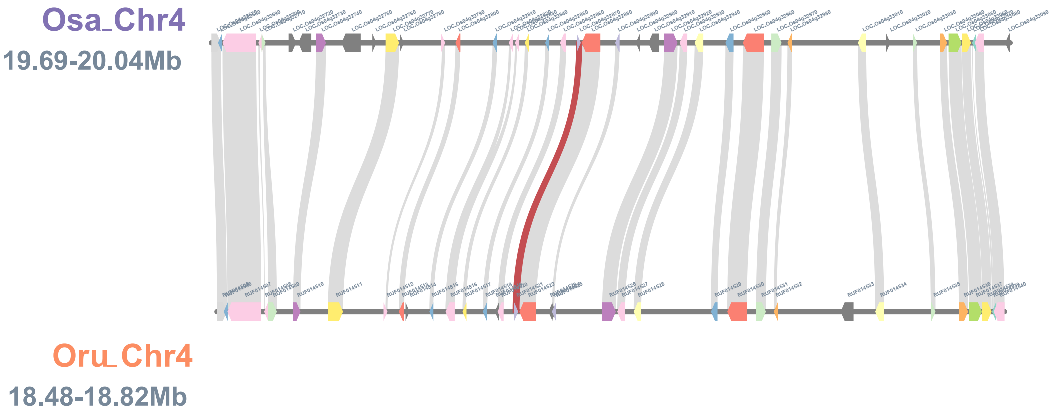

LOC_Os04g33840 RUF014614

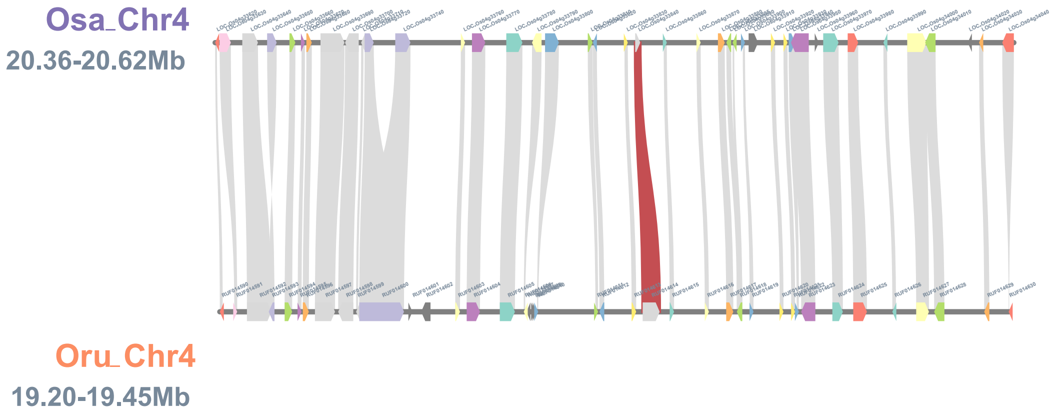

LOC_Os04g35305 RUF014711

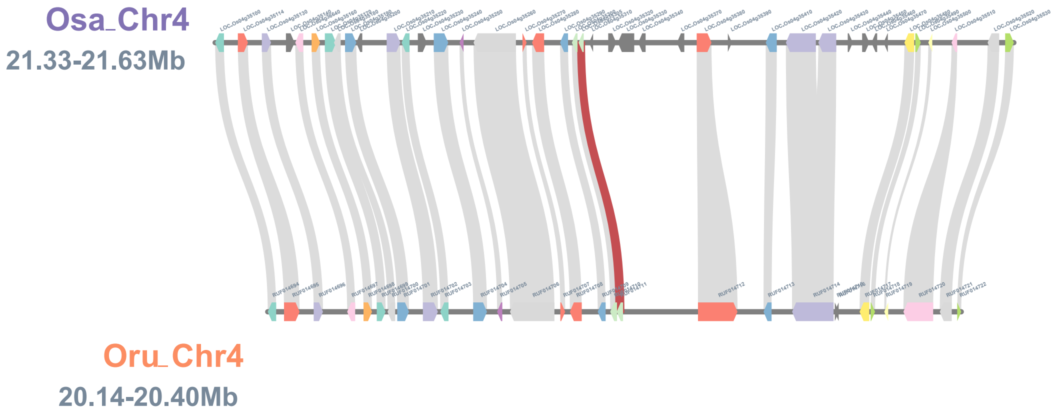

LOC_Os04g35610 RUF014732

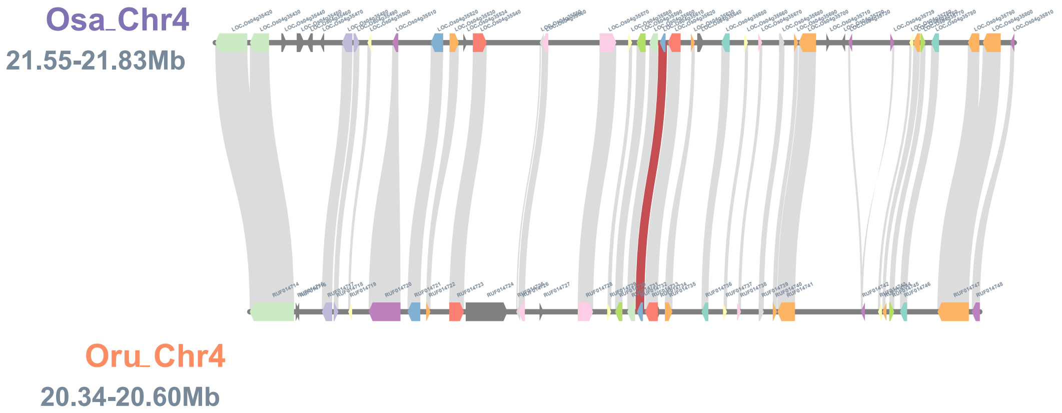

LOC_Os04g35650 RUF014736

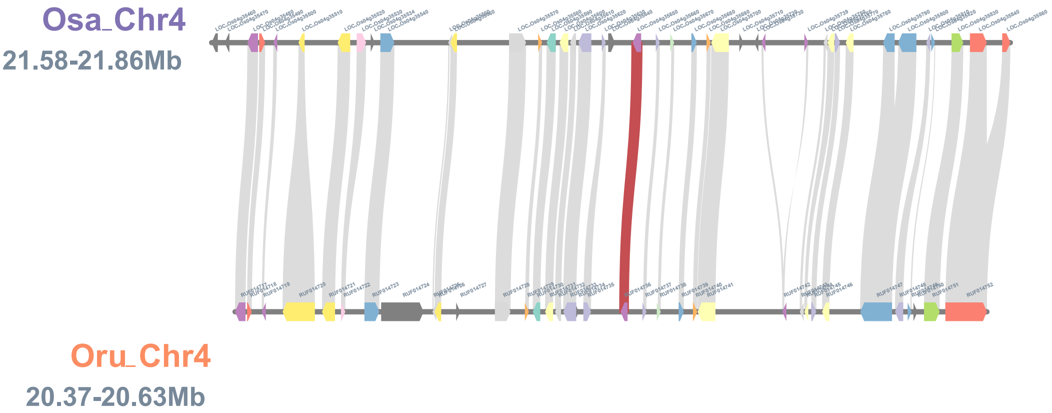

LOC_Os04g36840 RUF014810

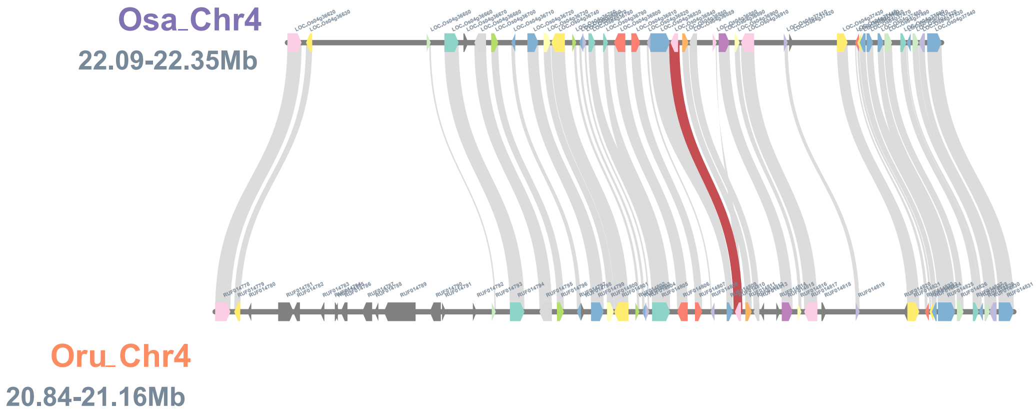

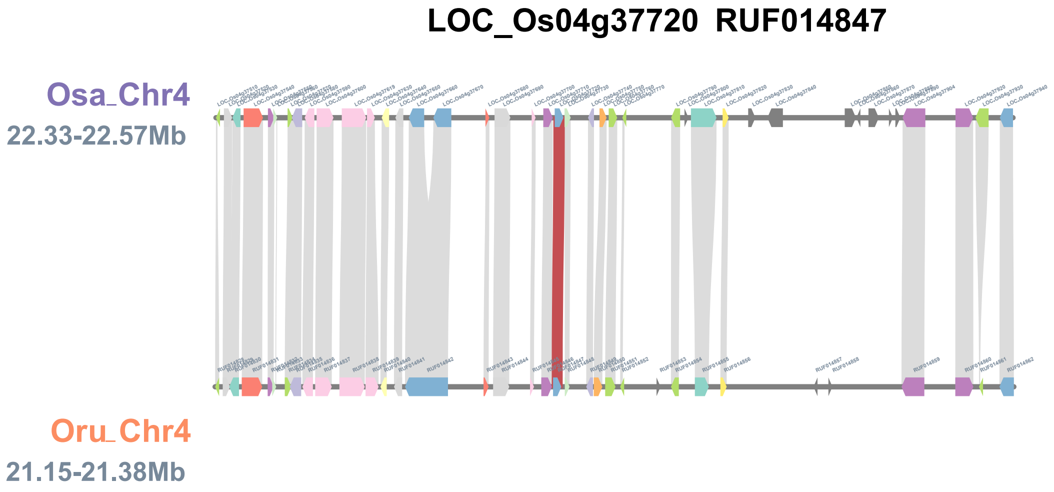
LOC_Os04g37720 RUF014847
Osa_Chr4
22.33-22.57Mb
Oru_Chr4
21.15-21.38Mb

LOC_Os04g38930 RUF014939

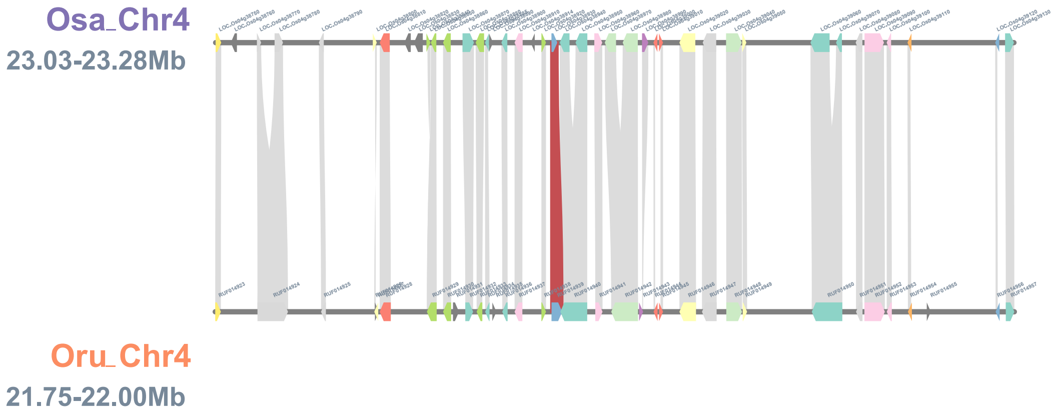

LOC_Os04g38980 RUF014942

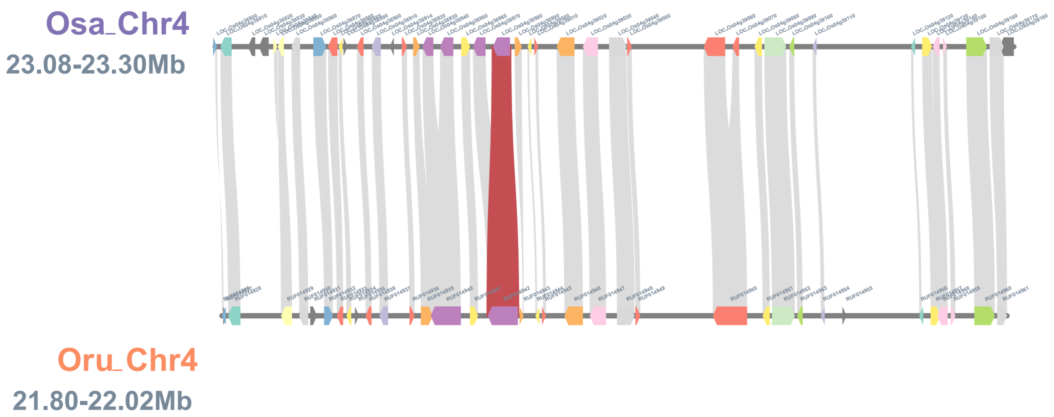

LOC_Os04g39000 RUF014944

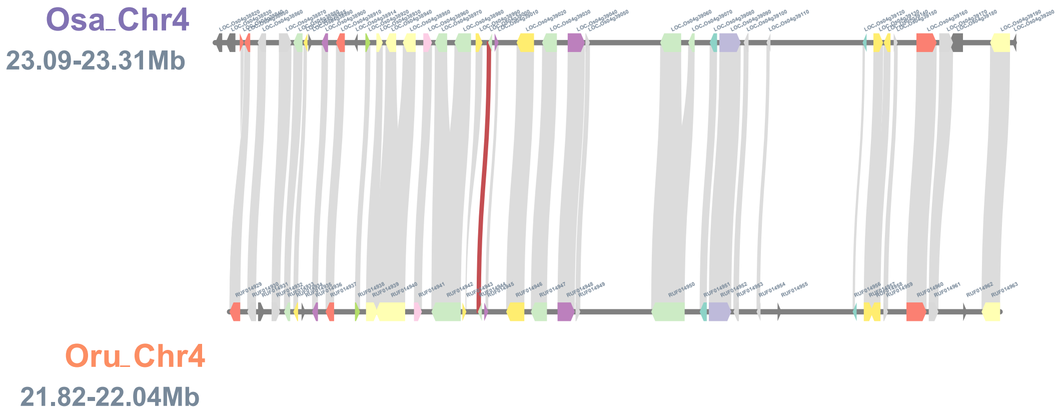

LOC_Os04g39410 RUF014979

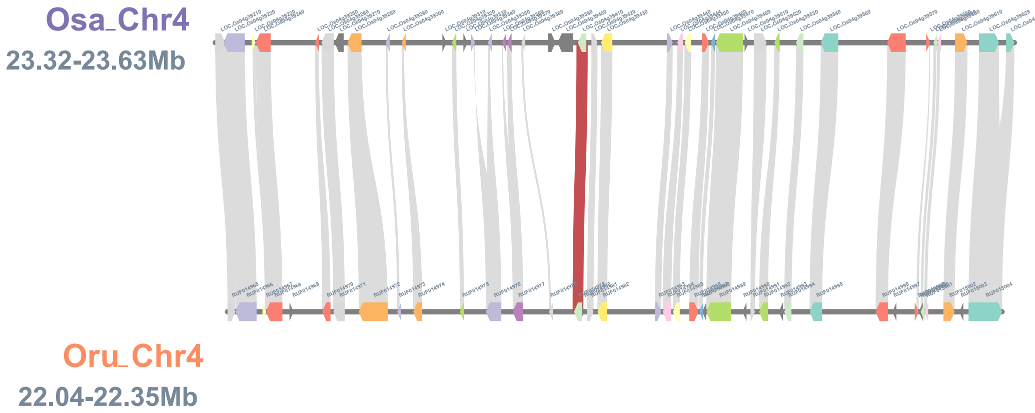

LOC_Os04g40010 RUF015020

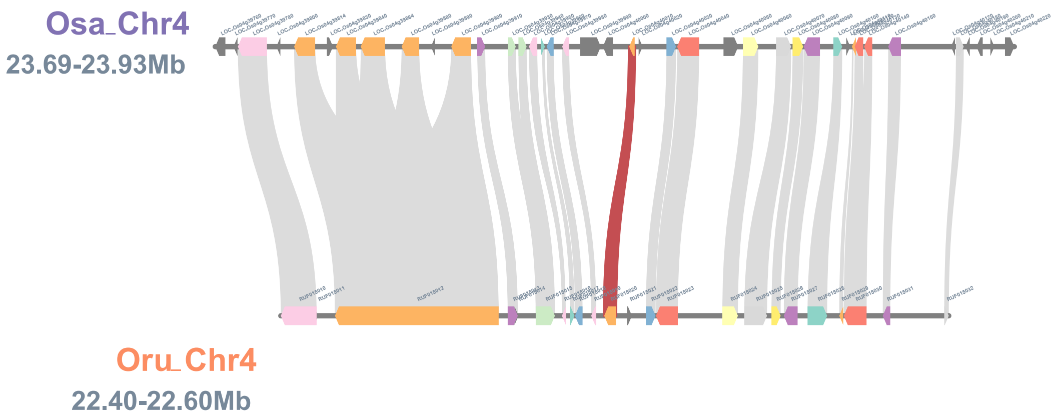

LOC_Os04g43430 RUF015250

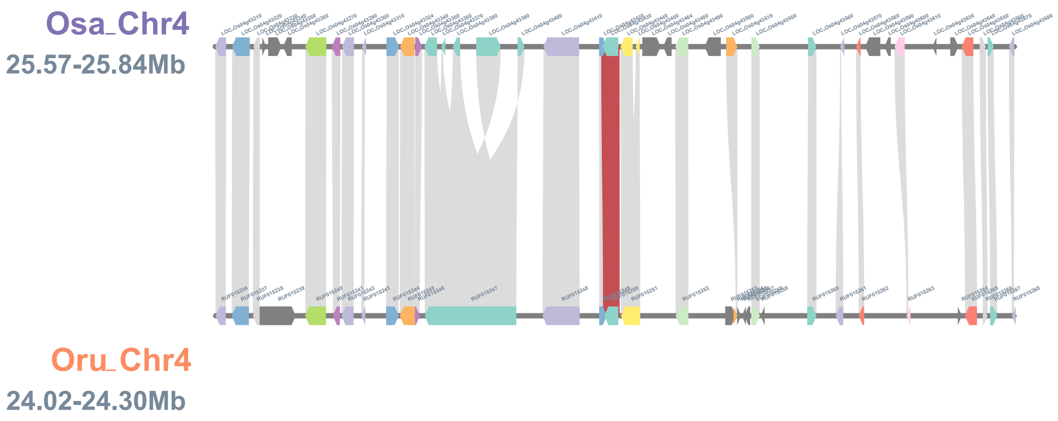

LOC_Os04g44030 RUF015291

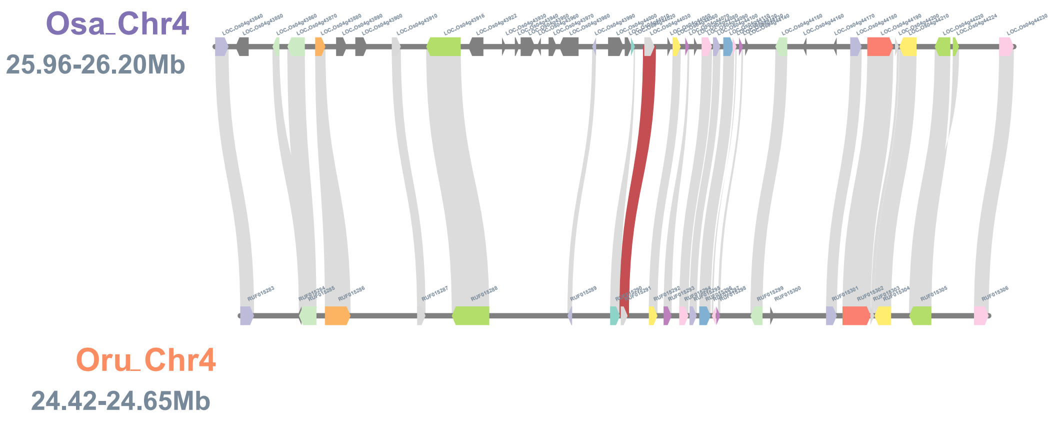

LOC_Os04g46010 RUF015463

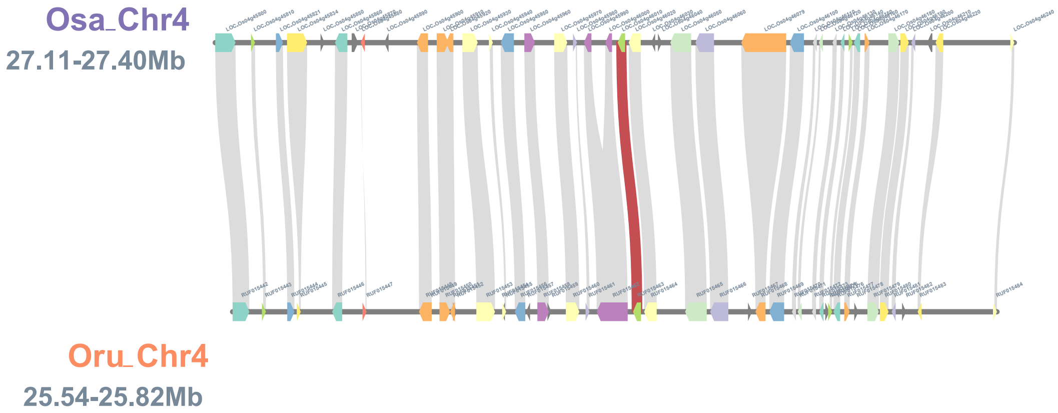

LOC_Os04g49350 RUF015724

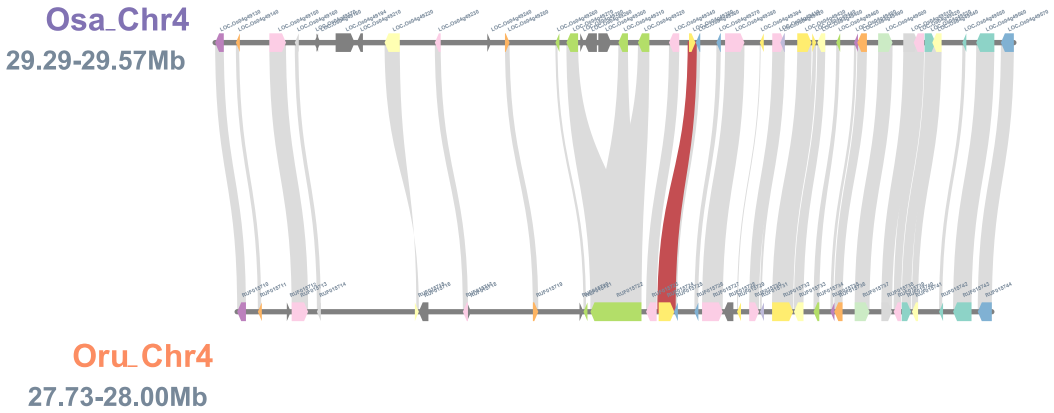

LOC_Os04g49520 RUF015739

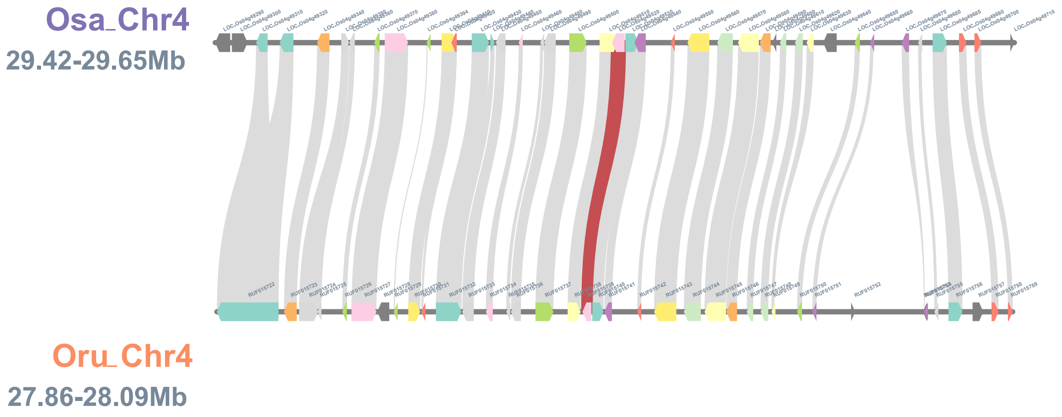

LOC_Os04g51350 RUF015870

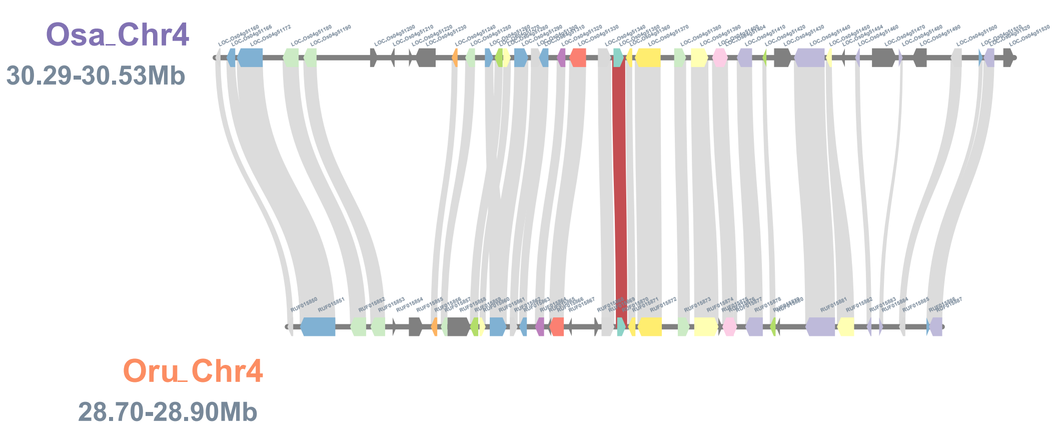

LOC_Os04g52290 RUF015953

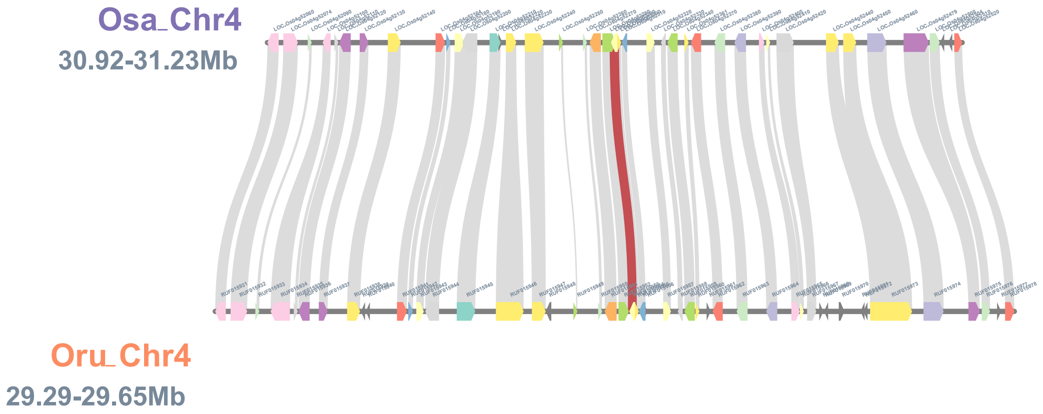

LOC_Os04g52725 RUF015996

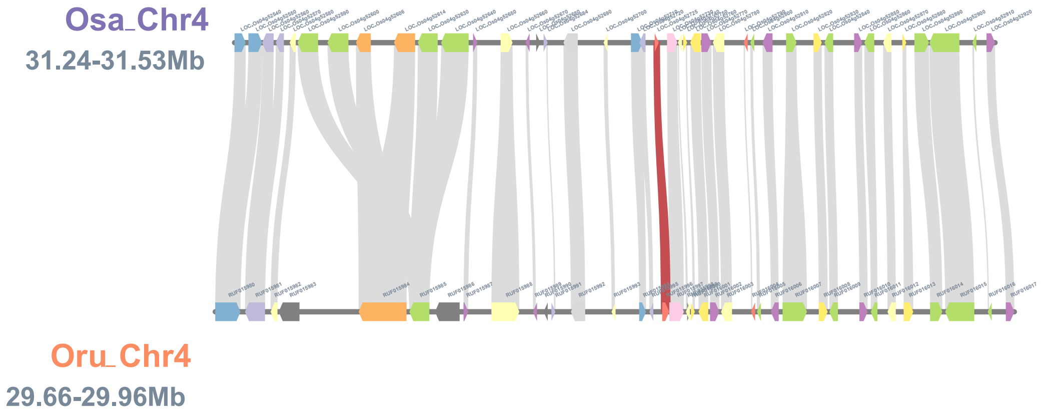

LOC_Os04g53630 RUF016085

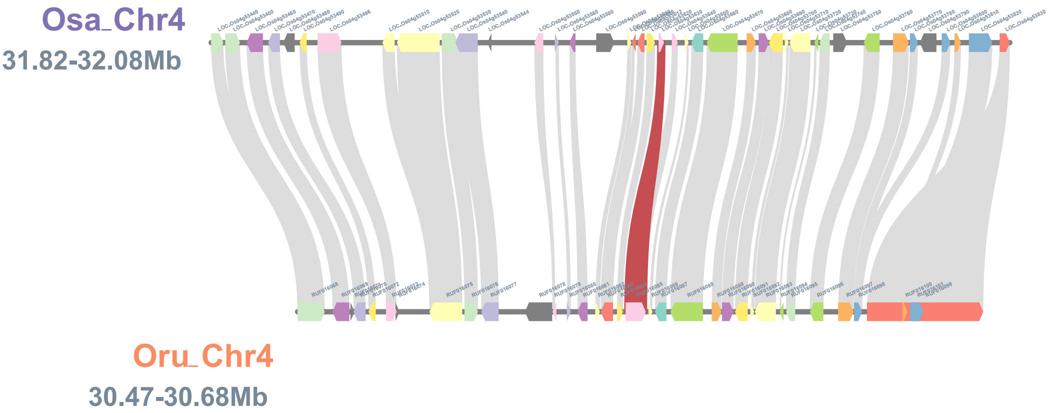

LOC_Os04g55090 RUF016173

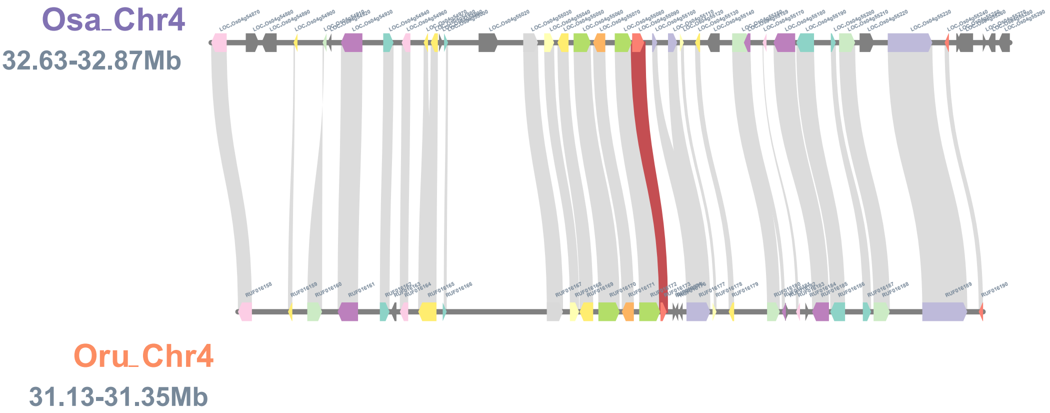

LOC_Os04g57900 RUF016363

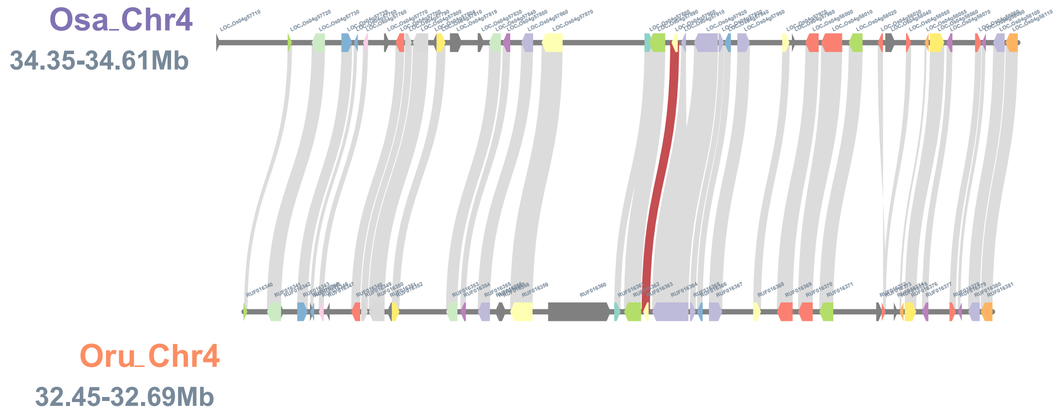

LOC_Os04g57940 RUF016366

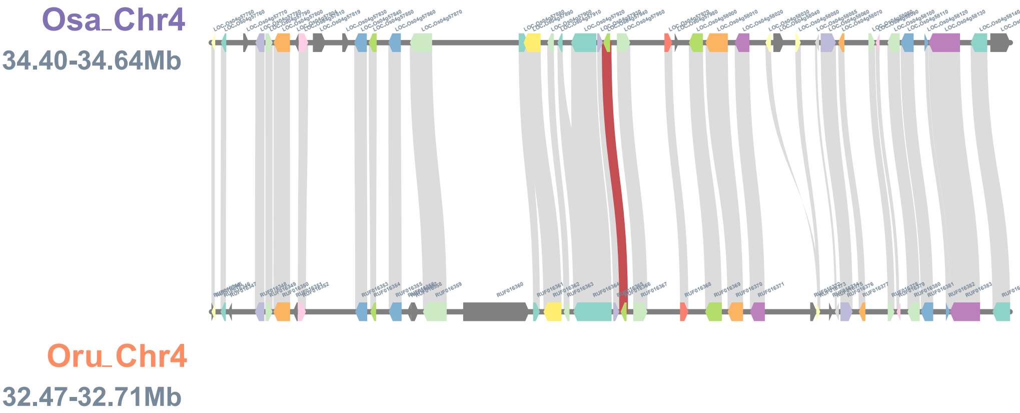

LOC_Os04g58780 RUF016441

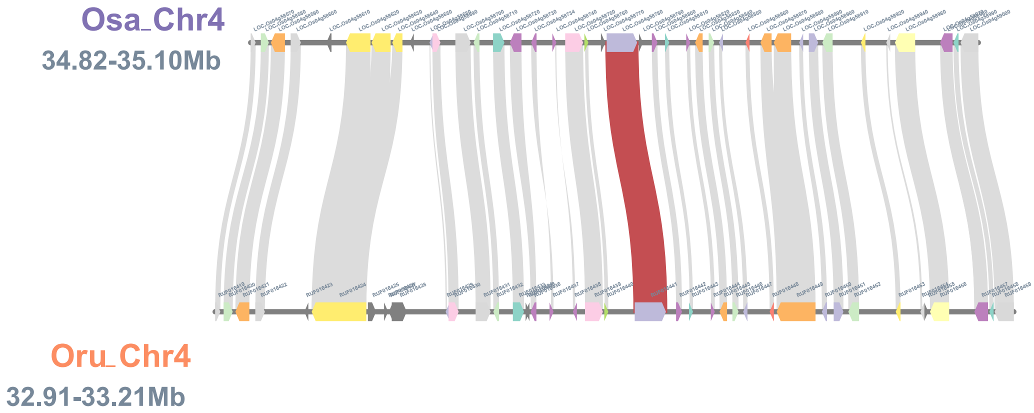

LOC_Os04g58980 RUF016457

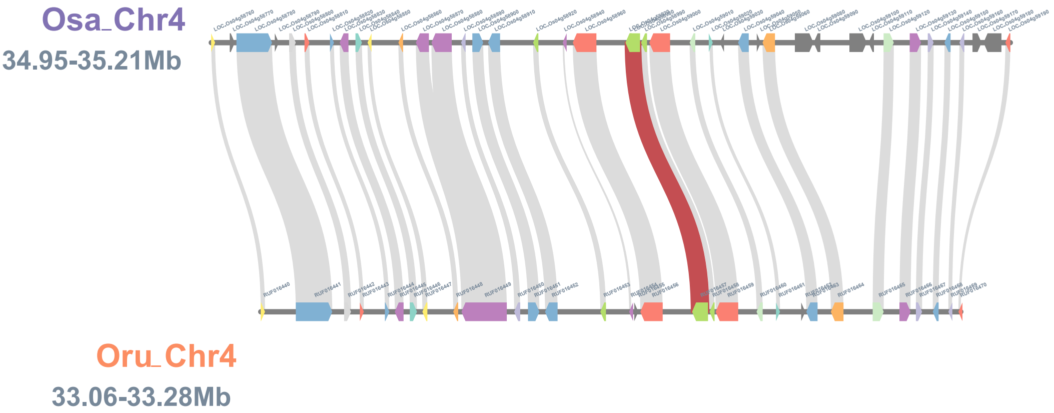

LOC_Os05g01635 RUF016552

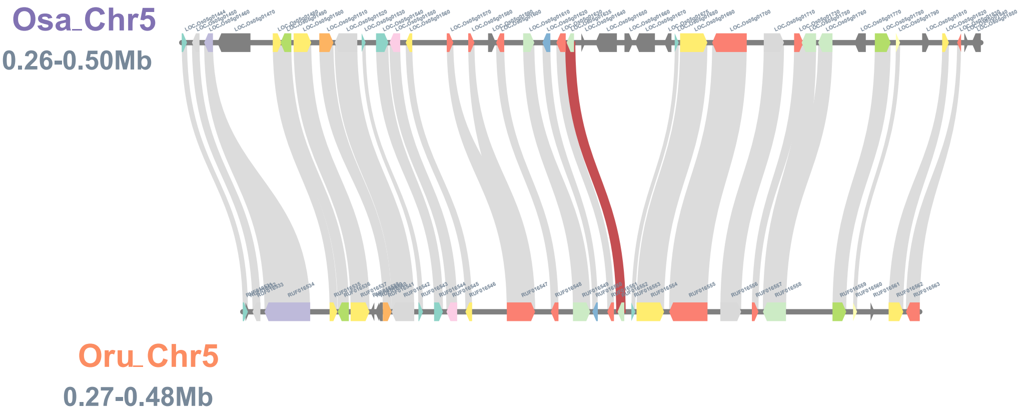

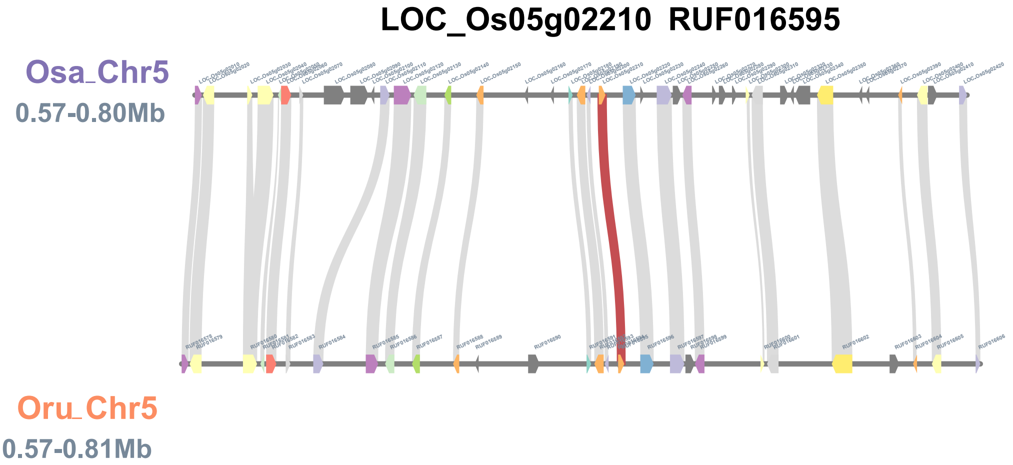
LOC_Os05g02210 RUF016595
Osa_Chr5
0.57-0.80Mb
Oru_Chr5
0.57-0.81Mb

LOC_Os05g04160 RUF016732

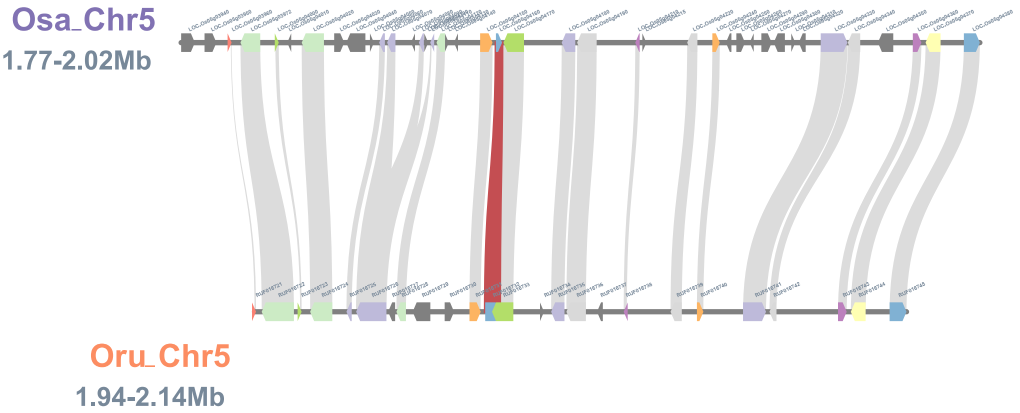

LOC_Os05g05490 RUF016840

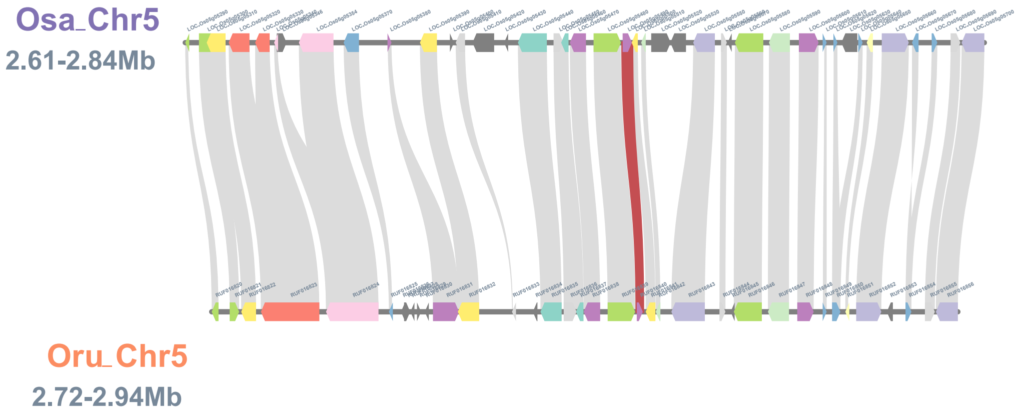

LOC_Os05g05490 RUF016840

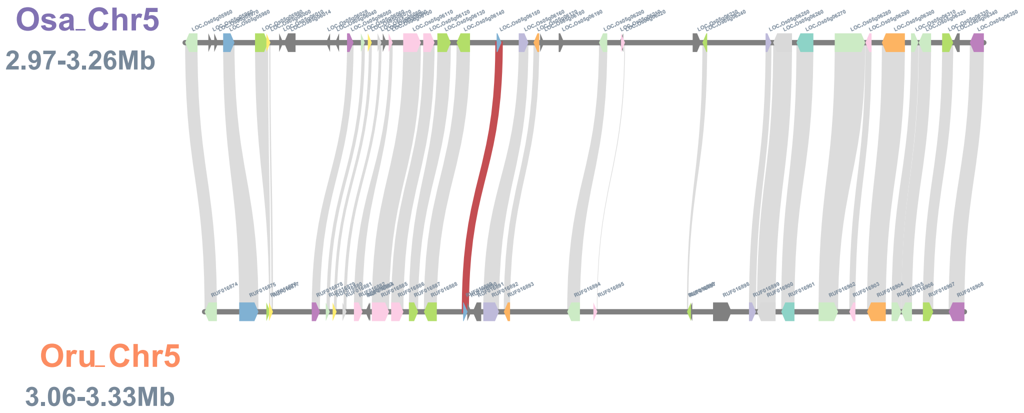

LOC_Os05g11700 RUF017220

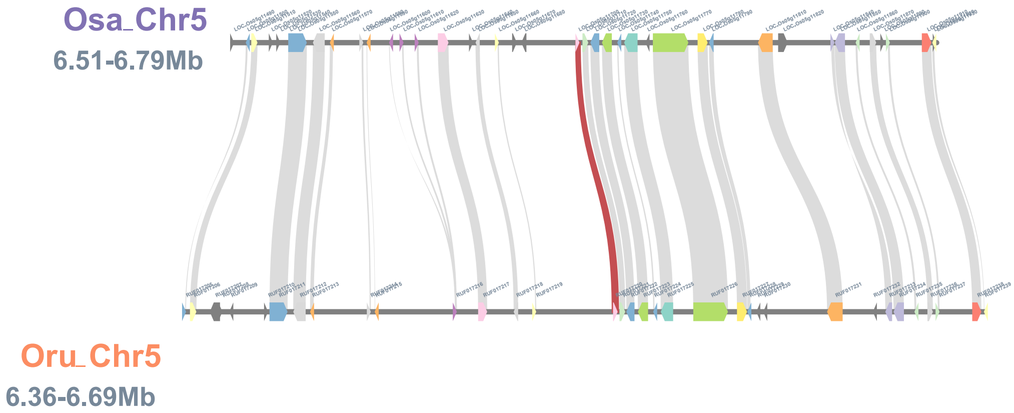

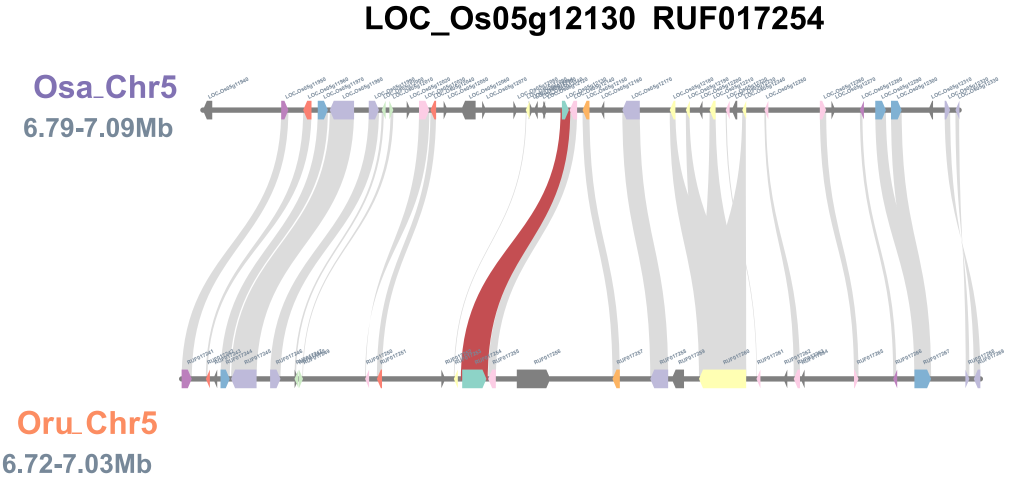
LOC_Os05g12130 RUF017254
Osa_Chr5
6.79-7.09Mb
Oru_Chr5
6.72-7.03Mb

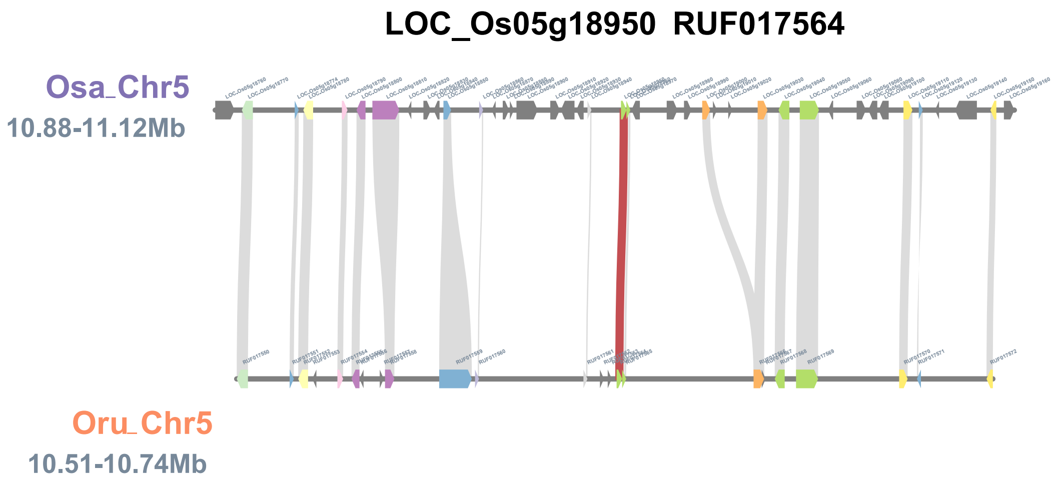
LOC_Os05g18950 RUF017564
Osa_Chr5
10.88-11.12Mb
Oru_Chr5
10.51-10.74Mb

LOC_Os05g19380 RUF017580

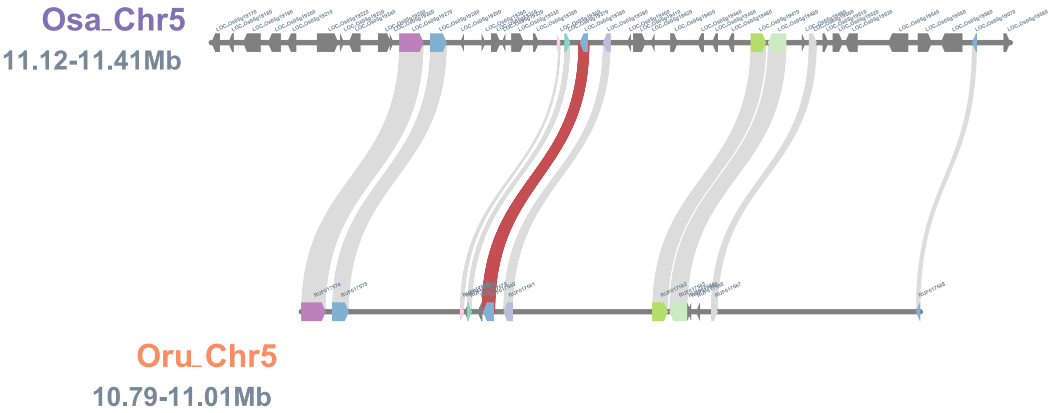

LOC_Os05g19390 RUF017581

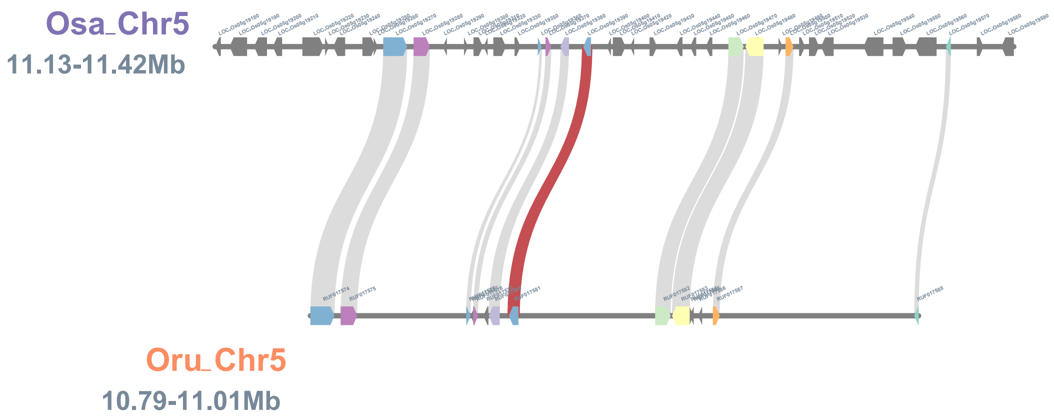

LOC_Os05g22870 RUF017639

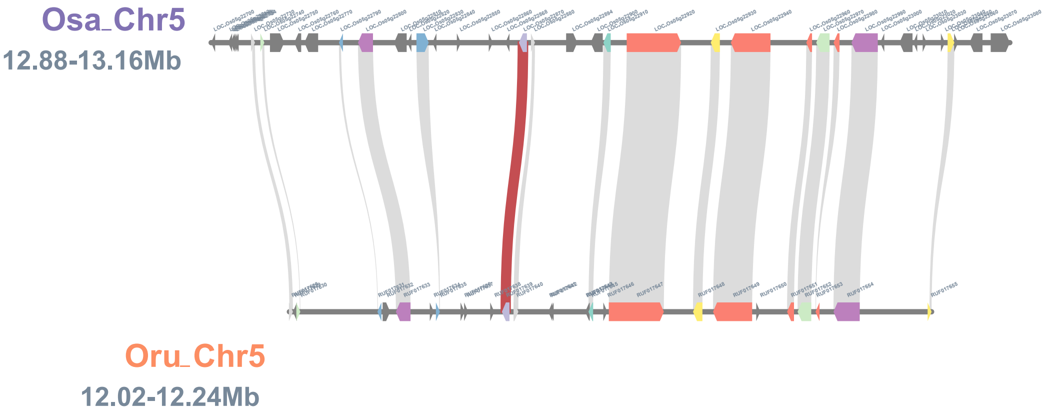

LOC_Os05g24930 RUF017735

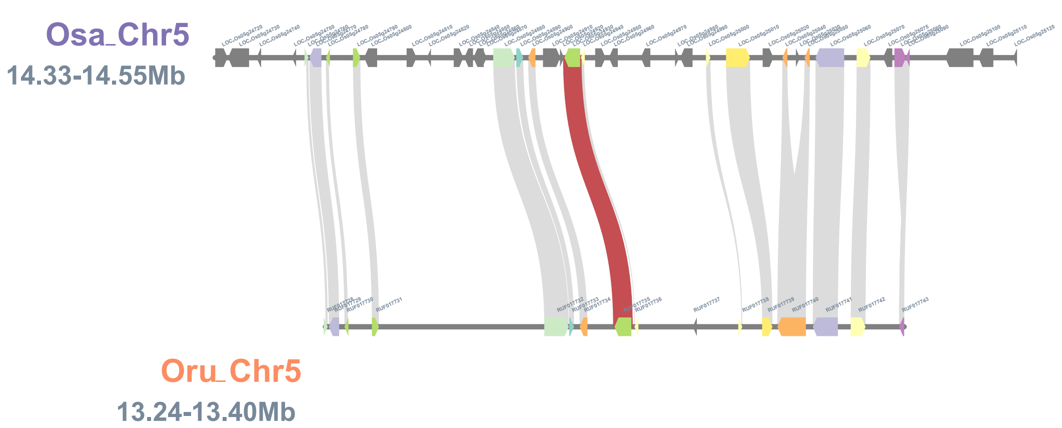

LOC_Os05g25060 RUF017741

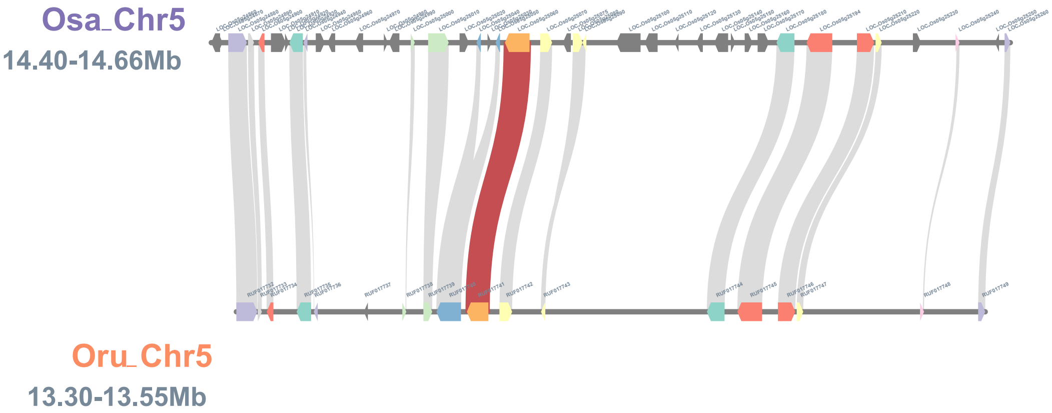

LOC_Os05g28720 RUF017950

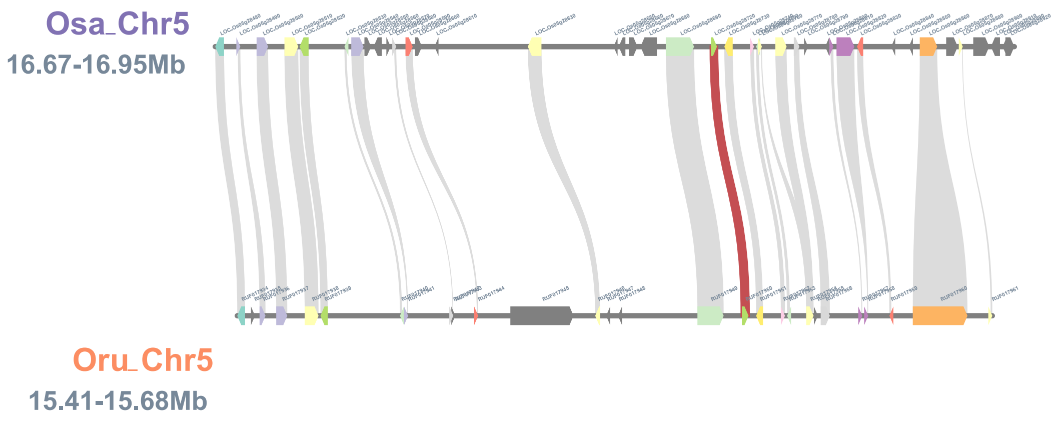

LOC_Os05g30240 RUF018011

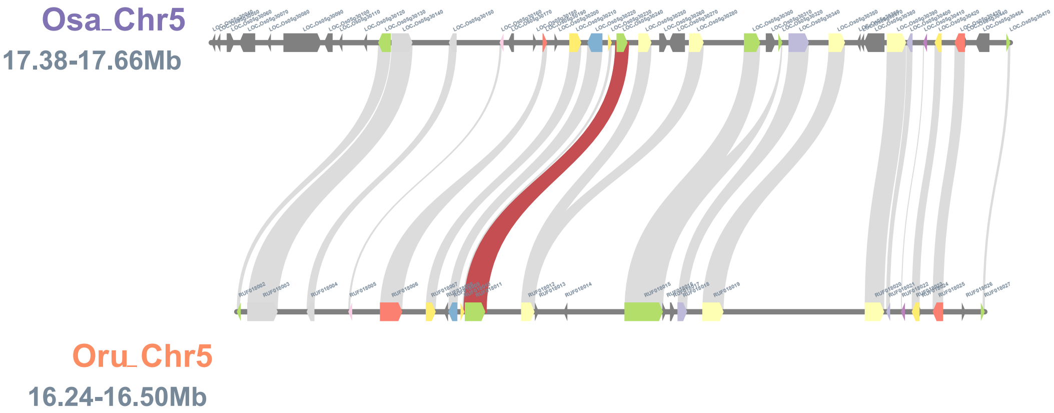

LOC_Os05g30710 RUF018052

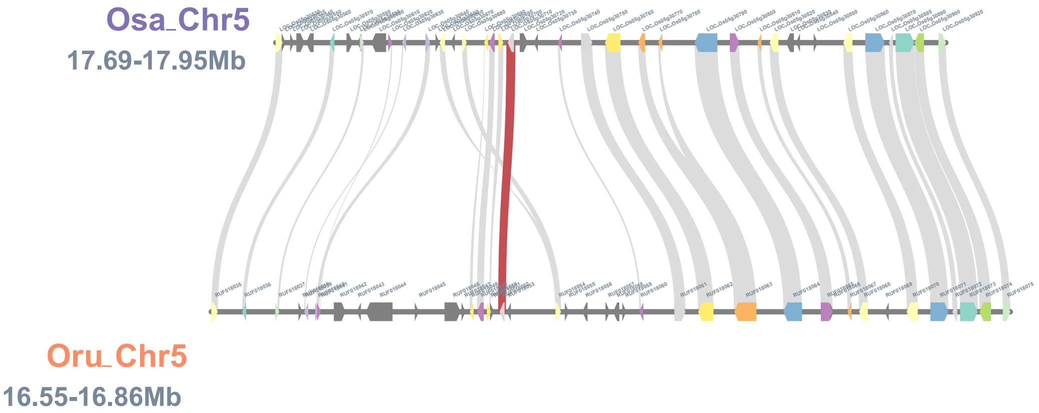

LOC_Os05g32730 RUF018213

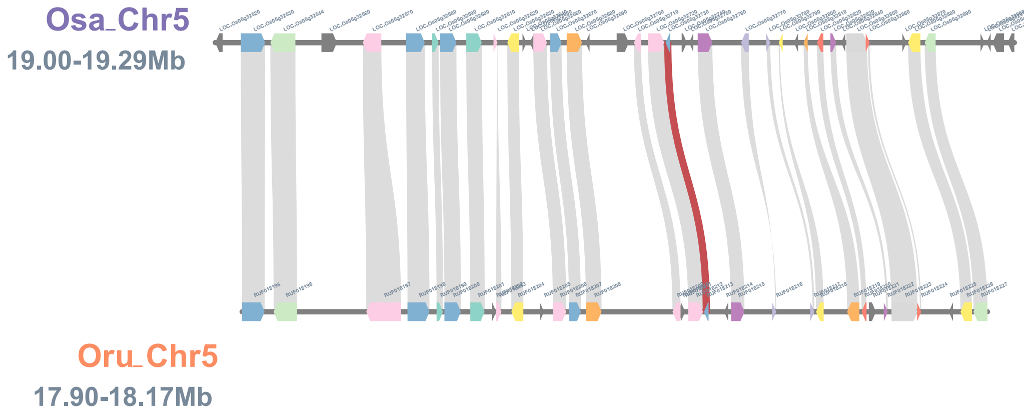

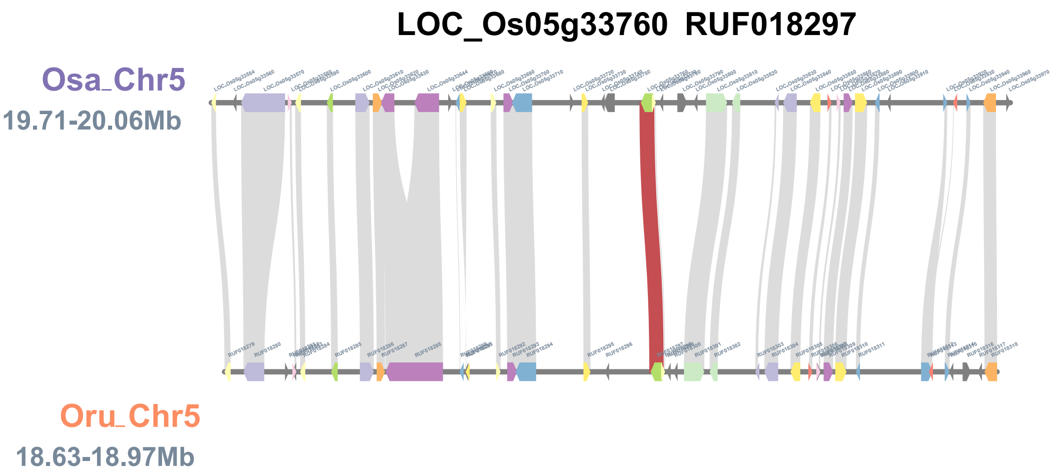

LOC_Os05g33760 RUF018297
Osa_Chr5
19.71-20.06Mb
Oru_Chr5
18.63-18.97Mb

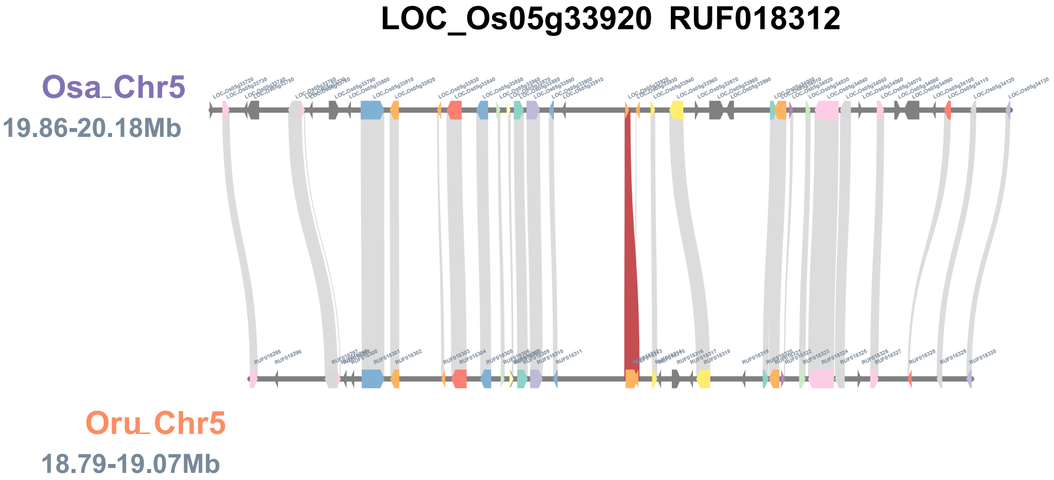
LOC_Os05g33920 RUF018312
Osa_Chr5
19.86-20.18Mb
Oru_Chr5
18.79-19.07Mb

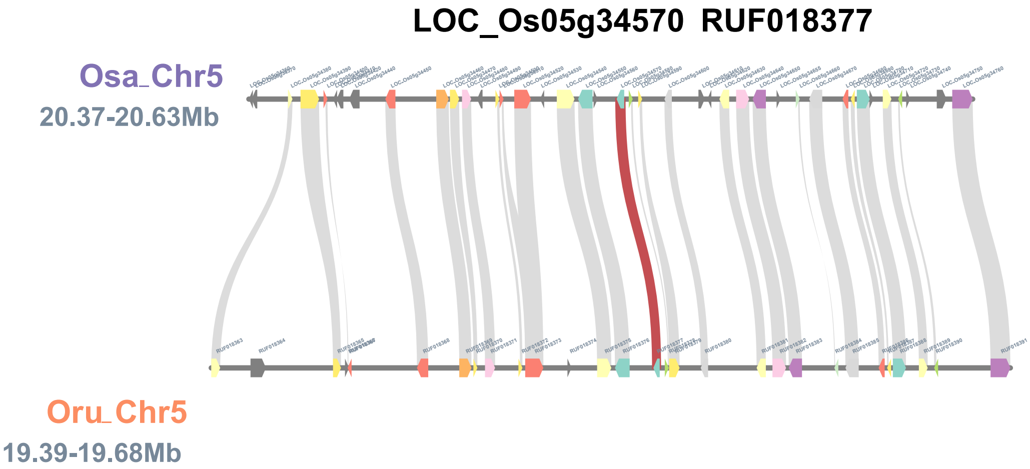
LOC_Os05g34570 RUF018377
Osa_Chr5
20.37-20.63Mb
Oru_Chr5
19.39-19.68Mb

LOC_Os05g36350 RUF018496

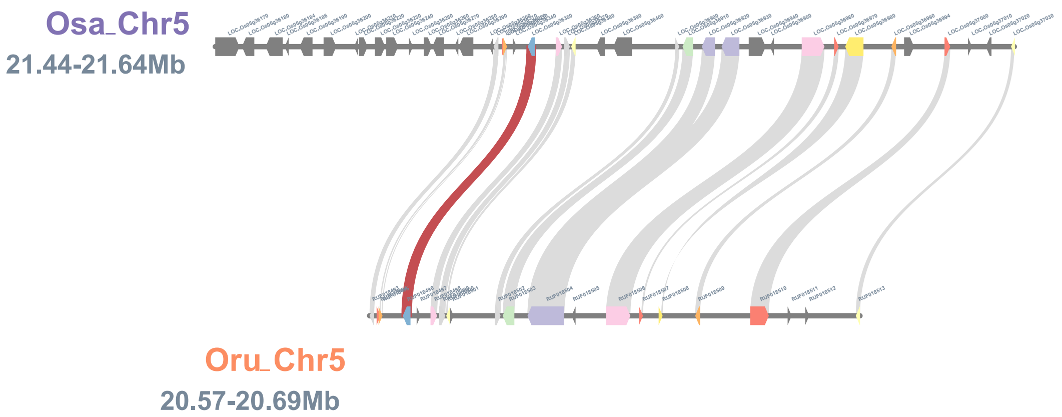

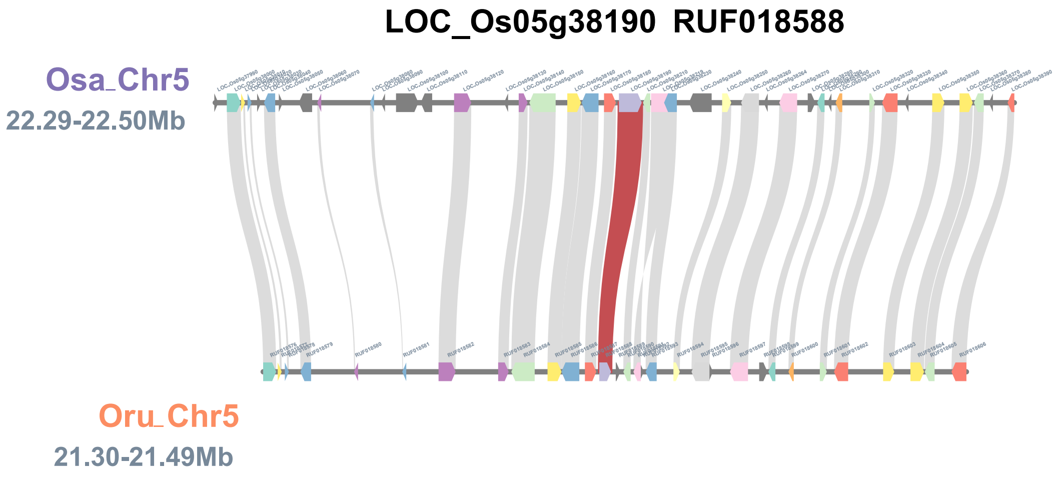
LOC_Os05g38190 RUF018588
Osa_Chr5
22.29-22.50Mb
Oru_Chr5
21.30-21.49Mb

LOC_Os05g40320 RUF018772

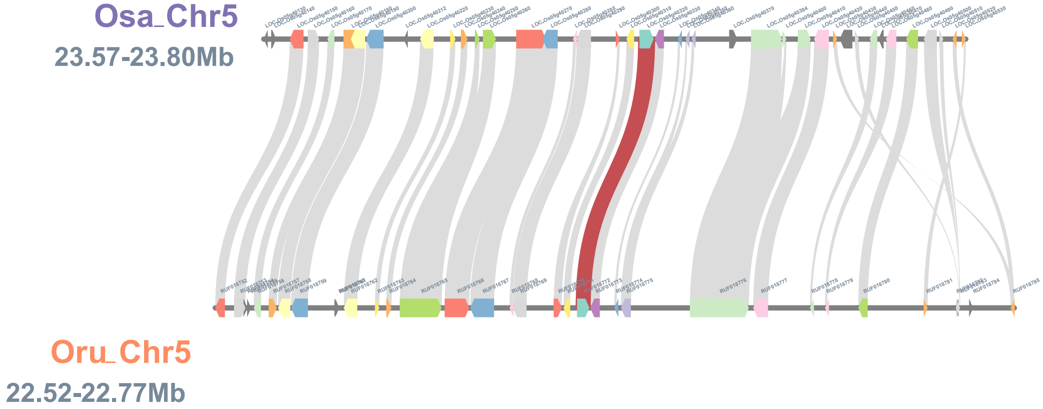

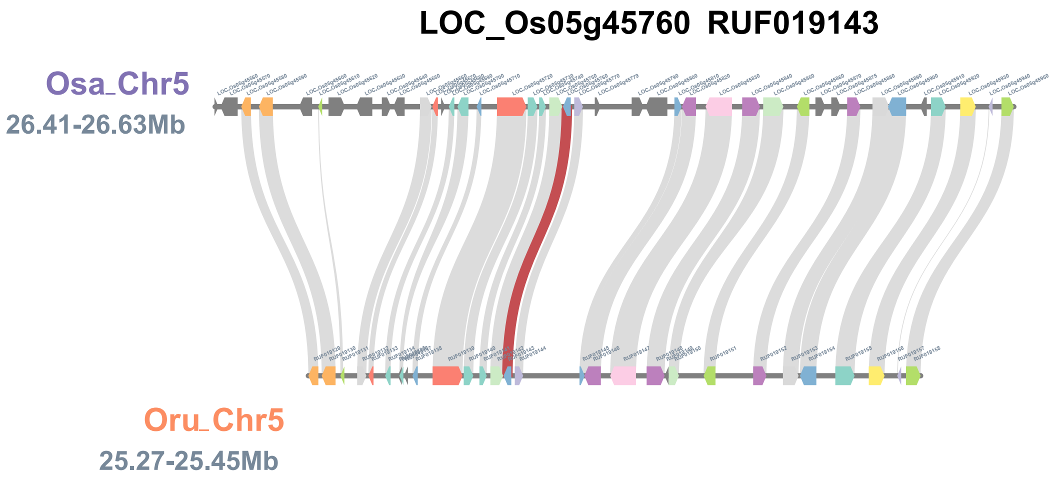
LOC_Os05g45760 RUF019143
Osa_Chr5
26.41-26.63Mb
Oru_Chr5
25.27-25.45Mb

LOC_Os05g45850 RUF019150

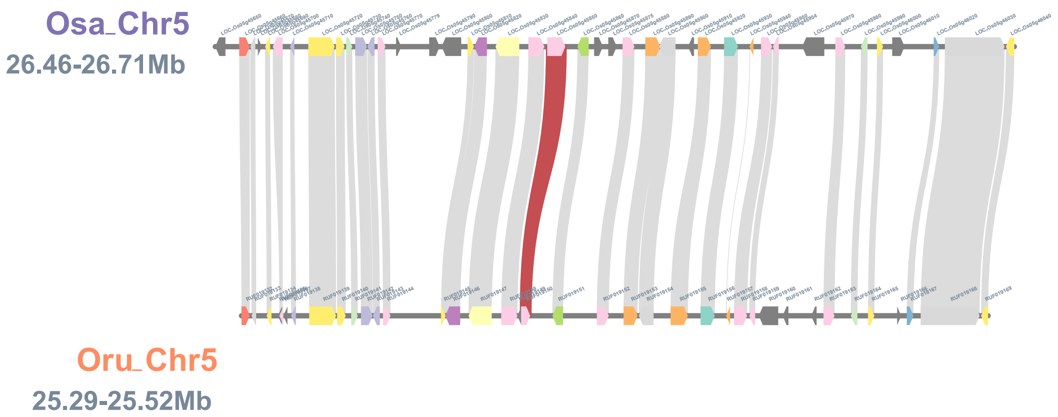

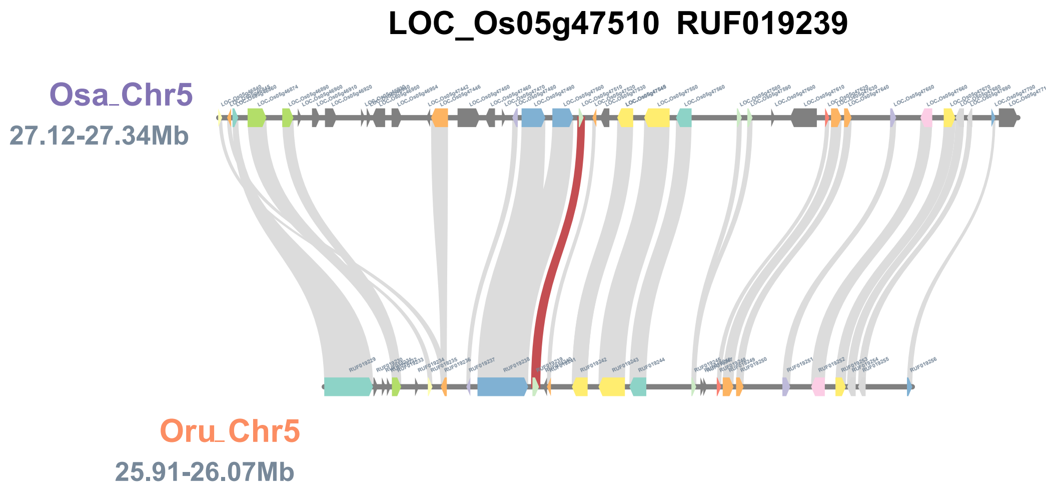
LOC_Os05g47510 RUF019239
Osa_Chr5
27.12-27.34Mb
Oru_Chr5
25.91-26.07Mb

LOC_Os05g49740 RUF019430

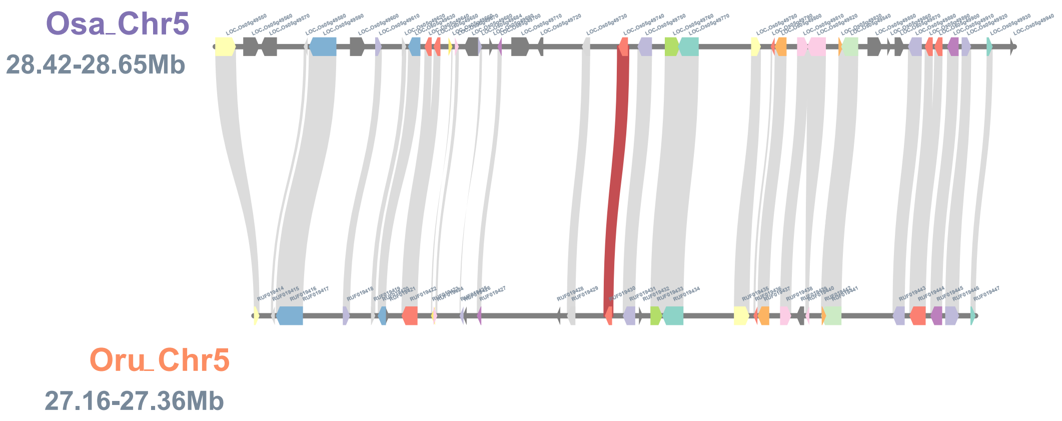

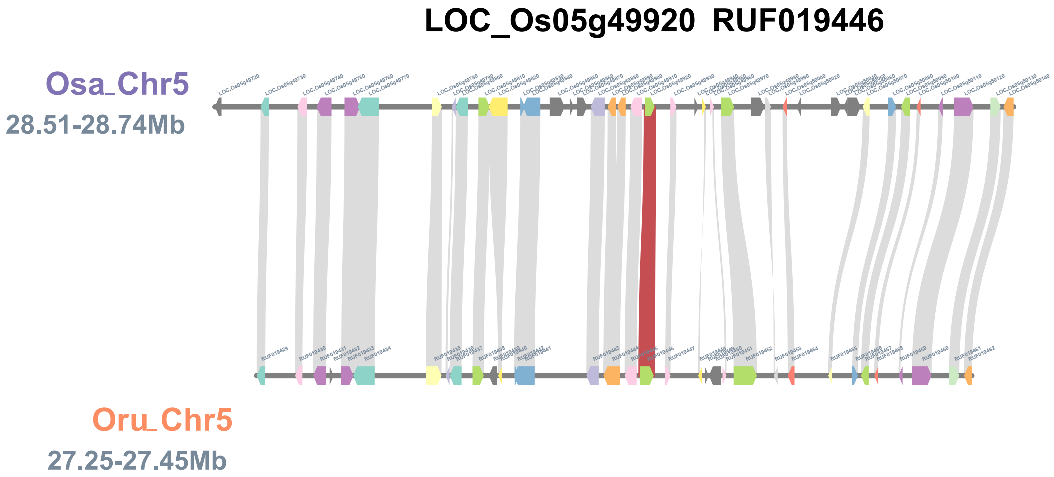
LOC_Os05g49920 RUF019446
Osa_Chr5
28.51-28.74Mb
Oru_Chr5
27.25-27.45Mb

LOC_Os05g50460 RUF019488

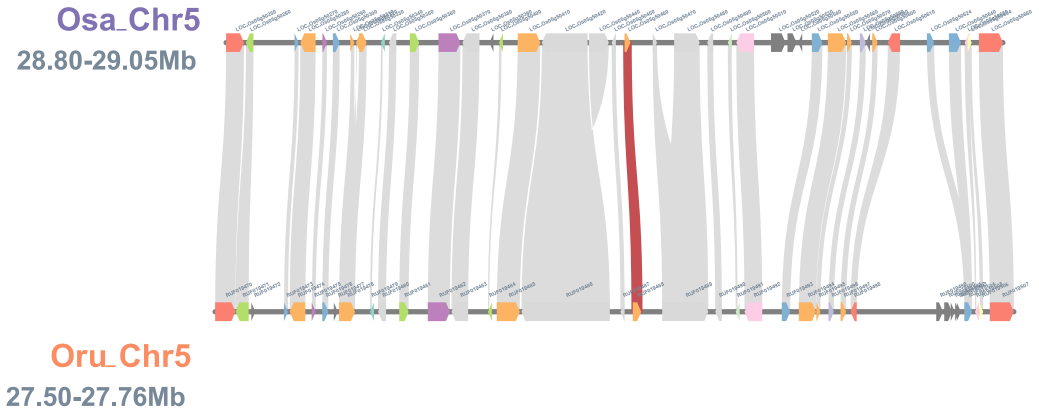

LOC_Os05g50690 RUF019508

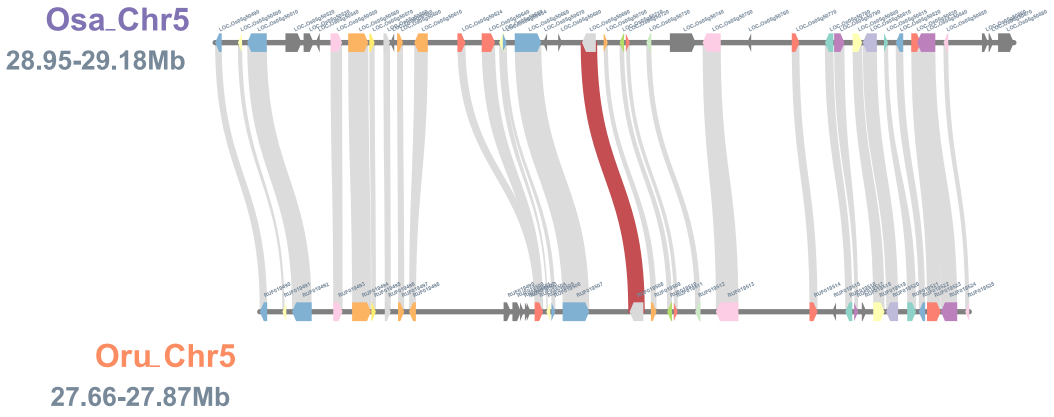

LOC_Os05g50950 RUF019536

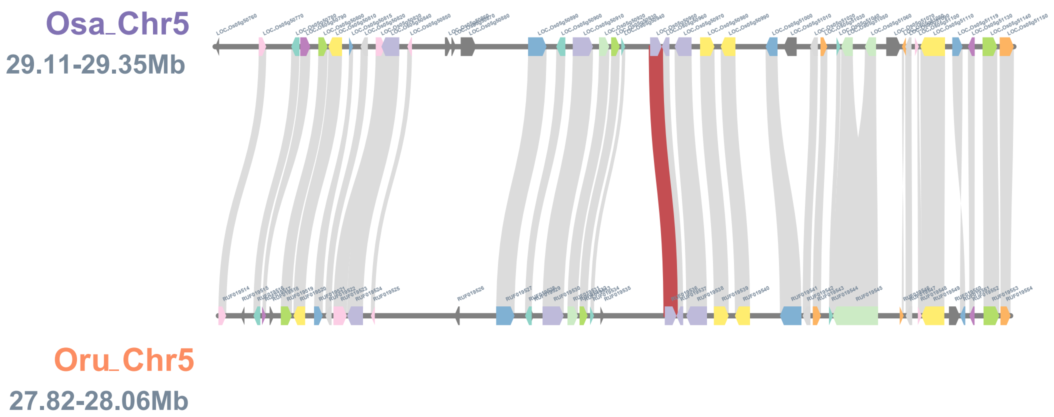

LOC_Os06g02120 RUF019649

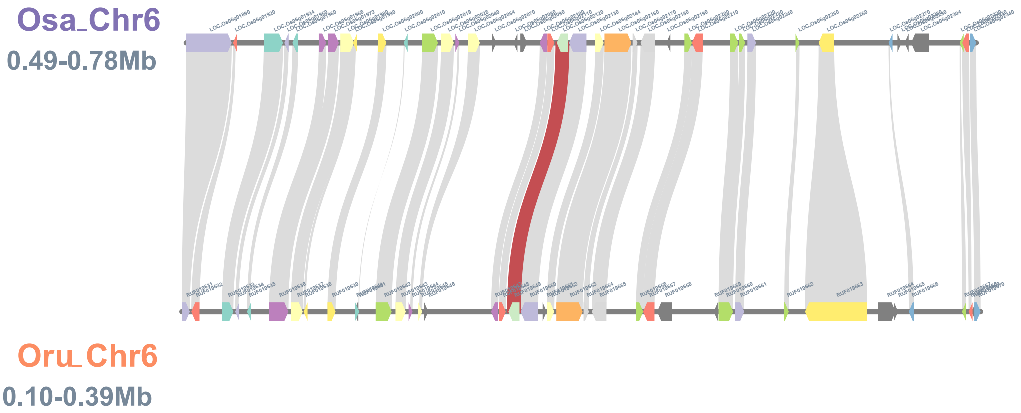

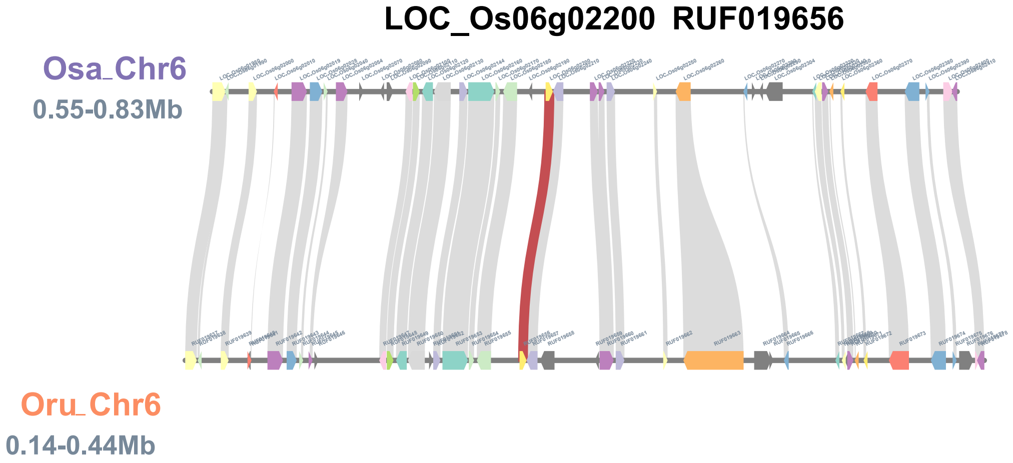
LOC_Os06g02200 RUF019656
Osa_Chr6
0.55-0.83Mb
Oru_Chr6
0.14-0.44Mb

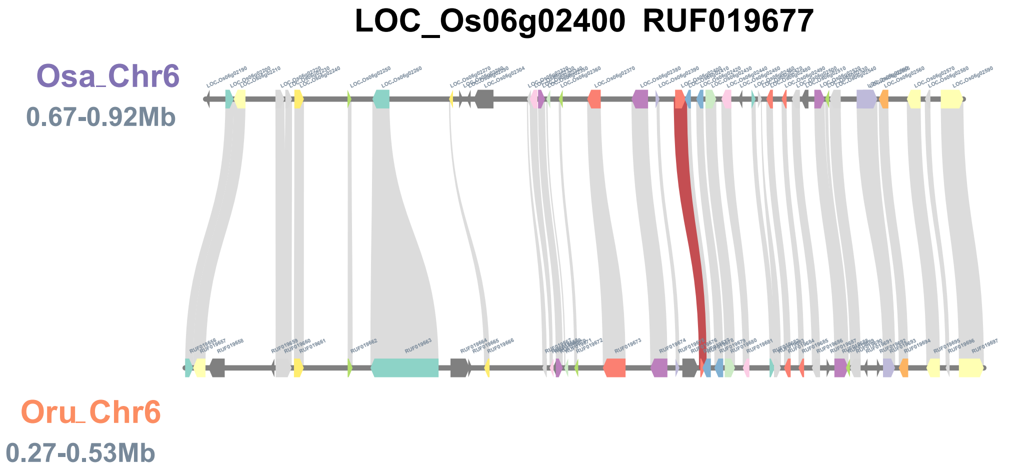
LOC_Os06g02400 RUF019677
Osa_Chr6
0.67-0.92Mb
Oru_Chr6
0.27-0.53Mb

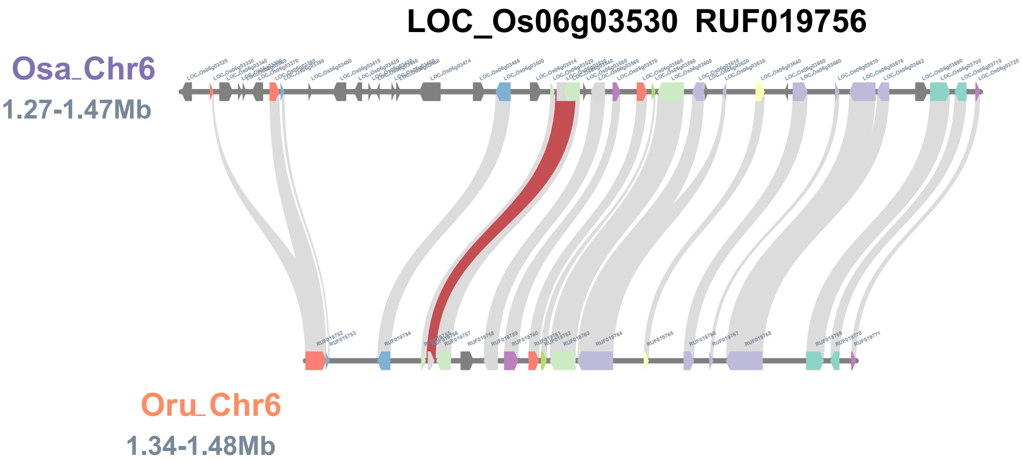
LOC_Os06g03530 RUF019756
Osa_Chr6
1.27-1.47Mb
Oru_Chr6
1.34-1.48Mb

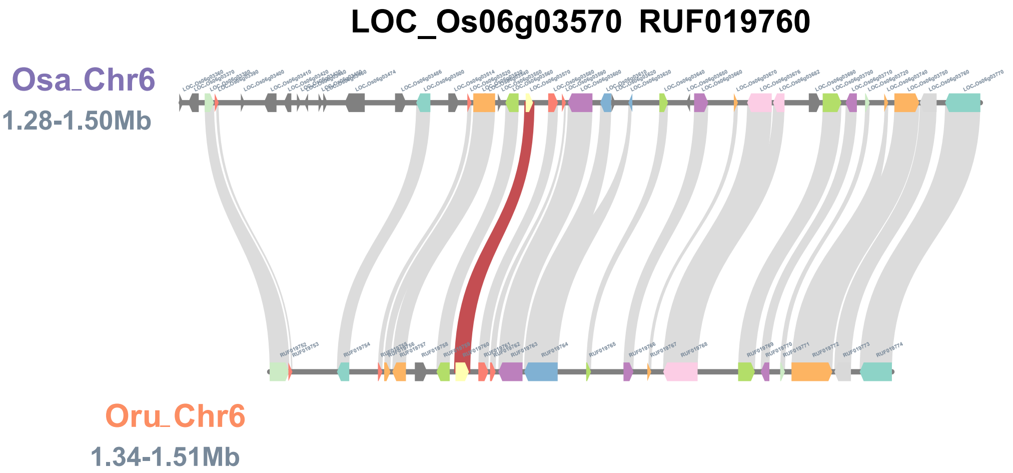
LOC_Os06g03570 RUF019760
Osa_Chr6
1.28-1.50Mb
Oru_Chr6
1.34-1.51Mb

LOC_Os06g05140 RUF019886

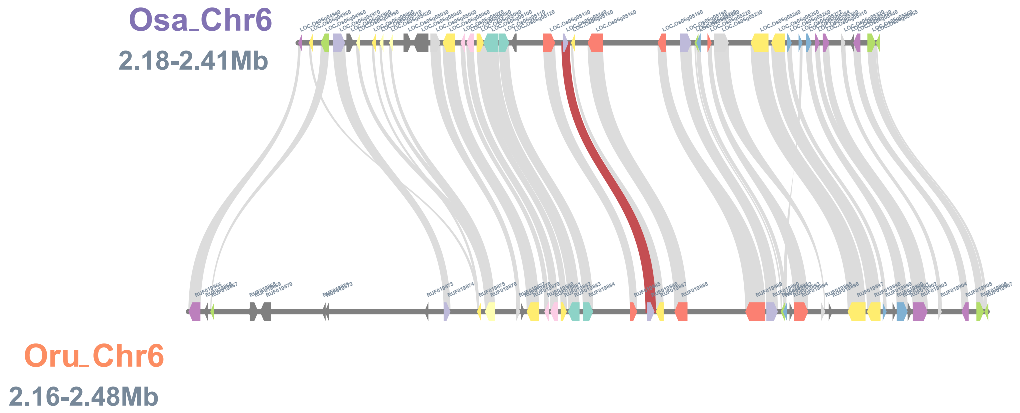

LOC_Os06g05920 RUF019965

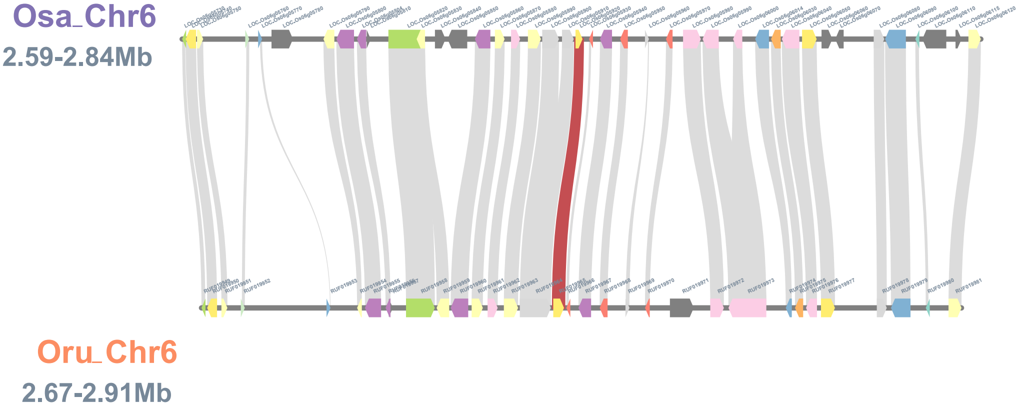

LOC_Os06g07550 RUF020110

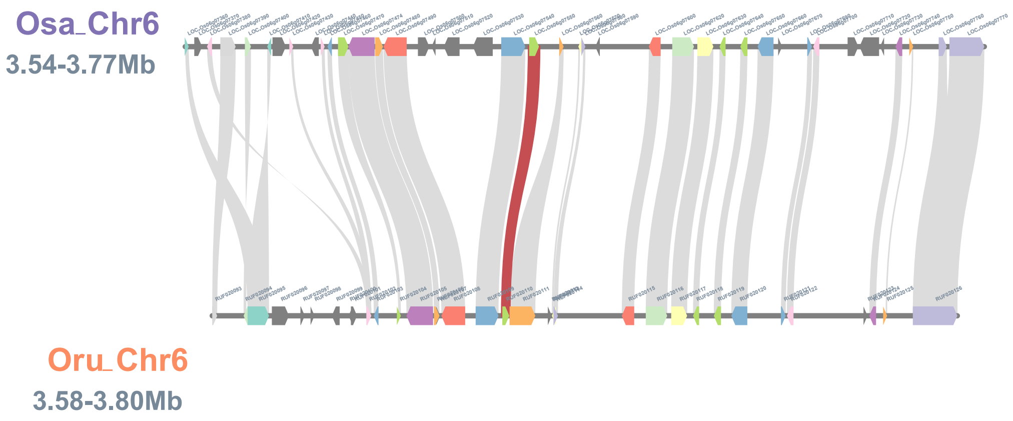

LOC_Os06g08570 RUF020177

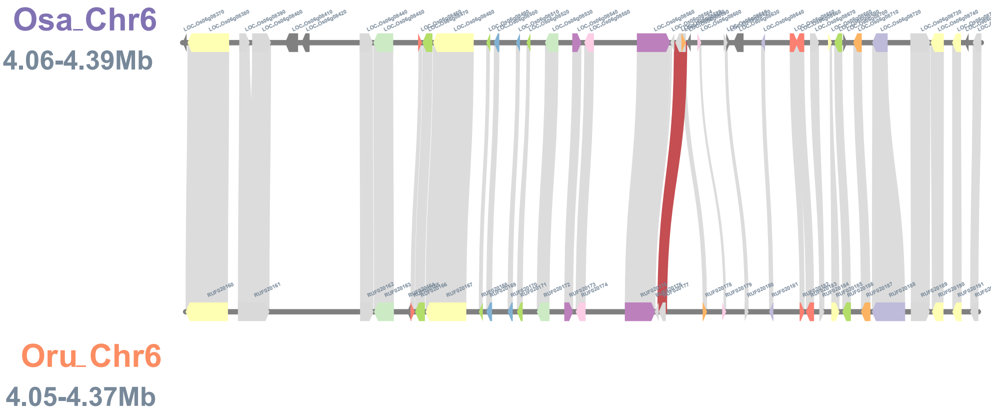

LOC_Os06g08650 RUF020182

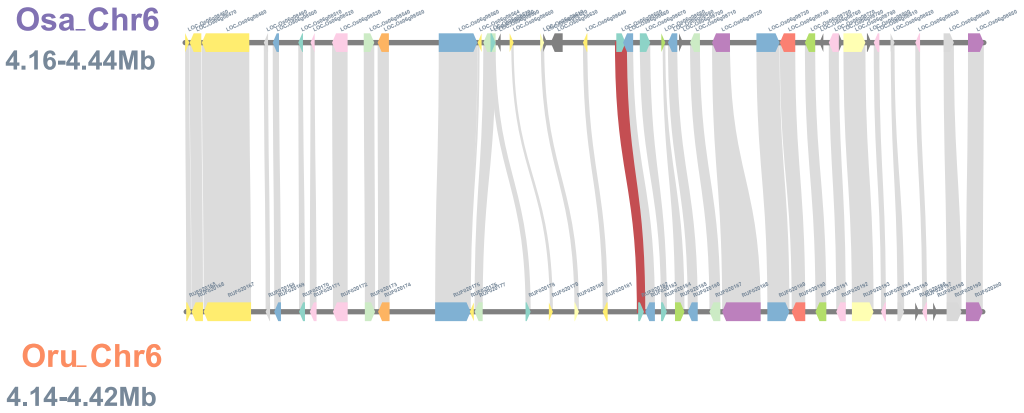

LOC_Os06g08660 RUF020183

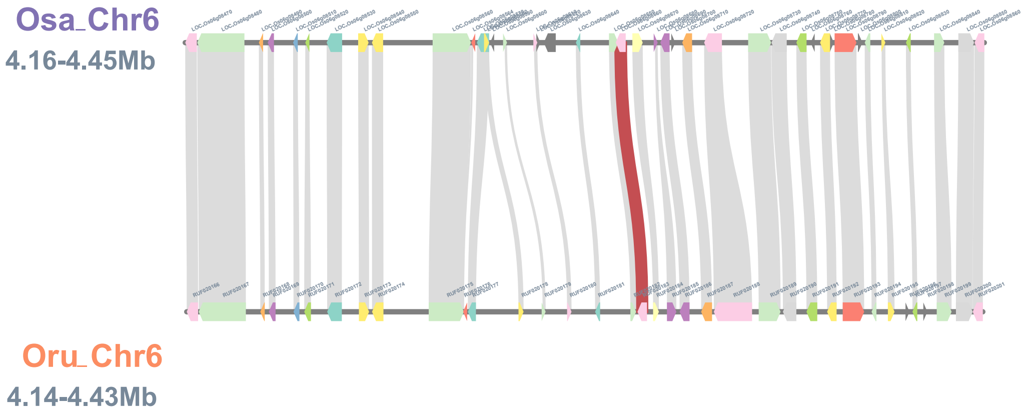

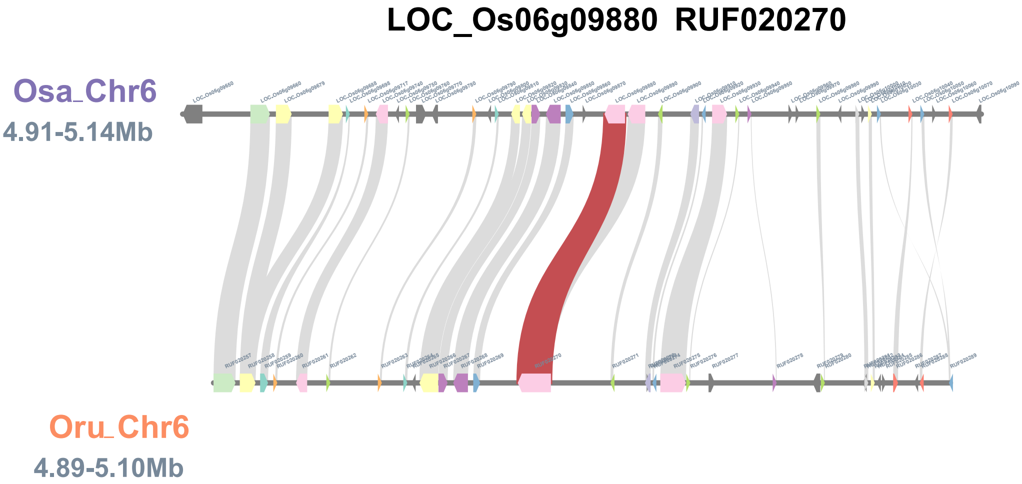
LOC_Os06g09880 RUF020270
Osa_Chr6
4.91-5.14Mb
Oru_Chr6
4.89-5.10Mb

LOC_Os06g11300 RUF020385

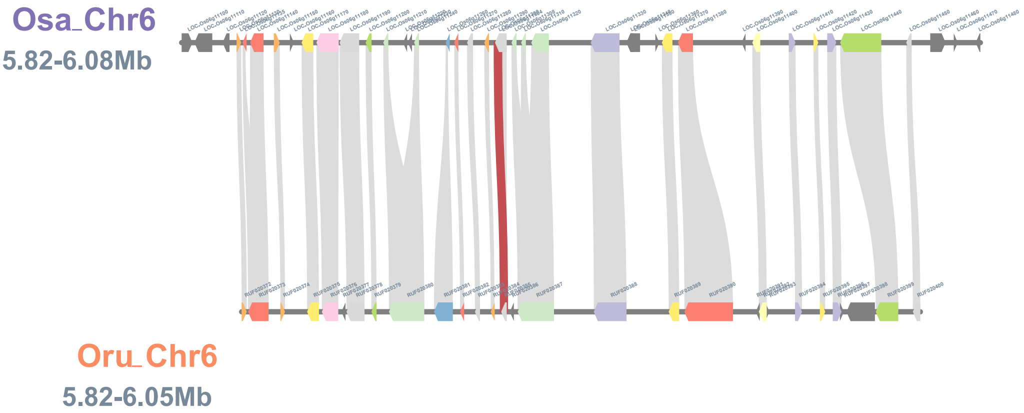

LOC_Os06g12360 RUF020471

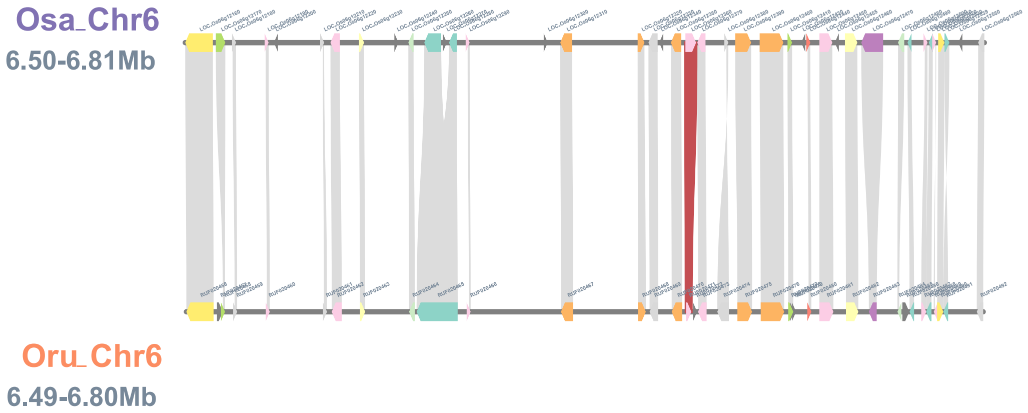

LOC_Os06g12510 RUF020488

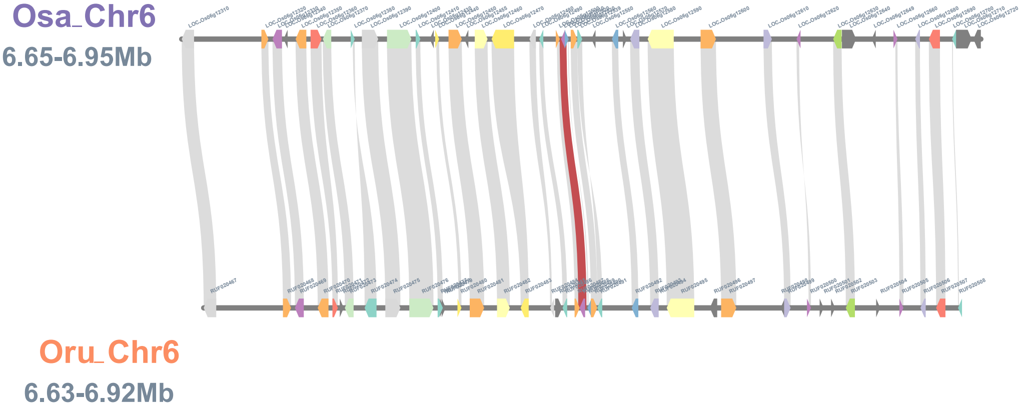

LOC_Os06g13950 RUF020601

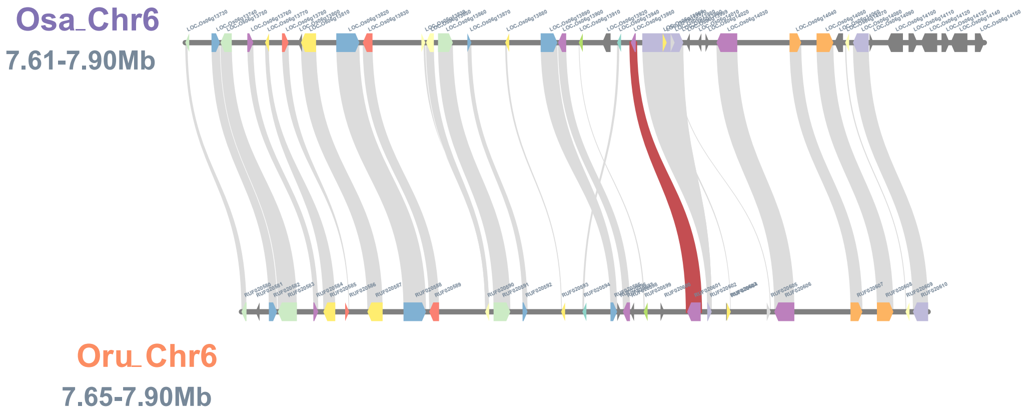

LOC_Os06g20870 RUF020951

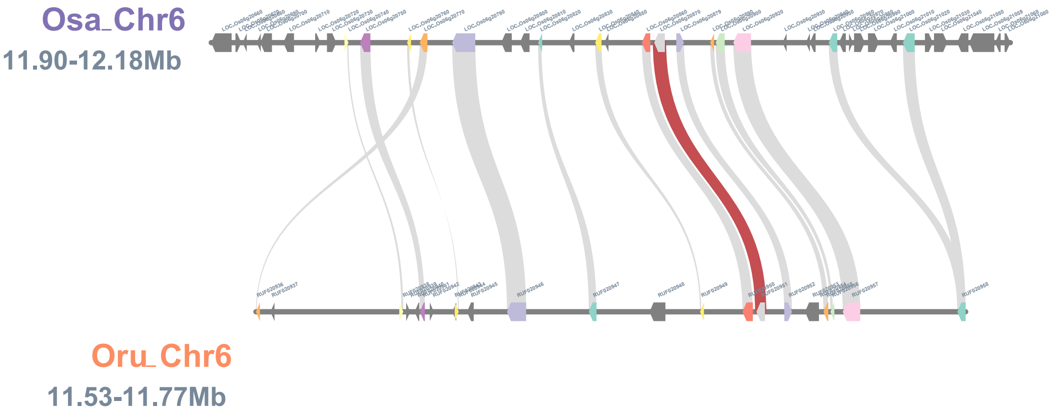

LOC_Os06g27790 RUF021238

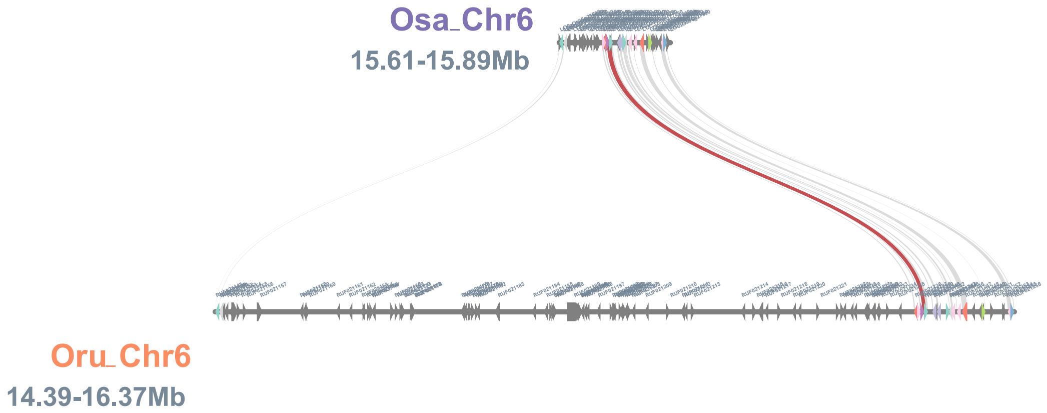

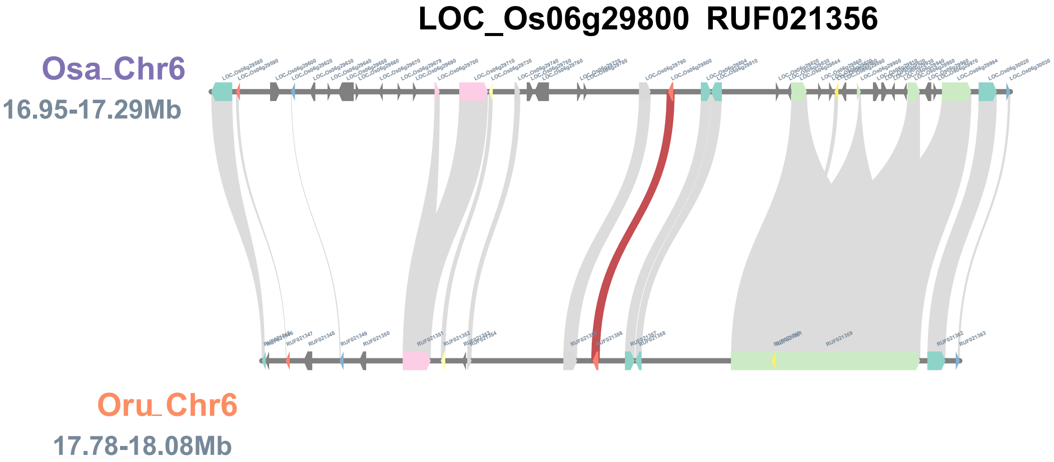
LOC_Os06g29800 RUF021356
Osa_Chr6
16.95-17.29Mb
Oru_Chr6
17.78-18.08Mb

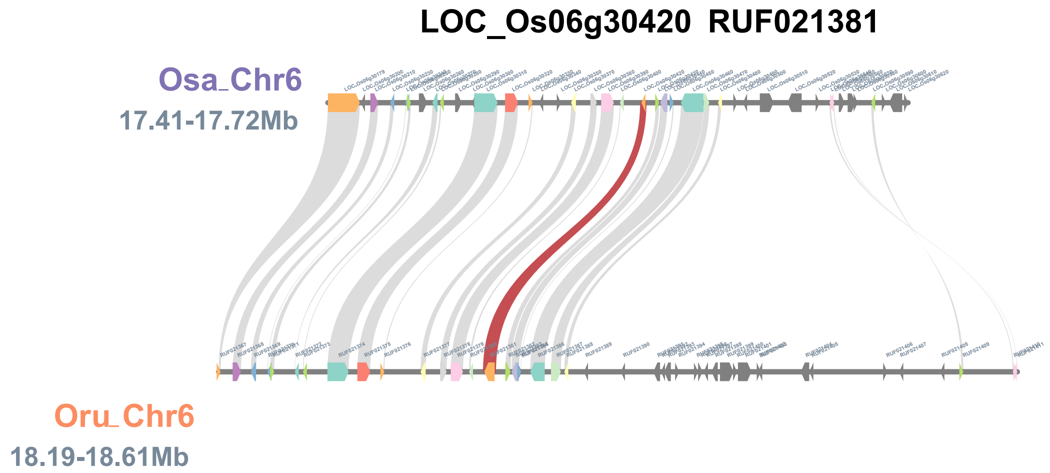
LOC_Os06g30420 RUF021381
Osa_Chr6
17.41-17.72Mb
Oru_Chr6
18.19-18.61Mb

LOC_Os06g30940 RUF021426

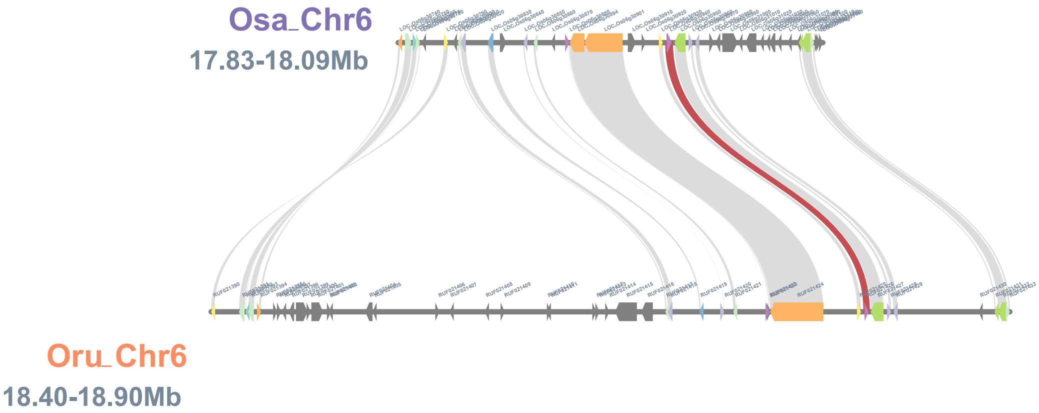

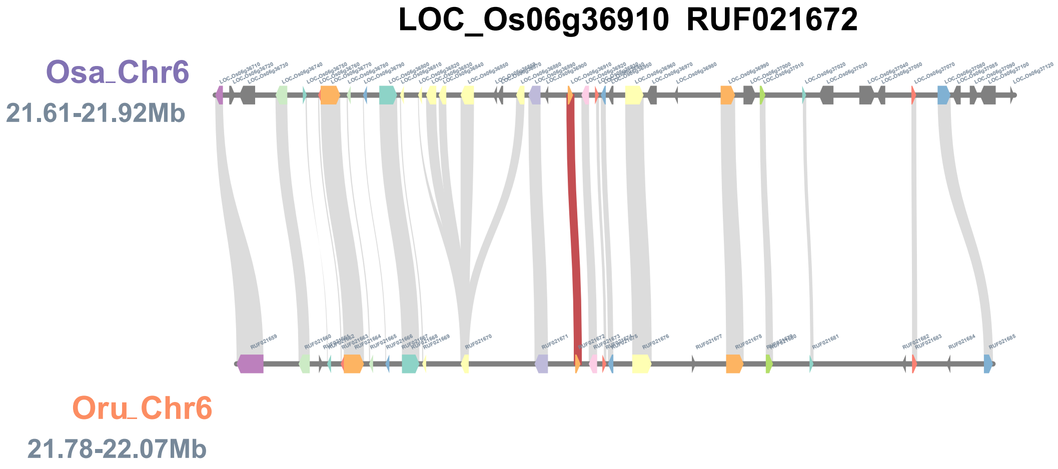
LOC_Os06g36910 RUF021672
Osa_Chr6
21.61-21.92Mb
Oru_Chr6
21.78-22.07Mb

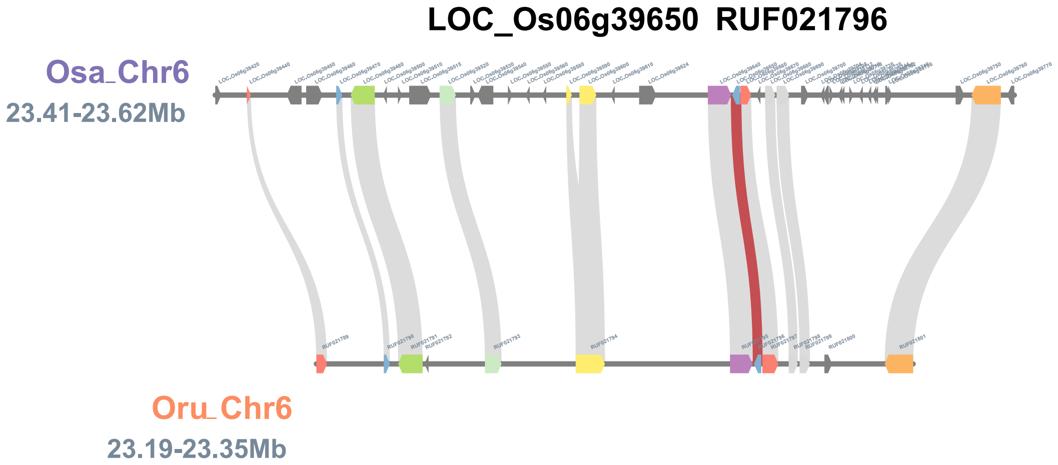
LOC_Os06g39650 RUF021796
Osa_Chr6
23.41-23.62Mb
Oru_Chr6
23.19-23.35Mb

LOC_Os06g40860 RUF021883

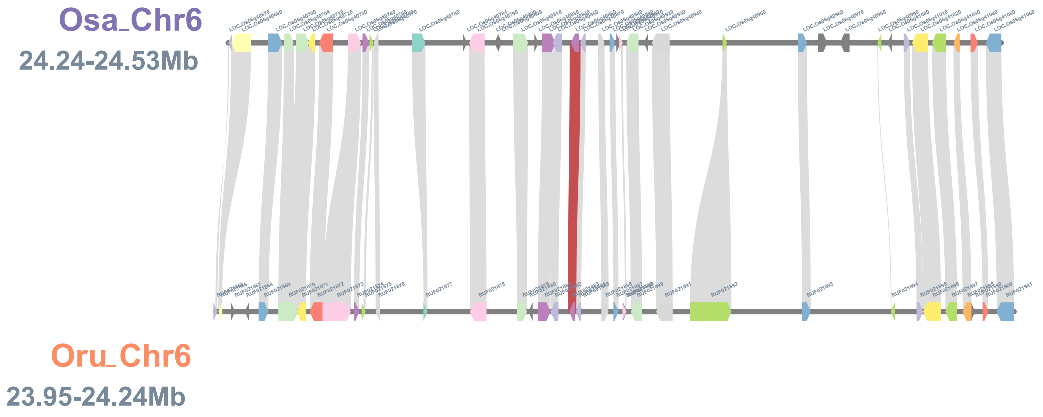

LOC_Os06g41040 RUF021898

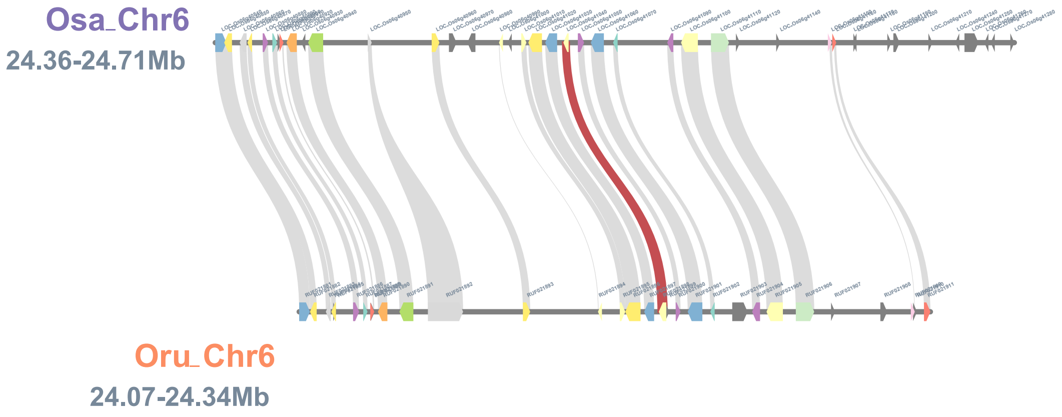

LOC_Os06g46840 RUF022354

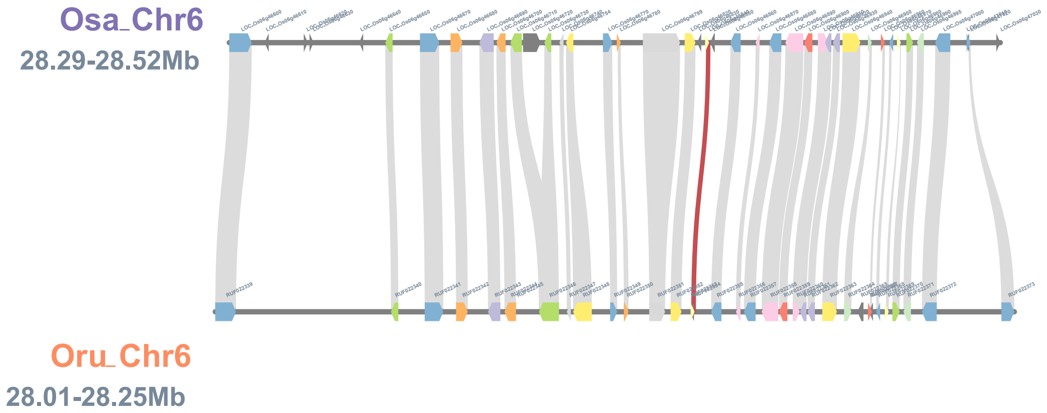

LOC_Os06g47570 RUF022412

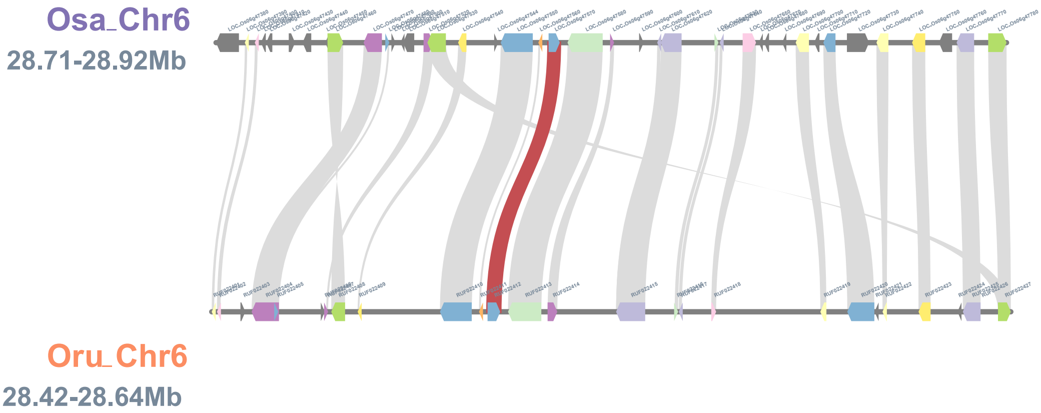

LOC_Os06g47920 RUF022442

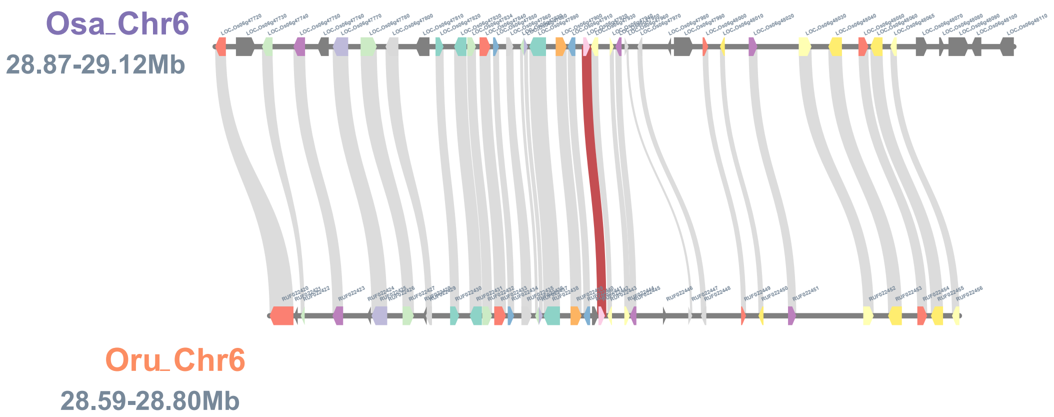

LOC_Os06g47950 RUF022445

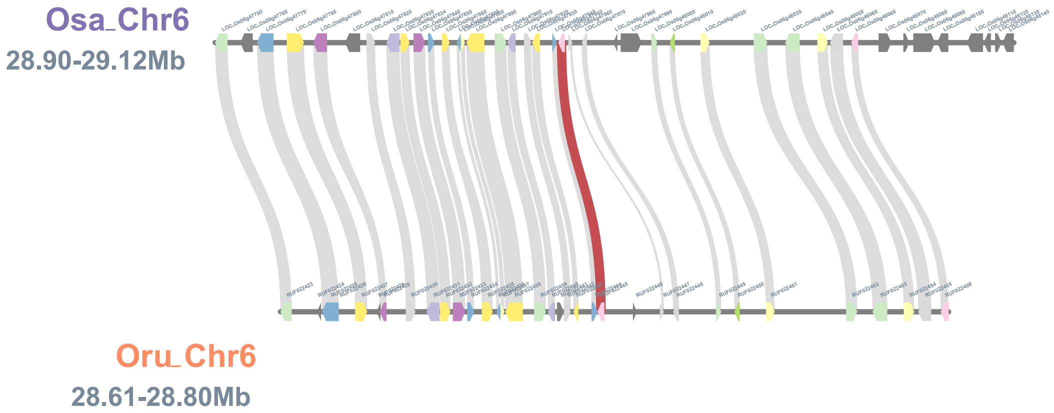

LOC_Os06g49670 RUF022558

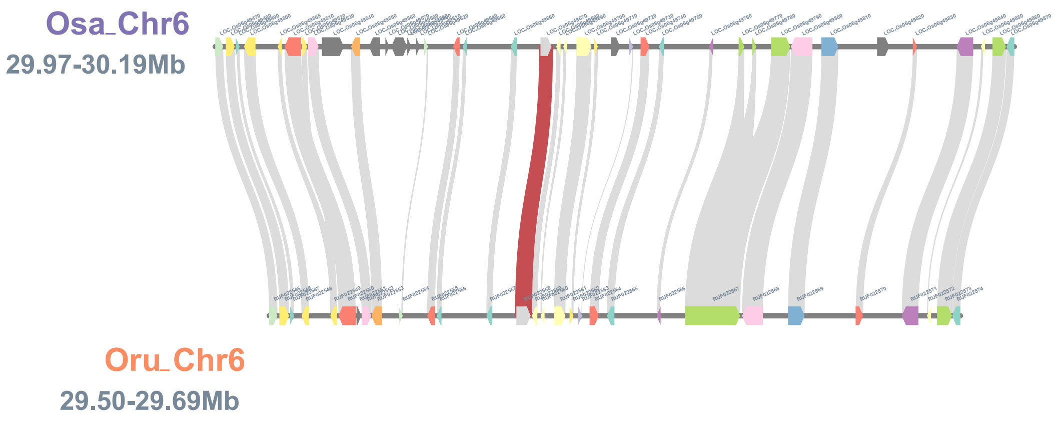

LOC_Os07g02280 RUF022780

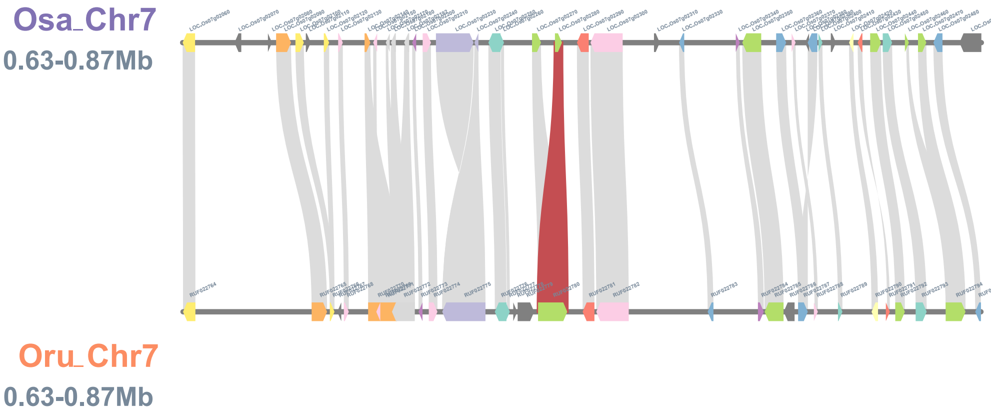

LOC_Os07g05560 RUF022958

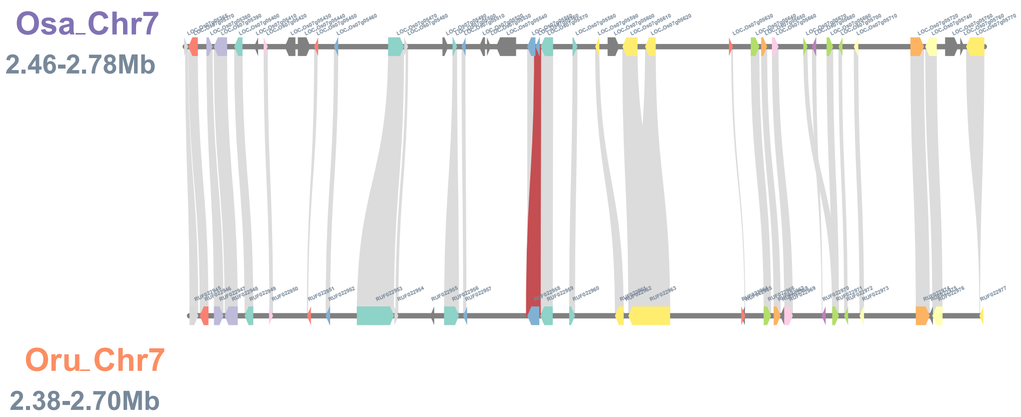

LOC_Os07g06960 RUF023051

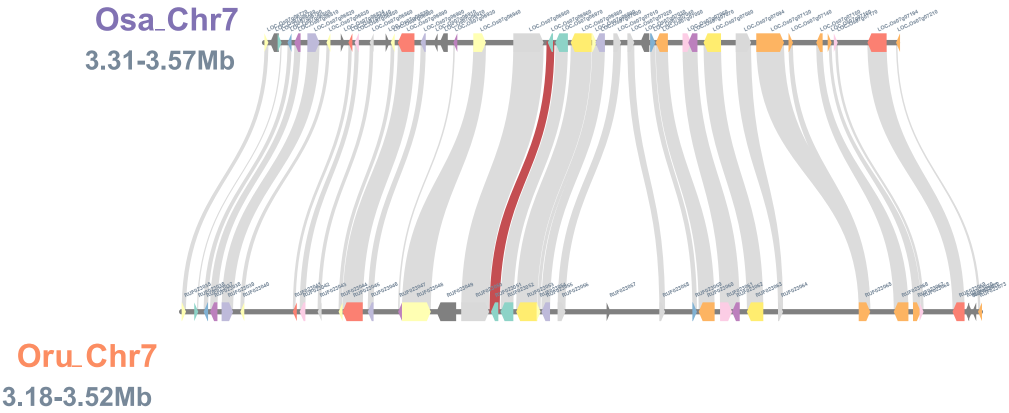

LOC_Os07g07620 RUF023118

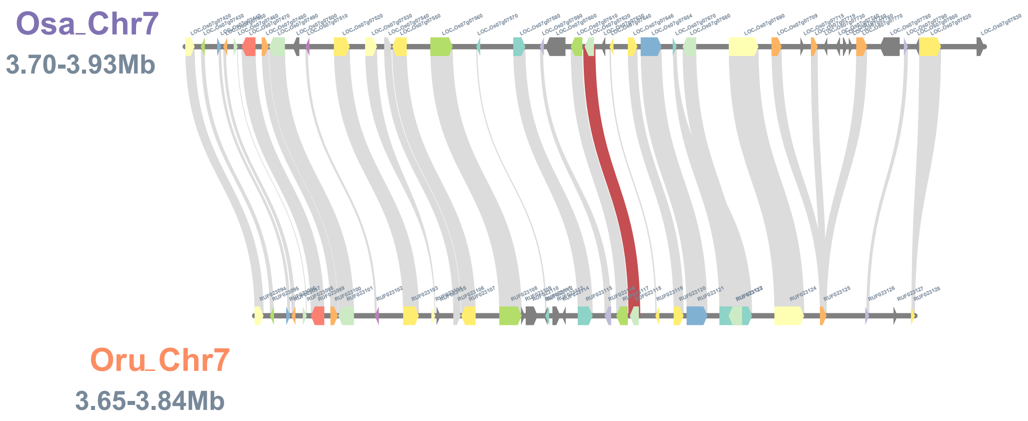

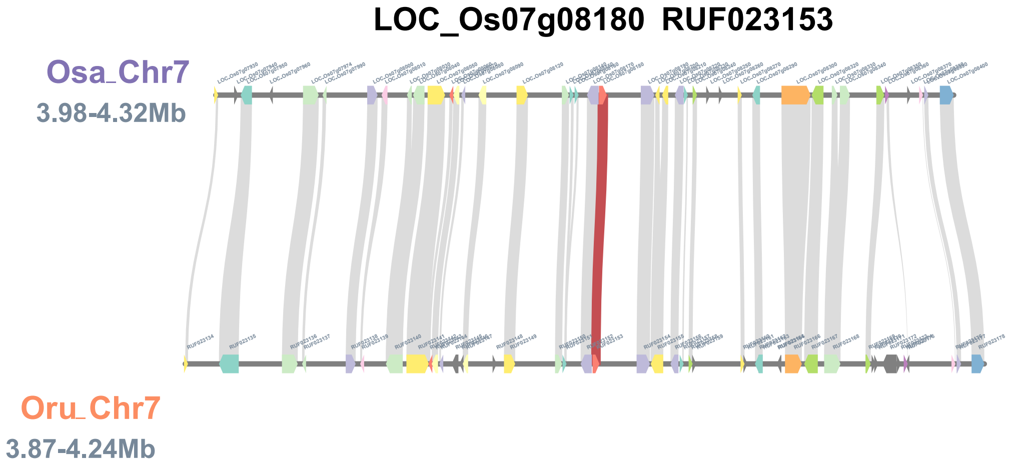
LOC_Os07g08180 RUF023153
Osa_Chr7
3.98-4.32Mb
Oru_Chr7
3.87-4.24Mb

LOC_Os07g09370 RUF023258

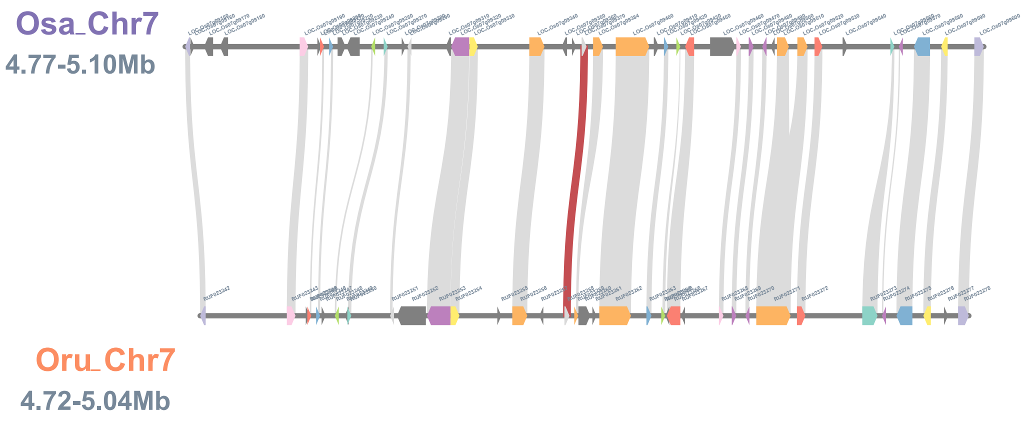

LOC_Os07g10400 RUF023348

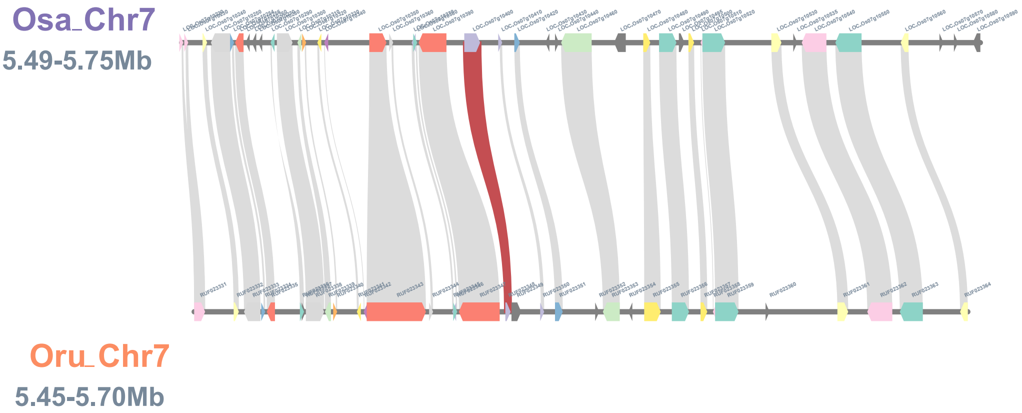

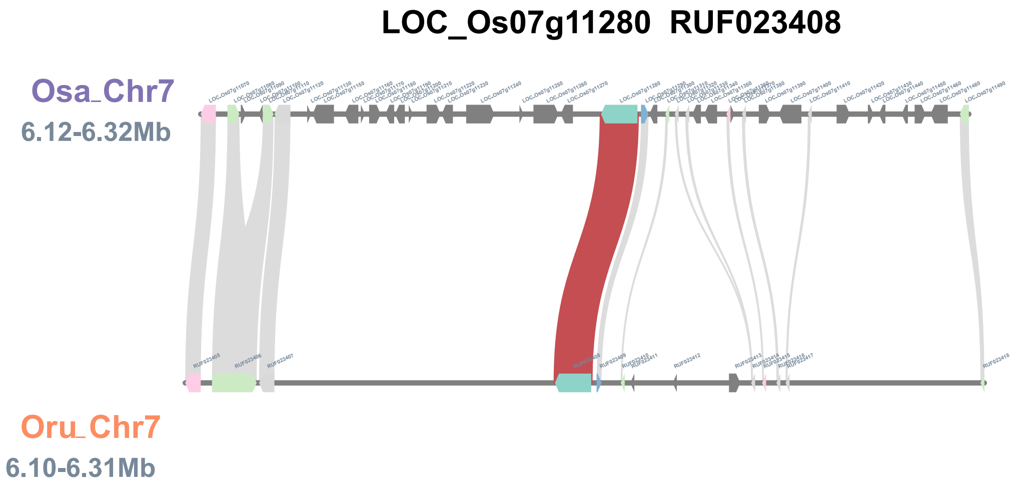
LOC_Os07g11280 RUF023408
Osa_Chr7
6.12-6.32Mb
Oru_Chr7
6.10-6.31Mb

LOC_Os07g12140 RUF023435

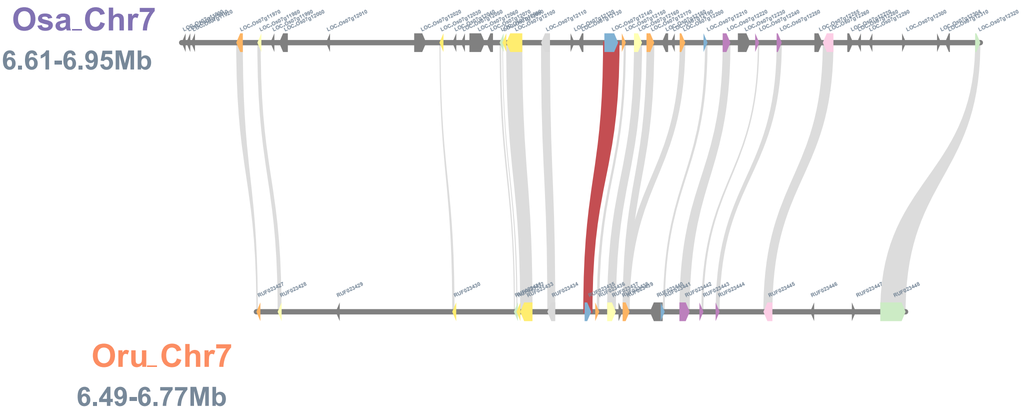

LOC_Os07g13540 RUF023508

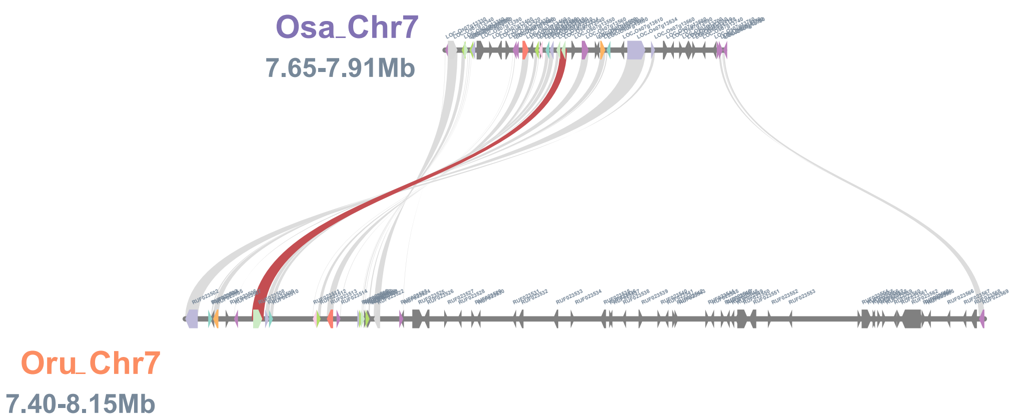

LOC_Os07g14100 RUF023550

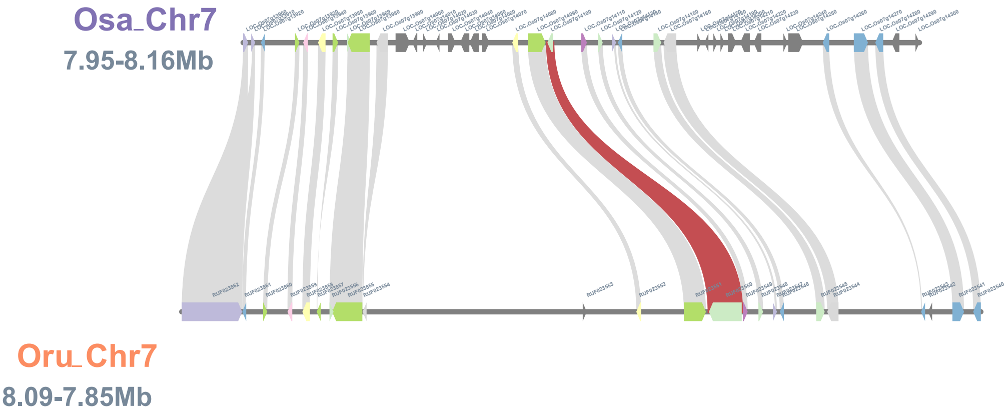

LOC_Os07g14530 RUF023583

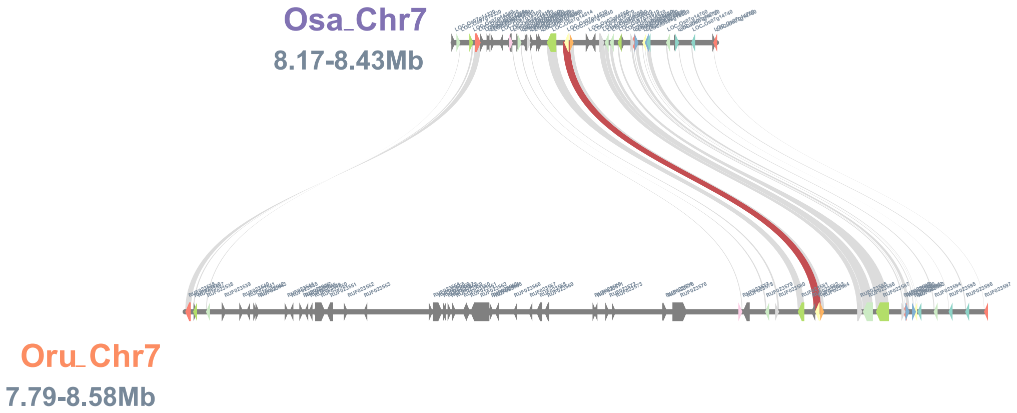

LOC_Os07g15570 RUF023640

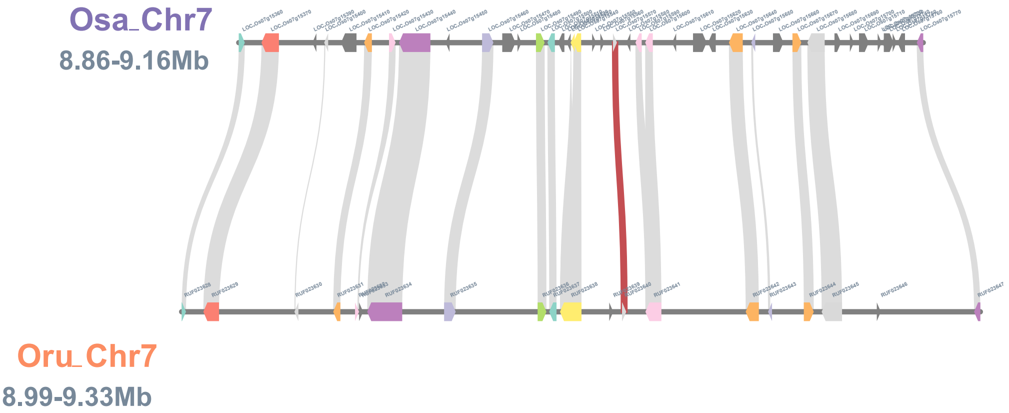

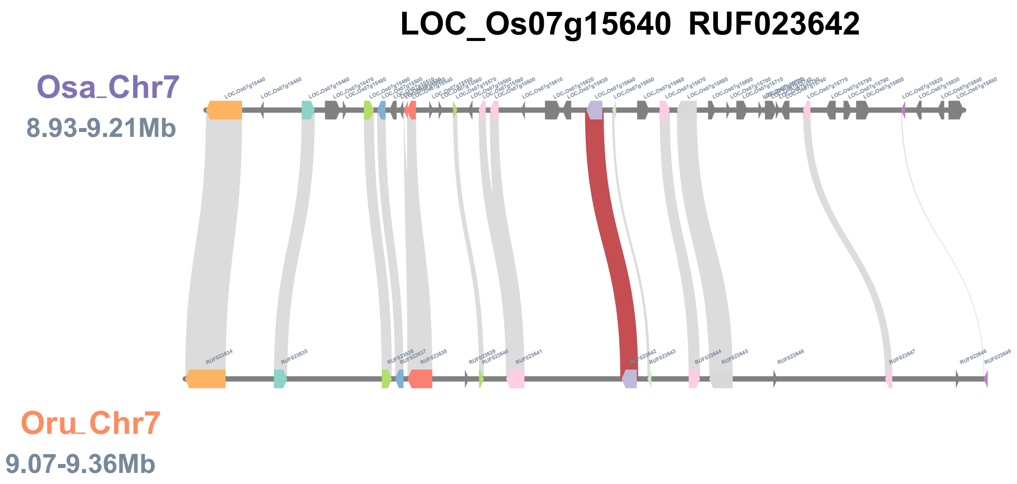
LOC_Os07g15640 RUF023642
Osa_Chr7
8.93-9.21Mb
Oru_Chr7
9.07-9.36Mb

LOC_Os07g20510 RUF025656

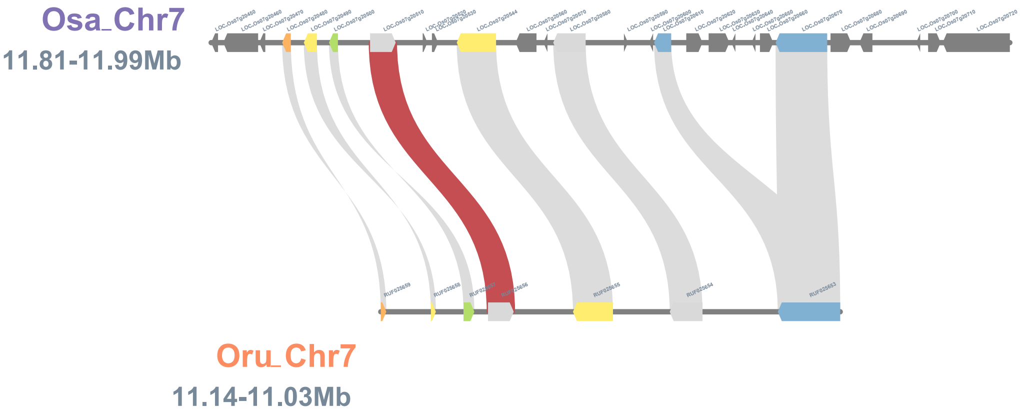

LOC_Os07g25500 RUF023918

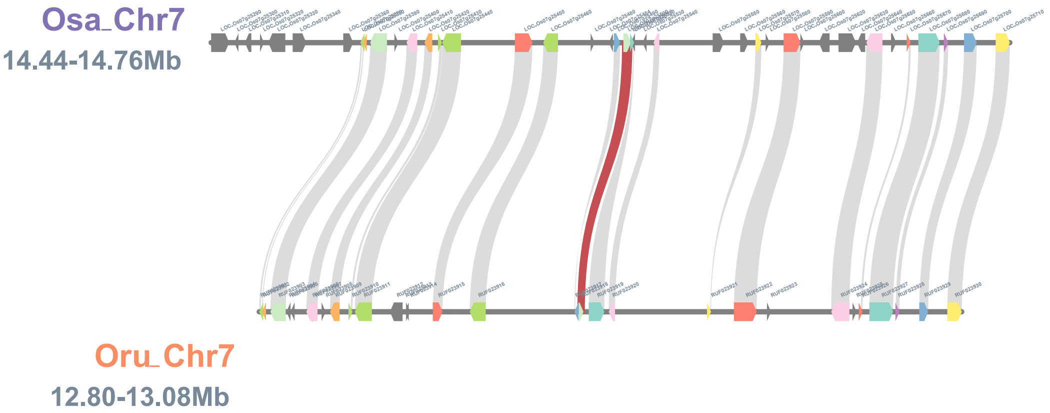

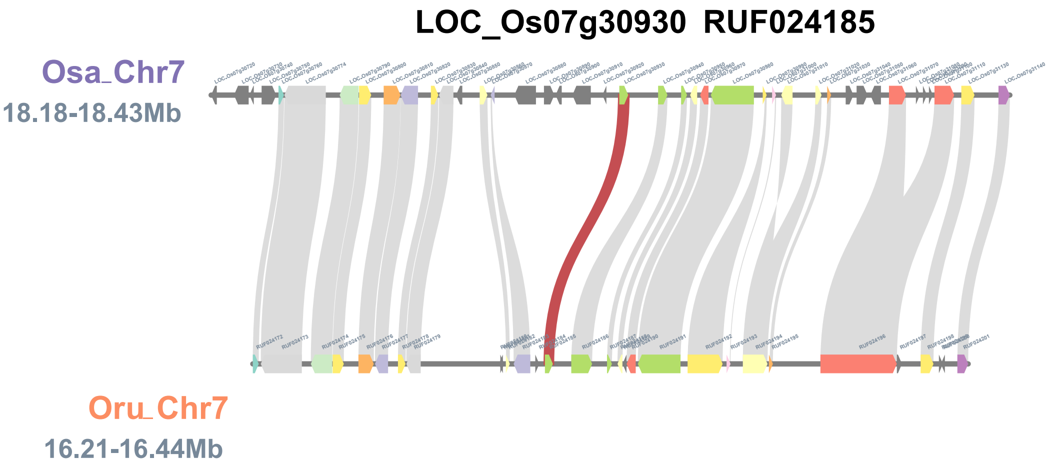
LOC_Os07g30930 RUF024185
Osa_Chr7
18.18-18.43Mb
Oru_Chr7
16.21-16.44Mb

LOC_Os07g32900 RUF024339

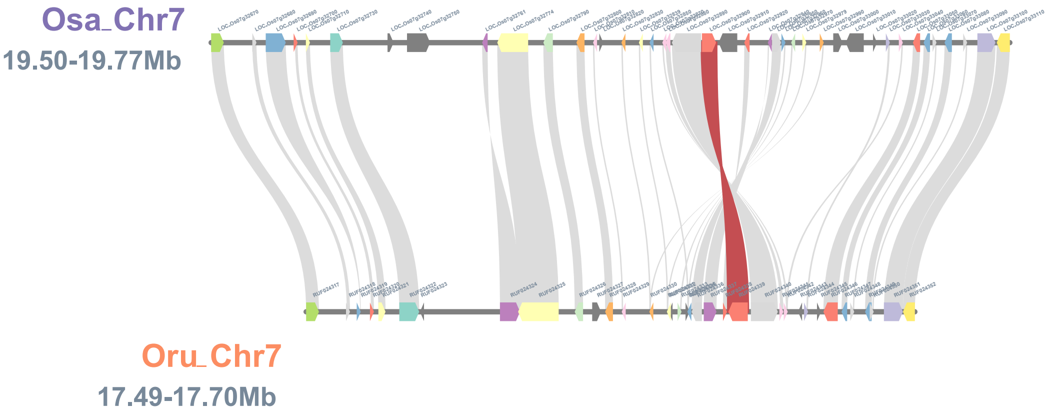

LOC_Os07g36390 RUF024596

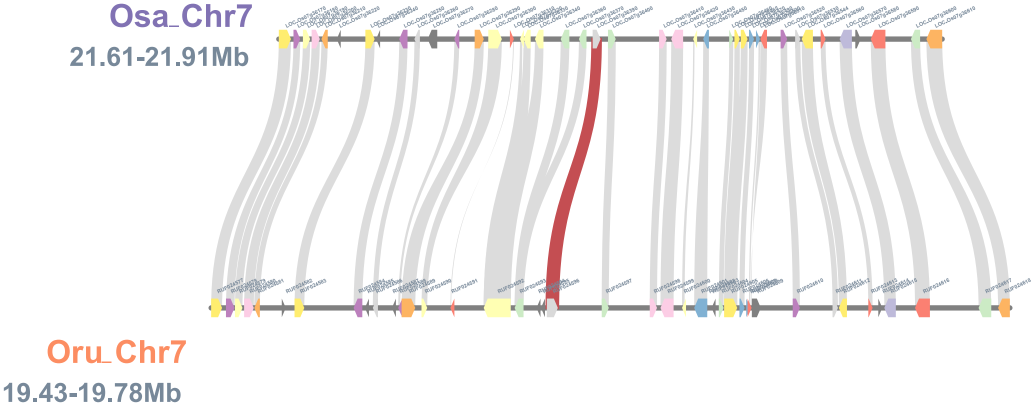

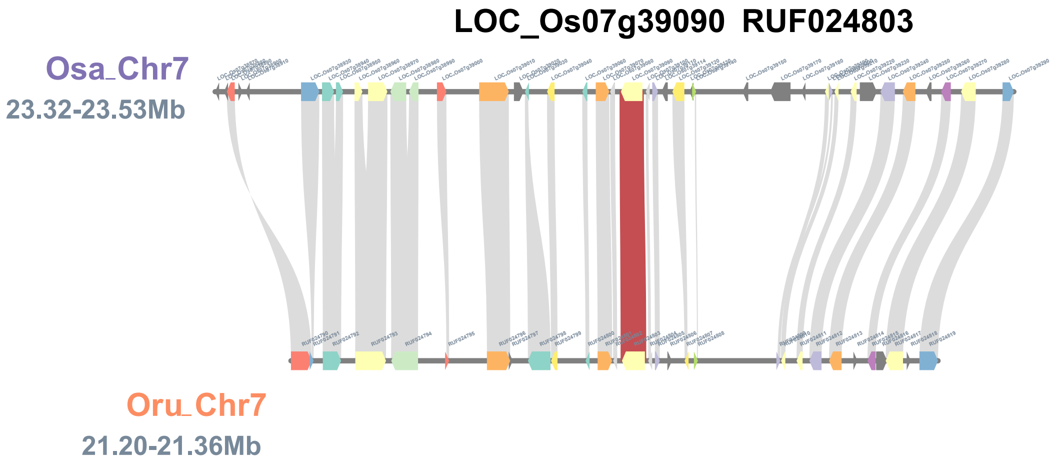
LOC_Os07g39090 RUF024803
Osa_Chr7
23.32-23.53Mb
Oru_Chr7
21.20-21.36Mb

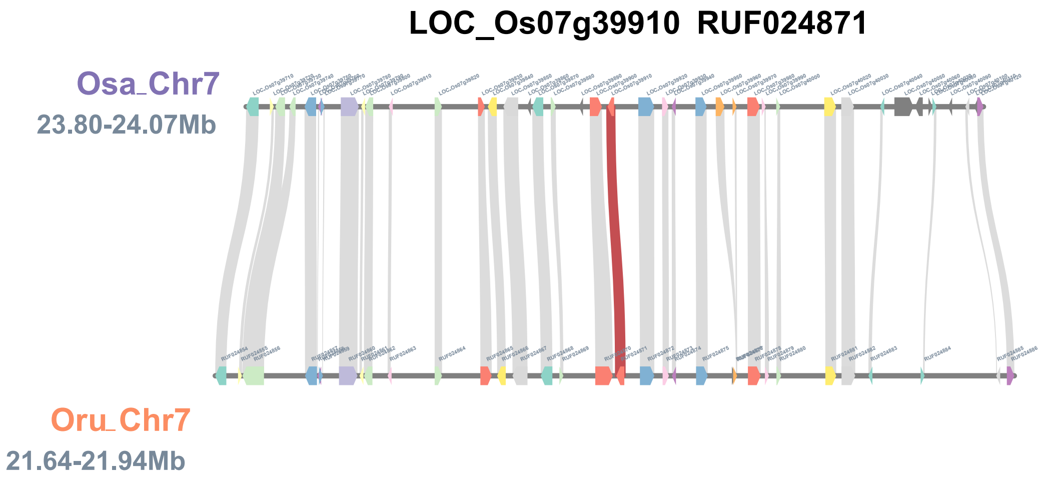
LOC_Os07g39910 RUF024871
Osa_Chr7
23.80-24.07Mb
Oru_Chr7
21.64-21.94Mb

LOC_Os07g40120 RUF024886

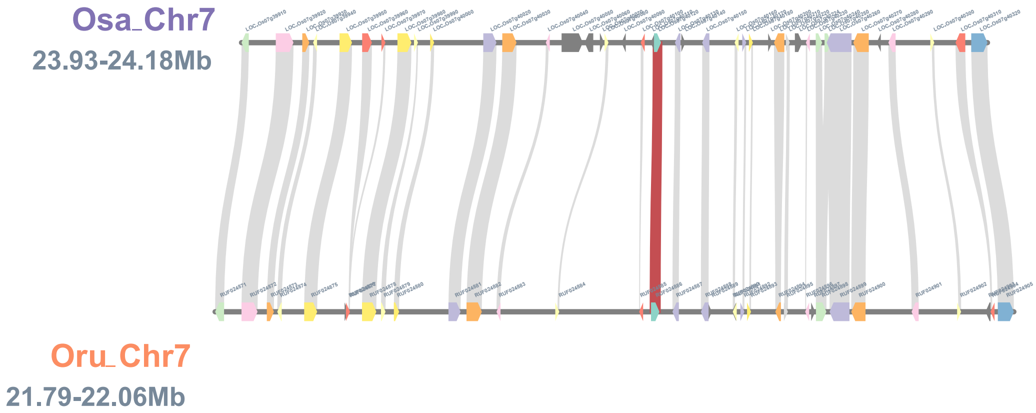

LOC_Os07g40750 RUF024935

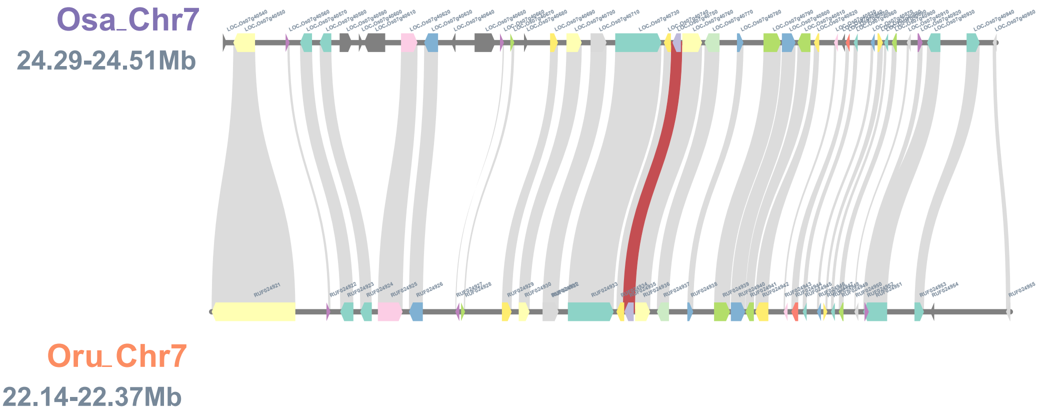

LOC_Os07g40800 RUF024940

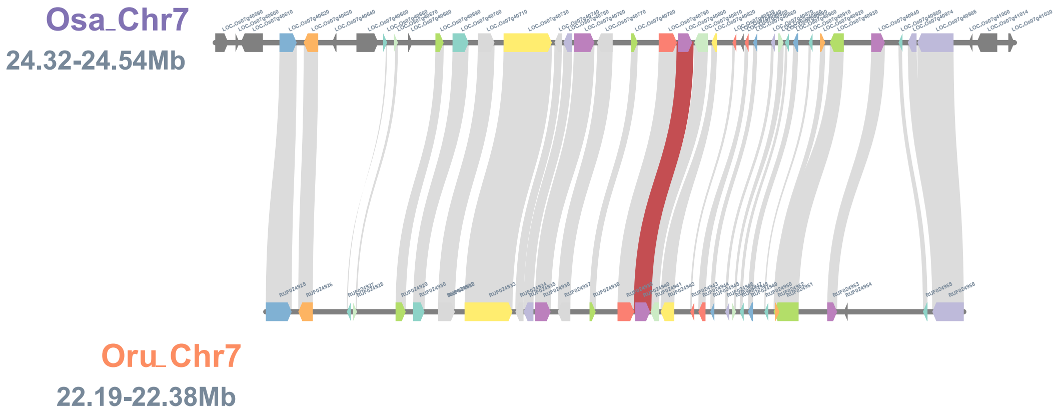

LOC_Os07g40820 RUF024942

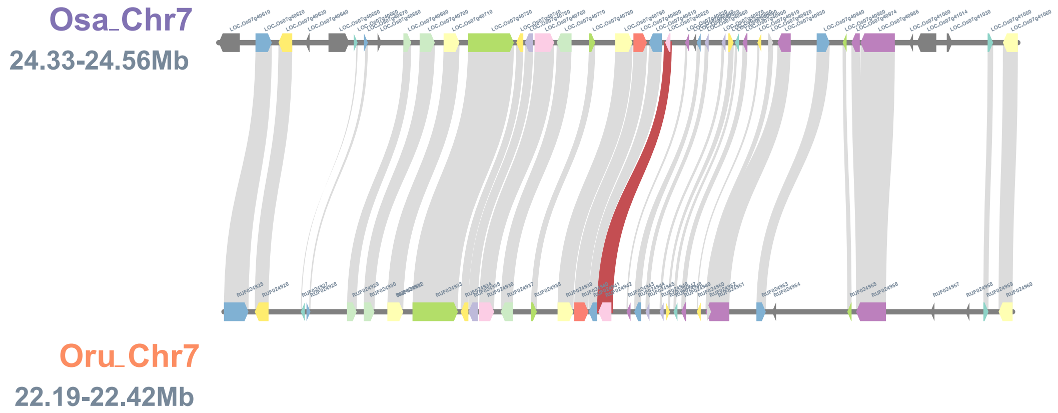

LOC_Os07g41260 RUF024978

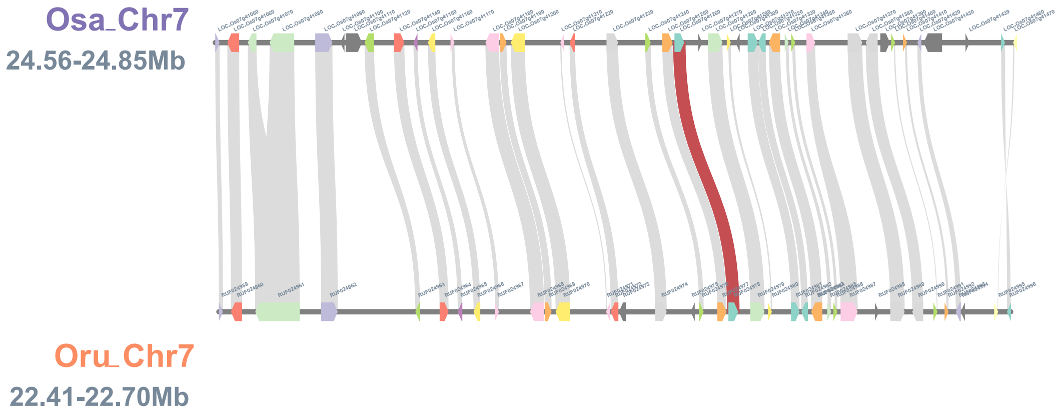

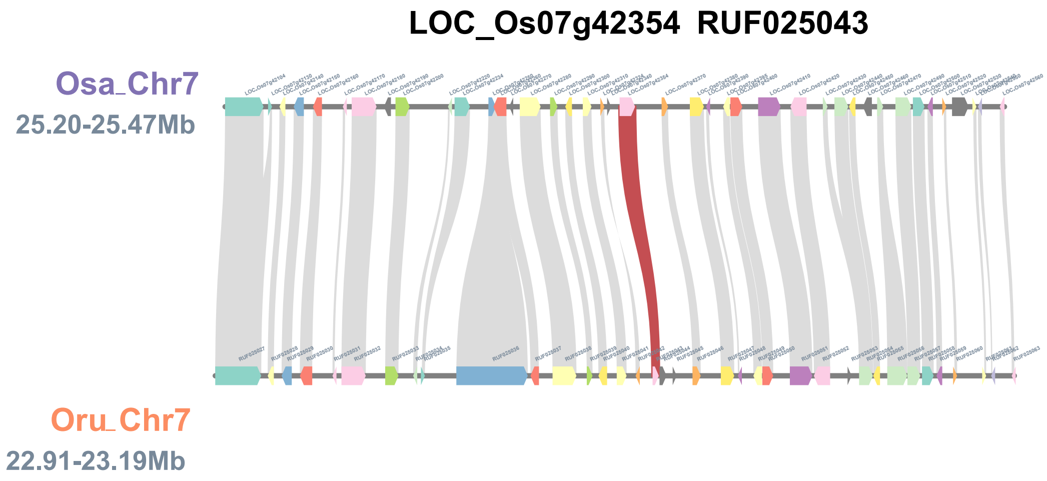
LOC_Os07g42354 RUF025043
Osa_Chr7
25.20-25.47Mb
Oru_Chr7
22.91-23.19Mb

LOC_Os07g42880 RUF025087

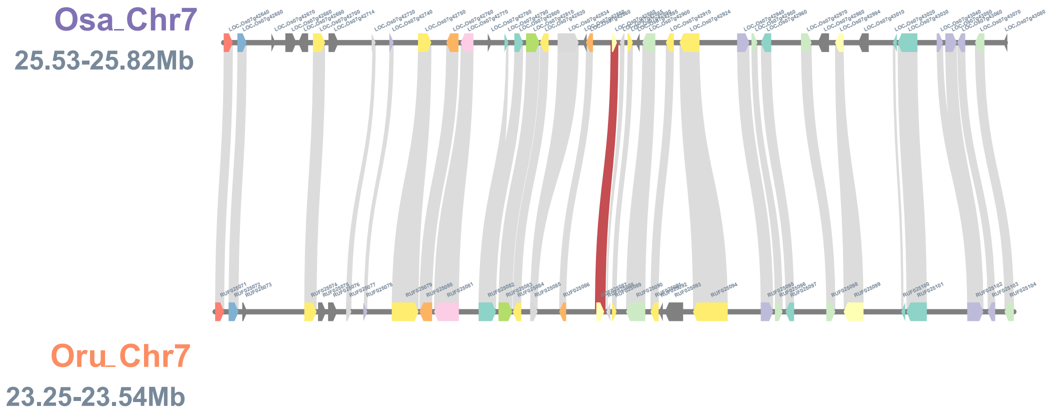

LOC_Os07g42880 RUF025087

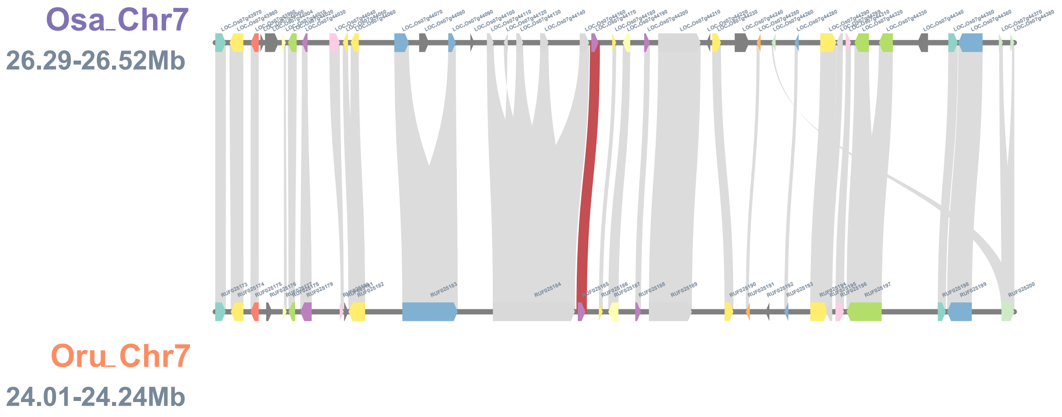

LOC_Os07g44299 RUF025195

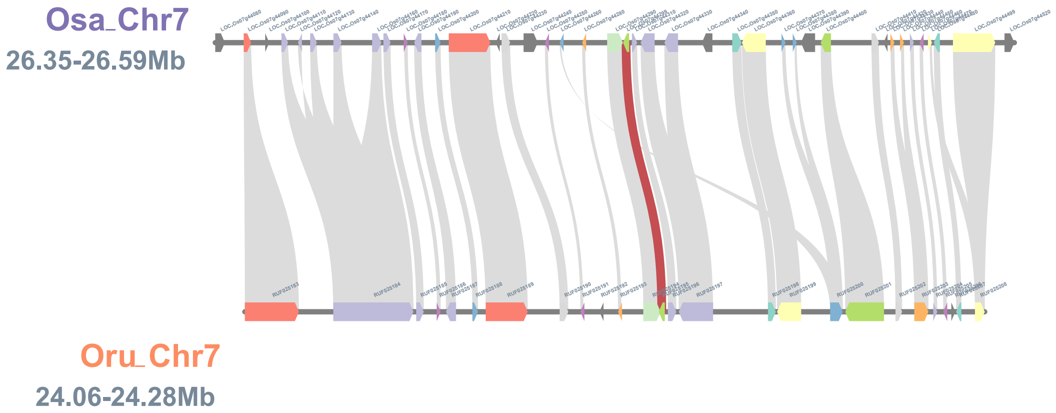

LOC_Os07g46730 RUF025381

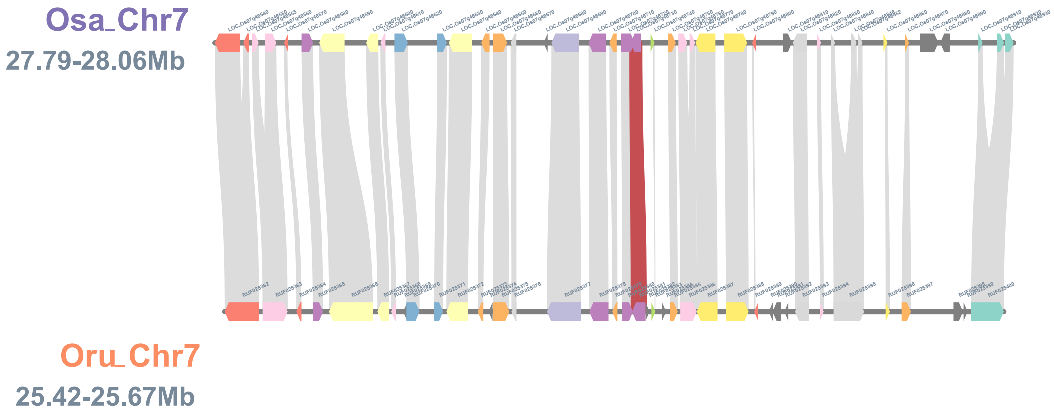

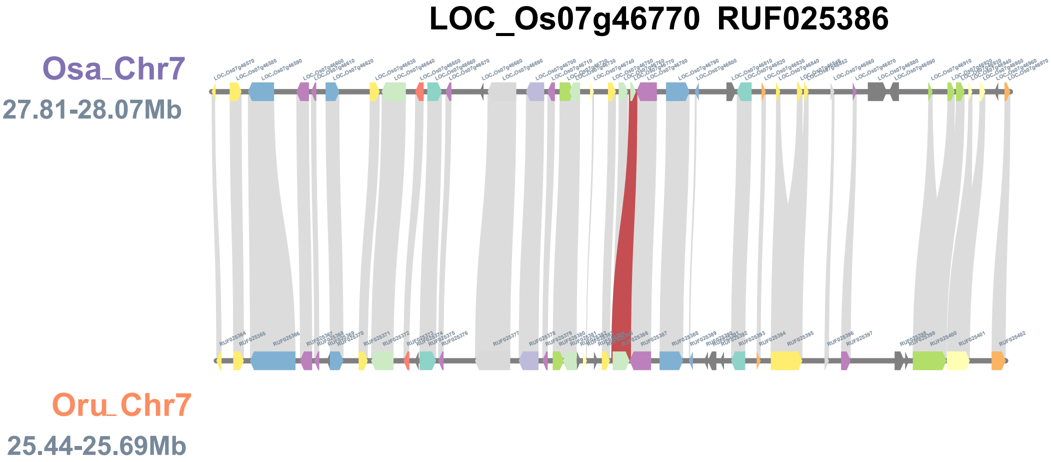
LOC_Os07g46770 RUF025386
Osa_Chr7
27.81-28.07Mb
Oru_Chr7
25.44-25.69Mb

LOC_Os07g47370 RUF025442

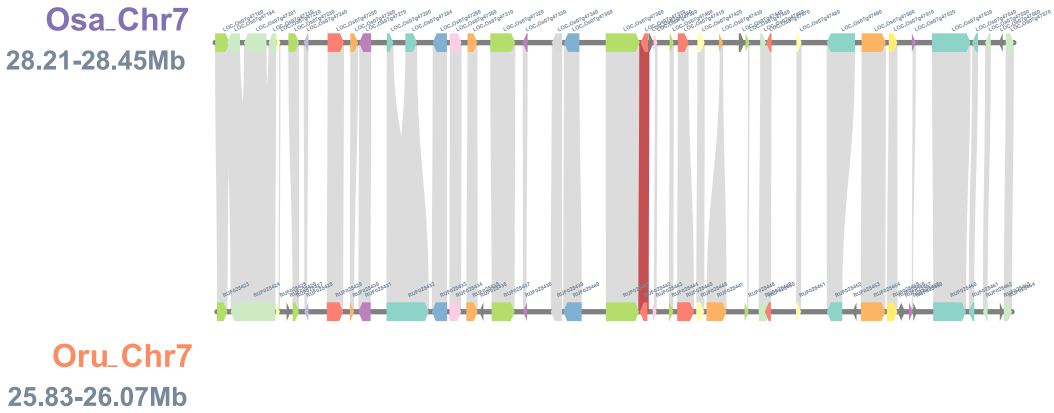

LOC_Os07g47470 RUF025450

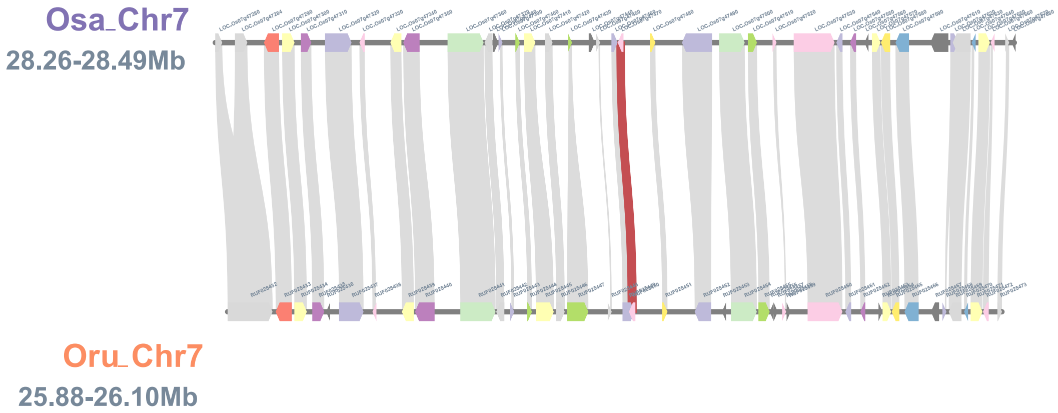

LOC_Os07g47720 RUF025476

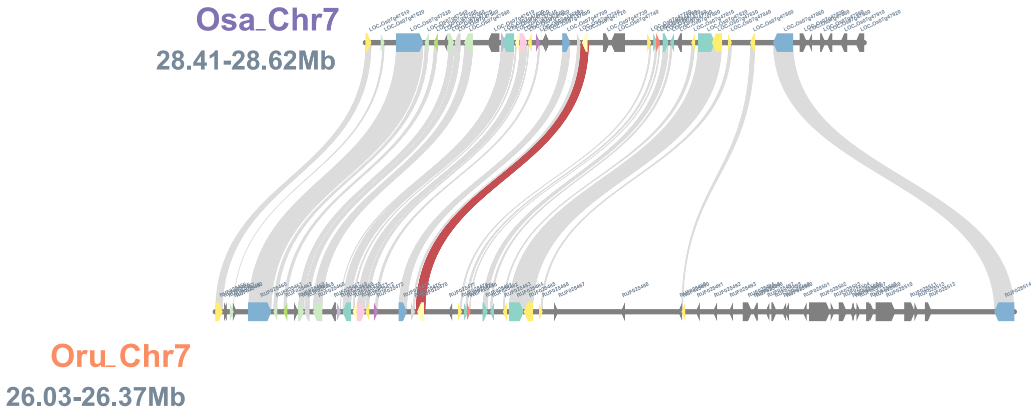

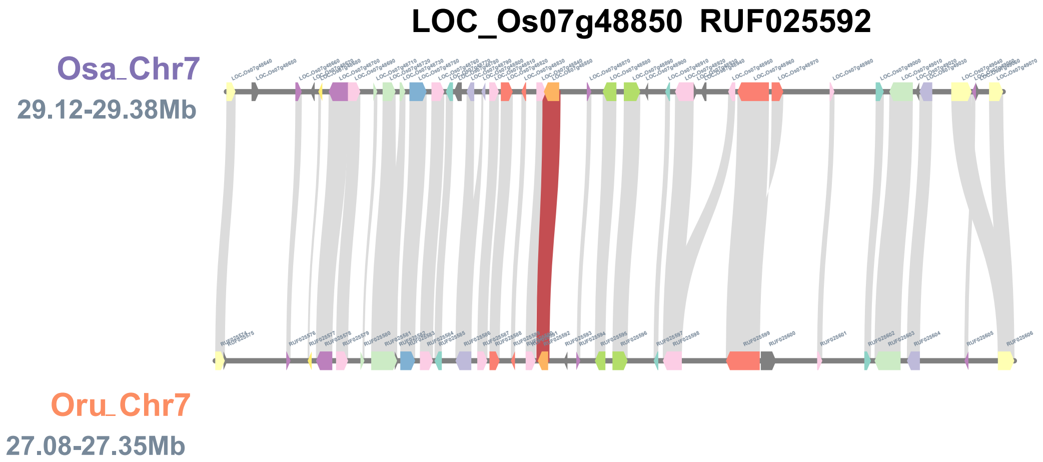
LOC_Os07g48850 RUF025592
Osa_Chr7
29.12-29.38Mb
Oru_Chr7
27.08-27.35Mb

LOC_Os08g01640 RUF025726

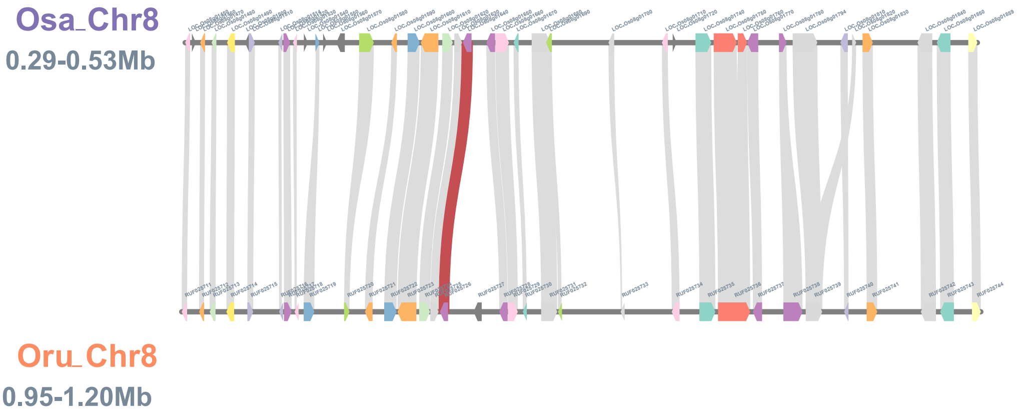

LOC_Os08g01650 RUF025728

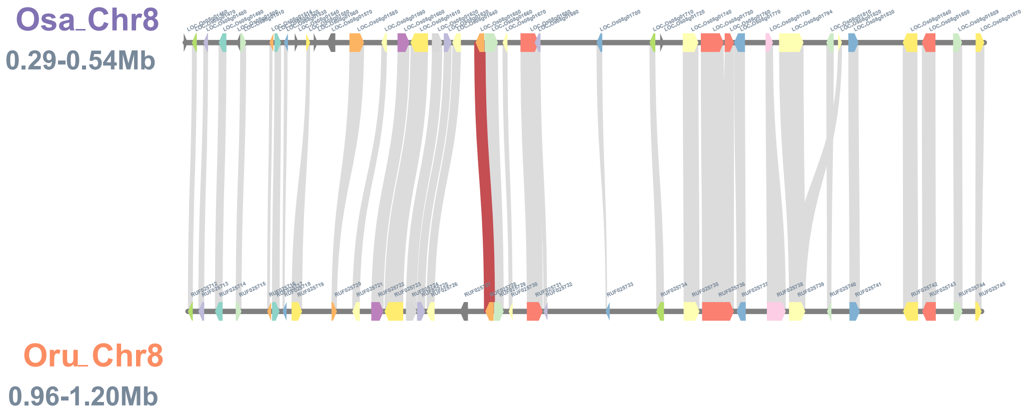

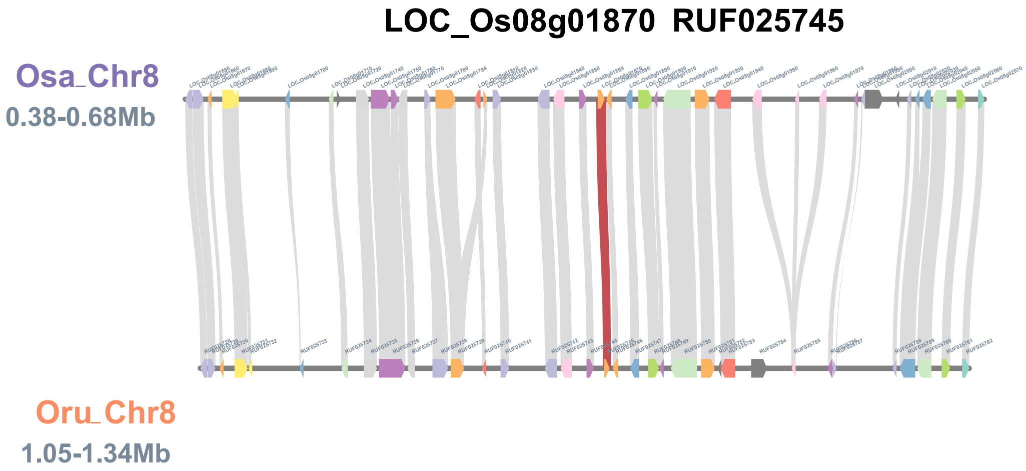
LOC_Os08g01870 RUF025745
Osa_Chr8
0.38-0.68Mb
Oru_Chr8
1.05-1.34Mb

LOC_Os08g02040 RUF025759

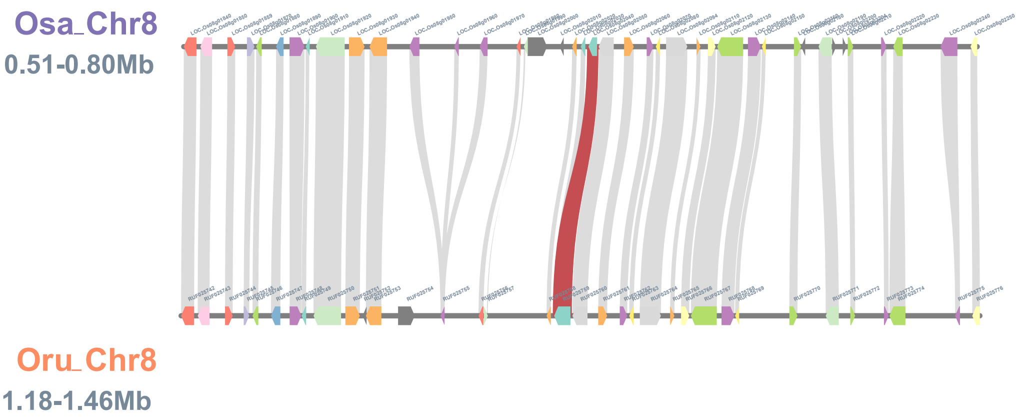

LOC_Os08g03676 RUF025885

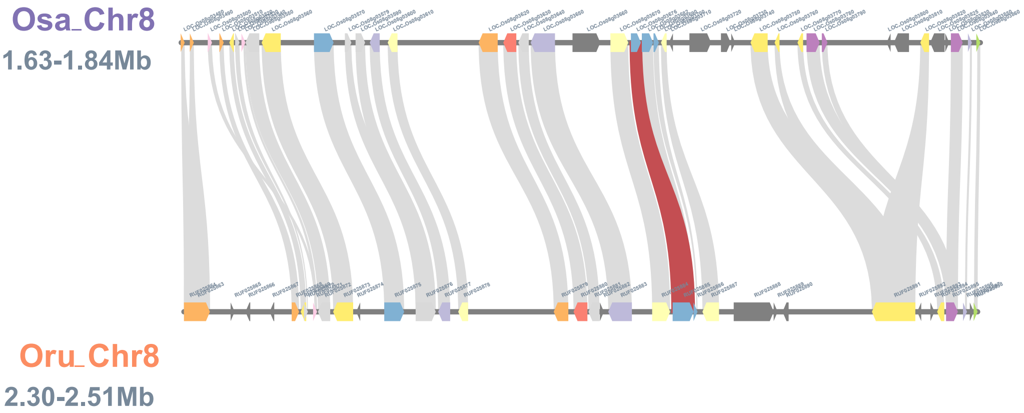

LOC_Os08g04400 RUF025928

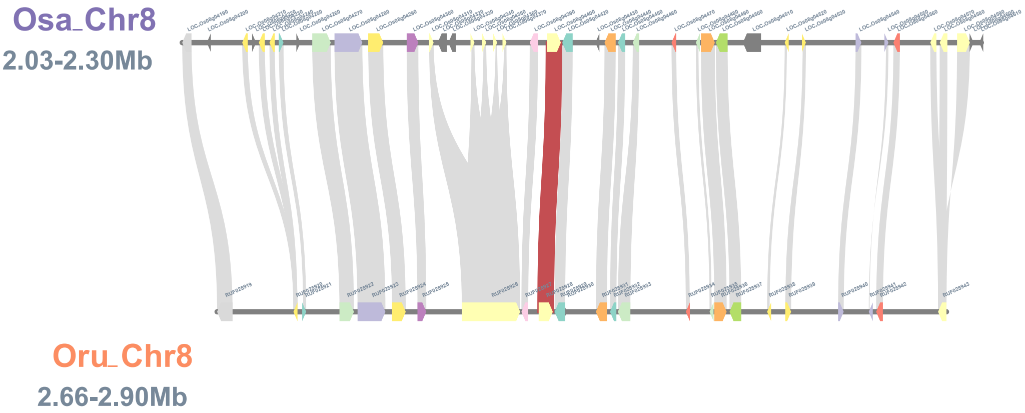

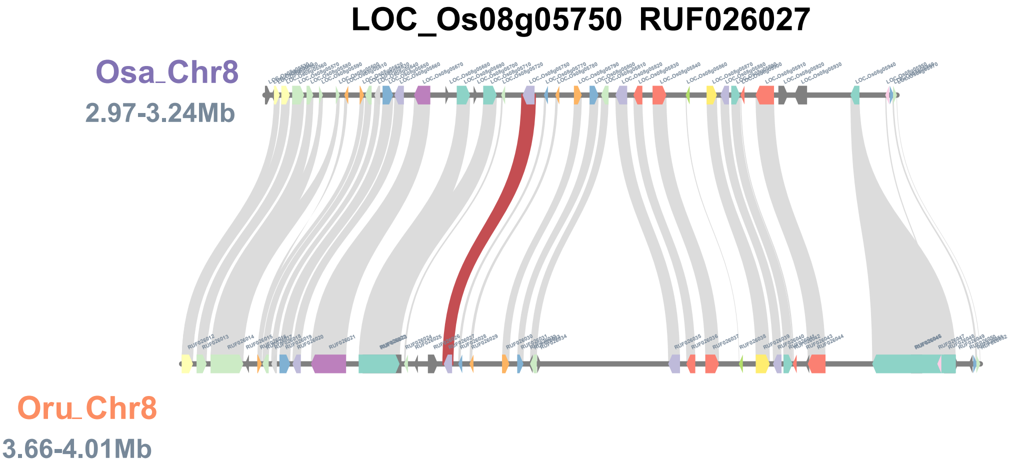
LOC_Os08g05750 RUF026027
Osa_Chr8
2.97-3.24Mb
Oru_Chr8
3.66-4.01Mb

LOC_Os08g06490 RUF026101

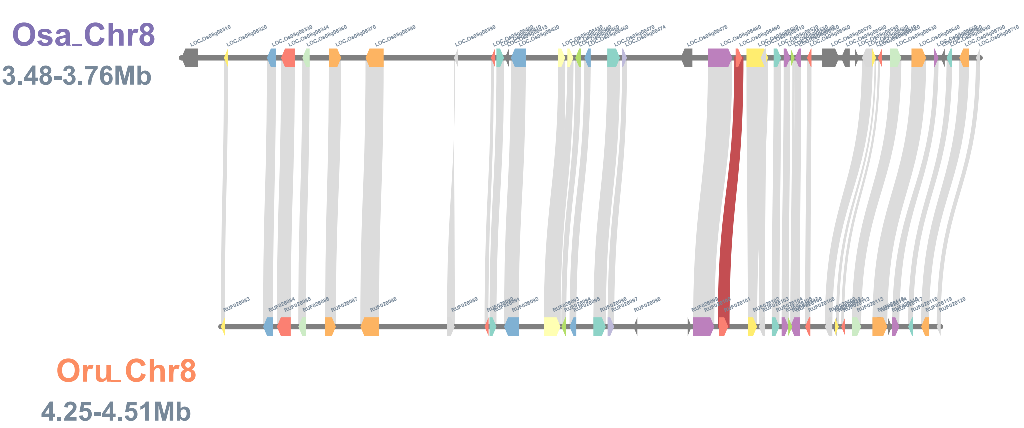

LOC_Os08g06500 RUF026102

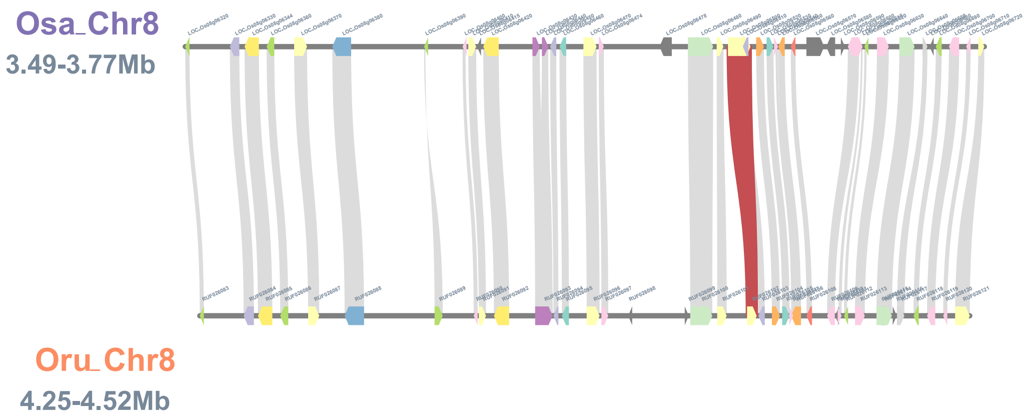

LOC_Os08g09270 RUF026277

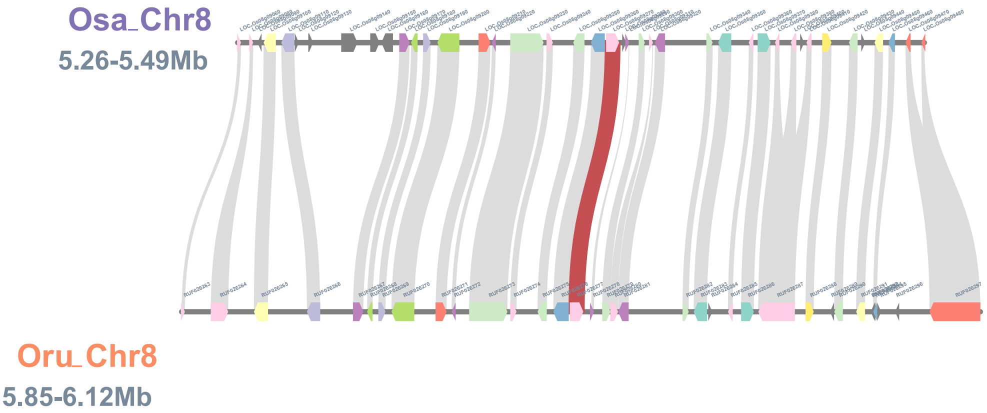

LOC_Os08g15090 RUF026566

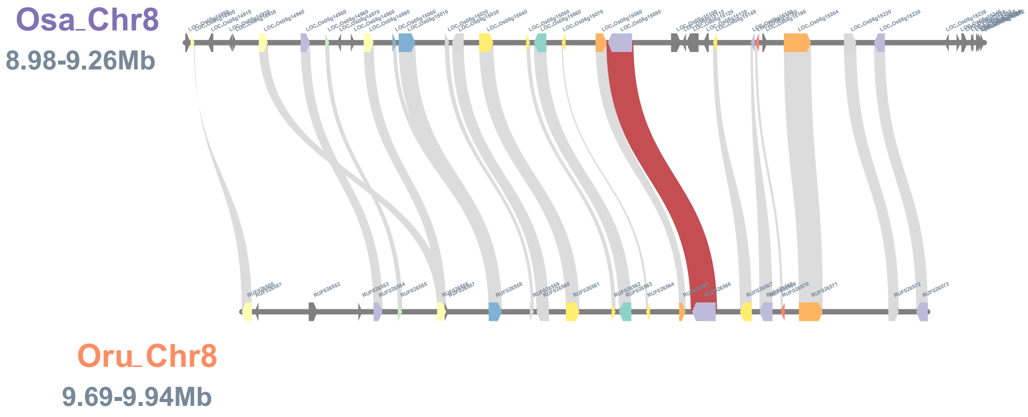

LOC_Os08g17080 RUF026651

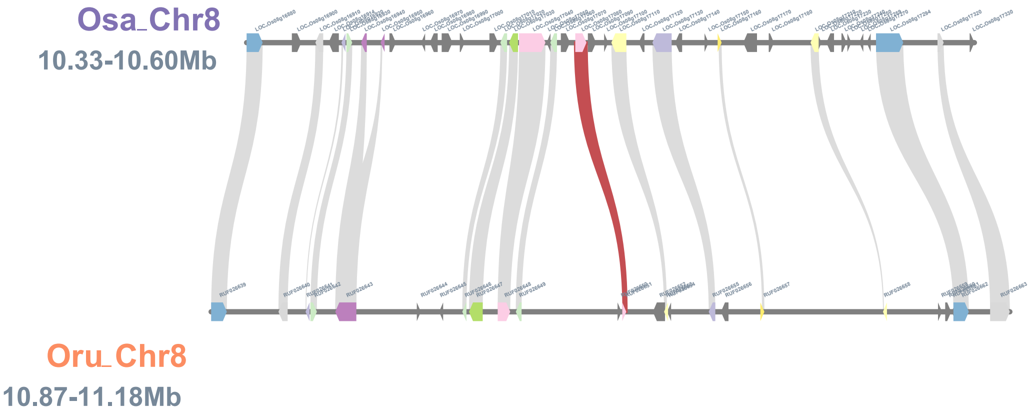

LOC_Os08g20610 RUF026749

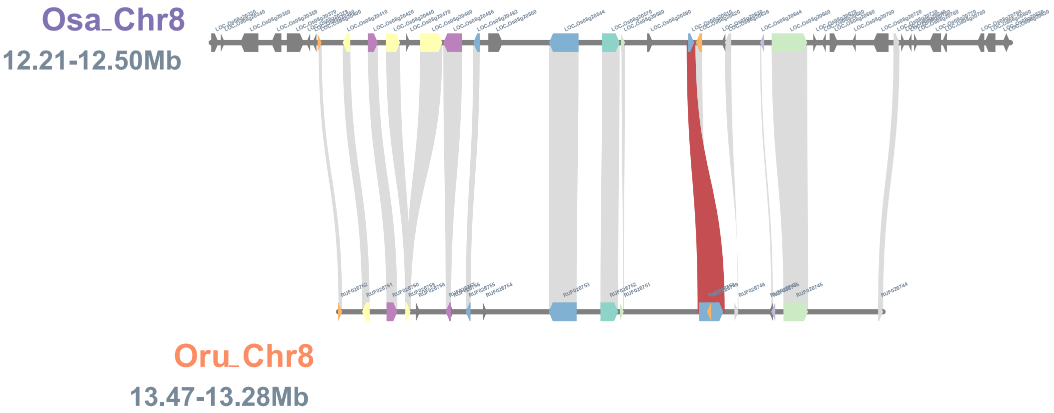

LOC_Os08g21561 RUF026790

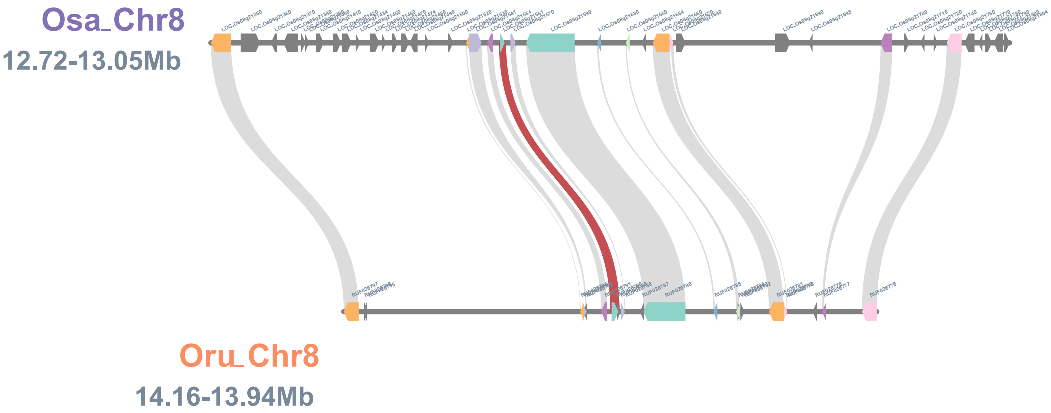

LOC_Os08g25280 RUF026936

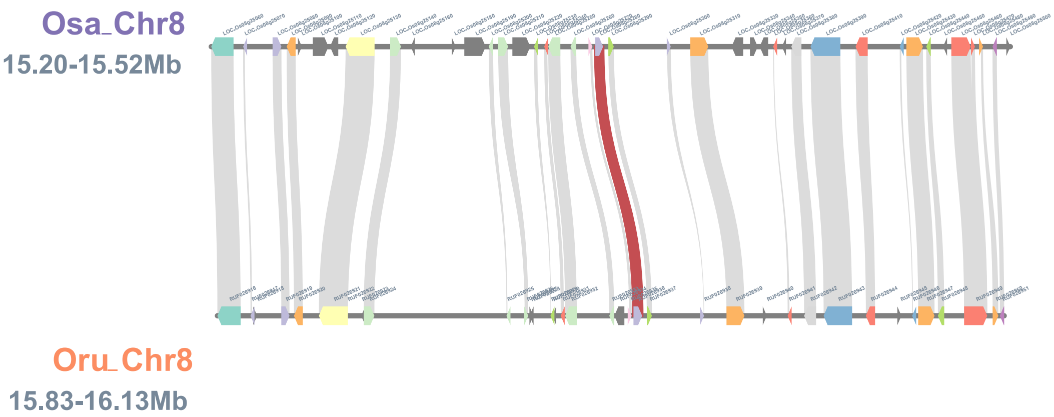

LOC_Os08g28180 RUF027097

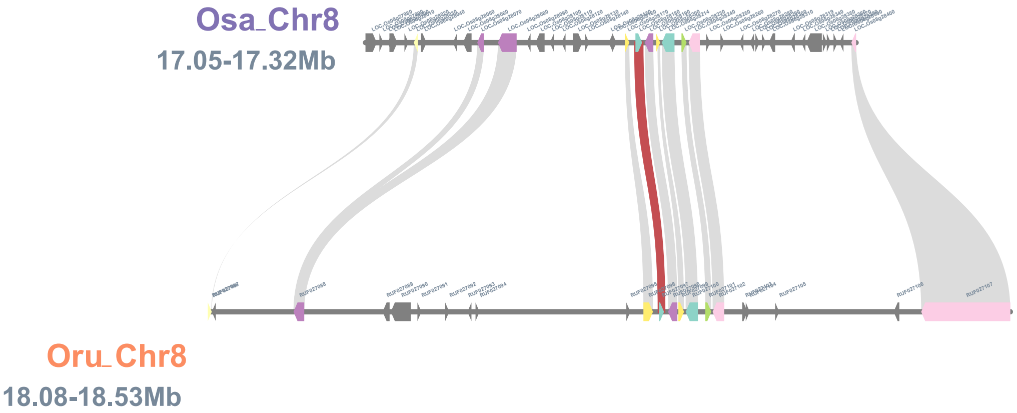

LOC_Os08g28830 RUF027124

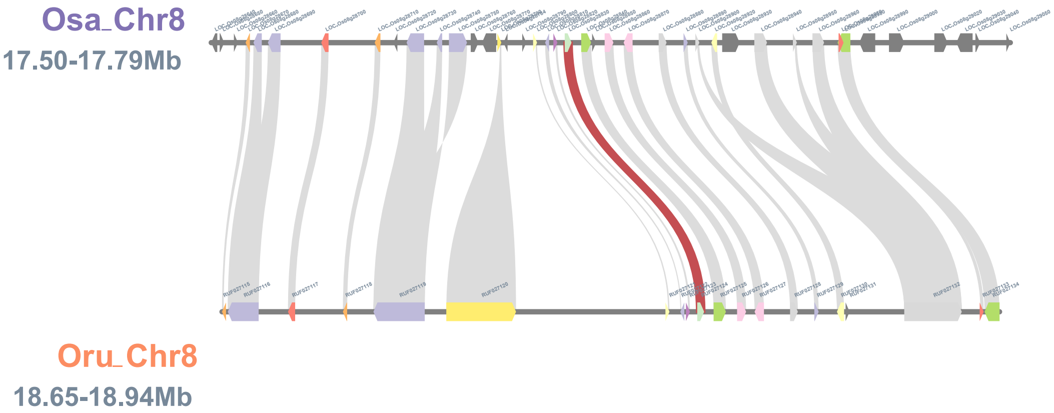

LOC_Os08g31110 RUF027240

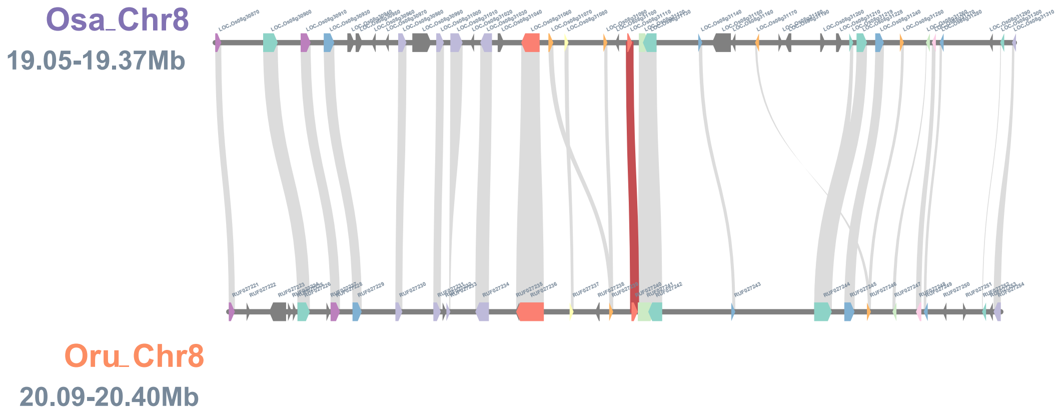

LOC_Os08g33700 RUF027415

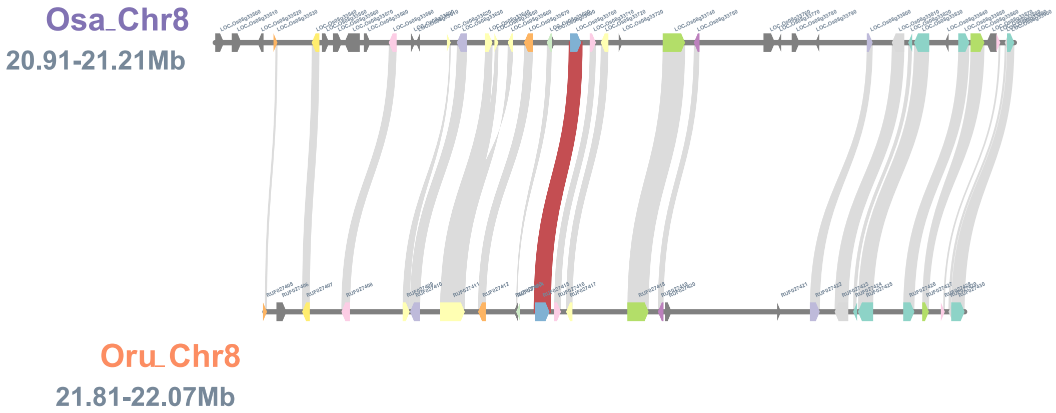

LOC_Os08g37520 RUF027703

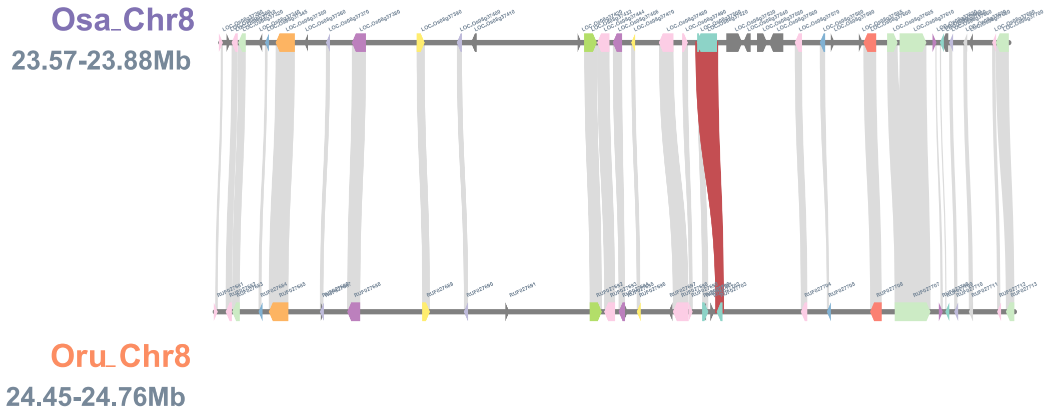

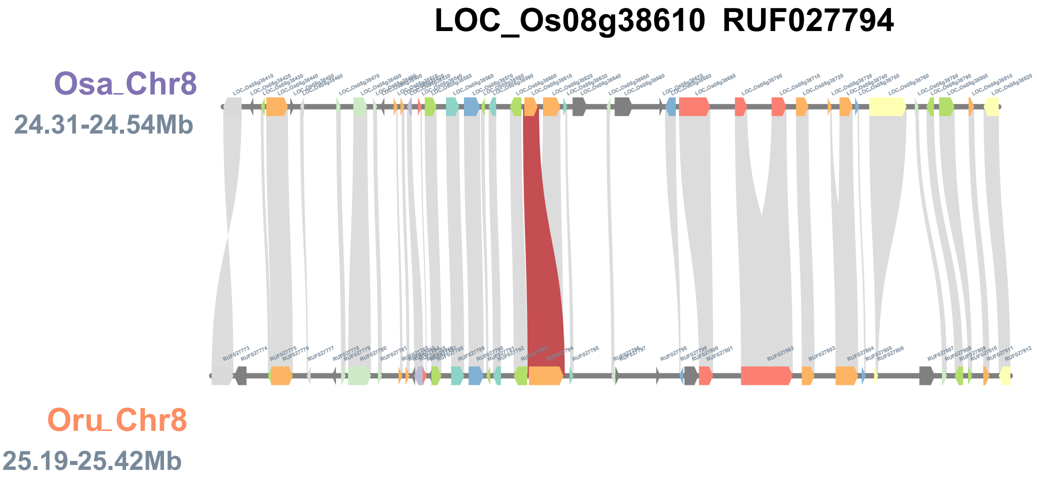
LOC_Os08g38610 RUF027794
Osa_Chr8
24.31-24.54Mb
Oru_Chr8
25.19-25.42Mb

LOC_Os08g39050 RUF027834

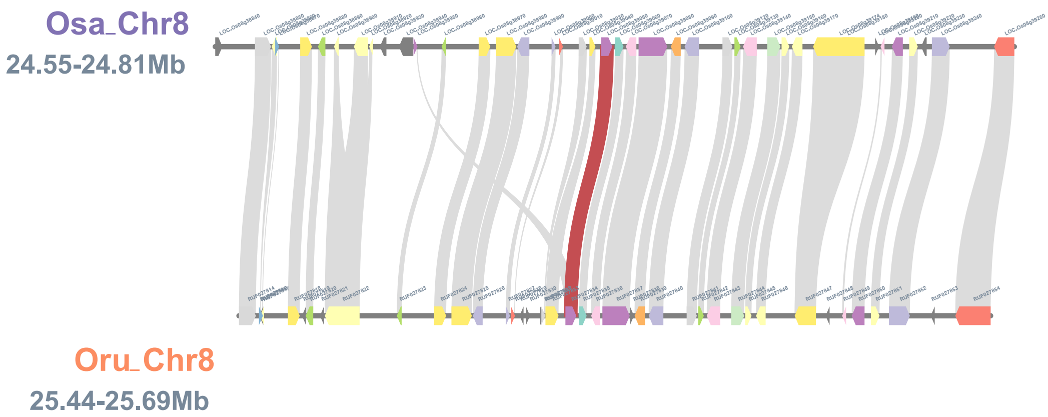

LOC_Os08g39130 RUF027842

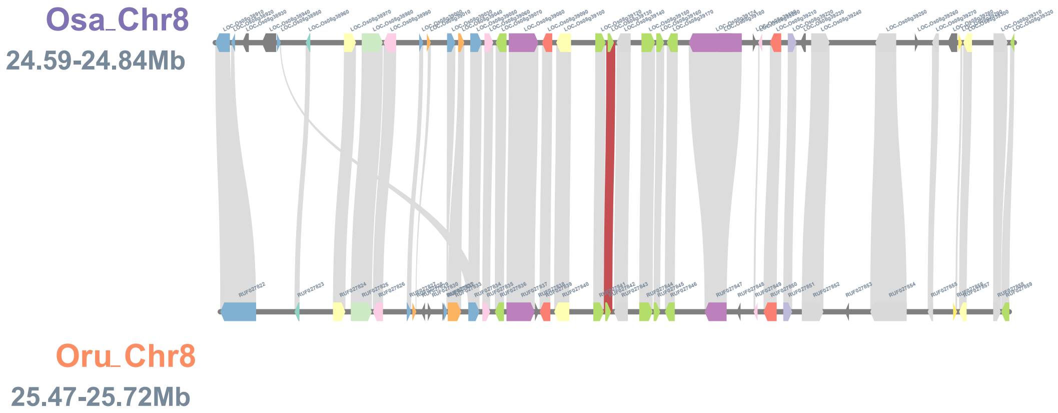

LOC_Os08g40020 RUF027911

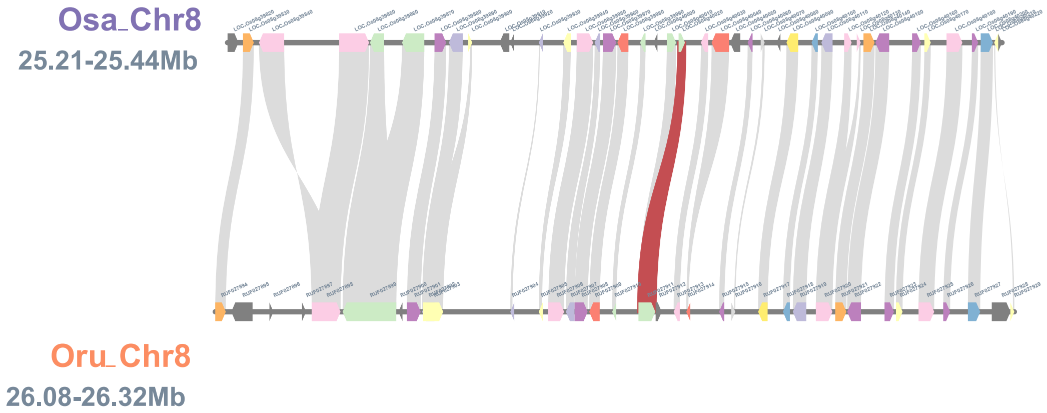

LOC_Os08g41380 RUF028025

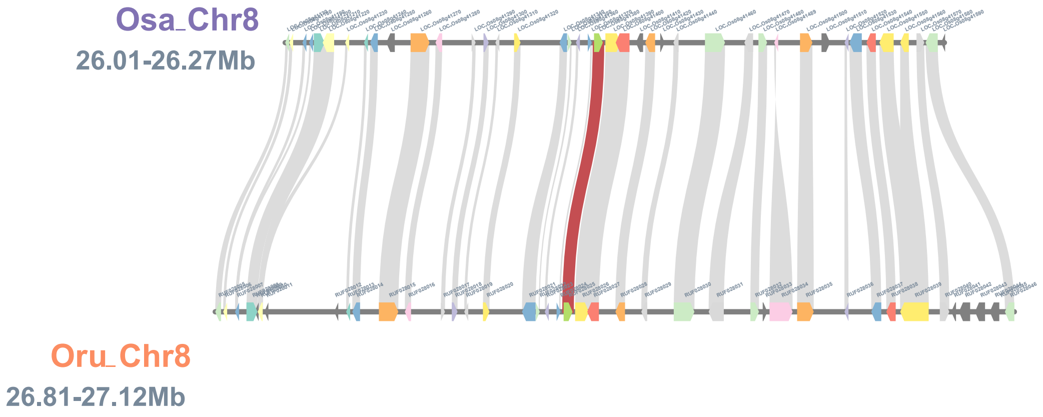

LOC_Os08g42610 RUF028119

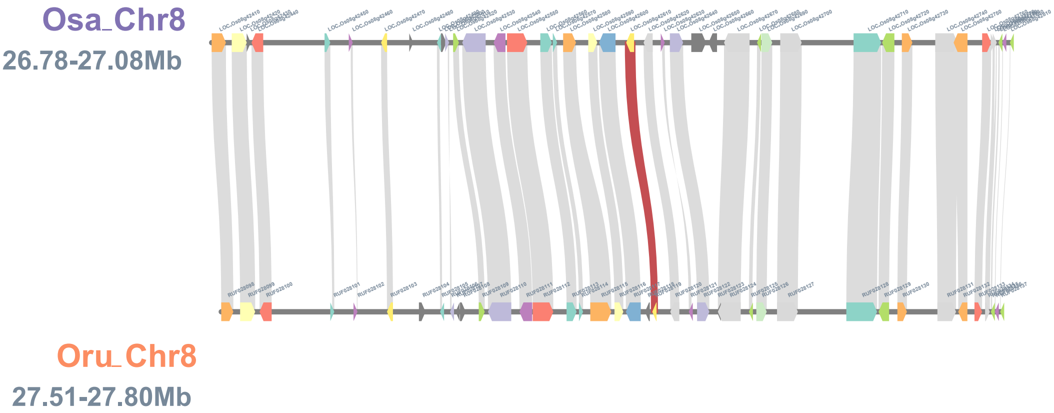

LOC_Os09g02260 RUF028386

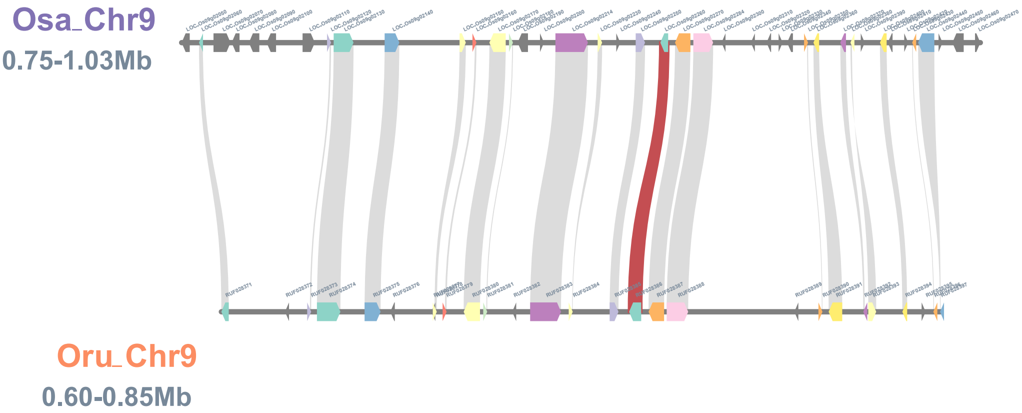

LOC_Os09g07780 RUF028549

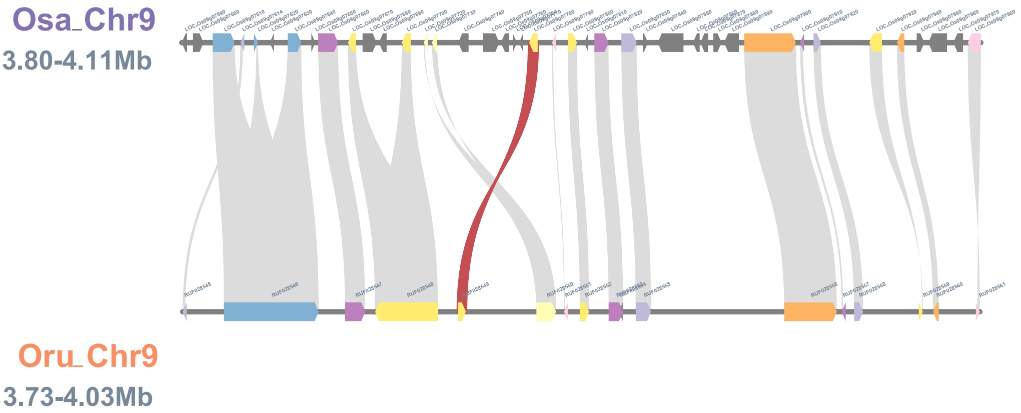

LOC_Os09g15800 RUF028908

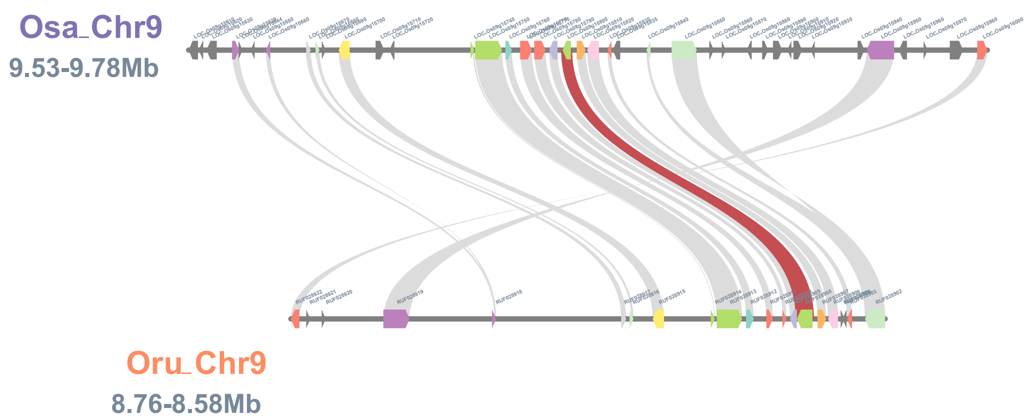

LOC_Os09g24240 RUF029323

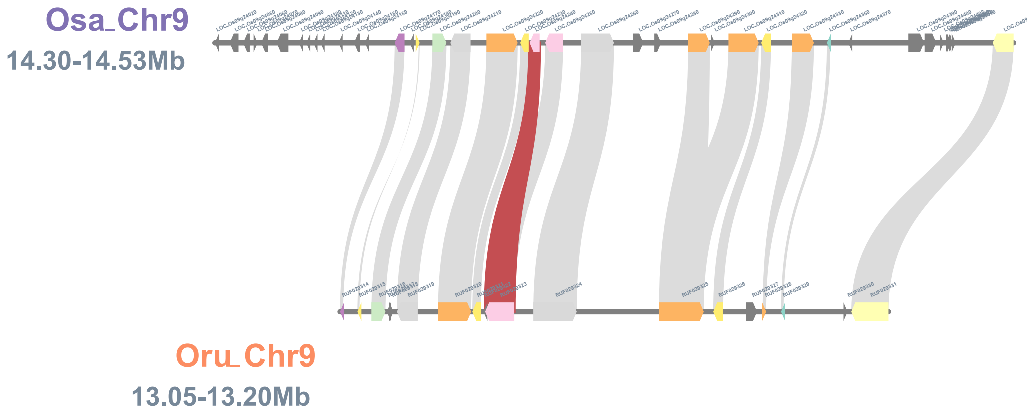

LOC_Os09g24520 RUF029340

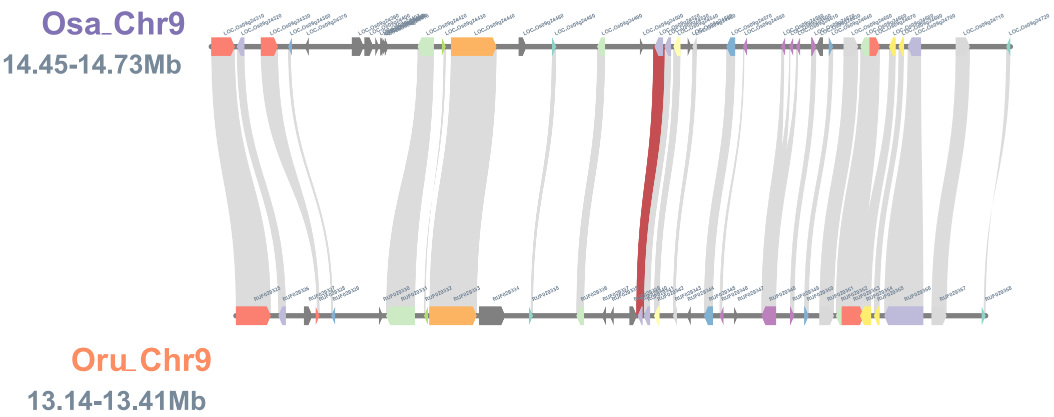

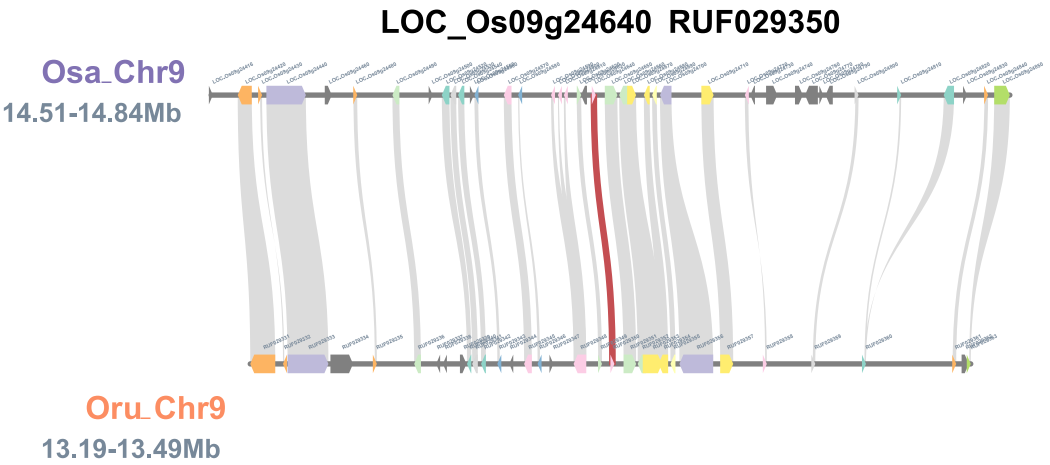

LOC_Os09g24640 RUF029350
Osa_Chr9
14.51-14.84Mb
Oru_Chr9
13.19-13.49Mb

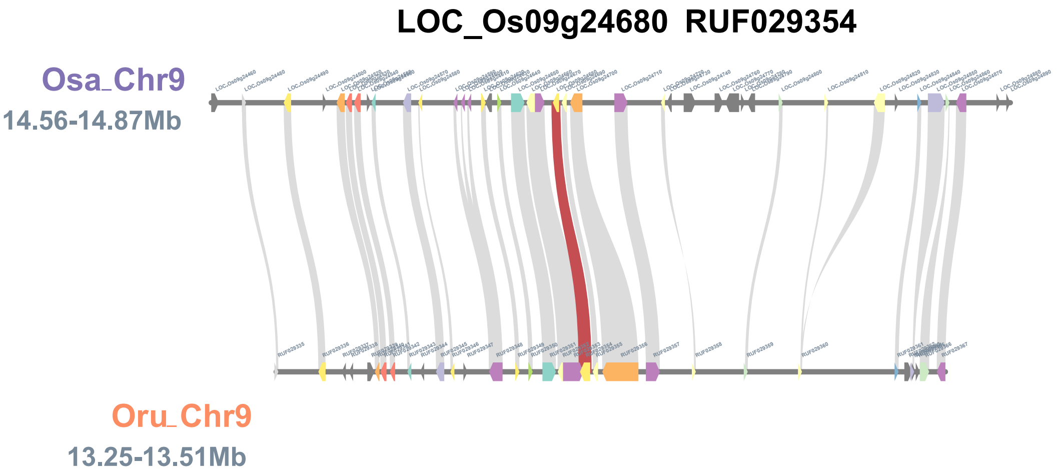
LOC_Os09g24680 RUF029354
Osa_Chr9
14.56-14.87Mb
Oru_Chr9
13.25-13.51Mb

LOC_Os09g25050 RUF029384

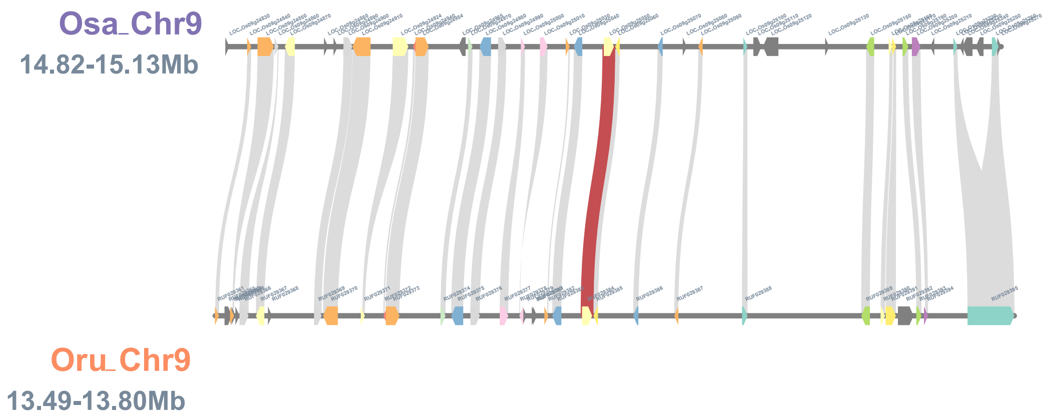

LOC_Os09g25550 RUF029413

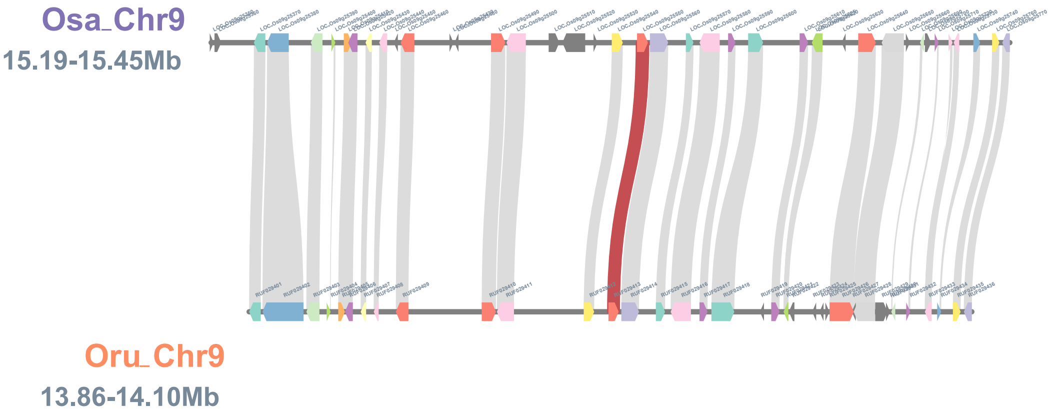

LOC_Os09g26190 RUF029470

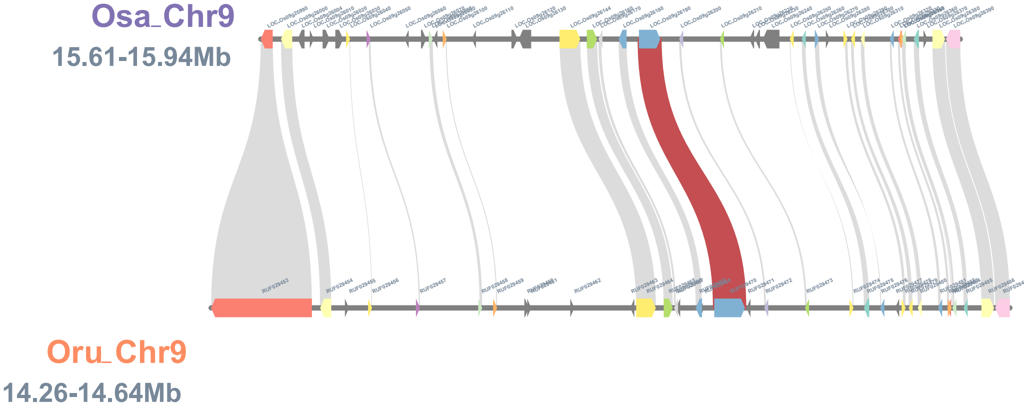

LOC_Os09g29790 RUF029759

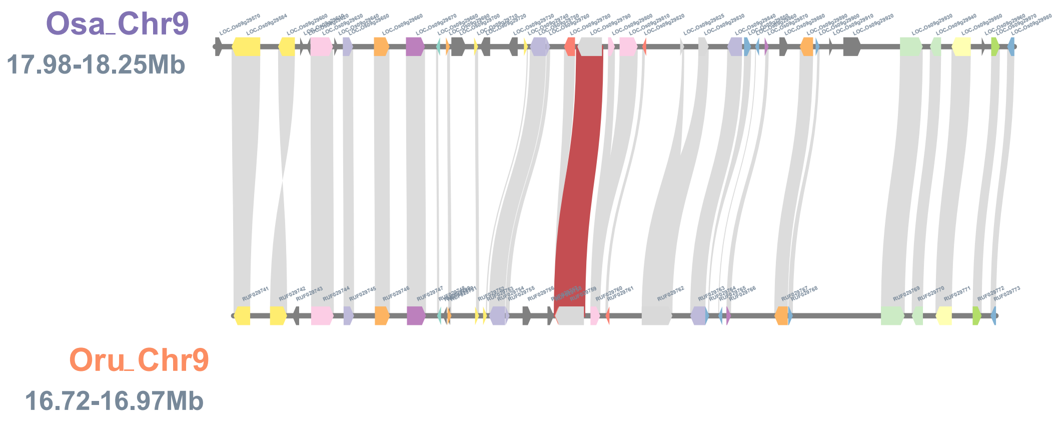

LOC_Os09g34870 RUF030031

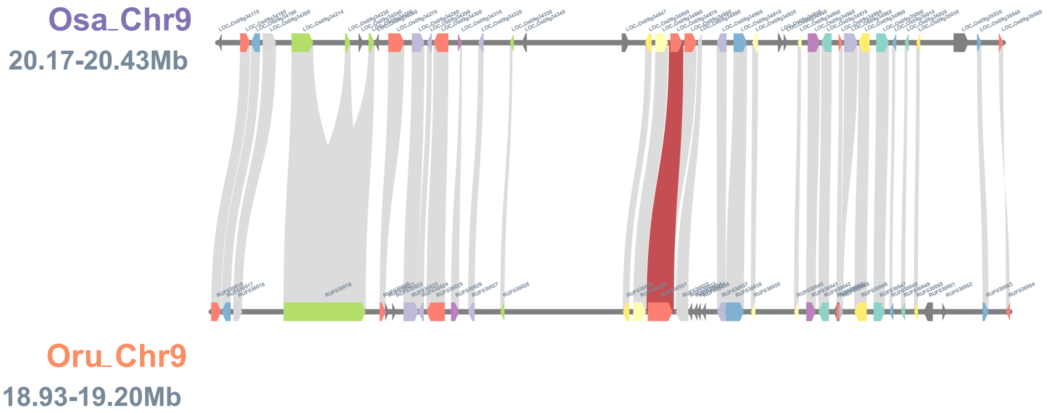

LOC_Os09g37070 RUF030179

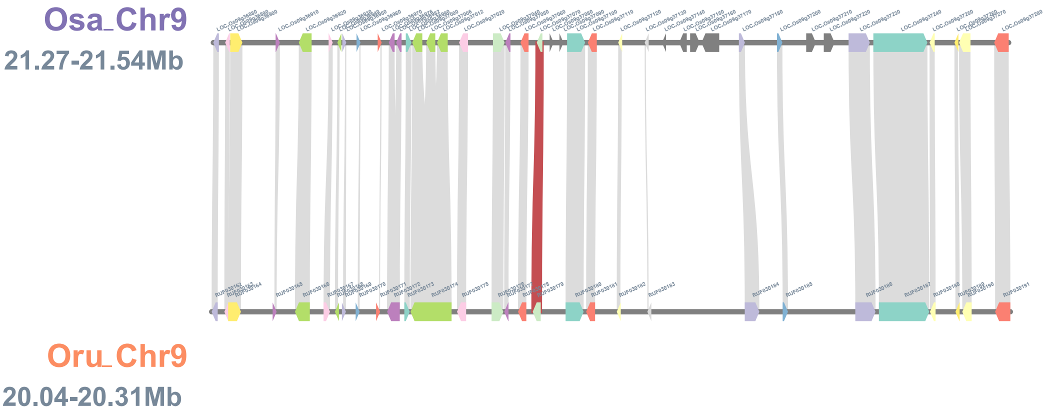

LOC_Os09g38310 RUF030281

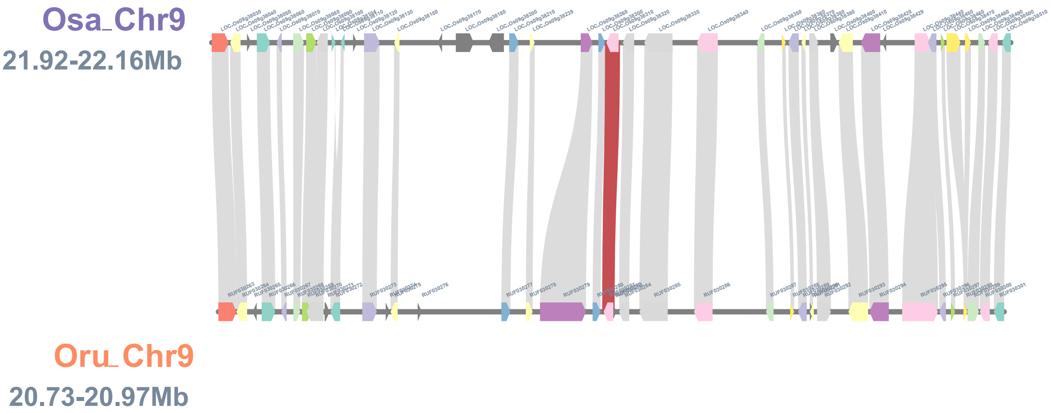

LOC_Os09g39970 RUF030412

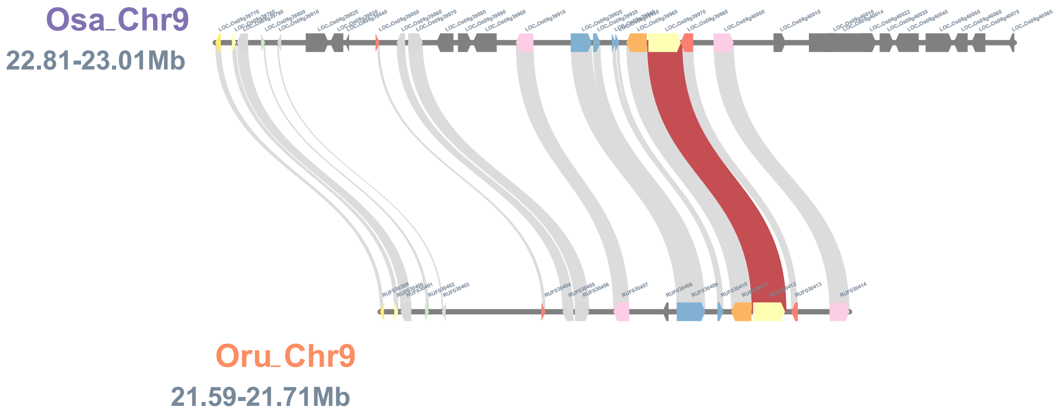

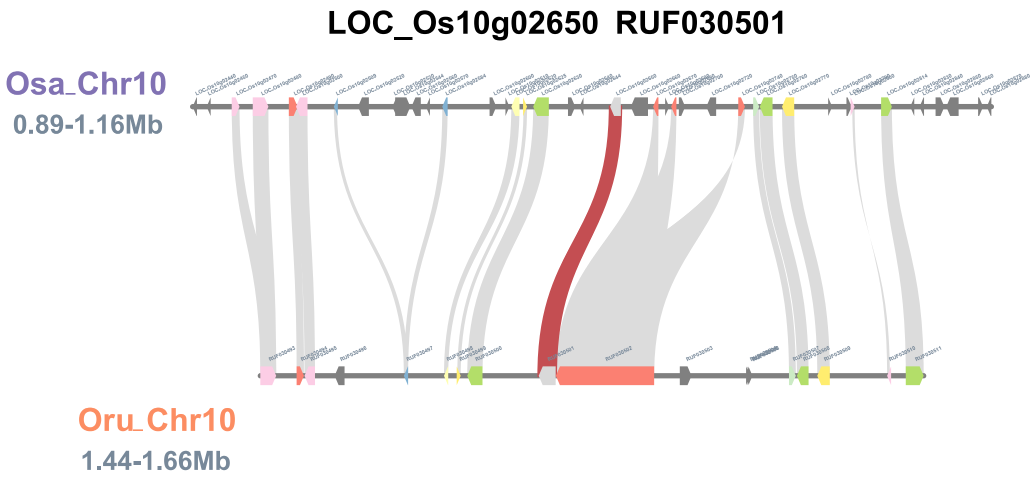
LOC_Os10g02650 RUF030501
Osa_Chr10
0.89-1.16Mb
Oru_Chr10
1.44-1.66Mb

LOC_Os10g10170 RUF030929

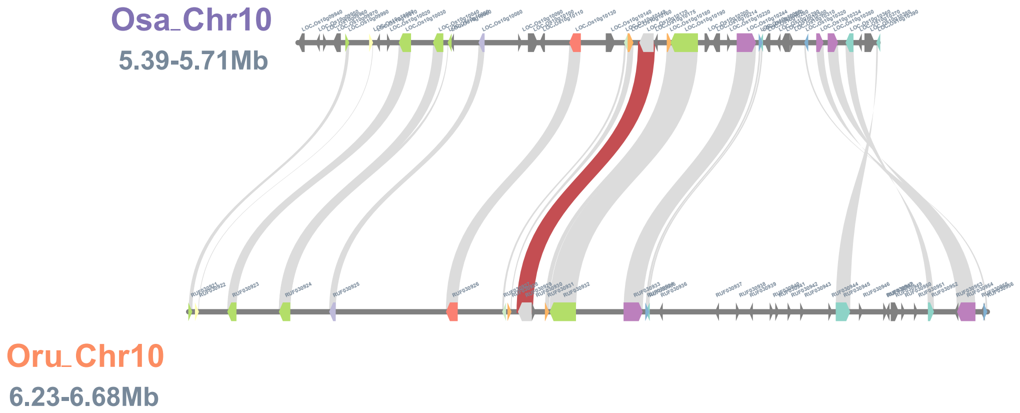

LOC_Os10g21470 RUF031294

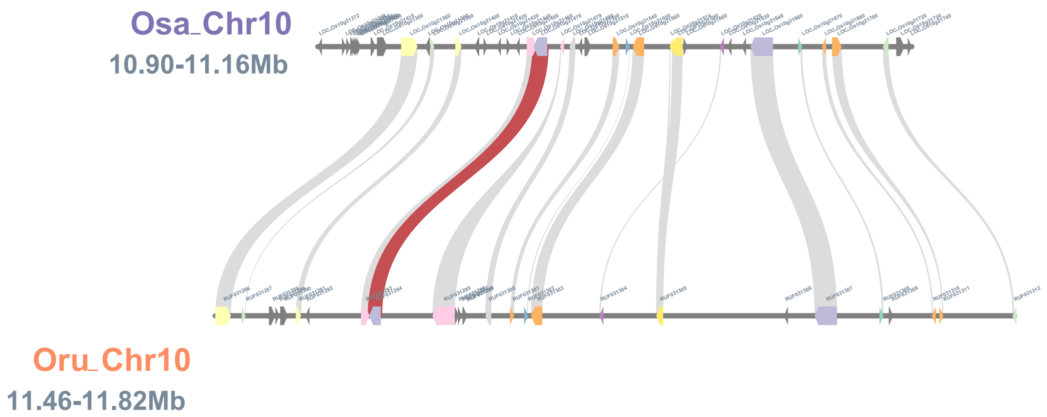

LOC_Os10g21920 RUF031321

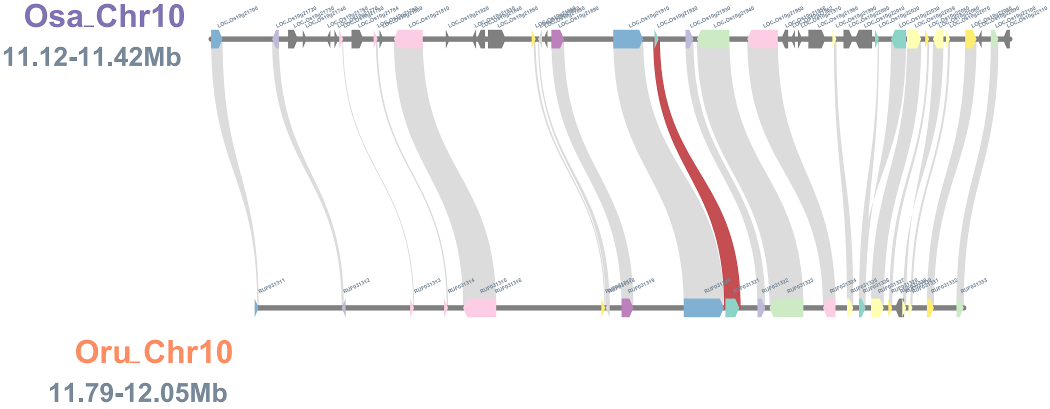

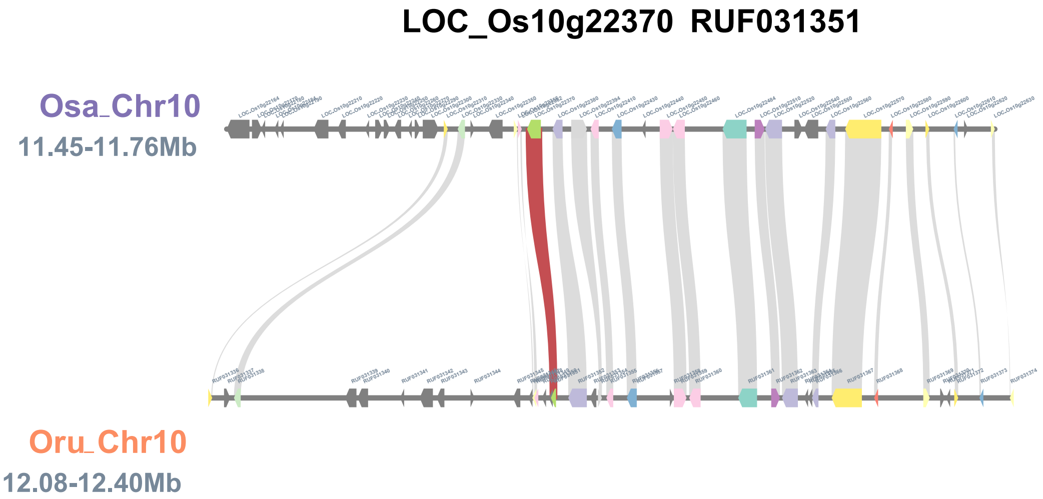

LOC_Os10g22370 RUF031351
Osa_Chr10
11.45-11.76Mb
Oru_Chr10
12.08-12.40Mb

LOC_Os10g26070 RUF031528

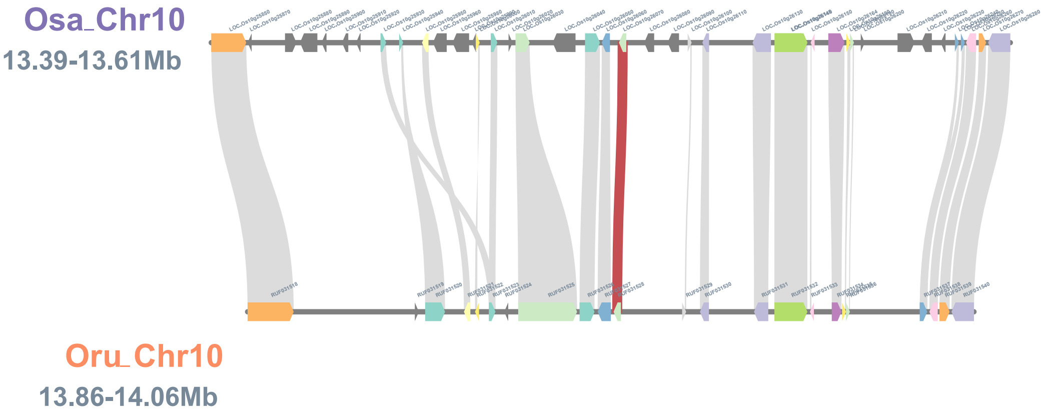

LOC_Os10g28600 RUF031674

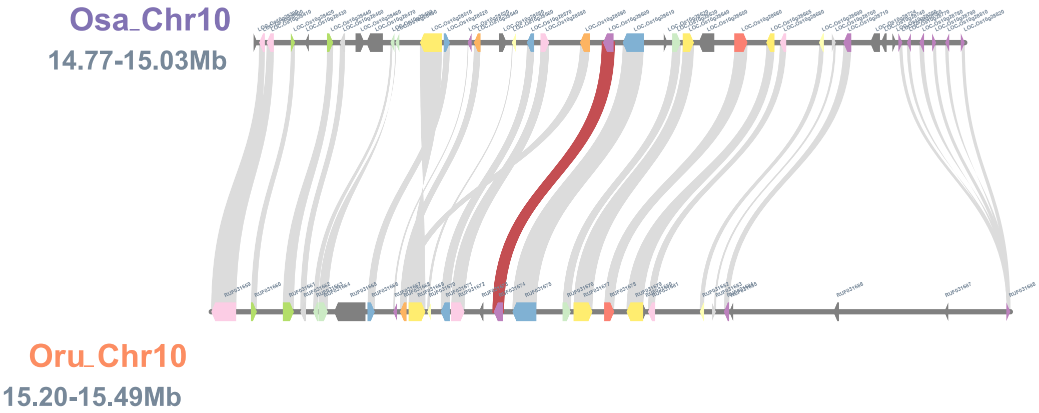

LOC_Os10g28640 RUF031677

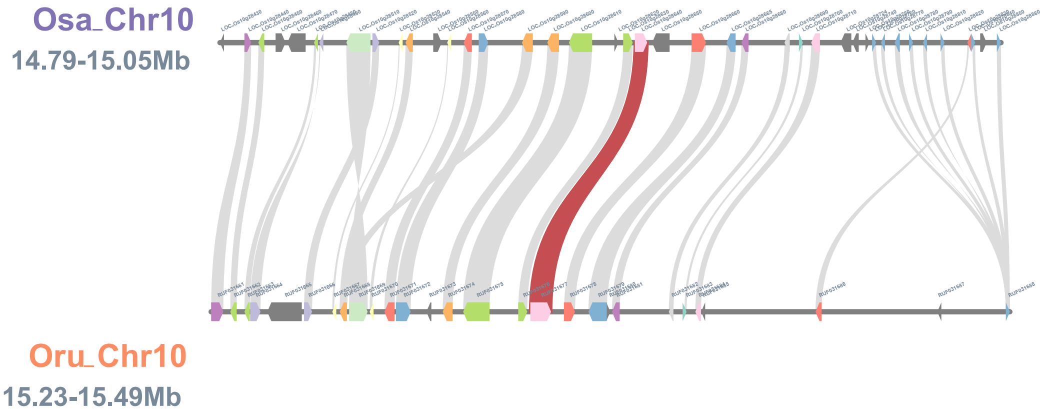

LOC_Os10g30760 RUF031772

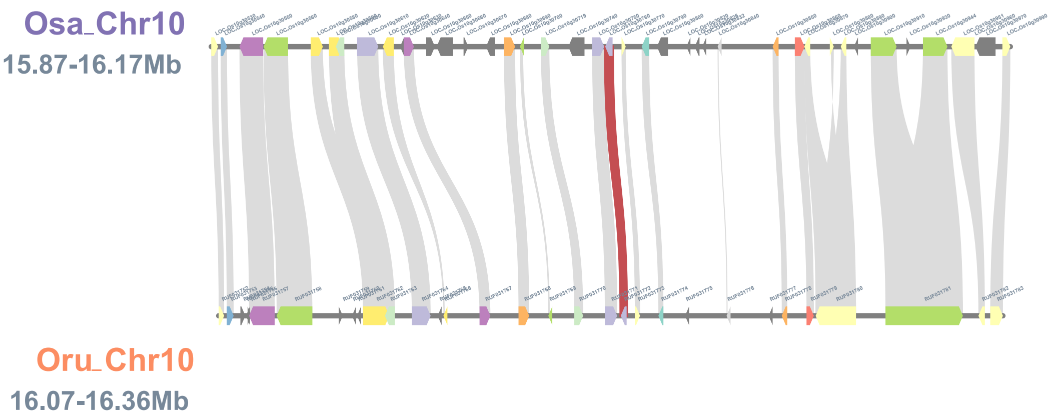

LOC_Os10g33700 RUF031968

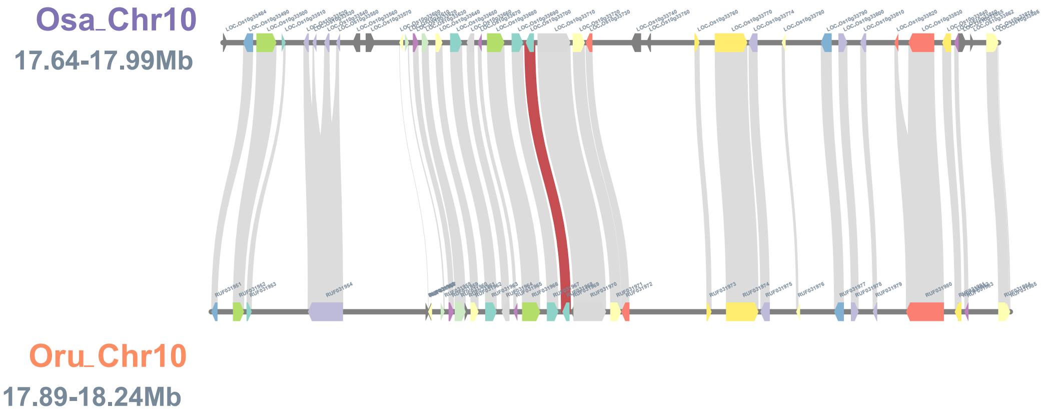

LOC_Os10g33730 RUF031972

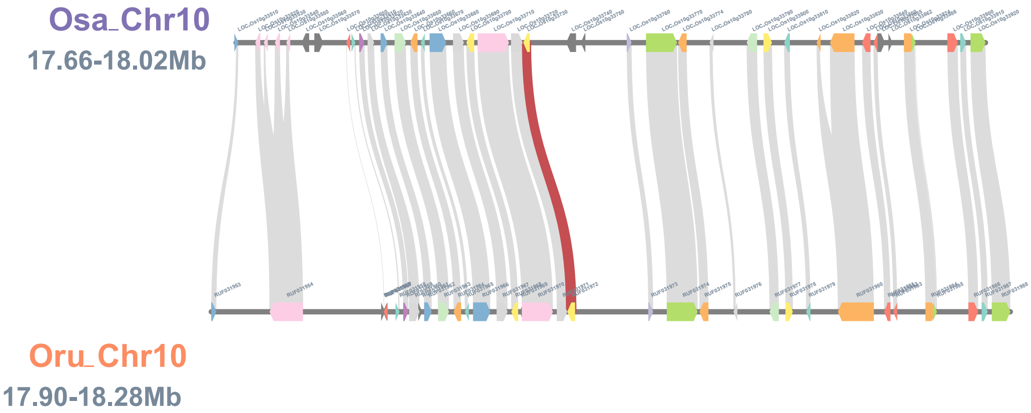

LOC_Os10g33874 RUF031984

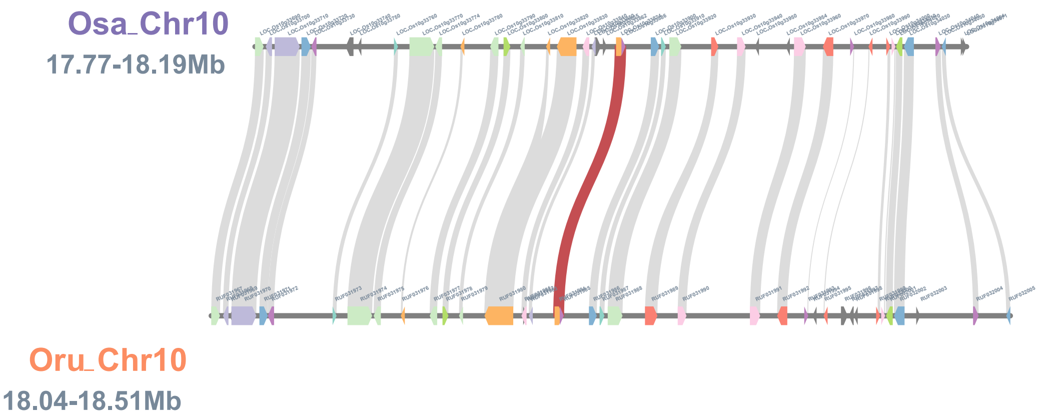

LOC_Os10g34310 RUF032021

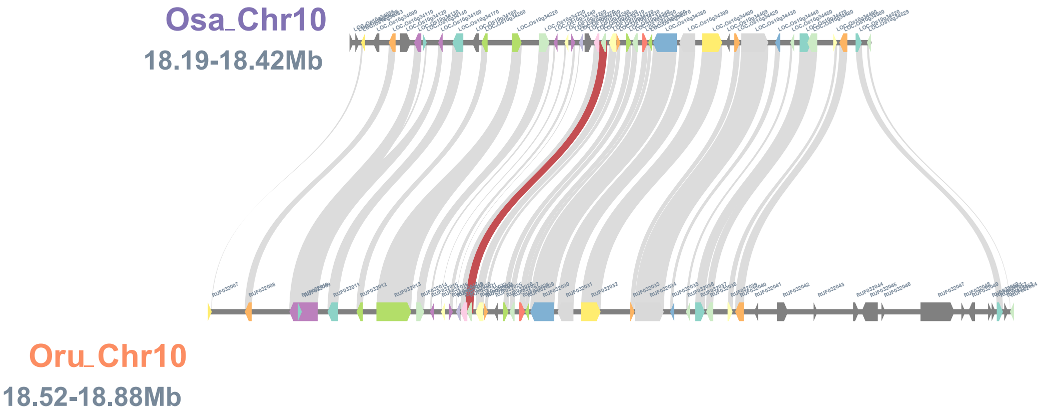

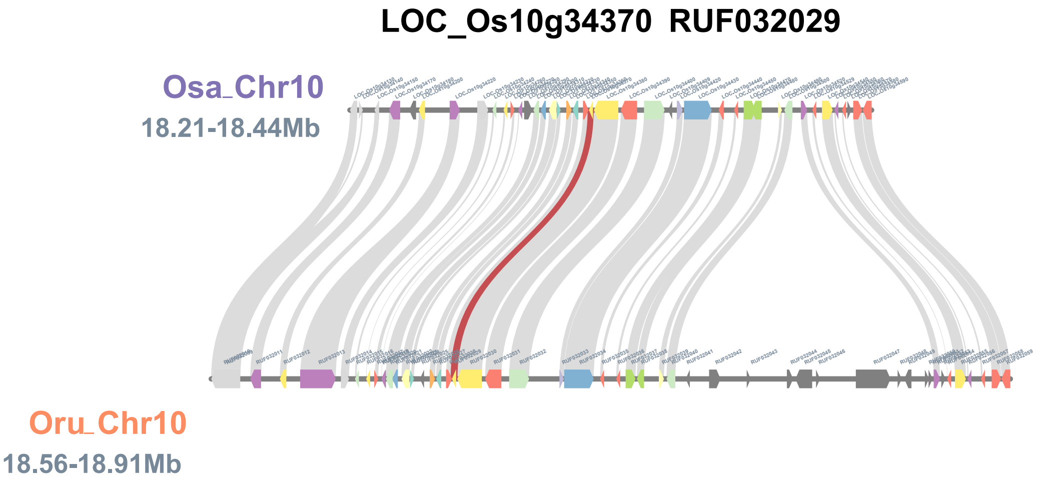

LOC_Os10g34370 RUF032029
Osa_Chr10
18.21-18.44Mb
Oru_Chr10
18.56-18.91Mb

LOC_Os10g34720 RUF032067

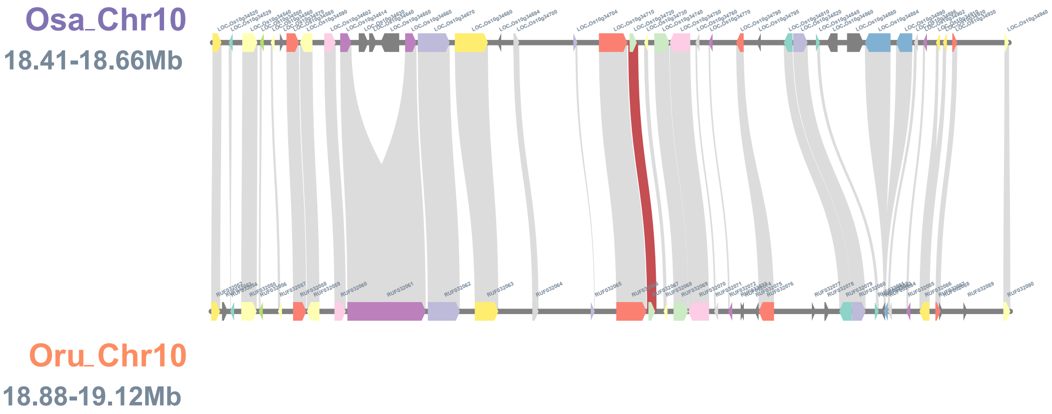

LOC_Os10g35230 RUF032111

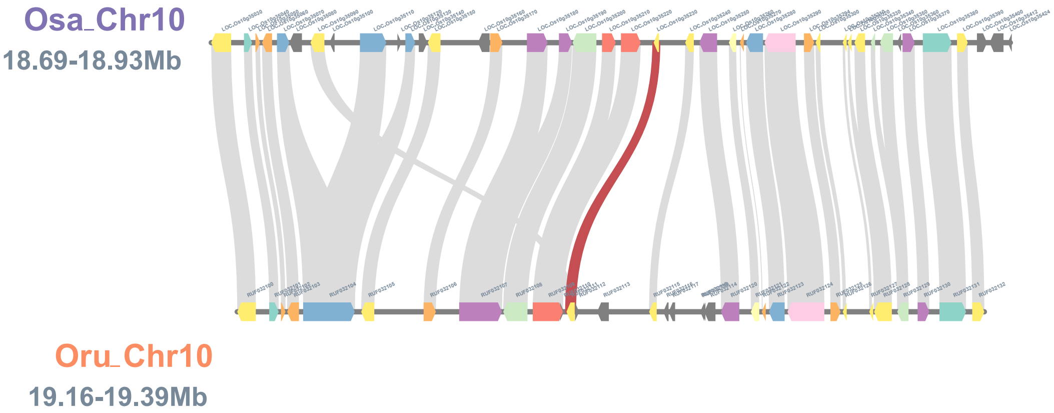

LOC_Os10g35240 RUF032115

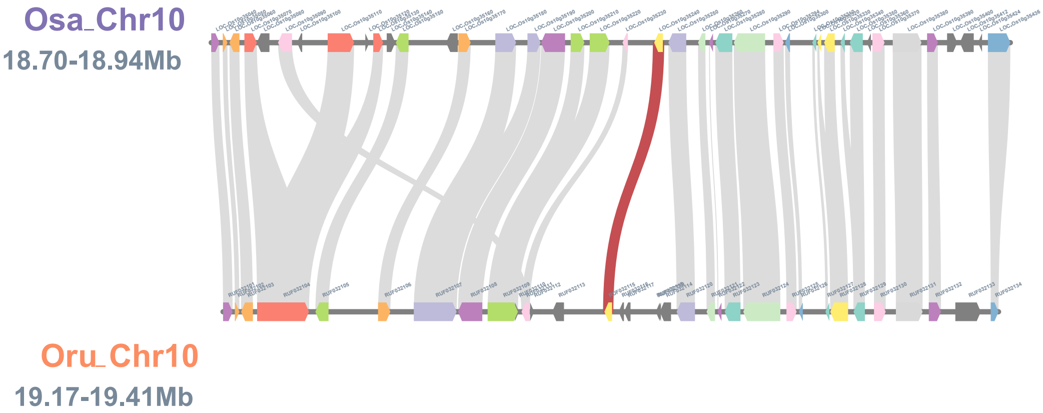

LOC_Os10g35260 RUF032121

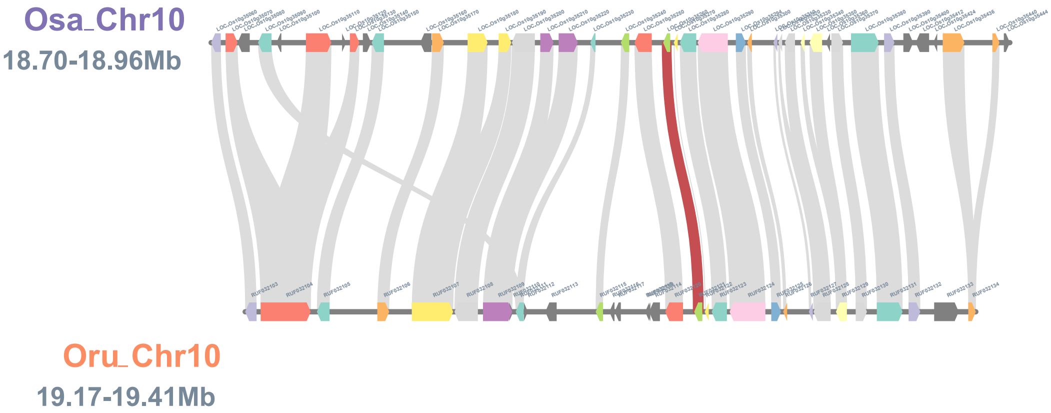

LOC_Os10g35436 RUF032134

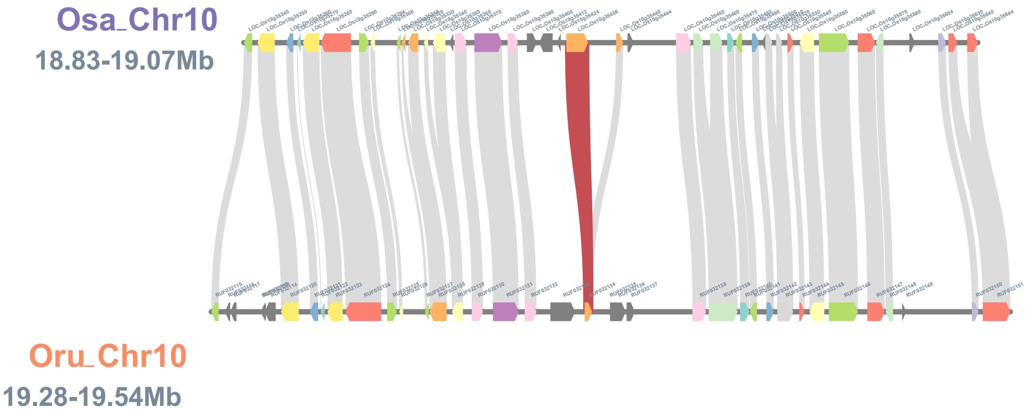

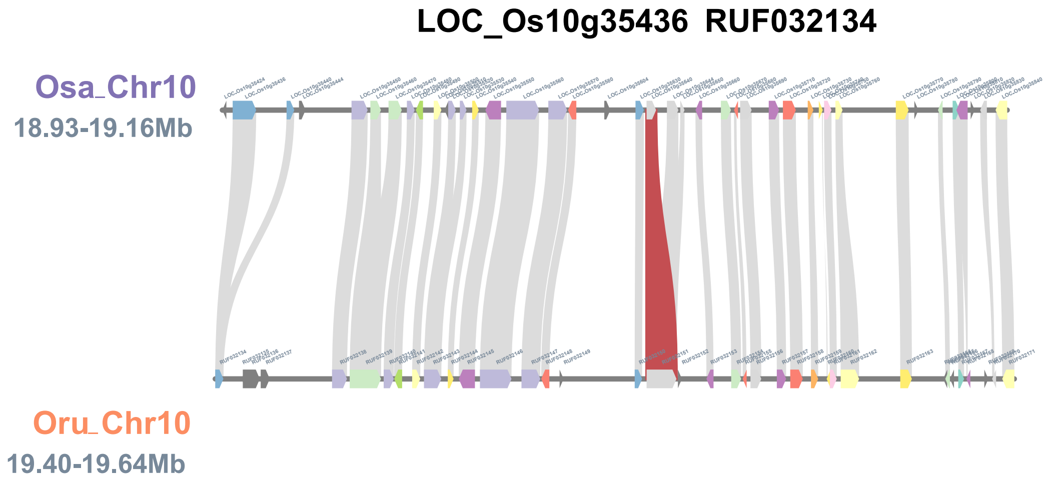
LOC_Os10g35436 RUF032134
Osa_Chr10
18.93-19.16Mb
Oru_Chr10
19.40-19.64Mb

LOC_Os10g35730 RUF032159

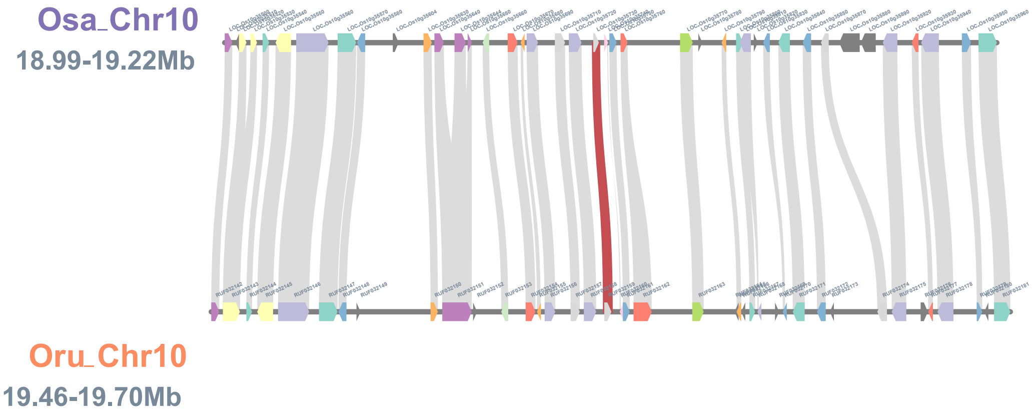

LOC_Os10g35750 RUF032161

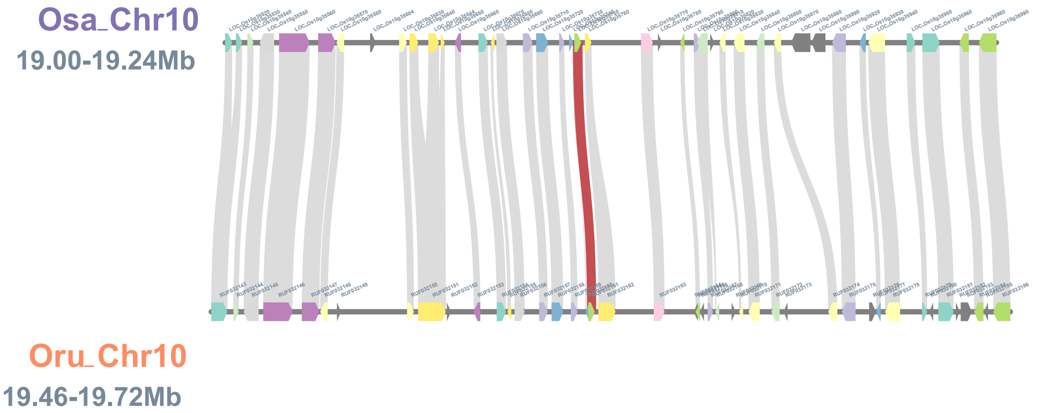

LOC_Os10g35760 RUF032162

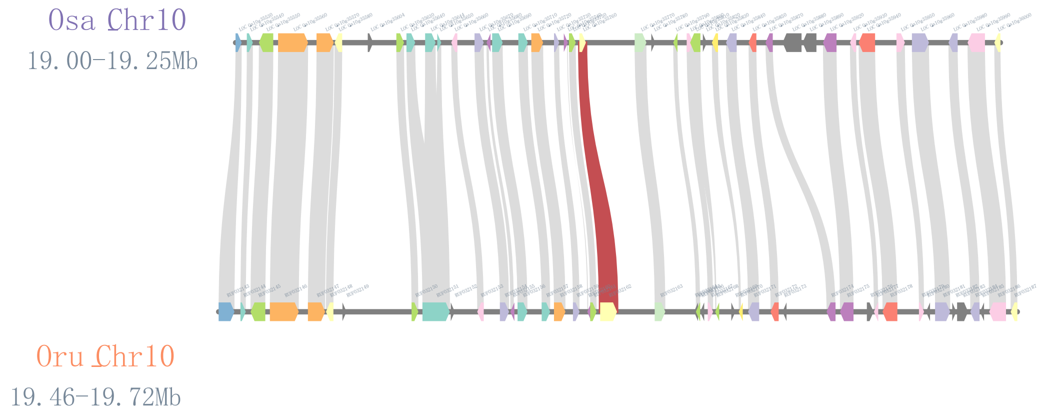

LOC_Os10g35790 RUF032165

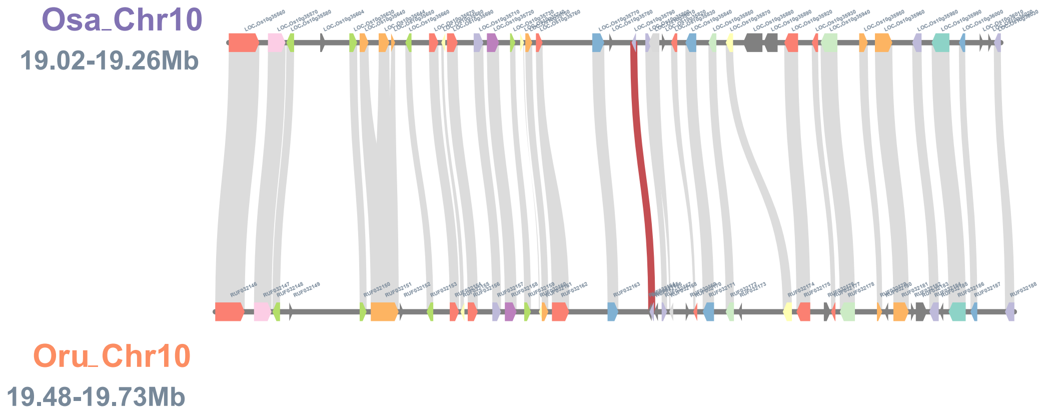

LOC_Os10g36190 RUF032198

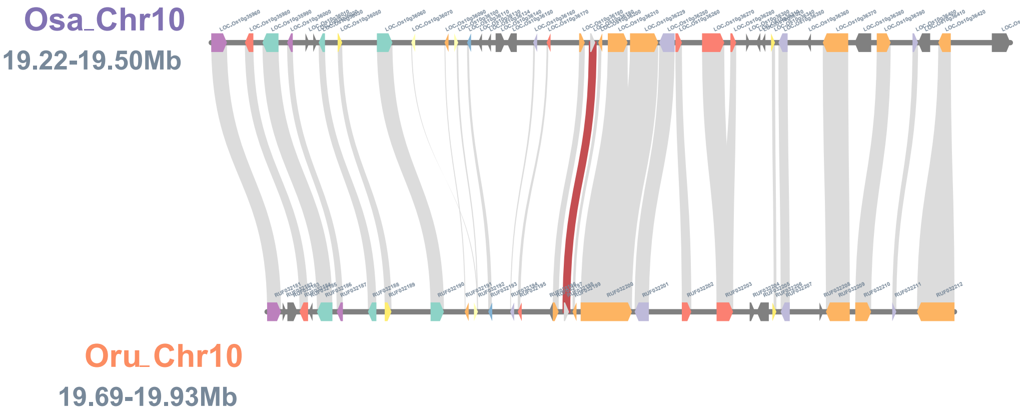

LOC_Os10g39460 RUF032417

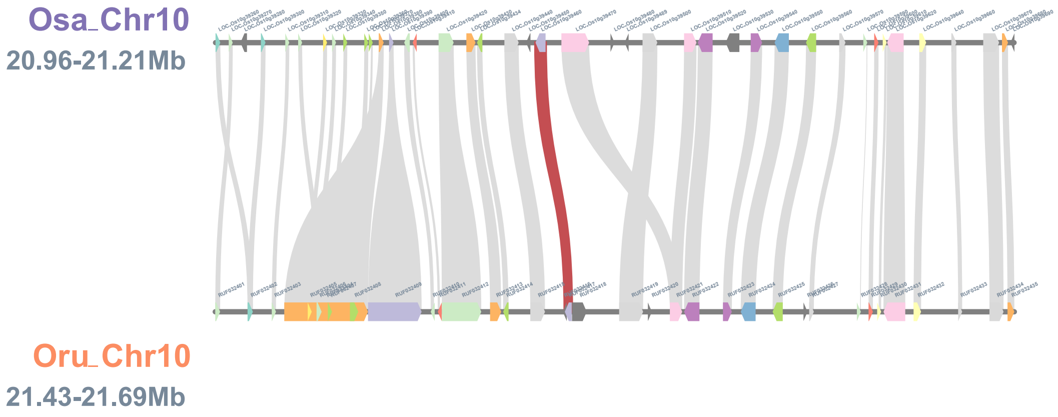

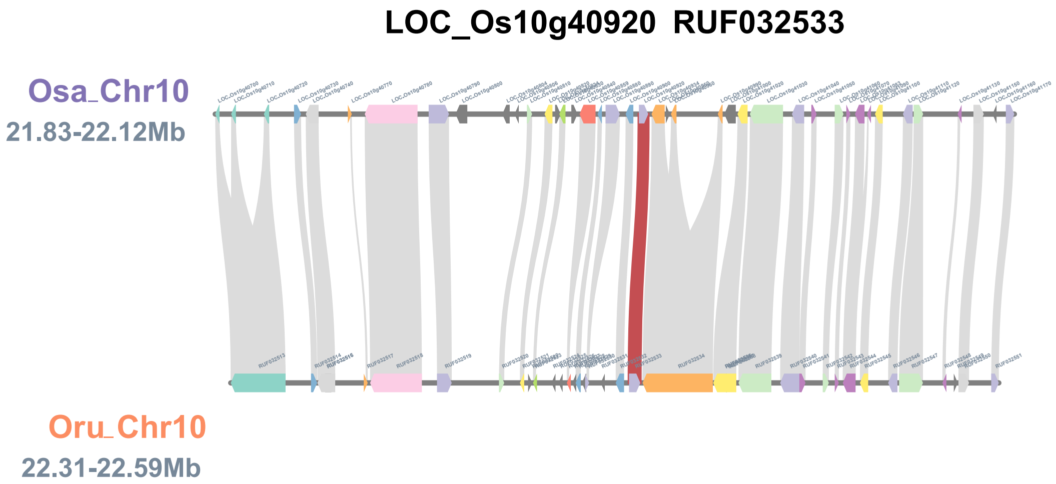
LOC_Os10g40920 RUF032533
Osa_Chr10
21.83-22.12Mb
Oru_Chr10
22.31-22.59Mb

LOC_Os10g41740 RUF032598

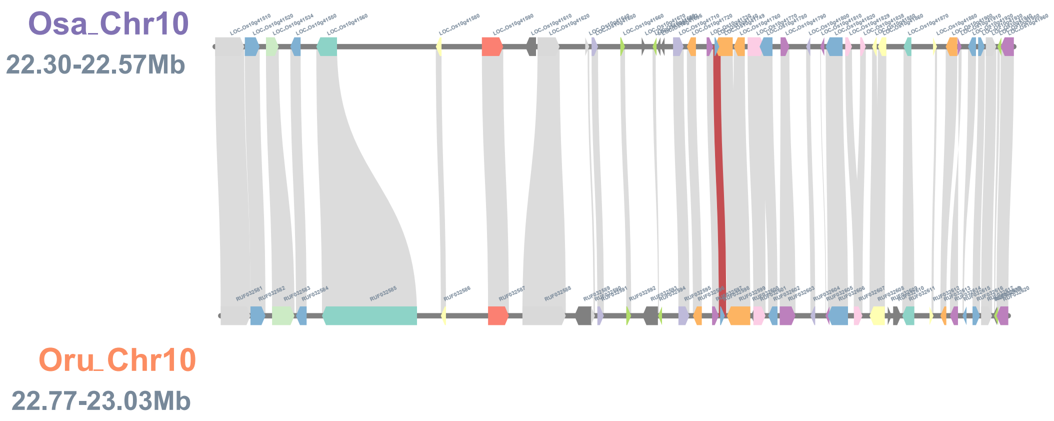

LOC_Os10g42760 RUF032681

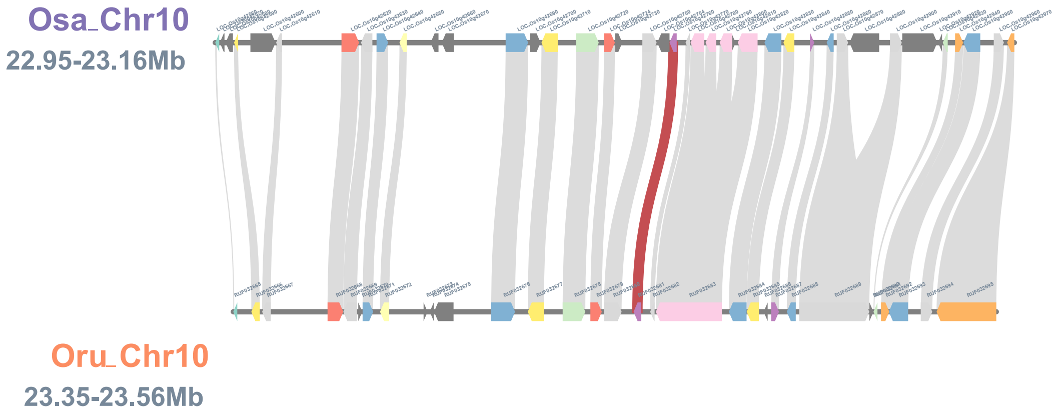

LOC_Os11g03690 RUF032847

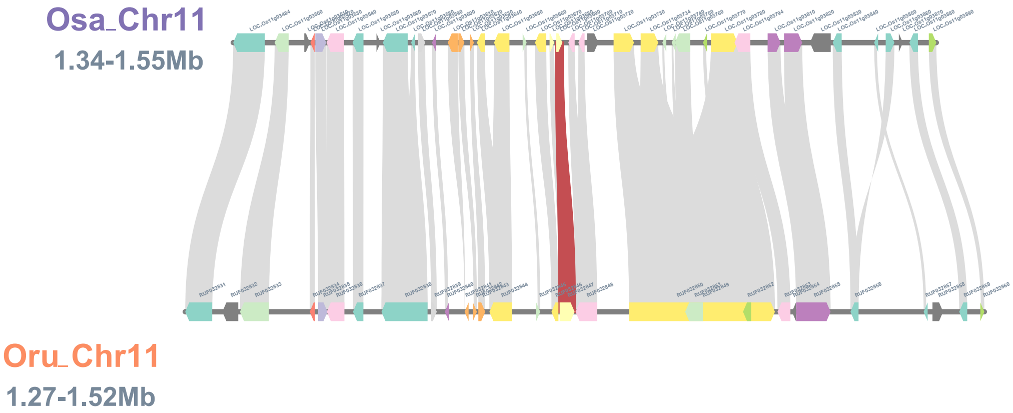

LOC_Os11g04295 RUF032883

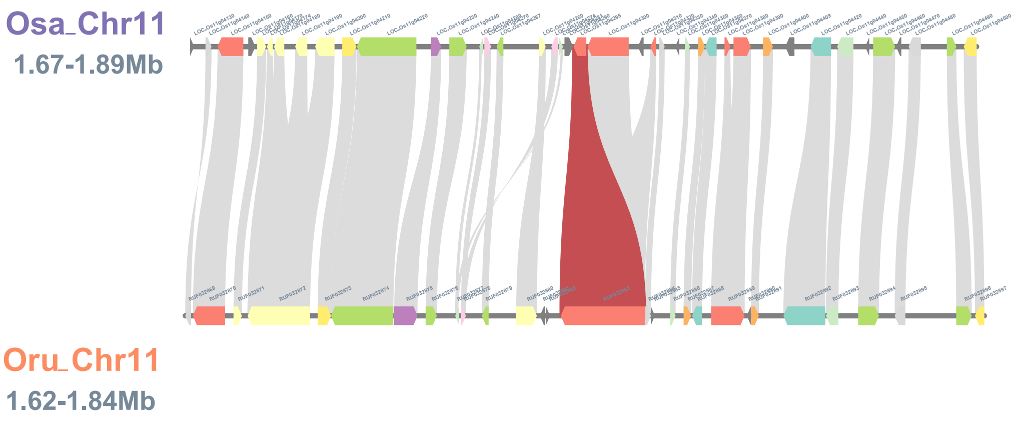

LOC_Os11g10740 RUF033344

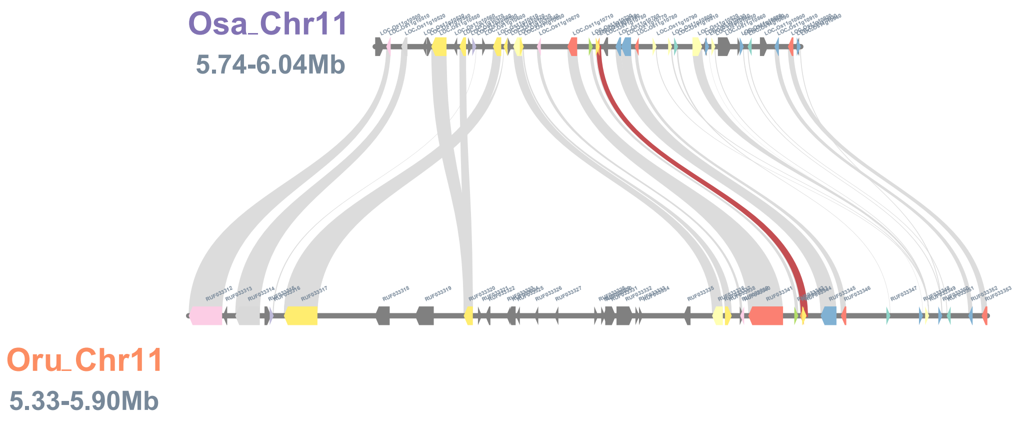

LOC_Os11g14980 RUF033553

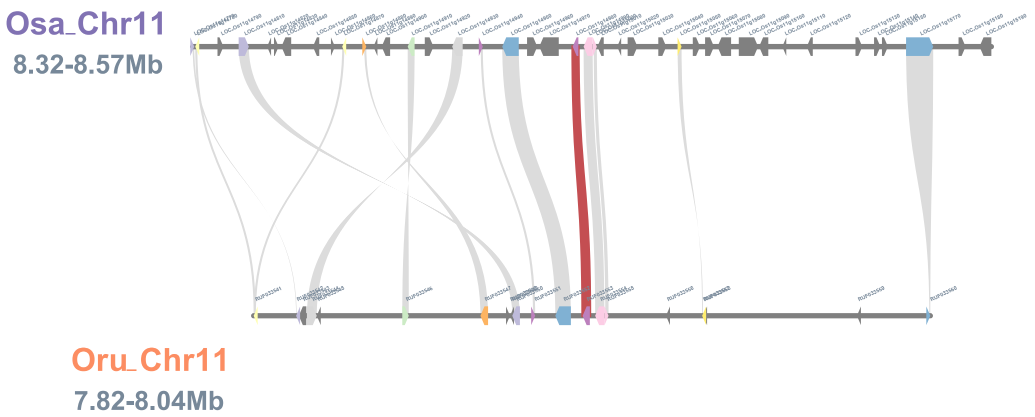

LOC_Os11g24530 RUF033804

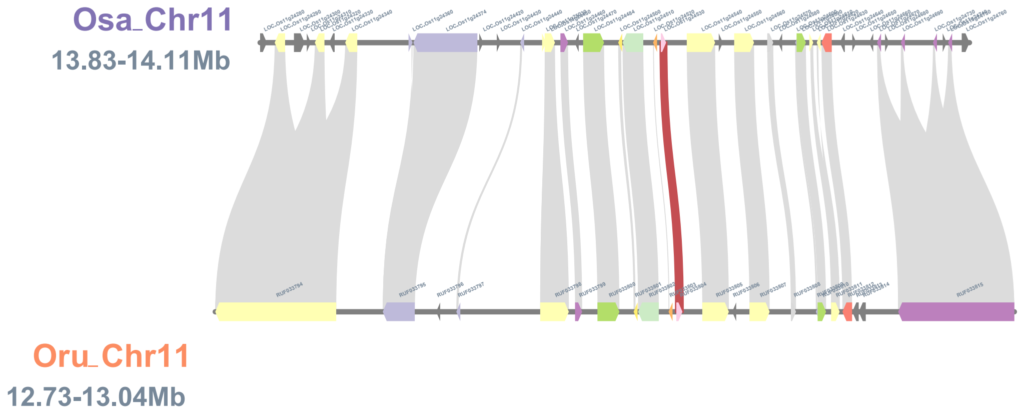

LOC_Os11g24570 RUF033808

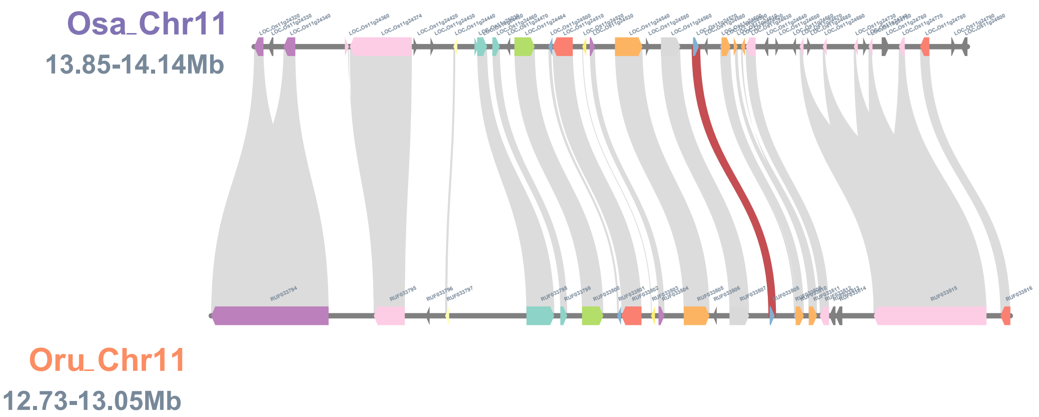

LOC_Os11g37330 RUF034583

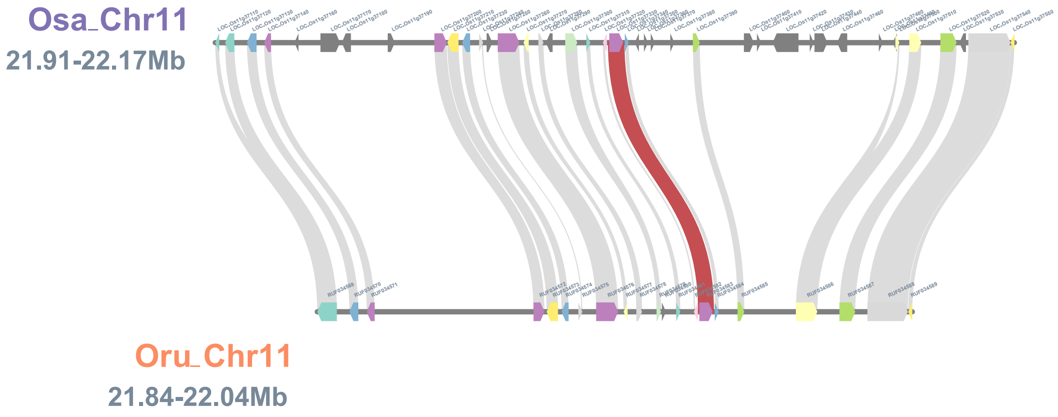

LOC_Os11g37660 RUF034594

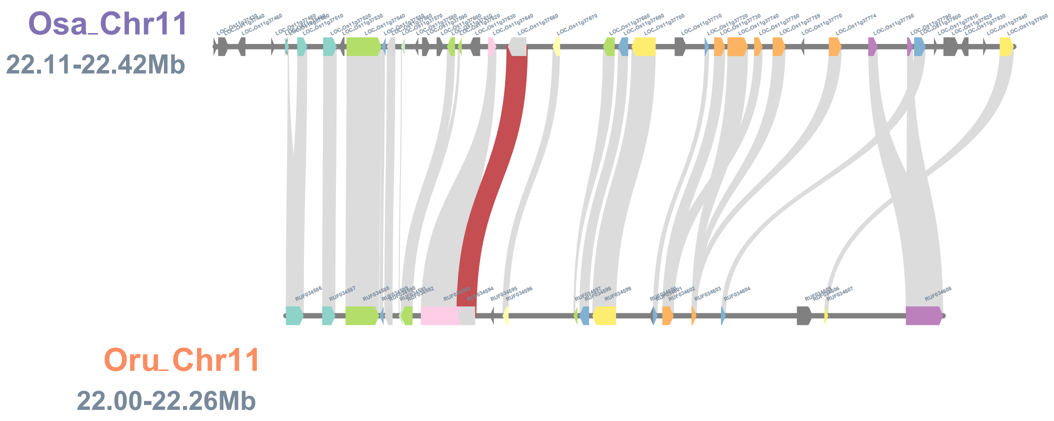

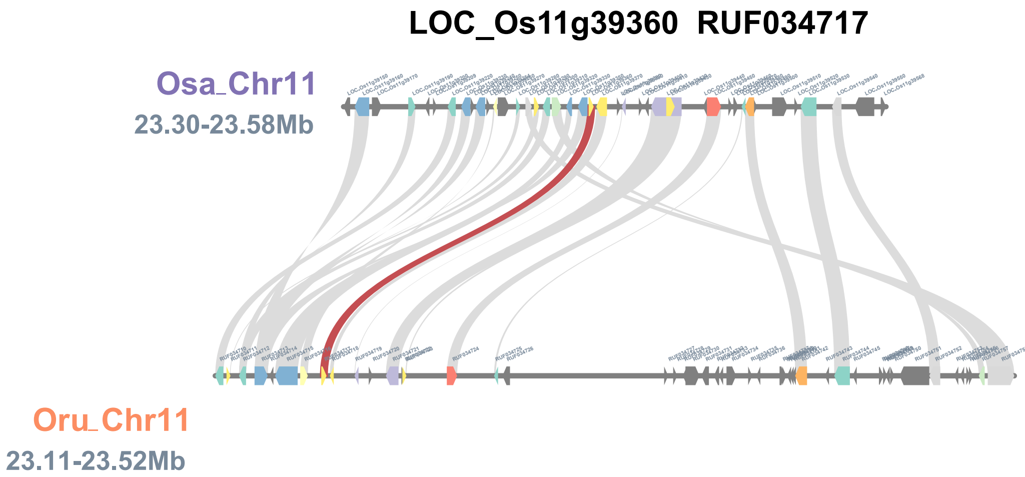
LOC_Os11g39360 RUF034717
Osa_Chr11
23.30-23.58Mb
Oru_Chr11
23.11-23.52Mb

LOC_Os11g44930 RUF035139

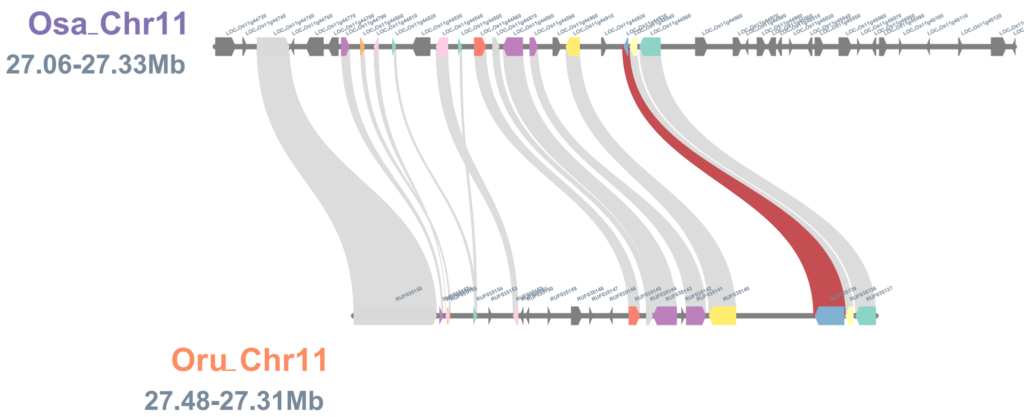

LOC_Os12g01210 RUF035397

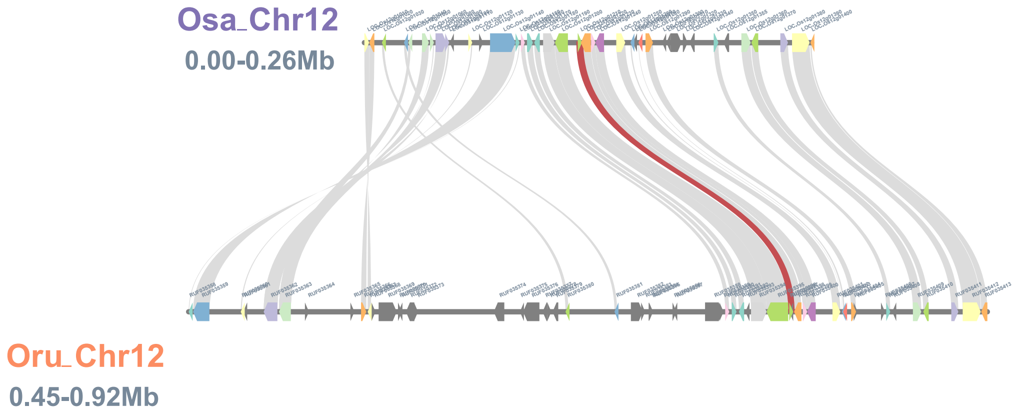

LOC_Os12g01910 RUF035442

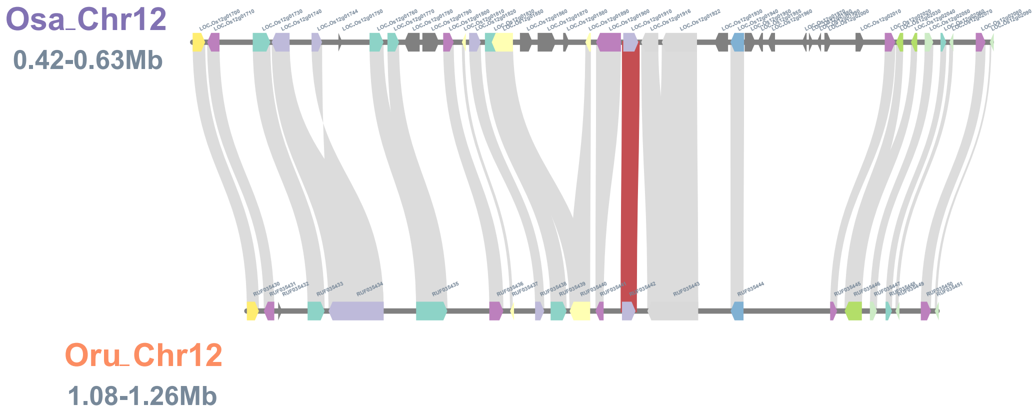

LOC_Os12g02280 RUF035463

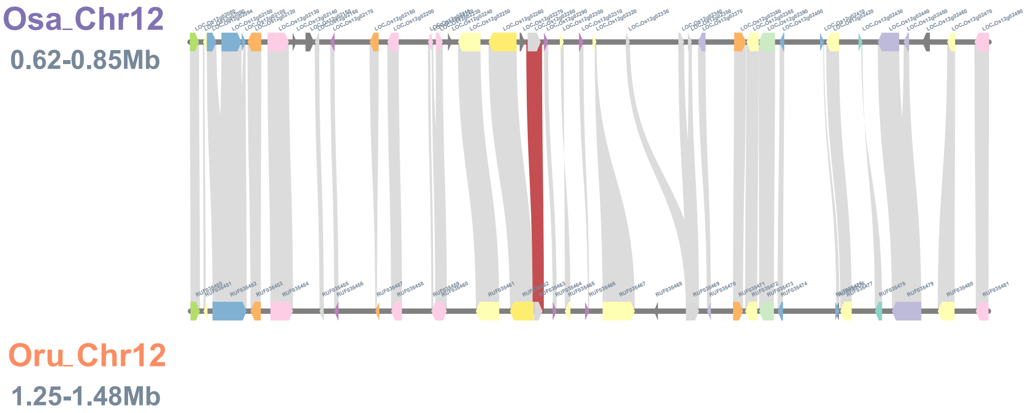

LOC_Os12g03450 RUF035549

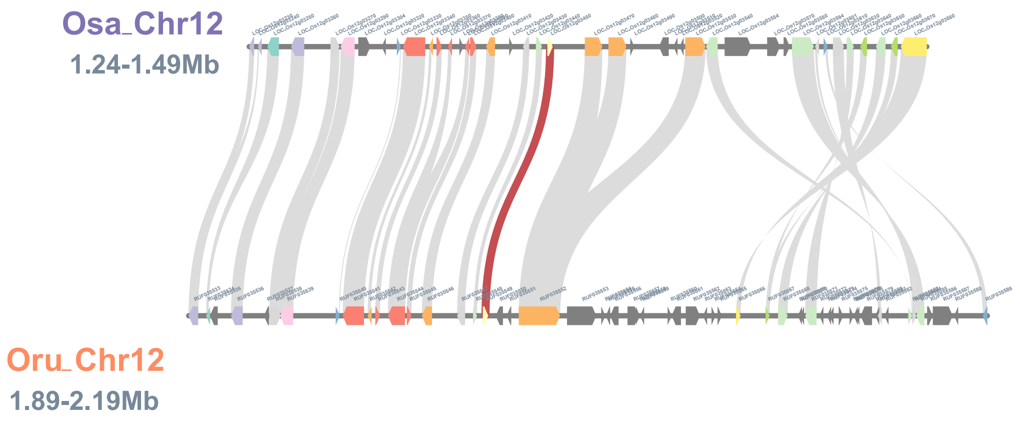

LOC_Os12g05640 RUF035737

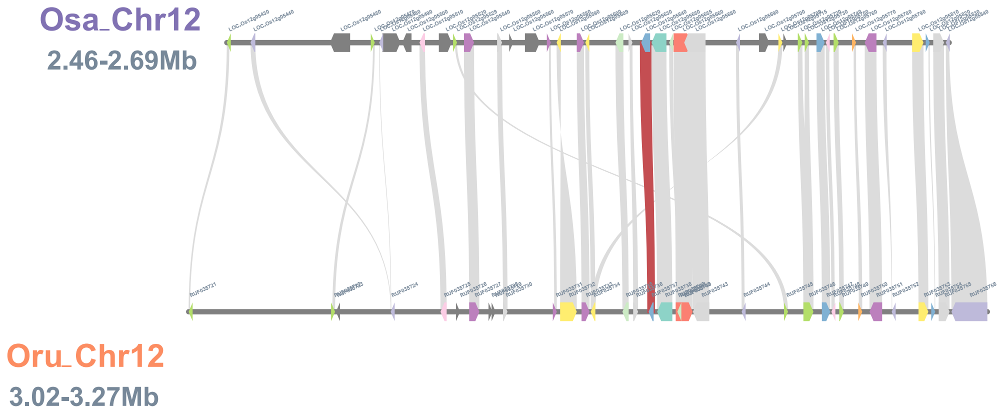

LOC_Os12g06070 RUF035773

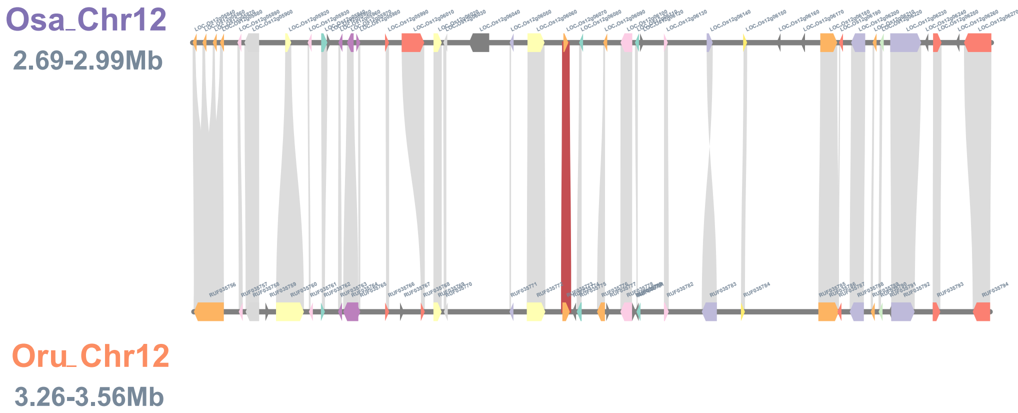

LOC_Os12g06650 RUF035821

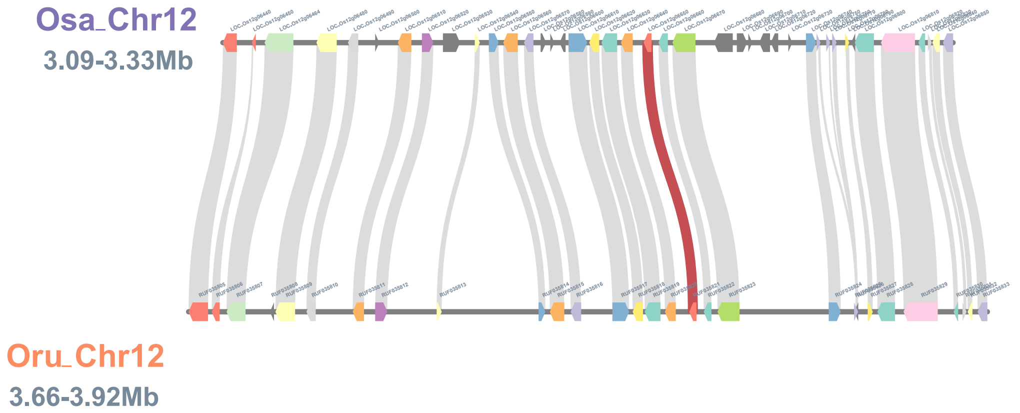

LOC_Os12g07260 RUF035863

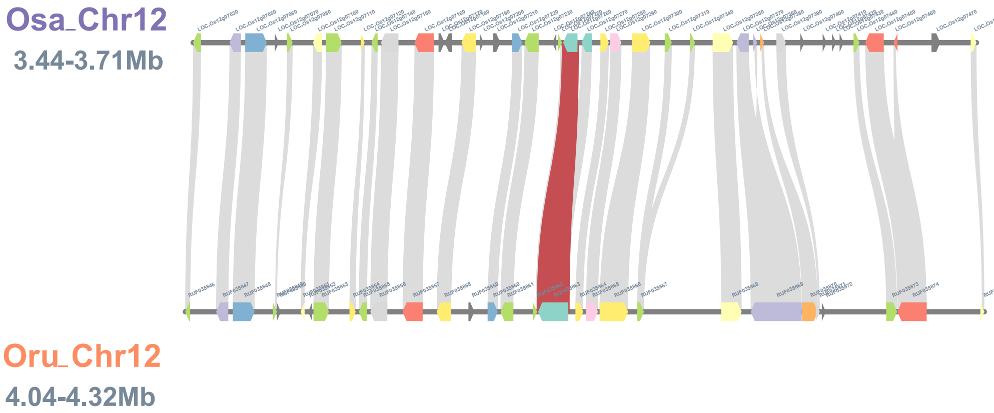

LOC_Os12g08140 RUF035917

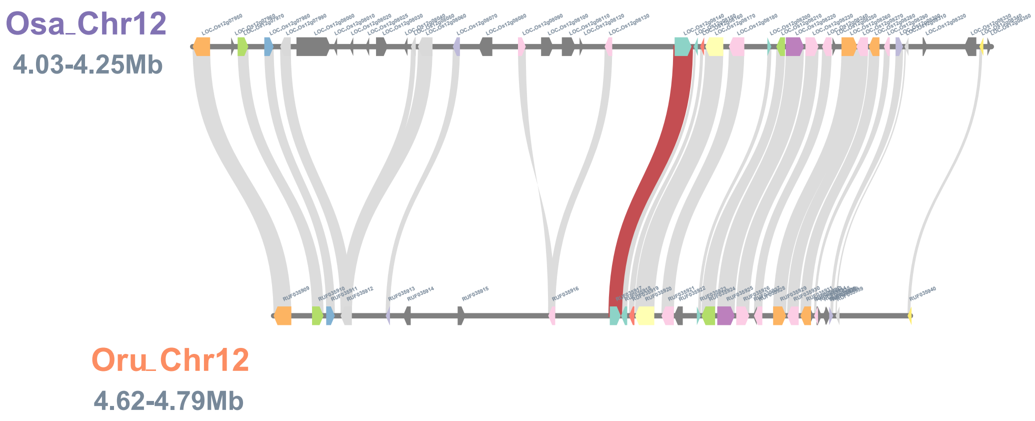

LOC_Os12g13110 RUF036086

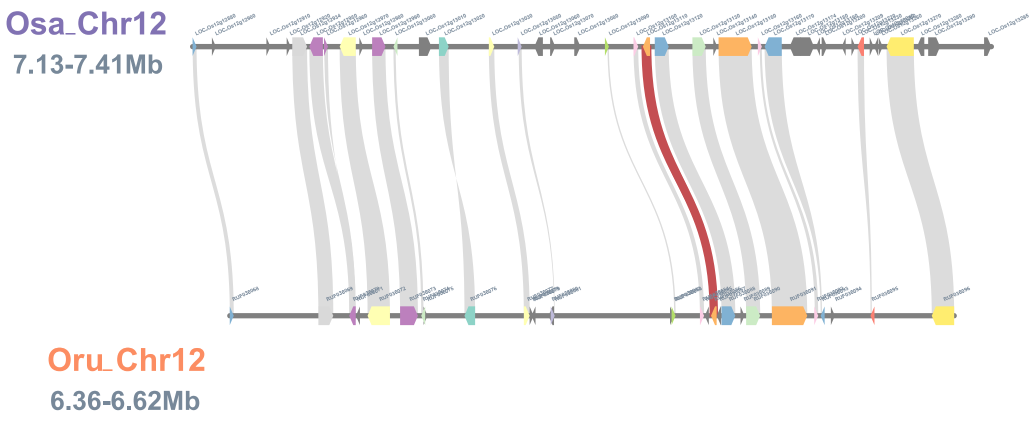

LOC_Os12g15460 RUF036173

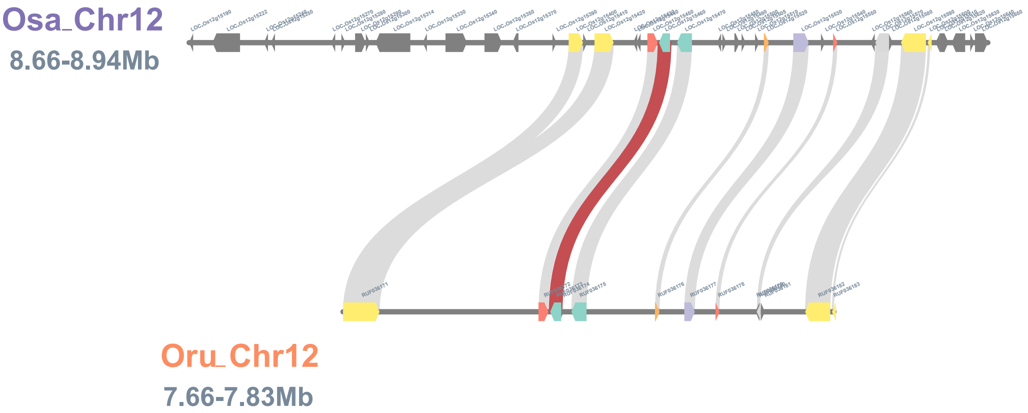

LOC_Os12g17080 RUF036231

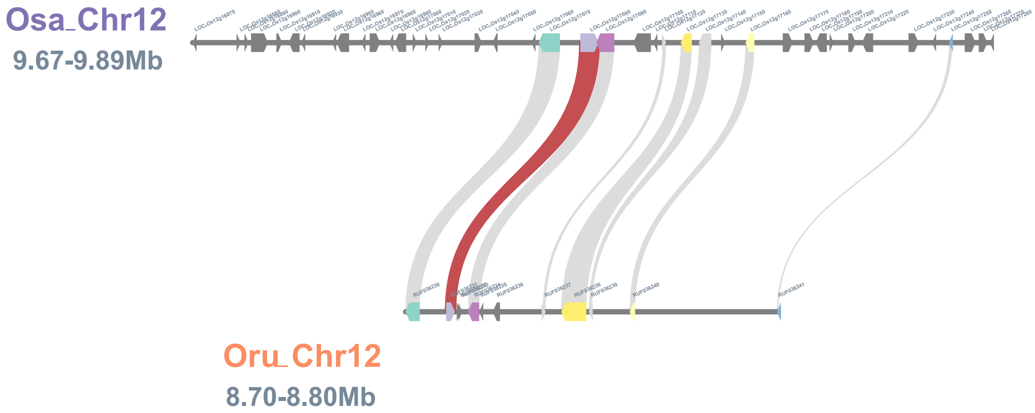

LOC_Os12g18640 RUF036324

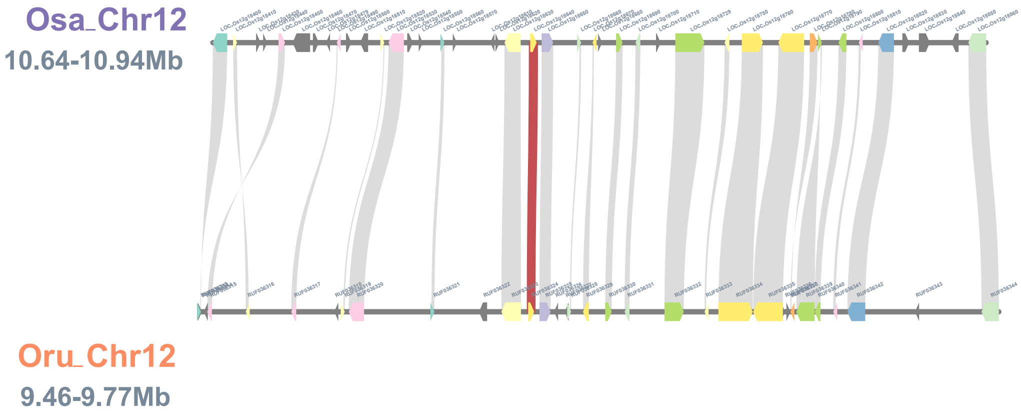

LOC_Os12g19260 RUF036357

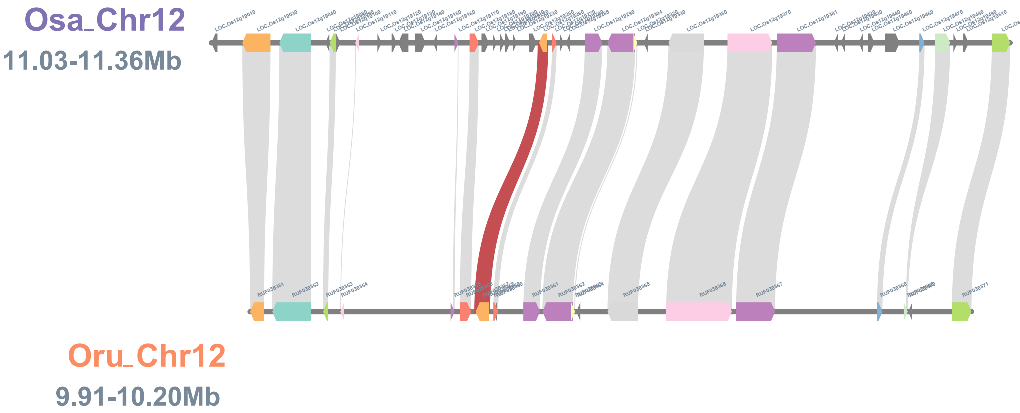

LOC_Os12g27060 RUF036568

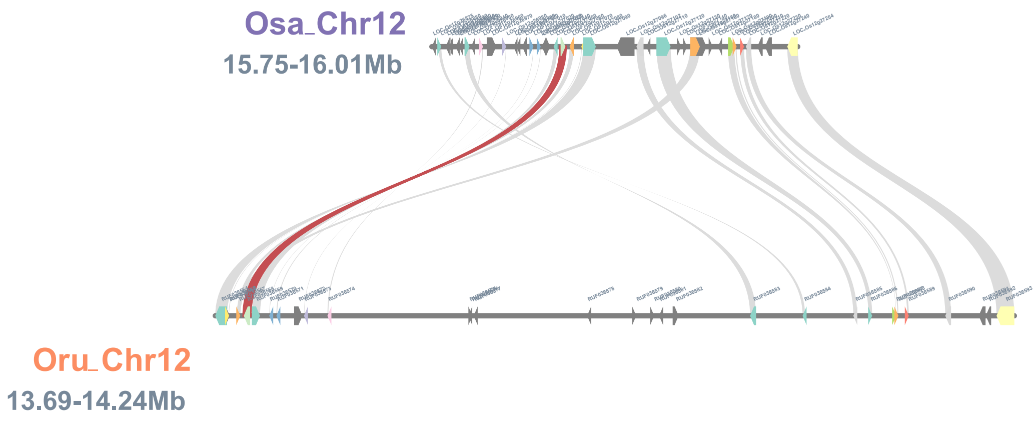

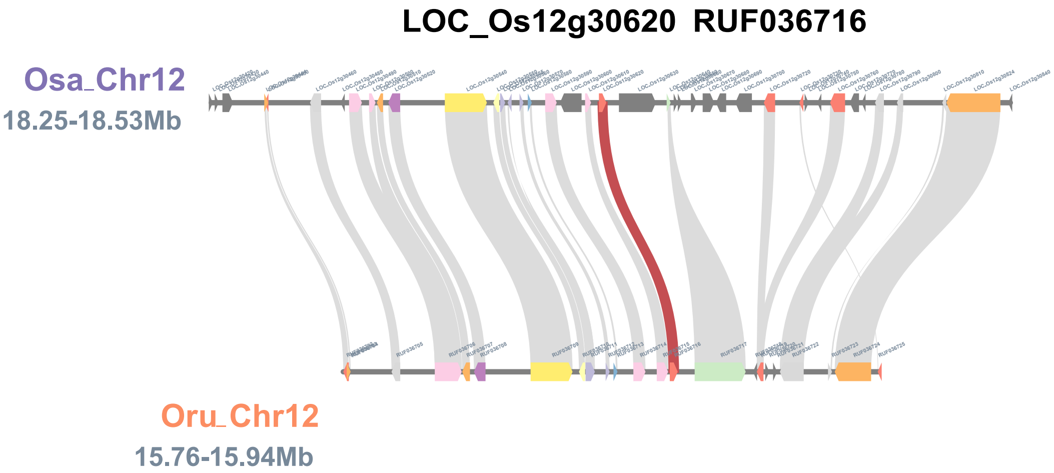
LOC_Os12g30620 RUF036716
Osa_Chr12
18.25-18.53Mb
Oru_Chr12
15.76-15.94Mb

LOC_Os12g34340 RUF037004

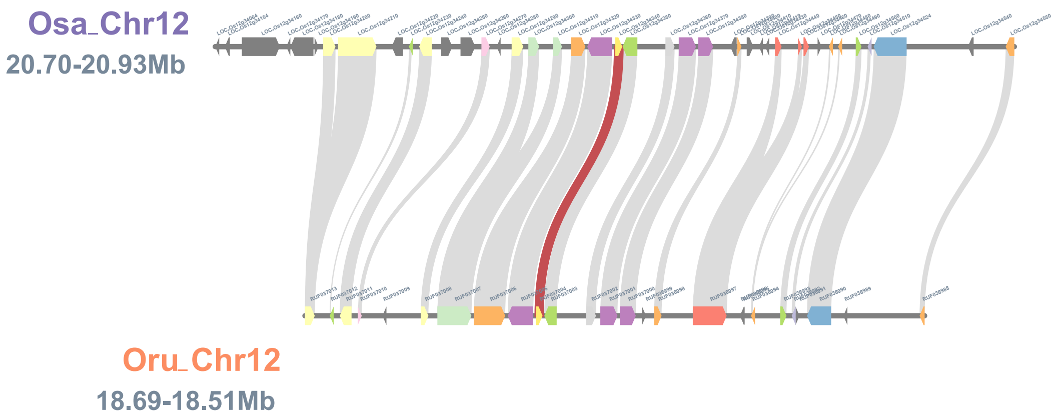

LOC_Os12g36620 RUF036859

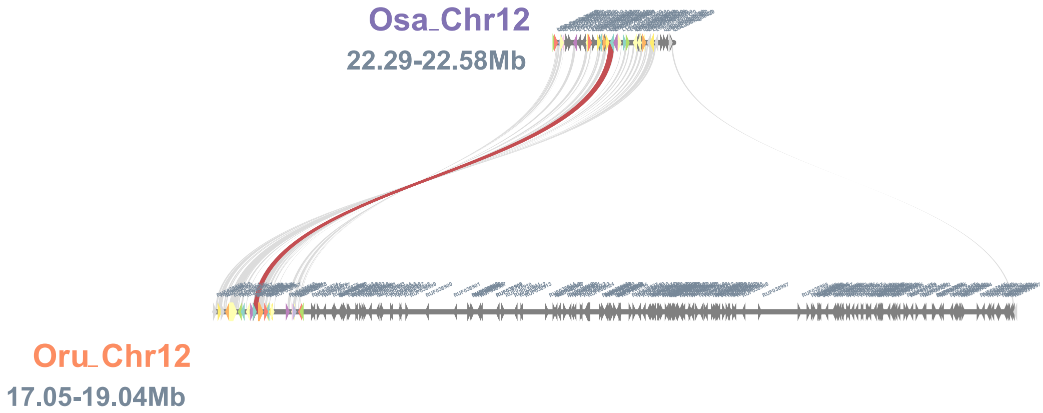

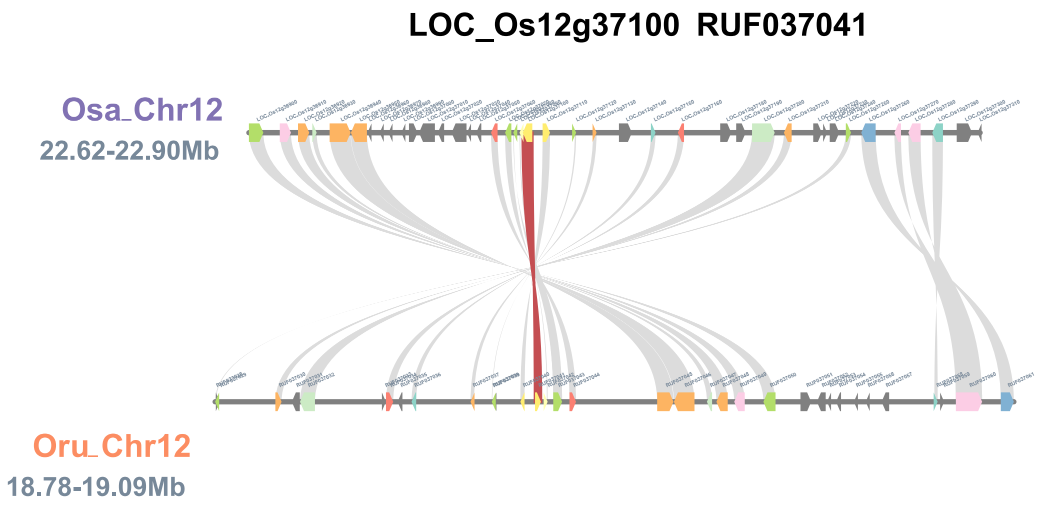
LOC_Os12g37100 RUF037041
Osa_Chr12
22.62-22.90Mb
Oru_Chr12
18.78-19.09Mb

LOC_Os12g38880 RUF037179

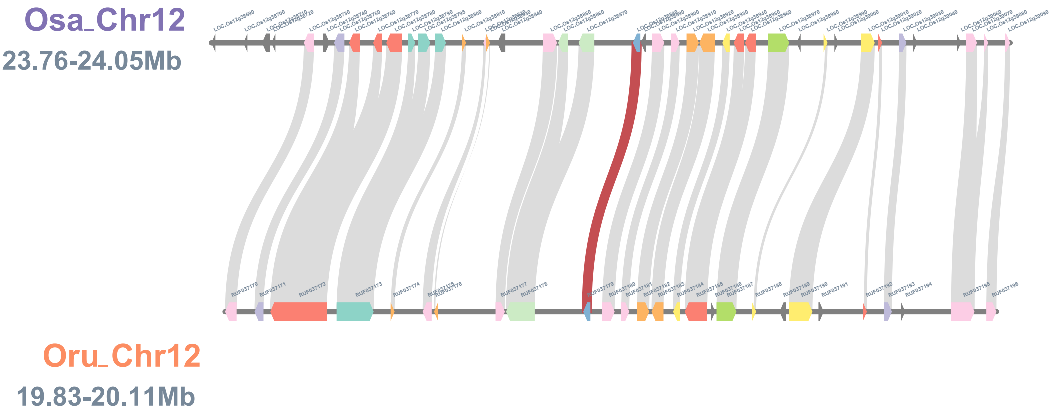

LOC_Os12g44170 RUF037528

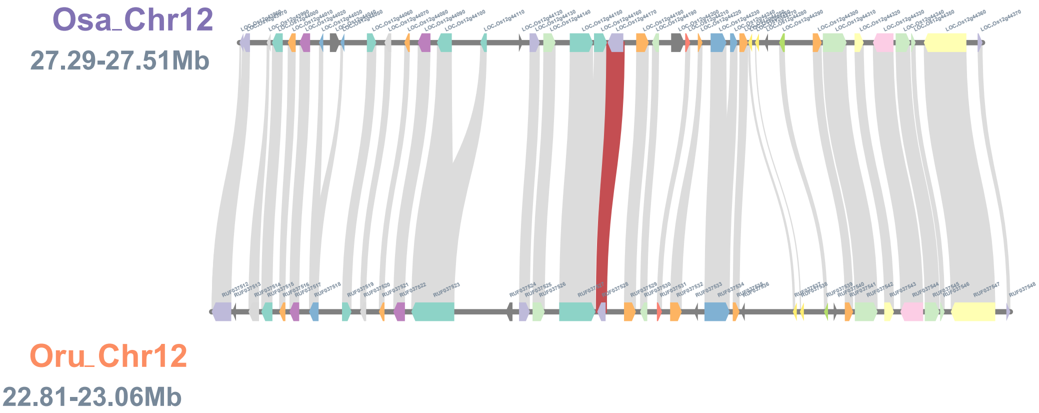

Supplement: Supplementary file 1 [file ijms-24-16313-s001.zip › Figure S10.pdf]
