# Supplementary material for: Comparative Genomic Analysis of Asian Cultivated Rice and Its Wild Progenitor (Oryza rufipogon) Has Revealed Evolutionary Innovation of the Pentatricopeptide Repeat Gene Family through Gene Duplication
Source: Int J Mol Sci. 2023 Nov 14;24(22):16313. doi: 10.3390/ijms242216313 (PMC10671101; doi:10.3390/ijms242216313)
Supplement: Supplementary file 1 [file ijms-24-16313-s001.zip › Figure S5.pdf]

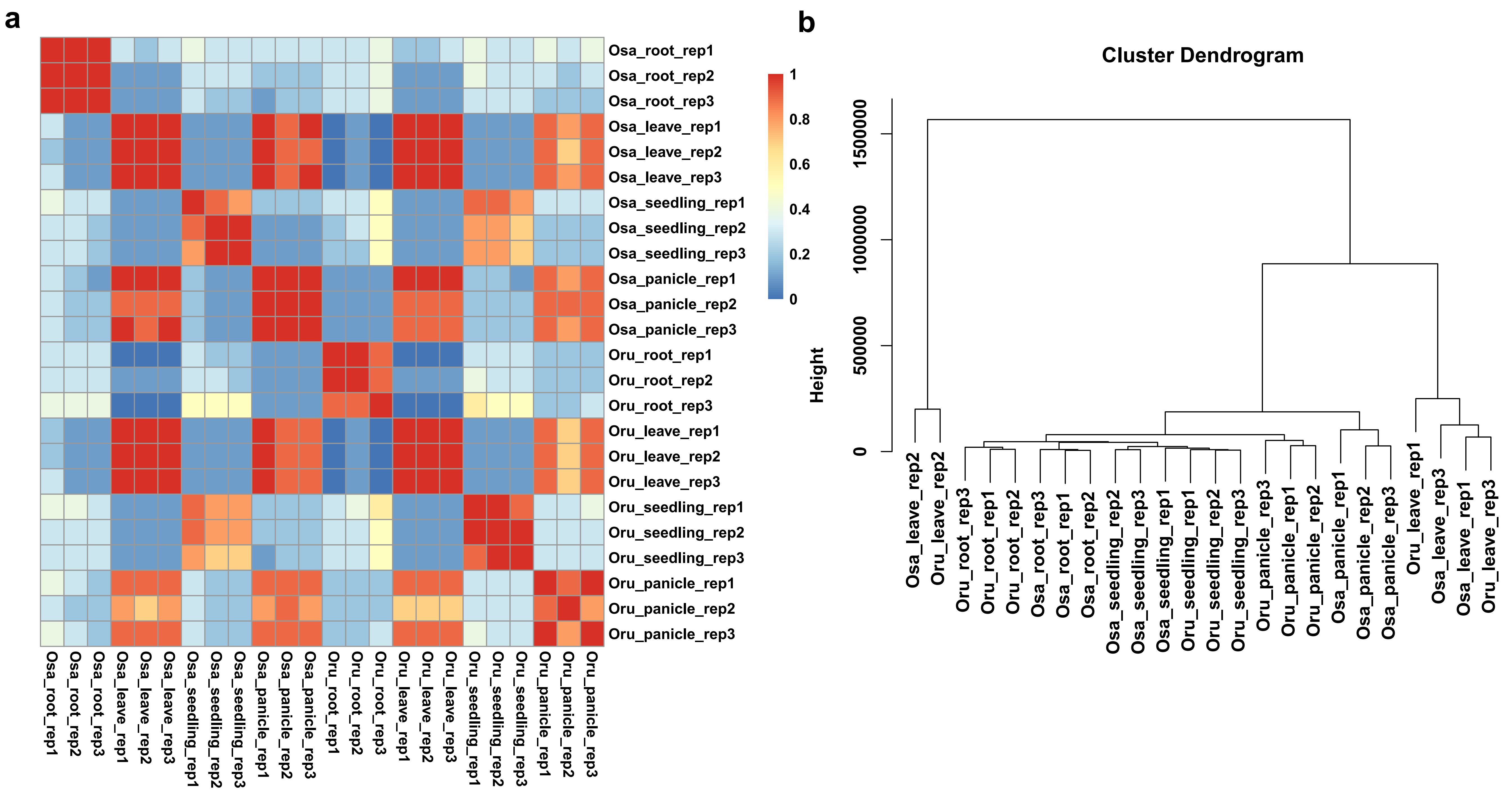
a
Osa_root_rep1
Osa_root_rep2
Osa_root_rep3
Osa_leave_rep1
Osa_leave_rep2
Osa_leave_rep3
Osa_seedling_rep1
Osa_seedling_rep2
Osa_seedling_rep3
Osa_panicle_rep1
Osa_panicle_rep2
Osa_panicle_rep3
Oru_root_rep1
Oru_root_rep2
Oru_root_rep3
Oru_leave_rep1
Oru_leave_rep2
Oru_leave_rep3
Oru_seedling_rep1
Oru_seedling_rep2
Oru_seedling_rep3
Oru_panicle_rep1
Oru_panicle_rep2
Oru_panicle_rep3
1
0.8
0.6
0.4
0.2
0
b
Cluster Dendrogram
Height
1500000
1000000
500000
0
Osa_leave_rep2
Oru_leave_rep2
Oru_root_rep3
Oru_root_rep1
Oru_root_rep2
Osa_root_rep3
Osa_root_rep1
Osa_root_rep2
Osa_seedling_rep2
Osa_seedling_rep3
Osa_seedling_rep1
Oru_seedling_rep1
Oru_seedling_rep2
Oru_seedling_rep3
Oru_panicle_rep3
Oru_panicle_rep1
Oru_panicle_rep2
Osa_panicle_rep1
Osa_panicle_rep2
Osa_panicle_rep3
Oru_leave_rep1
Osa_leave_rep3
Osa_leave_rep1
Oru_leave_rep3
