# Supplementary figures and images for: Comparative Genomic Analysis of Asian Cultivated Rice and Its Wild Progenitor (Oryza rufipogon) Has Revealed Evolutionary Innovation of the Pentatricopeptide Repeat Gene Family through Gene Duplication
Source: Int J Mol Sci. 2023 Nov 14;24(22):16313. doi: 10.3390/ijms242216313 (PMC10671101; doi:10.3390/ijms242216313)

**a**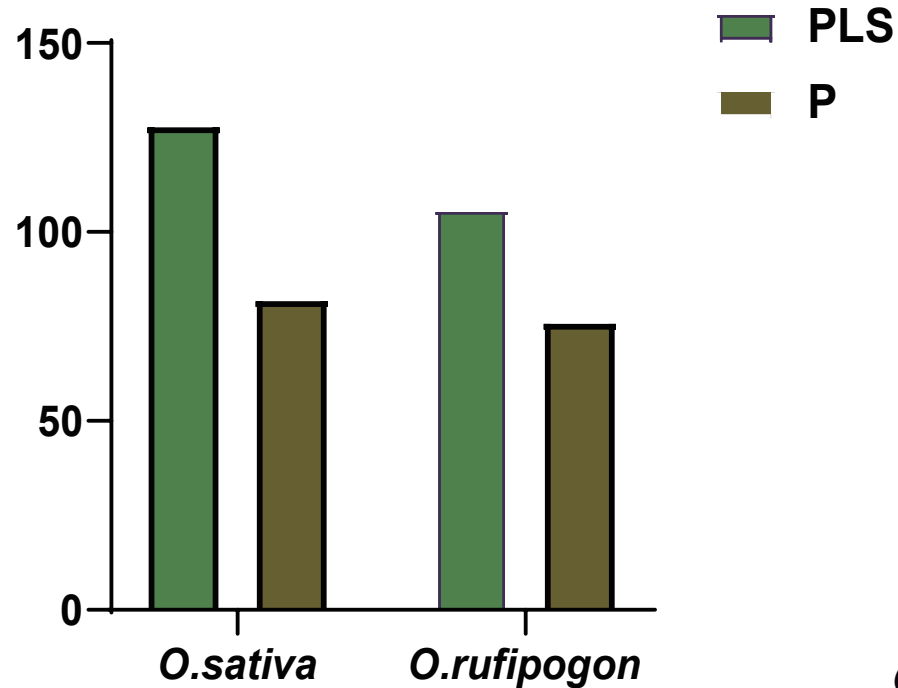**b**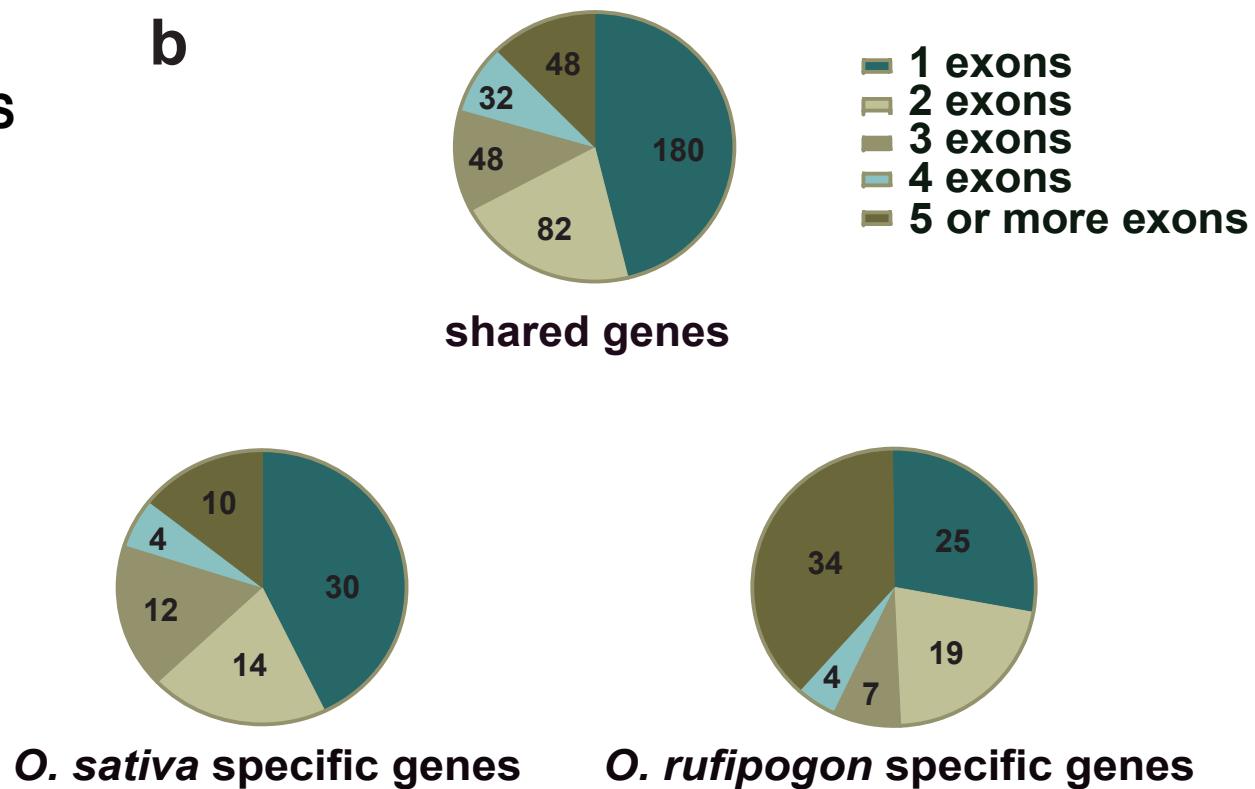

Supplement: Supplementary file 1 [file ijms-24-16313-s001.zip › Figure S1.pdf]

**a**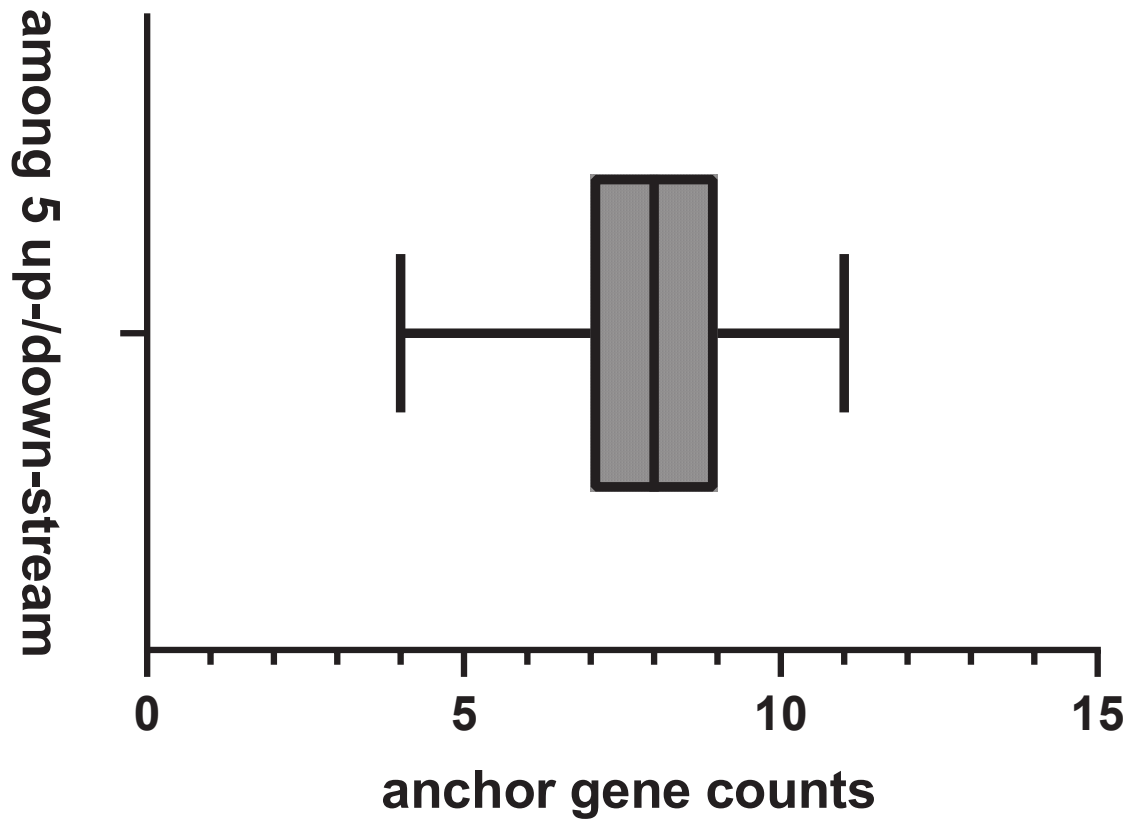**b**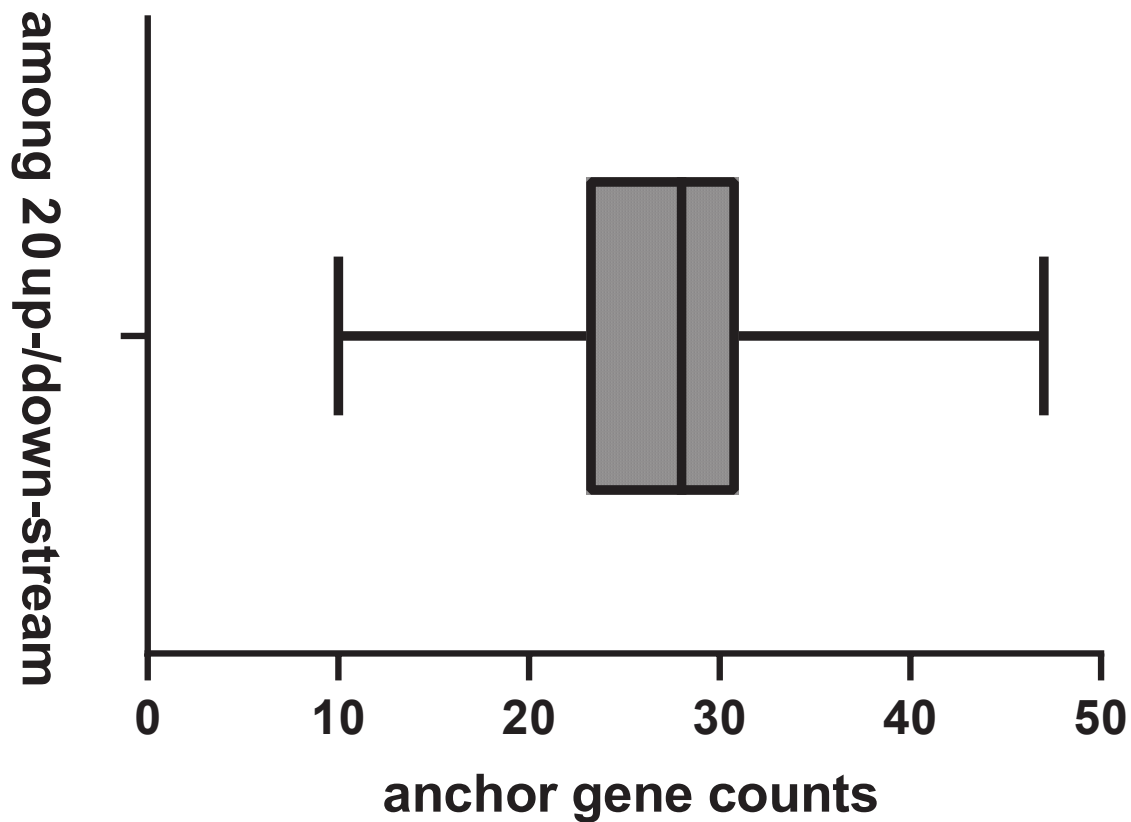

Supplement: Supplementary file 1 [file ijms-24-16313-s001.zip › Figure S11.pdf]

a

Oru Chr1:19568356-21576429

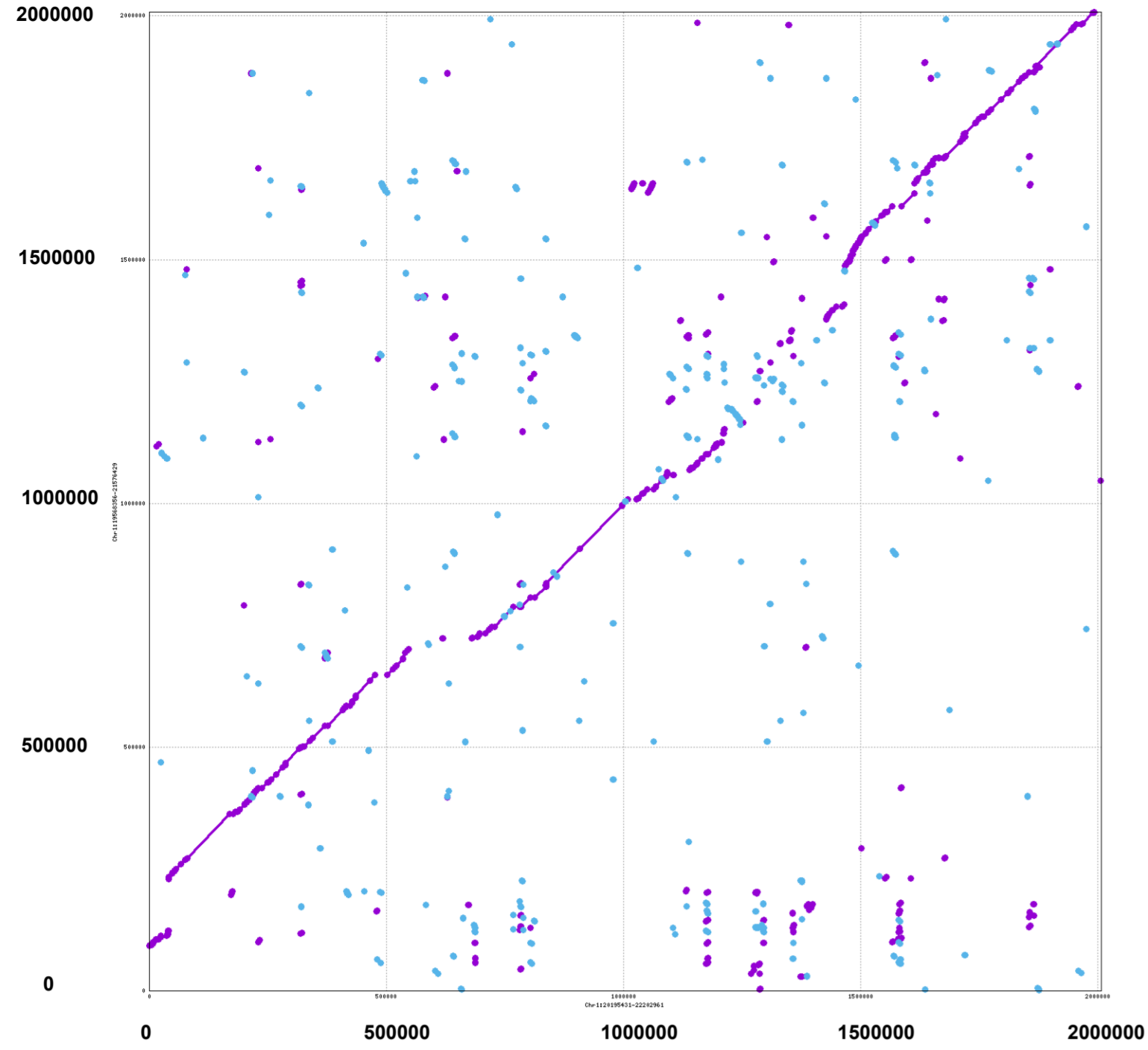

Osa Chr1:20195431-22202961

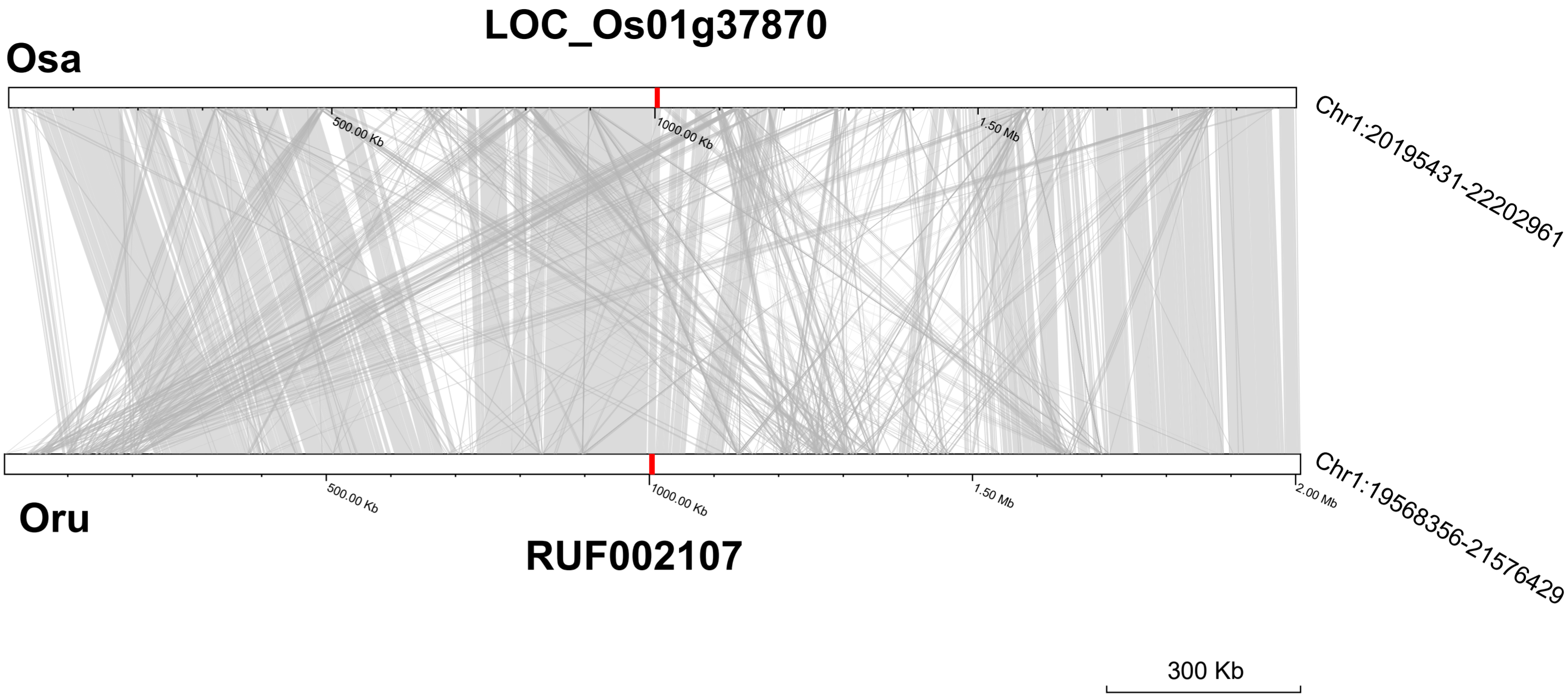

b

Oru Chr11:14810416-16813301

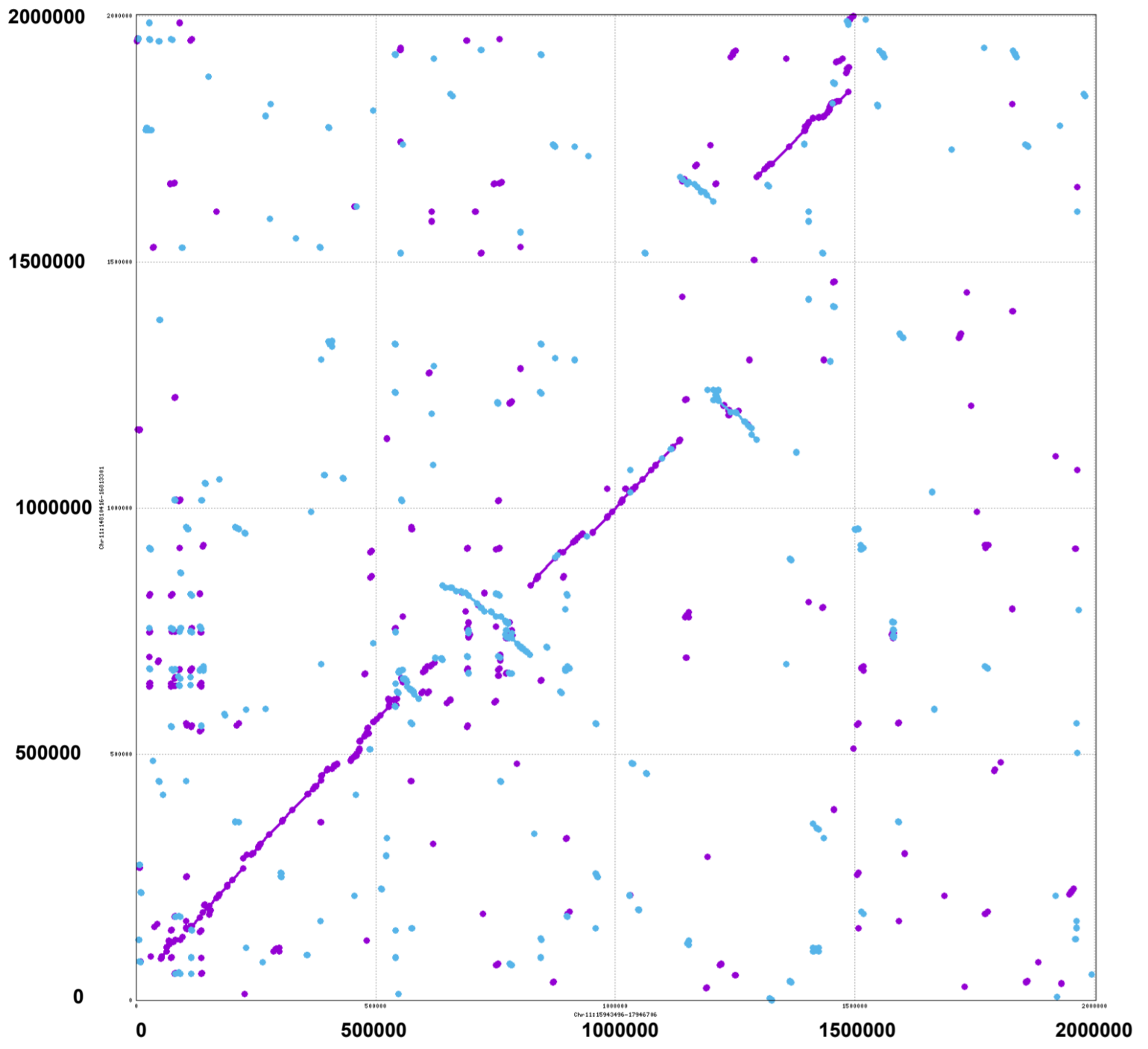

Osa Chr11:15943496-17946706

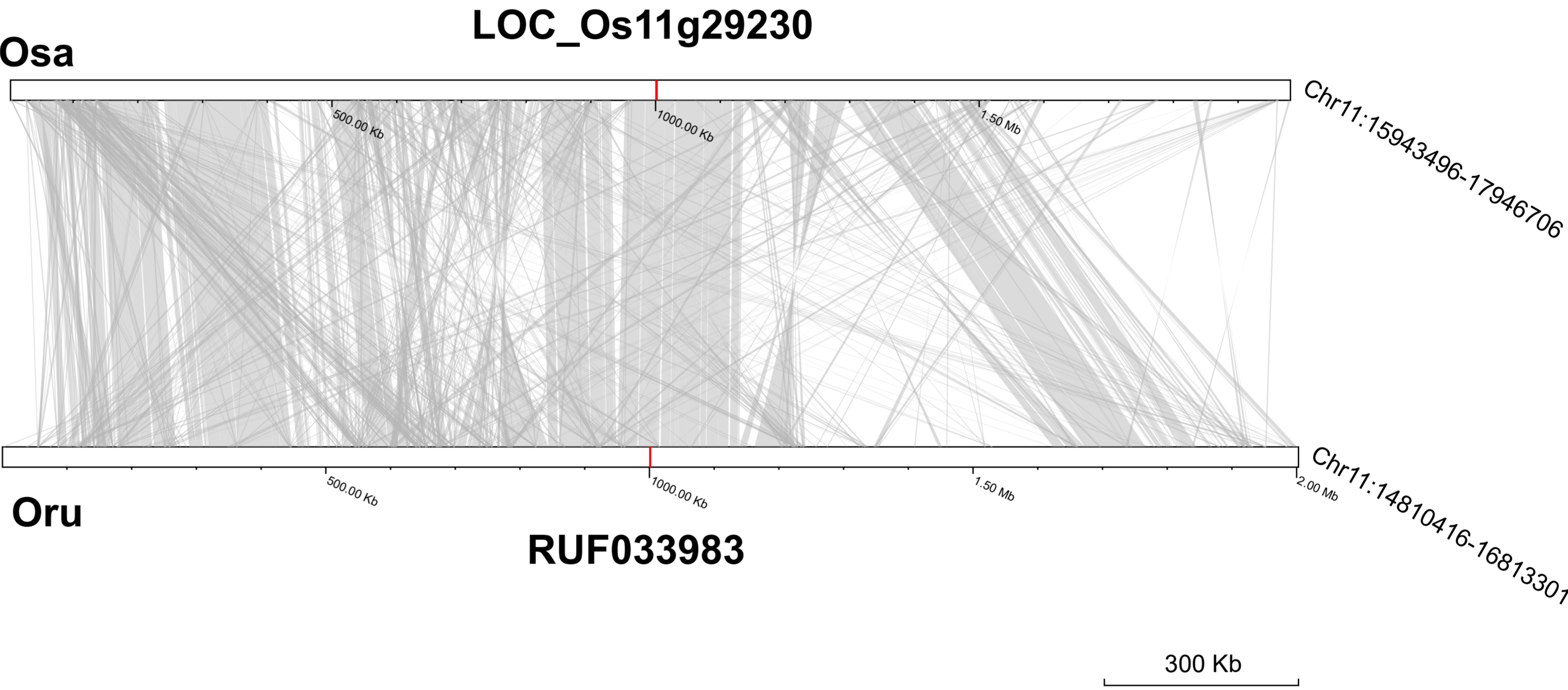

c

Oru Chr11:14903371-16904808

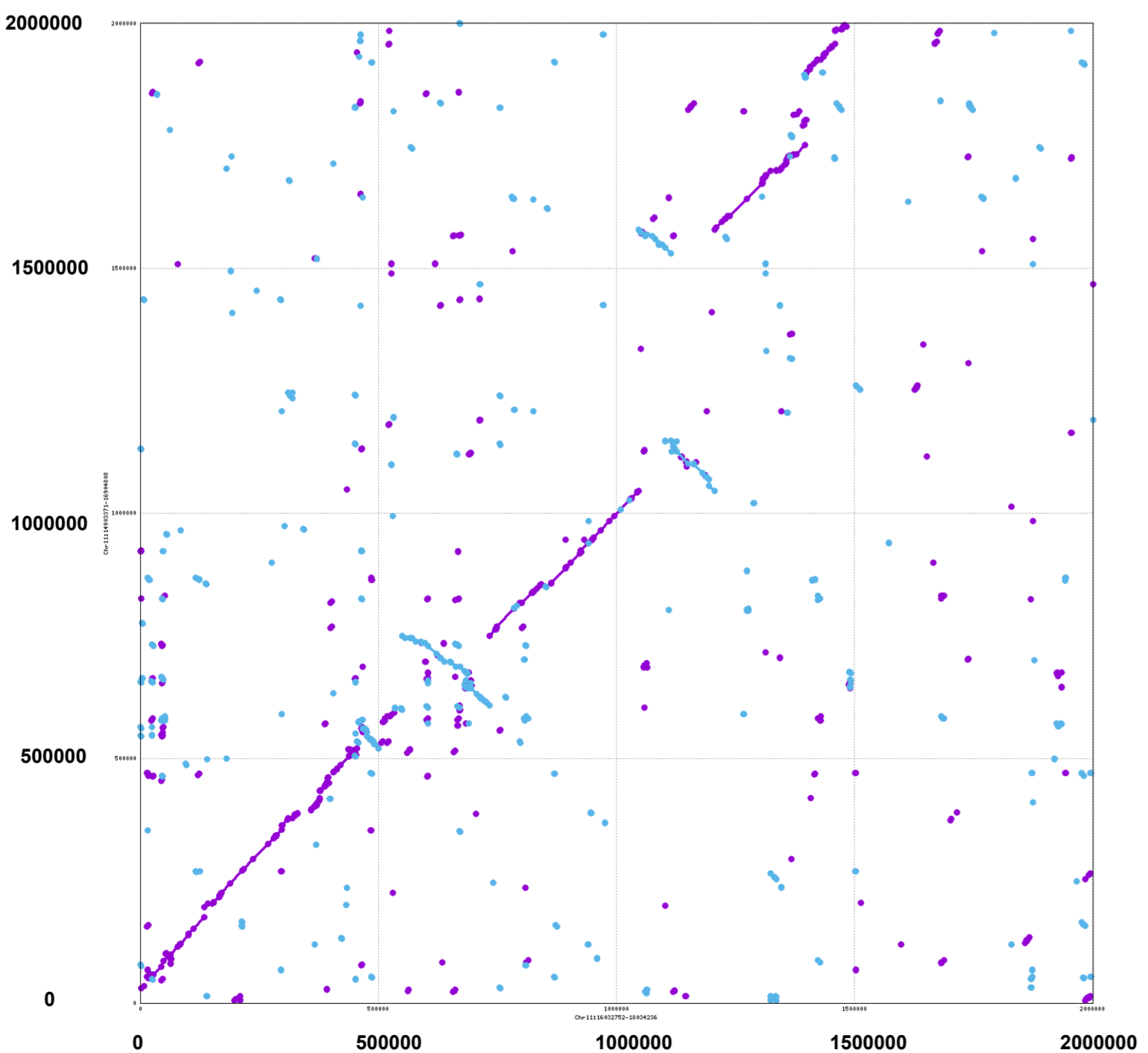

Osa Chr11:16032752-18034236

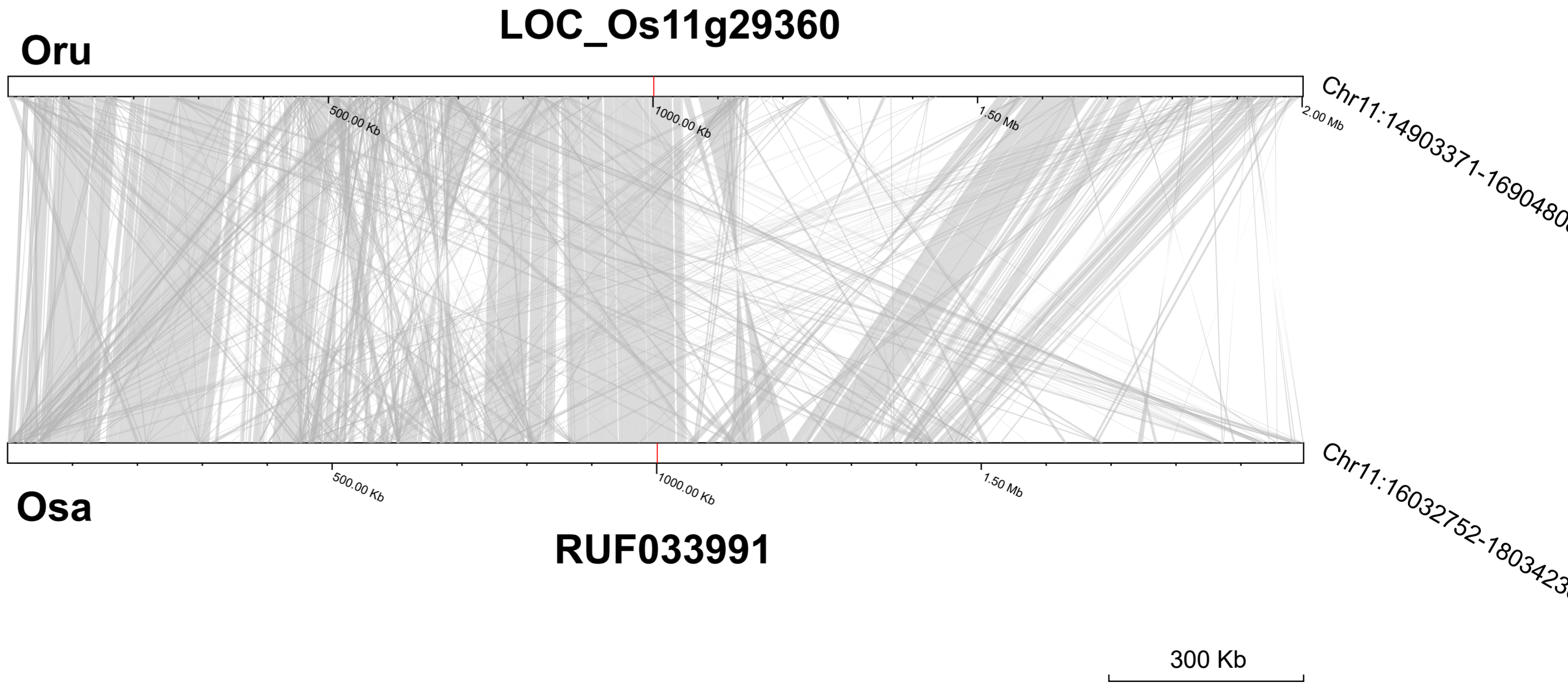

Supplement: Supplementary file 1 [file ijms-24-16313-s001.zip › Figure S12.pdf]

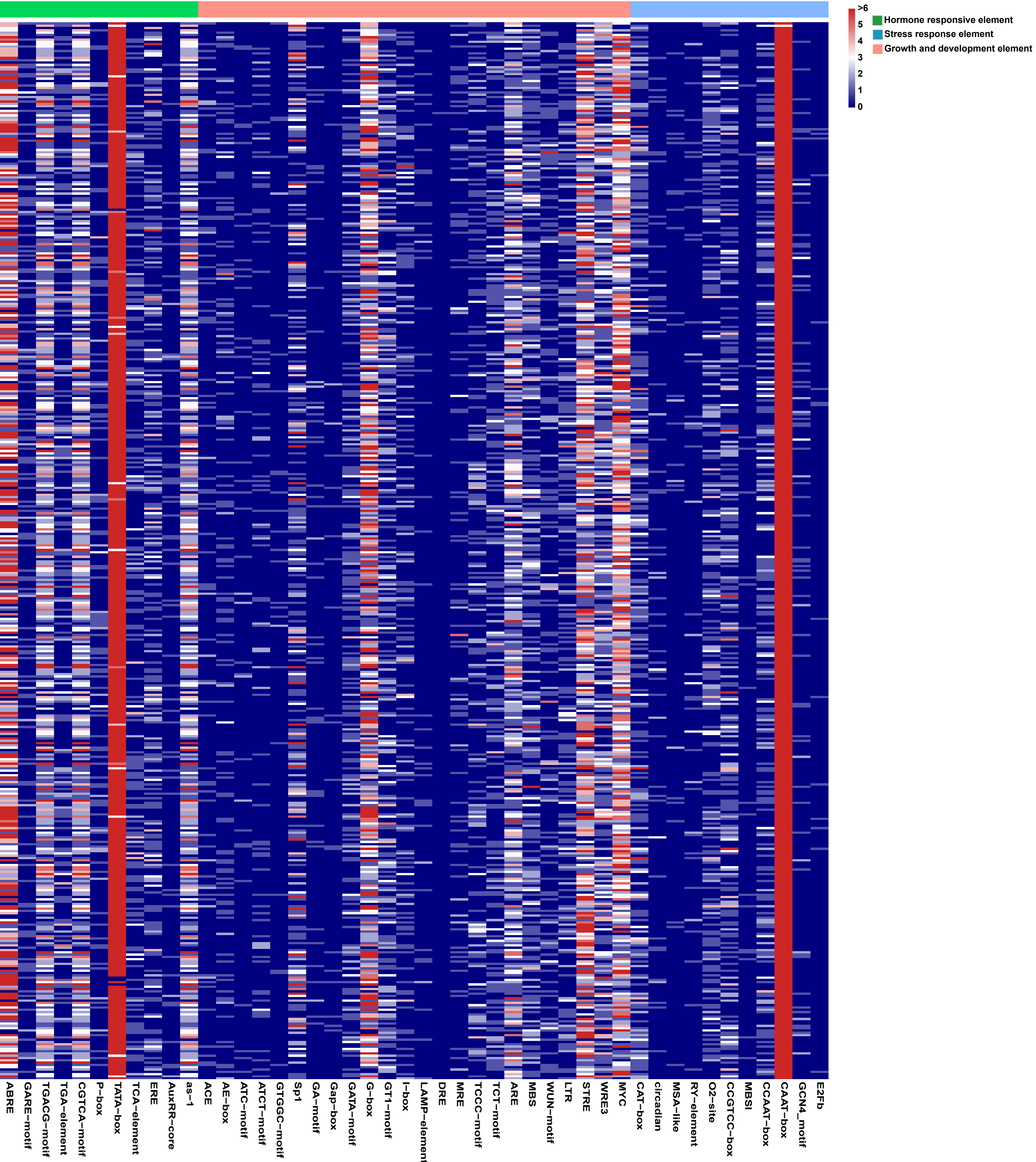

Supplement: Supplementary file 1 [file ijms-24-16313-s001.zip › Figure S2.pdf]

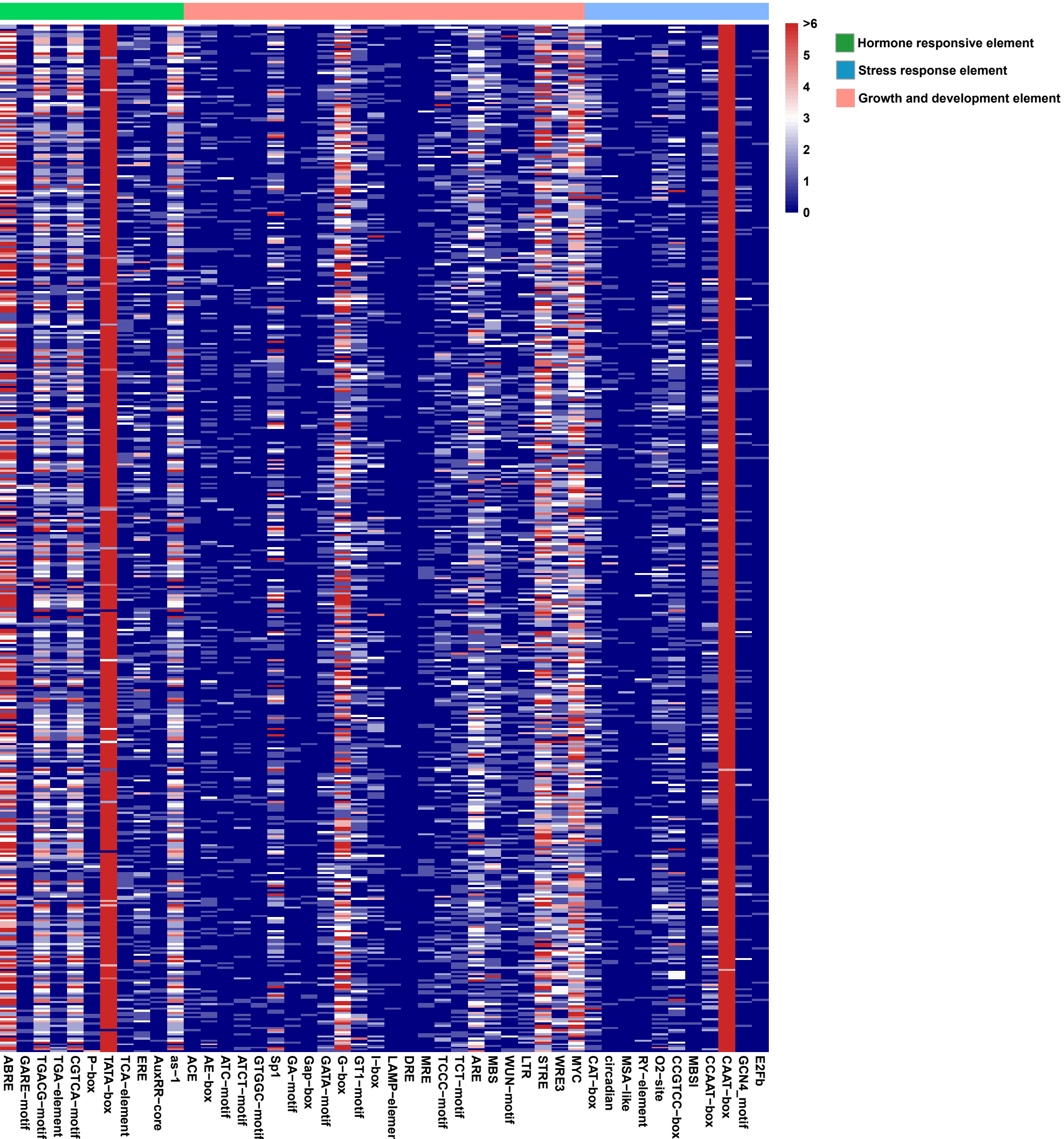

Supplement: Supplementary file 1 [file ijms-24-16313-s001.zip › Figure S3.pdf]

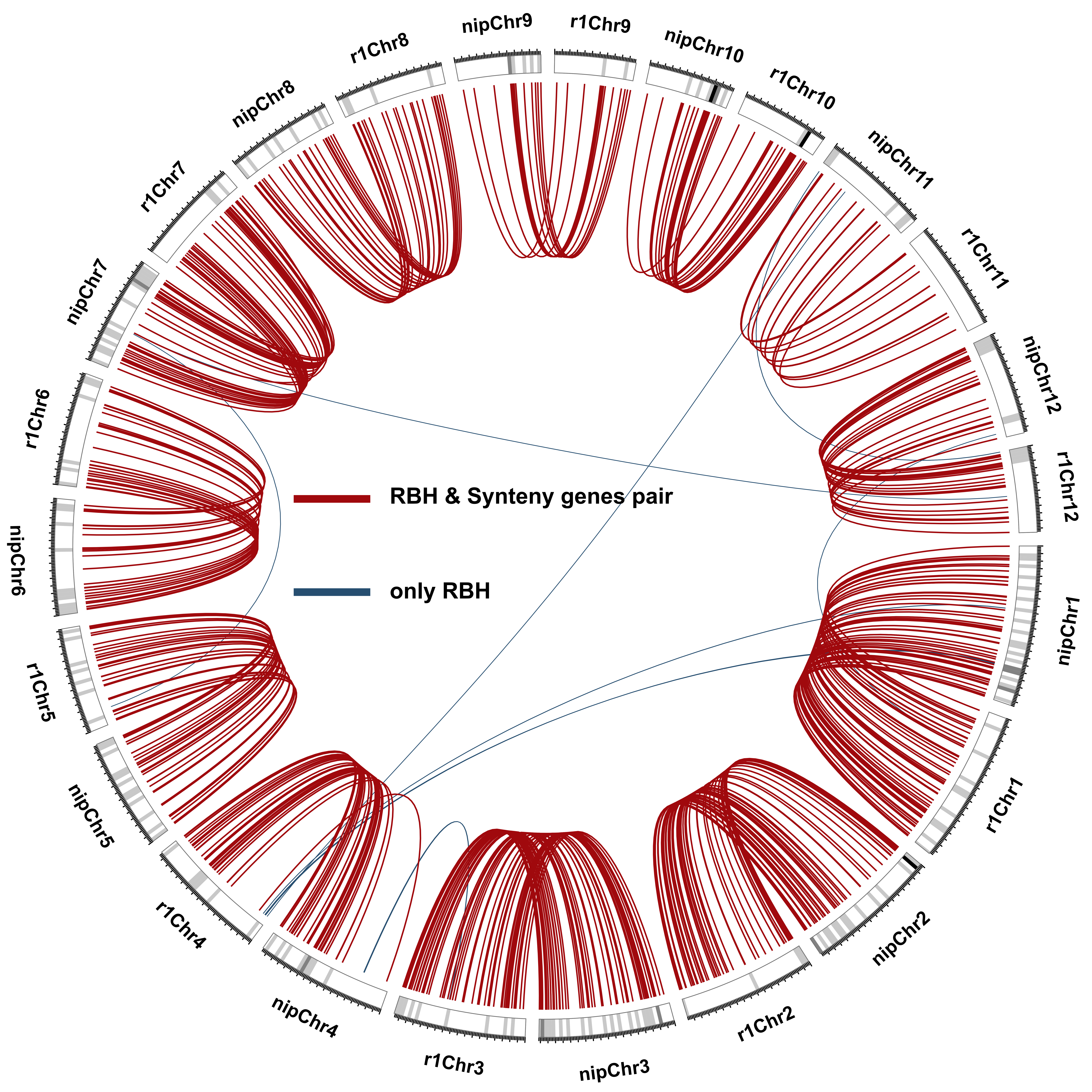

Supplement: Supplementary file 1 [file ijms-24-16313-s001.zip › Figure S4.pdf]

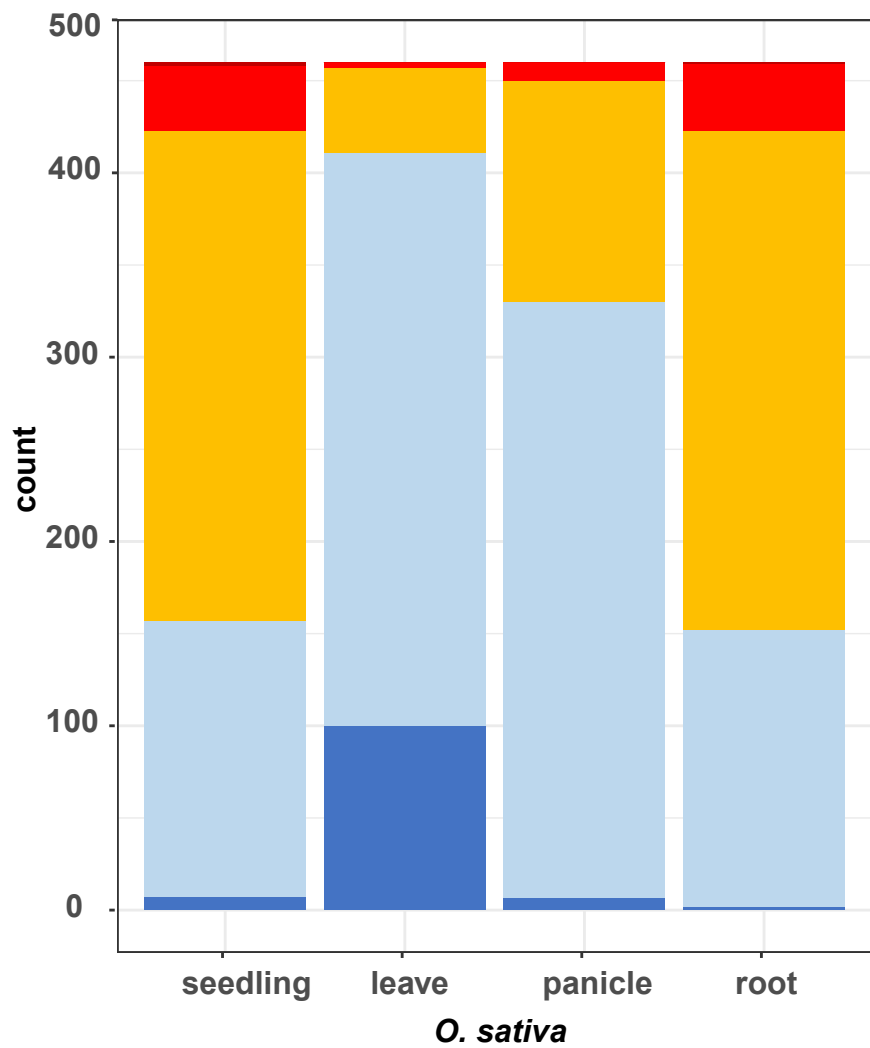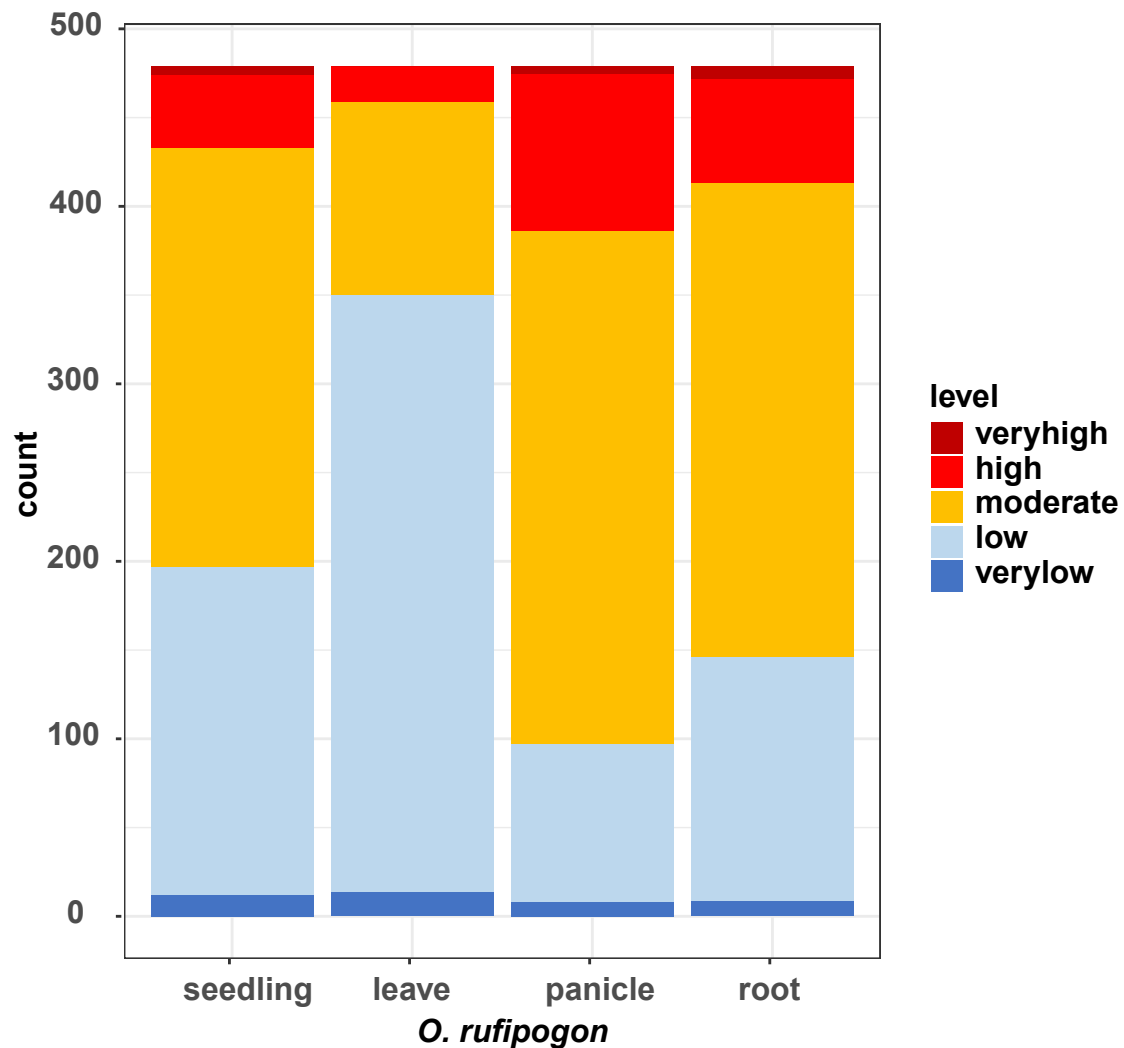

Supplement: Supplementary file 1 [file ijms-24-16313-s001.zip › Figure S6.pdf]

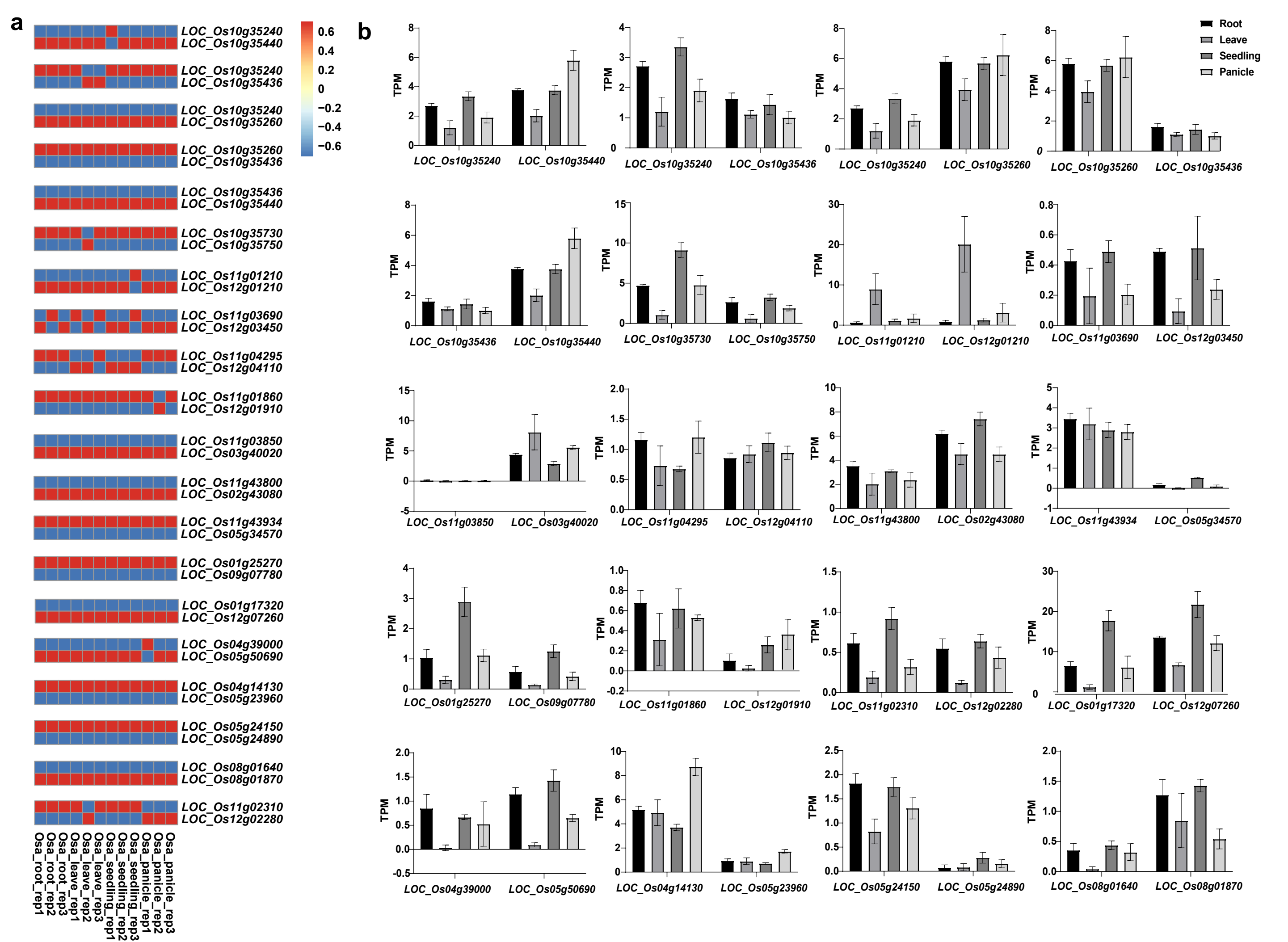

Supplement: Supplementary file 1 [file ijms-24-16313-s001.zip › Figure S7.pdf]

a

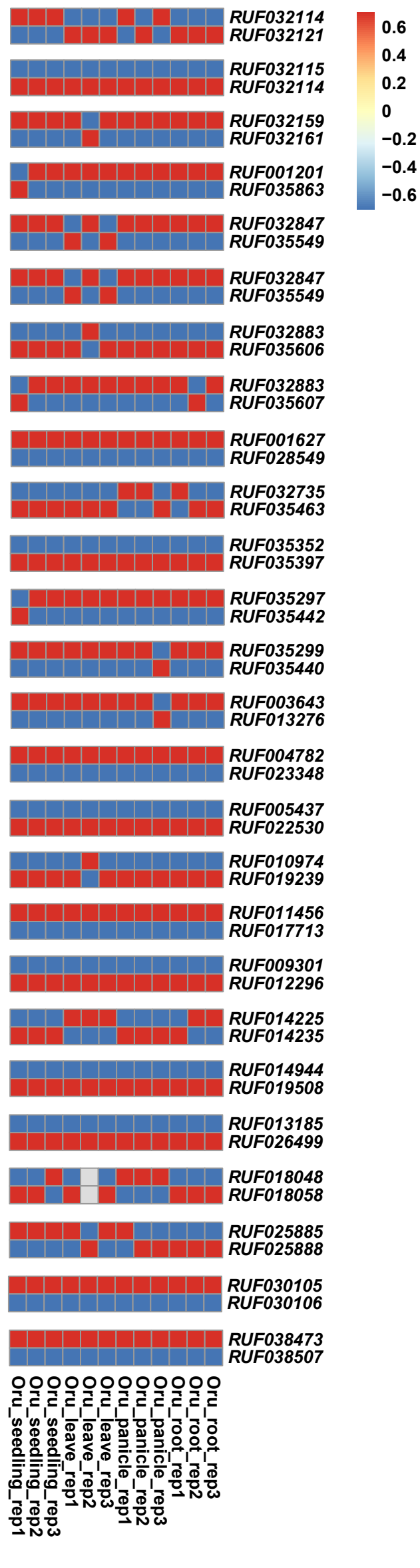

b

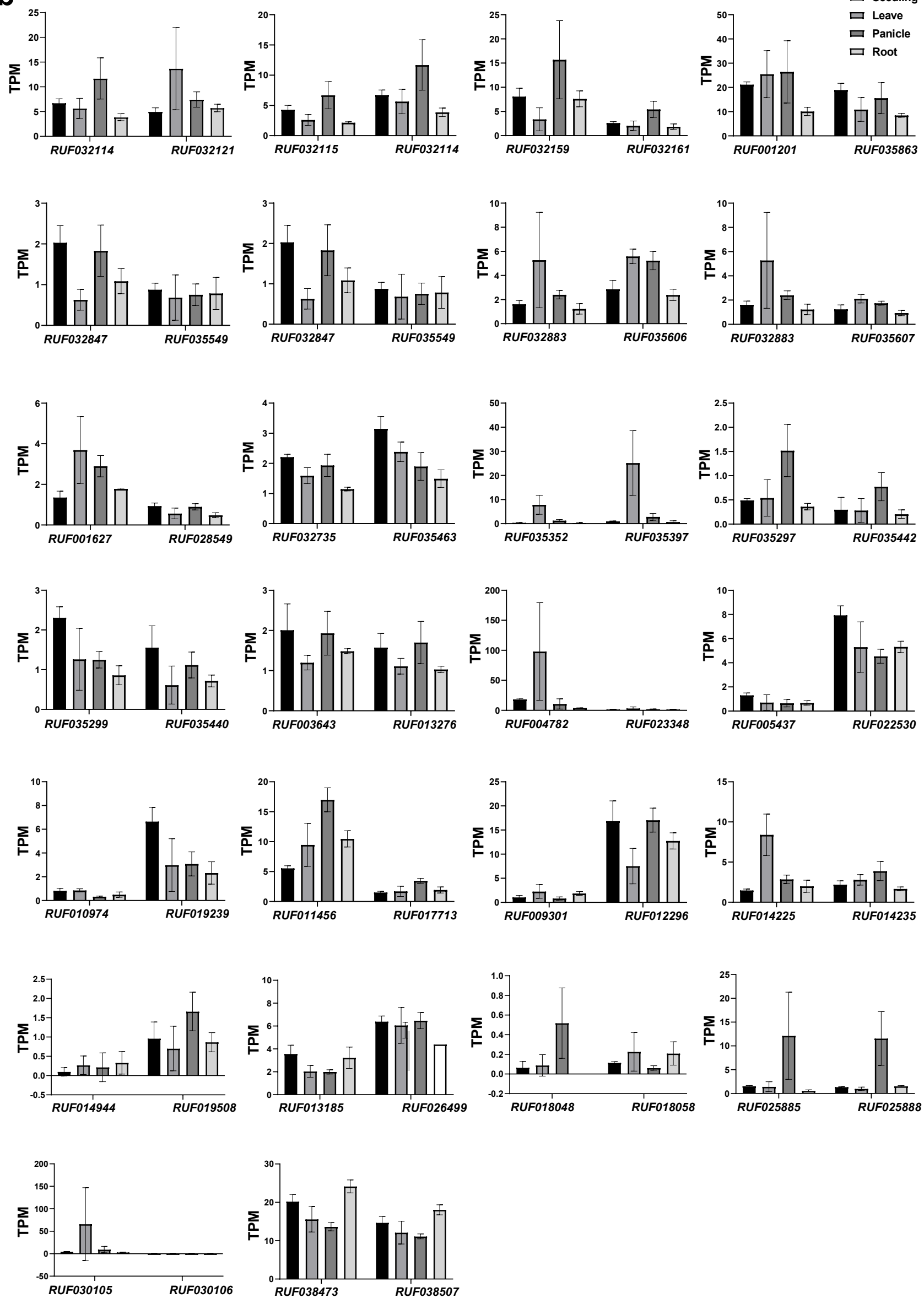

Supplement: Supplementary file 1 [file ijms-24-16313-s001.zip › Figure S8.pdf]

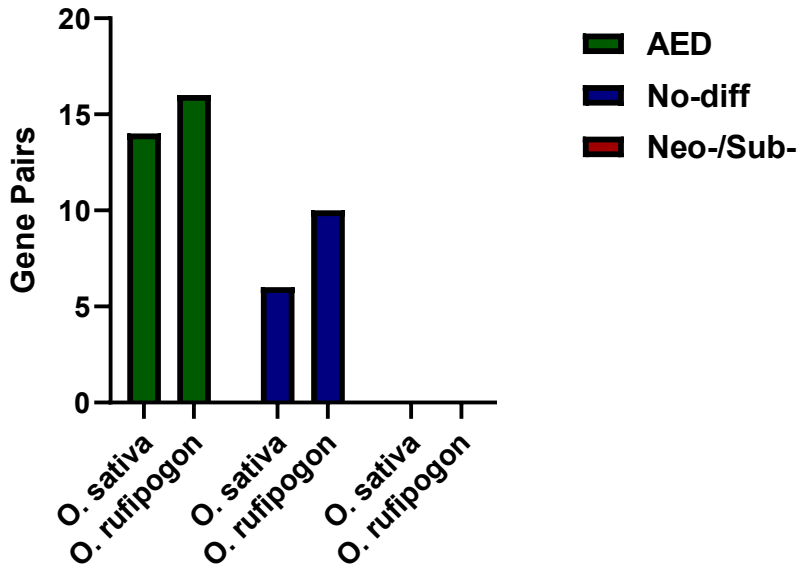

Supplement: Supplementary file 1 [file ijms-24-16313-s001.zip › Figure S9.pdf]
